# The National One Week Prevalence Audit of Universal Meticillin-Resistant *Staphylococcus aureus* (MRSA) Admission Screening 2012

**DOI:** 10.1371/journal.pone.0074219

**Published:** 2013-09-12

**Authors:** Christopher Fuller, Julie Robotham, Joanne Savage, Susan Hopkins, Sarah R. Deeny, Sheldon Stone, Barry Cookson

**Affiliations:** 1 University College London, London, United Kingdom; 2 Public Health England, London, United Kingdom; 3 Royal Free Hospital, London, United Kingdom; 4 Health Protection Agency, London, United Kingdom; Amphia Ziekenhuis, Netherlands

## Abstract

**Introduction:**

The English Department of Health introduced universal MRSA screening of admissions to English hospitals in 2010. It commissioned a national audit to review implementation, impact on patient management, admission prevalence and extra yield of MRSA identified compared to “high-risk” specialty or “checklist-activated” screening (CLAS) of patients with MRSA risk factors.

**Methods:**

National audit May 2011. Questionnaires to infection control teams in all English NHS acute trusts, requesting number patients admitted and screened, new or previously known MRSA; MRSA point prevalence; screening and isolation policies; individual risk factors and patient management for *all* new MRSA patients and random sample of negatives.

**Results:**

144/167 (86.2%) trusts responded. Individual patient data for 760 new MRSA patients and 951 negatives. 61% of emergency admissions (median 67.3%), 81% (median 59.4%) electives and 47% (median 41.4%) day-cases were screened. MRSA admission prevalence: 1% (median 0.9%) emergencies, 0.6% (median 0.4%) electives, 0.4% (median 0%) day-cases. Approximately 50% all MRSA identified was new. Inpatient MRSA point prevalence: 3.3% (median 2.9%). 104 (77%) trusts pre-emptively isolated patients with previous MRSA, 63 (35%) pre-emptively isolated admissions to “high-risk” specialties; 7 (5%) used PCR routinely. Mean time to MRSA positive result: 2.87 days (±1.33); 37% (219/596) newly identified MRSA patients discharged before result available; 55% remainder (205/376) isolated post-result. In an average trust, CLAS would reduce screening by 50%, identifying 81% of all MRSA. “High risk” specialty screening would reduce screening by 89%, identifying 9% of MRSA.

**Conclusions:**

Implementation of universal screening was poor. Admission prevalence (new cases) was low. CLAS reduced screening effort for minor decreases in identification, but implementation may prove difficult. Cost effectiveness of this and other policies, awaits evaluation by transmission dynamic economic modelling, using data from this audit. Until then trusts should seek to improve implementation of current policy and use of isolation facilities.

## Introduction

Due to the historically high prevalence, mortality and cost of healthcare associated meticillin resistant *Staphylococcus aureus* (MRSA) infection [1,2], legislation [3] and many national infection control interventions [4-6] were introduced in the English National Health Service (NHS). Several of these interventions were associated with subsequent reductions in MRSA [3]. Annual MRSA bacteraemia rates fell by 85% between April 2003 and March 2011 [7]. Preventing healthcare associated infections (HCAI) remains a national priority and the introduction of a zero tolerance approach (and a target of zero MRSA bacteraemias for all healthcare organisations) is a major requirement in the NHS Operating Framework 2012-13 [8].

The basis of reduction of transmission of MRSA is hand hygiene (6), isolation of MRSA positive patients, suppression/decolonisation therapy and screening for asymptomatic carriers [2,9,10]. United Kingdom (U.K.) national guidance in 2006, recommended “targeted screening” of individual patients in “high-risk” specialties (defined as Nephrology, Neurosurgery, Orthopaedics and Trauma, Haematology and Oncology, Vascular Surgery and Cardiothoracic Surgery) where infections were likely to be deep-seated and hard to treat [2] and/or screening of patients with known risk factors for MRSA carriage. Hospitals had discretion to implement these guidelines according to local circumstances.

The English Department of Health introduced universal mandatory MRSA screening of all elective admissions, except for paediatric, maternity and some day-cases (ophthalmology, endoscopy, minor dermatology) from April 2009 and of all emergency admissions to acute NHS hospitals from December 2010, on the basis of an impact assessment model of the cost-effectiveness of different screening and decolonisation strategies [11]. It is unclear, however, from the limited clinical studies performed in the UK [12-16] or from modelling studies [10,17-19] which of these two screening strategies (“targeted screening of admissions to high-risk specialties” or “universal”) is more clinically or cost-effective, or how they compare with checklist activated screening (i.e. assessing all admissions with a checklist of clinical risk factors for MRSA carriage and screening those with at least one risk factor). One modelling study reported little difference between long term prevalence levels achieved by these strategies but found that targeted and checklist activated screening were associated with substantial savings [19].

As the Department of Health’s impact assessment and other mathematical models of the effectiveness and cost-effectiveness of universal mandatory screening were not populated by representative data from NHS hospitals [10,17-19] the Department was committed to reviewing the effectiveness of the new policy. The National Audit Office [20] and the Parliamentary Accounts Committee [21] also called for a robust review of the implementation of the policy, its effectiveness and cost-effectiveness, and its impact on patients and their management. The English Department of Health, therefore, commissioned a national audit of MRSA screening with the following aims:

1 To report on current implementation of the policy of universal screening of all emergency and elective admissions.
2 To report the prevalence rates of MRSA carriage on admission in emergency and elective admissions and the proportion of carriage that was previously unknown.
3 To report on screening, swabbing, isolation and decolonisation policies and practices and laboratory methods.
4 To report on how patients were managed: how soon results were available, numbers of patients isolated and/or decolonised pre-emptively or after the result was known, numbers treated for MRSA infection.
5 To determine the extra yield of MRSA positive patients identified by universal admission screening, compared to: (a) Screening only admissions to “high-risk” specialties (b) “Checklist activated screening” of all admissions (c) Screening all admissions to high-risk specialties plus checklist activated screening for all other admissions.

## Methods

### Ethics Statement

The National Research Ethics Service considered the study to be an audit and that it therefore did not require formal ethical approval.

### Study Design

A national one-week prevalence audit of MRSA screening was carried out through a questionnaire (see File S1) sent to infection control teams in all 167 English NHS acute trusts (a trust being the administrative unit of a small number of acute hospitals) at the end of April 2011, for completion in the audit week of 9-15^th^ May 2011.

The questionnaire was piloted in 10 trusts in early 2011 for face-validity and feasibility of data collection with further changes made following focus groups with representatives from 125 hospital infection control teams in nine regional meetings.

The questionnaire requested data on:

1 The number of elective, emergency and day-case patients admitted and screened in the week of 11^th^-17^th^ April and the numbers admitted to the “high-risk” specialties defined as per previous guidelines [2](nephrology, neurology, trauma & orthopaedics, haematology & oncology, vascular surgery and cardithoracic surgery). ITU was not included as a “high-risk” specialty for the purposes of data collection since patients are classified on database systems according to their admitting specialty and not whether they were admitted to ITU.
2 The numbers of new and previously known MRSA positive screens that week.
3 Local screening, isolation, decolonisation practices, routine laboratory methods and costs.
4 Point-prevalence of MRSA colonised and infected patients in each trust on Wednesday 11^th^ May 2011 and whether they were isolated or not.
5 Individual patient level data for *all* newly identified MRSA positive patients and a random sample of 5-10 MRSA negative patients detected on admission or at preadmission clinics during the audit week of 9^th^-15^th^ May: age, specialty (high-, or low-risk), acute-, or elective-screen, date of screen, admission and discharge, time to result, pre-, and post-result management (isolation and decolonisation) and the presence of well- recognised [12] risk factors for MRSA carriage on a six item checklist (admission to the trust within the last year, admission to any other trust in the last year, transfer from another hospital, care home resident, presence of indwelling devices/presence of skin breaks). For MRSA negative patients, previous history of MRSA carriage was also sought.

The random sample of MRSA negative patients was generated by consecutive numbering of all such patients that week and using an online research randomiser tool (http://www.randomizer.org/form.htp) to select 5-10 MRSA negative patients. Infection control teams were asked to make all reasonable efforts to interrogate relevant databases, review medical and nursing notes, and, if possible, the patient, and discuss with nursing staff to clarify uncertainties. Patients were regarded as checklist positive if they had at least one risk factor. If there was no documented, clinical or database evidence of these risk factors, it was assumed that they were not present.

Simple descriptive statistics were used to describe the results for all acute trusts combined and for different trust types, which were defined according to standard definitions used in mandatory reporting as “acute” (ie middle sized, general hospitals providing services for local populations), “teaching” (ie larger hospitals, providing medical training and general services locally plus more specialised services regionally), “specialist” (ie smaller single specialty hospitals providing services at a regional and/or national level).

When calculating the yield of different screening strategies, numbers were derived from questionnaire data (average number of newly identified and previously known MRSA positive admission screens per trust divided by the average numbers of MRSA screens). Numbers were broken down by trust type, specialty and admission type (emergency or elective). Proportions of admissions that were expected to be checklist positive were derived from the individual level patient data collected from MRSA positive and MRSA negative patients.

## Results

### Response rate

Responses were received from 144/167 (86%) trusts (comprising 19/20 (95%) specialist trusts, 23/26 (89%) teaching and 100/121 (83%) acute trusts). Data were also received from two trusts that did not include their unique identifier; their data are therefore included only when data are presented for “all trusts”.

Data on isolation, decolonisation, screening policies and laboratory methods were provided by 143/144 trusts.

Individual patient data on risk factors and management of 951 randomly selected patients screening negative for MRSA in the audit week was collectively provided by 141/167 trusts (84%) (mean [standard deviation] 6.7 [± 2.5] per trust). 131/167 (78%) trusts provided data on 760 MRSA patients newly identified by screening that week (mean per trust 5.3 [±4.96]). Eleven trusts (7%) reported no newly identified MRSA positive patients in that week.

### Implementation of national universal MRSA screening policy across the NHS

Table 1 shows that for the NHS as a whole, 82% of elective, 61% of emergency and 48% of day-case admissions were screened (median and interquartile range presented in Table 1). However, screening coverage of day-case patients in Specialist trusts was greater, with 74% being screened.

**Table 1:** Mean and median (inter-quartile range) proportion per trust of admissions screened for MRSA.

**Admission category**	**All trusts^1^**	**Acute Trusts** ^2^	**Specialist**	**Teaching** ^2^
Emergency	**60.6%** (52,788/87,165) 130 trusts Median 67.3% IQR (47.5%-85.8%)	**60%** (38,127/63,577) 91 trusts Median 67.1% IQR (47.4-85.8%)	**56.4%** (657/1,166) 17 trusts Median 85.9% IQR (68.3-100%)	**62.5%** (13,736/21,988) 21 trusts Median 59.4% IQR (48.9-89.2%)
Elective^3^	**81.8%** (22,773/27,838) 115 trusts Median 59.4% IQR (48.9-89.2%)	**87.7%** (14,477/16,497) 77 trusts Median 92% IQR (59-136%)	**75.4%** (1652/2,191) 16 trusts Median 86% IQR (62-100%)	**72.6%** (6,569/9,044) 20 trusts Median 73% IQR (30-102%)
Day-cases^4^	**47.9%** (22,416/46,777) 110 trusts Median 41.4% IQR (23.2-78.9%)	**43.3%** (14,255/32,927) 77 trusts Median 36.5% IQR (17.4-73.9%)	**73.5%** (1,153/1,568) 13 trusts Median 67.3% IQR (42.6-100%)	**57.8%** (6,894/1,1927) 19 trusts Median 48.3% IQR (36.1-77.7%)

^1^ Two trusts did not include their unique identifier; their data are therefore included under “all trusts”; one of these did not return day-case data and one did not return emergency data.

^2^ Median values may exceed 100% due to the numerator including pre-admission screens from patients not admitted in that week and the denominator including patients who were admitted but not screened in that week (i.e. screened at a pre-admission clinic in a previous week).

^3^ not including day-cases

^4^ not including dermatology, endoscopy, ophthalmic and paediatrics

### Prevalence of MRSA carriage on admission

The overall prevalence of MRSA on admission was 1.4% (1420/102,397) (median 1.1%, IQR 0.8%-1.9%): 2.1% for emergency admissions, 0.9% for elective admissions and 0.7% for day-case admissions (Table 2). The prevalence of newly identified MRSA was approximately half of this at 0.8% (overall): 1% (emergency), 0.6% (elective) and 0.4% (day-cases).

**Table 2:** Mean and median (inter-quartile range) proportion per trust of admission screens that were MRSA positive (all MRSA positives and newly positive for MRSA).

**Admission Category**	**MRSA +ves**	**All trusts**	**Acute**	**Specialist**	**Teaching**
Emergency	Total	**2.1%** (1,075/52,064 in 129 trusts) Median 1.6% IQR (1.1-2.7%)	**2.2%**(836/37,408 in 90 trusts) Median 2% IQR (1.2-2.7)	**1%** (5/652 in 16 trusts) Median 0% IQR (0-0.2%)	**1.7%** (230/13,736 in 22 trusts) Median 1.7% IQR (1.1-2.4%)
	New	**1%** (498/50,739 in 127 trusts) Median 0.9% IQR (0.4-1.3%)	**1%** (374/36,083 in 88 trusts) Median 1.0% IQR (0.5-1.5%)	**0.6%** (4/652 in 16 trusts) Median 0% IQR (0-0%)	**0.9%** (119/13,736 in 22 trusts) Median 0.8% IQR (0.5-1.3%)
Elective^1^	Total	**0.9%** (188/20,798 in 101 trusts) Median 0.7% IQR (0-1.9%)	**0.8%** (110/13,532 in 68 trusts) Median 0.7% IQR (0-2.5%)	**1.7%** (25/1,488 in 15 trusts) Median 0.5% IQR (0.3-1.5%)	**0.9%** (53/5,703 in 17 trusts) Median 0.6% IQR (0.5-1.1%)
	New	**0.6%** (107/19,283 in 98 trusts) Median 0.4% IQR (0-1.2%)	**0.5%** (68/12,953 in 68 trusts) Median 0.4% IQR (0-1.5%)	**1.2%** (16/1,346 in 14 trusts) Median 0.5% IQR (0-1.4%)	**0.5%** (23/4,909 in 15 trusts) Median 0.5% IQR (0-1.4%)
Day-case ^2^	Total	**0.7%** (150/21,501 in 112 trusts) Median 0% IQR (0-1%)	**0.4%** (58/13,509 in 76 trusts) Median 0% IQR (0-0.6%)	**0.6%** (6/1,062 in 16 trusts) Median 0% IQR (0-1.1%)	**1.2%** (85/6,816 in 19 trusts) Median 0.7% IQR (0.3-1.2%)
	New	**0.4%** (79/20,461 in 110 trusts) Median 0% IQR (0-0.1%)	**0.2%** (27/12,469 in 74 trusts) Median 0% IQR (0-0.2%)	**0.5%** (5/1,062 in 16 trusts) Median 0% IQR (0-0%)	**0.7%** (47/6,816 in 19 trusts) Median 0.1% IQR (0-0.7%)

^1^ Not including day-cases

^2^ not including dermatology, endoscopy, ophthalmic paediatrics.

The number of patients needing to be screened for identification of one new MRSA positive patient was 102, 180 and 259 for emergency, elective and day-cases respectively (Table 3). Screening efficiencies varied by trust type and type of admission screened: the most efficient being screening of electives in Specialist trusts, requiring only 84 screens for one new MRSA positive identification, while day-cases in Acute trusts required 462 screens to detect one new MRSA positive patient.

**Table 3:** Numbers needed to screen to identify one new MRSA case.

	**All trusts**	**Acute**	**Specialist**	**Teaching**
Emergency	102 (50,739/498)	97 (36,083/374)	163 (652/4)	115 (13,736/119)
Elective (not including day-cases)	180 (19,283/107)	191 (12,953/68)	84 (1,346/16)	213 (4,909/23)
Day-cases	259 (20,461/79)	462 (12,469/27)	212 (1,062/5)	145 (6,816/47)

### Screening, isolation and decolonisation policies and practices, and laboratory methods

Screening policies: Only 10% (14/143) of trusts (all 14 being specialist trusts) screened all patients on admission, while the majority exempted all or some of the following categories as per Department of Health advice: low-risk paediatrics or endoscopy cases, dermatology, ophthalmic and dental day-cases.

Isolation and decolonisation policies: The use of pre-emptive isolation for patients with a past history of MRSA or of patients in locally defined high-risk specialties or categories was reported by 77% and 44% of trusts respectively (Table 4). Pre-emptive decolonisation of these patients was reported by 35% and 41% of trusts respectively.

**Table 4:** Trusts initiating precautions on admitted patients before MRSA results available (n= 143 trusts; more than one response possible).

	**Isolation**	**Contact precautions ^1^**	**Decolonisation**
All patients	2 (1.4%)	30 (21%)	14 (10%)
All previous MRSA +ves	110 (77%)	85 (59%)	50 (35%)
High-risk patients/wards	63 (44%)	33 (23%)	59 (41%)
Other	12 (8%)	5 (3.5%)	3 (2%)
None	10 (7%)	20 (14%)	49 (34%)

^1^ i.e. disposable gloves and aprons

Laboratory methods: The most common technique reported for routine processing of admission swabs was a chromogenic agar plating for both emergencies (116 of 142 responding trusts: 81.7%) and electives (123/141: 87.2%). PCR for routine processing of emergency admission screens was reported in 7/142 responding trusts (5%) and in 1/141 (0.7%) for elective admissions.

Further details of policies, decolonisation regimes and laboratory methods are reported in the (File S2).

### Patient management – point prevalence data

On the day of the point-prevalence audit 140 trusts collectively reported that 3.3% (3,076/92,619) of all inpatients in the hospital were known to be MRSA positive [median 2.9%, IQR 1.8-4%]. By trust type this proportion was 3.6% (2191/61628 patients) [median 3.6%, IQR 2.1-4.8%] for acute trusts, for teaching trusts 3.0% (758/25146 patients) [median 3.1%, IQR 1.9-3.8%] and for specialist trusts 2% (65/3285 patients) [median 1.4%, IQR 0.9-3.4%].

In the 139 trusts reporting data on intervention measures, 67% (2,026/3,033) of MRSA patients were isolated. Of these, 91% (1,837/2,026) were in side-rooms, 4% (82/2,026) in a designated ward and 5% (107/2,026) in a cohort bay. The remaining third were not isolated. In specialist trusts isolation rates were greater with 98.5% (64/65) of MRSA positive in-patients being isolated. In addition, 127 trusts reported that 11% (286/2,680) of MRSA positive inpatients were receiving antibiotic treatment for MRSA infection on that day [median 9.1%, IQR 0-16.7%].

### Patient management – individual patient data

In addition to the point-prevalence audit, individual patient management data were available for the group of 760 newly identified MRSA positive patients and for the group of 951 randomly sampled patients screening negative for MRSA. Checklists for risk factors for MRSA colonisation were completed (or partially completed) for all patients. In total, 63% MRSA positive patients (458/760) and 50.5% of MRSA negatives (481/951) were checklist positive (i.e. had at least one risk factor documented). However, data on whether the patient had a skin break or an in-situ device were commonly missing. Among MRSA positives 237/760 (31%) and 234/760 (31%) were missing data on the presence of skin-breaks and/or in-situ devices respectively. For MRSA negative admissions the corresponding proportions were 343/951 (36%) and 315/951 (33%).

Mean sample turn-around time (the time between swabbing and the result becoming available) was 2.87 (±1.33) days for MRSA positive results and 1.75 (± 0.9) days for MRSA negative results.

Individual patient management data showed that 78% (596/760) of newly identified MRSA positive patients and 67% (640/951) of the MRSA negative patients were admitted in the same week that they were screened, the remainder having been screened in pre-admission clinics. As shown in Table 5, 16% of the newly identified MRSA positive patients were pre-emptively isolated on admission, 6% were pre-emptively decolonised, and 37% were discharged before their screen result was available. Once the result was known, 55% of those newly identified positive patients who were still in-patients were isolated. Similarly, a third (33%) of the admitted MRSA negative patients were discharged before their screening result was available.

**Table 5:** Patient management of admitted new MRSA positives and MRSA negatives.

	**MRSA +ves n= 596**	**MRSA –ves n= 640**
Proportion isolated pre-emptively on admission	16% (93/596)	6% (38/640)
Proportion decolonised pre-emptively on admission	6% (36/596)	9% (59/640)
Proportion discharged before result available	37% (219/596)	33% (213/640)
Proportion of MRSA positive admissions who were still in-patients and who were isolated after result known	55%(205/376^1^)	N/A
Proportion of MRSA positive admissions who were still in-patients and who were decolonised after result known	97% (363/376)	N/A

^1^ data missing for one admission

### The extra yield of MRSA positive patients using universal admission screening compared to other screening strategies

Table 6 compares the number of screens that would be performed in an “average” trust each week using four different screening strategies, and estimates the number of MRSA positive patients that would be identified by each strategy.

**Table 6:** The yield of MRSA achieved by universal admission screening for the average NHS trust compared to three other screening strategies.

	**Screen all admissions**	**Checklist activated screening (CLAS**)** all admissions**	**Screen only high-risk specialty admissions**	**Screen high-risk specialty admissions + CLAS for low-risk speciality admissions**
No. of screens per week	790	398	87	448
Total no. MRSA positives identified	11.3	9.1	1.0	9.3
Percentage of admissions screened	100%	50%	11%	56%
Percentage of MRSA positives identified	100%	81%	8.5%	82%

Compared to universal screening of all admissions, checklist activated screening would detect 80% of the MRSA positive patients (equivalent to an average of two fewer MRSA patients detected/week/trust) whilst halving the number of screens (Table 6). Results were similar across each trust category.

Screening high-risk specialty admissions only would detect less than 10% of MRSA positive patients whilst reducing screening by nearly 90%, although the reduction in screening would be less, 73% and 80% in specialist and teaching trusts respectively, and the detection of MRSA would be higher at 24% and 16% respectively.

Combining screening of all admissions to high-risk specialties with checklist activated screening for all other admissions would yield similar results to using checklist activated screening for all admissions. Again results were similar across trust category, although the reduction in screening would be lower (30%) in specialist trusts and detection of MRSA would be slightly higher (89%) in acute trusts.

## Discussion

This study, with its high response rate (86%), provides a more complete and representative picture of MRSA screening practice in the current NHS in England than is available from the literature on implementation, detection rates, potential yield from different screening strategies and management of patients.

There were three main findings. Firstly, implementation of universal mandatory admission screening was poor for emergency admissions (61%) and eligible day-case admissions (47%), although better for electives (81%). Secondly, the prevalence of MRSA colonisation on admission was low at 1.5% (overall), 2.1% (emergency), 0.9% (electives) and 0.7% (day -cases). Approximately half of these were already known to be MRSA positive. The point prevalence of MRSA amongst in-patients was 3.3%. Numbers needed to screen in order to identify one new positive were, therefore, high (ranging from 97 for emergency admissions in acute trusts to 462 for day-case admissions in acute trusts). Thirdly, checklist activated screening would detect 80% of the MRSA positive patients detected by universal screening of all admissions, whilst halving the number of laboratory screens. Screening high-risk specialty admissions would detect only 10% of MRSA positive patients (more in teaching and specialist trusts) whilst reducing screening by 90%.

Other important findings concern patient management, with over three quarters of trusts reporting a policy of pre-emptive isolation for patients with previous MRSA and nearly half reporting a policy of pre-emptive isolation for admissions to high-risk specialties. In practice, however, although most patients with MRSA received decolonisation, isolation of positive patients was limited. A third were discharged before their screen result was available and nearly half of those with newly identified MRSA who remained in hospital after the result was known were not isolated, and nor were a third of those with MRSA on the day of the in-patient point prevalence study. An exception was specialist trusts where nearly all were isolated due to the lower prevalence.

Comparison of our findings to those from outside the English NHS system or Republic of Ireland is hampered by a lack of comparable studies in the literature [22-27] because of large differences in their settings and healthcare systems. This includes the political imperative in the UK to reduce MRSA infections, which led to legislation and a multitude of national infection control interventions. Many of these preceded the introduction of universal admission screening, during which time MRSA rates fell considerably [3,7]. Comparison with some of the studies outside the UK is also difficult because they focussed on comparing PCR screening to more traditional culture methods [22,23,26]. However, we found PCR to be little used in England, which may due in part to a cluster randomised controlled trial in the NHS that failed to show its clinical benefit over chromogenic agar plating for universal screening [14].

The major findings of this audit differed from the only comparable multi-hospital study within the UK, the Scottish Pathfinder study [28-31]. While both studies reported that approximately half the admission prevalence was made up of newly identified MRSA positives [28,29], implementation of universal admission screening was lower in our study compared to the Pathfinder, where 85% of emergency and 98% of elective admissions were screened. This may reflect the fact that for Pathfinder, universal admission screening had been implemented as part of a specific year-long (2008-9) research study in 6 hospitals in three NHS “Boards” with a team of research nurses following admissions and reminding wards to screen if they had overlooked individual patients.

The overall admission prevalence of MRSA on admission (1.5%) is lower than that reported in Pathfinder (2.4%) (20) and a study in a single English hospital [12]. This may reflect a temporal decline of MRSA bacteraemias in England [32] with a 38% decrease reported between April 2009 and June 2011 [32]. A parallel reduction in MRSA colonisation was reported in the Pathfinder report (5.5% to 3.5% between 2008 and 2009) and in acute care of the elderly wards in an Irish teaching hospital (from 9% to 2% between 2007 and 2010 [15]).

The proportion of MRSA positive patients who would be identified by checklist activated screening (81%) was the same as that reported in Pathfinder [33] and in a study from an acute English trust in 2007 [12], despite differences between checklists. The proportions of admissions that would be checklist positive and therefore require screening were also similar (50% in this National One-Week (NOW) audit study and 57% in the Scottish Pathfinder and the acute English trust studies) [12,33]. Pathfinder also examined use of a short three item checklist (previous history of MRSA, presence of wounds or indwelling devices, and admissions not from home). They reported that this would detect 50% of MRSA positive patients but require only 10% of admissions to be screened. When we applied this checklist to our data we found it detected 68% of MRSA positive patients but that 26% of all admissions would require screening. When comparing the two studies it should be noted that the Pathfinder study was much smaller than the NOW study (6 hospitals admitting a total of 160,000 patients between them in a year, compared to 144 hospitals admitting over 80,000 emergency patients between them in a week) but collected much more detailed individual level information.

Universal MRSA admission screening involves a lot of work for a very low yield of new cases of MRSA carriage. In addition, many of these cases will not be optimally managed as the ability to isolate patients in the English NHS continues to be limited [20] even though levels of MRSA are falling. Checklist activated screening would seem to be an attractive option, reducing the number of laboratory screens required by 50% whilst still identifying 80% of MRSA positives. However, given the relatively low compliance we report with a national mandatory universal admission screening programme (presumably the easiest screening policy to routinise and implement), this should give pause for thought in respect of how well any policy of checklist activated screening would be implemented. NOW study infection control nurses used the standard prevalence surveillance techniques of interrogation of hospital databases, review of notes and examination or interview of patients but were unable to find risk data on a significant number of patients, which may be more reflective of actual clinical practice. This indicates that accurate implementation of a checklist by admitting nurses may not be a feasible option and that audit of compliance with such a strategy may prove difficult.

Screening all admissions to “high-risk” specialties, the national strategy which preceded universal admission screening [2], gives a low yield of MRSA colonised patients, but given the likely difficulties in implementing checklist activated screening, this strategy may prove a more attractive and feasible option as it would reduce the amount of screening to 10% of present levels but may identify the patients with the highest potential excess morbidity, mortality and probable costs due to MRSA infection. Indeed a recent model simulating transmission in three hospitals in one region, based on Dutch parameters, but considered from the USA perspective, found that “high-risk” screening was more cost effective than universal [22]. Cost effectiveness varied with admission prevalence, as in a Swiss modelling study [23]. It remains unclear what the most cost-effective screening strategy for hospitals in England should be, until data from the NOW audit is used to populate an extended version of an individual-based transmission dynamic health economic model (30) to evaluate the effectiveness and cost-effectiveness of different screening strategies in different types of trust and MRSA prevalence levels. This health economic evaluation is the subject of a subsequent paper. Until the Department of Health has reviewed all the evidence and policy implications, English hospitals are recommended to continue to implement the current policy & maximise use of isolation rooms.

## Supporting Information

File S1 **The NOW questionnaire.**
(PDF)

File S2 **Screening, isolation & decolonisation policies/practices, and laboratory methods.**
(DOC)
