# Supplementary material for: The National One Week Prevalence Audit of Universal Meticillin-Resistant Staphylococcus aureus (MRSA) Admission Screening 2012
Source: PLoS One. 2013 Sep 12;8(9):e74219. doi: 10.1371/journal.pone.0074219 (PMC3772122; doi:10.1371/journal.pone.0074219)

File S1. The NOW Questionnaire

The National One Week Prevalence Audit of MRSA Screening

Final report prepared for the Department of Health by the NOW study team.

Delivered January 2013

Authors:
Chris Fuller (Project Manager), Julie Robotham (Health Economics Modellling, Joanne Savage (Research Associate), Sarah Deeny (Health Economist), Susan Hopkins (Epidemiologist & Infection Control Doctor), Barry Cookson & Sheldon Stone (Principal Investigators).

ACKNOWLEDGEMENTS

Thanks to Infection Control Teams from the following trusts for help with piloting of the questionnaire and for providing data as a part of the Sentinel study:

1. Barnet and Chase Farm Hospitals NHS Trust
2. Harrogate and District NHS Foundation Trust
3. Cambridge University Hospitals NHS Foundation Trust
4. Royal Free Hampstead NHS Trust
5. Mid Essex Hospital Services NHS Trust
6. Colchester Hospital University NHS Foundation Trust
7. East and North Herts NHS Trust
8. University College Hospitals NHS Foundation Trust
9. York Teaching Hospital NHS Foundation Trust
10. Brighton and Sussex University Hospitals NHS Trust
11. North Cumbria University Hospitals NHS Trust
12. North Tees and Hartlepool NHS Foundation Trust
13. Portsmouth Hospitals NHS Trust
14. Whipps Cross University Hospital NHS Trust

Thanks also to Infection Control Teams in the 144 English NHS acute trusts that returned questionnaires.

This is an independent report commissioned and funded by the Policy Research Programme in the Department of Health. The views expressed are not necessarily those of the Department.

STEERING GROUP MEMBERS

Chair: Prof Brian Duerden

Bill Maton-Howarth (DH)

Carole Fry (DH)

Mike Murnane (DH)

Tommy Denning (DH)

Sally Wellsteed (DH)

Beryl Oppenheim (BIA)

Kath Banfield (IPS)

Adam Fraise (HIS)

Helen Crombie (NHS Northwest)

Graham Tanner (NCHI)

Jacqui Reilly (HPS)

Barry Cookson (NOW study)

Chris Fuller (NOW study)

Sheldon Stone (NOW study)

TABLE OF CONTENTS

	Glossary	5
	Exec summary	6
	Core report	12
	Full Report	31
	References	143
Appendix 1	DH impact assessment document	150
Appendix 2.	The NOW Questionnaire	164
Appendix 3	Methodology: modelling	188
Appendix 4	Model parameterisation	198
Appendix 4a	Parameters derived from the NOW audit	211
Appendix 5a	Sentinel Questionnaire	216
Appendix 5b	Sentinel study results and methods	223
Appendix 6	Expected number of screens per week	225
Appendix 7	Results cost –effectiveness analysis	227
	– Acute trusts	229
	– Teaching Trusts	274
	– Specialist trusts	300

GLOSSARY

CAS – Checklist activated screening

Check-list activated screening – MRSA admission screening for those patients that have one or more of the following risk factors for MRSA carriage (admission from a residential-, or nursing-home or another hospital, inpatient admission within the previous year, presence of an indwelling device or skin-break, ever previously known to be MRSA positive).

Checklist negative – Patients that have no risk-factors for MRSA carriage on admission. See "checklist activated screening".

Checklist positive – Patients that have one or more risk-factors for MRSA carriage on admission. See "checklist activated screening".

Cost effectiveness acceptability curve (CEAC)- the proportion of model simulations in which individual interventions are cost-effective at a given willingness to pay.

Cost effectiveness acceptability frontier (CEAF) - The CEAF shows only the strategy with the highest *expected* net monetary benefit at a given willingness to pay, and describes the probability of cost-effectiveness at this point.

High-risk specialties – Specialties in which patients are at particular risk of deep seated and difficult to treat infections (Haematology/Oncology, Nephrology, Trauma & Orthopaedics, Neurosurgery, Cardiothoracic Surgery, Vascular Surgery).

HR – High risk specialties

Incremental cost-effectiveness ratio (ICER). The change in cost when moving from one intervention to another, divided by the change in effect.

Low-risk specialties – All other specialties not included under high-risk specialties.

LR – Low risk specialties.

Newly MRSA positive – Patients for whom there is no previous record that they are MRSA positive

Previously MRSA positive –Patients that are flagged as being MRSA positive at any time in the past.

Risk-based screening – See "checklist activated screening".

EXECUTIVE SUMMARY

BACKGROUND: Following government and public concern at high levels of meticillin resistant *Staphylococcus aureus* between 2001-4, many national infection control interventions were introduced. These included: the clean**your**hands campaign (December 2004), the Saving Lives campaign (July 2005), a national MRSA target (November 2005), Department of Health improvement team visits to reinforce Saving Lives (from April 2006) and publication of the Health Act in October 2006, which legislated statutory criteria for infection control. Annual MRSA bacteraemia rates fell by 56% between April 2004 and March 2009).

Until April 2009 national guidelines for MRSA admission screening recommended screening patients in "High Risk" specialties (Nephrology, Neurosurgery, Orthopaedics and Trauma, Haematology and Oncology, Vascular Surgery and Cardiothoracic Surgery) where infections were likely to be deep-seated and difficult to treat. It is unclear from modelling or clinical studies, whether this is the most cost-effective strategy. A modelled Department of Health (appendix 1) impact assessment suggested that the most cost-effective strategy would be mandatory screening of appropriate elective & emergency admissions. This policy was introduced in April 2009 for electives and in December 2010 for emergencies. As models were populated by limited literature-based data, possibly unrepresentative of NHS hospitals overall, the impact assessment committed the DH to reviewing the implementation of this policy, its impact on patients and their management, its effectiveness and cost-effectiveness.

AIMS: To report on:

(1) implementation of mandatory screening in acute, teaching & specialist trusts

(2) admission prevalence of new & previously known MRSA

(3) screening, isolation, decolonisation & laboratory practices

(4) the time to result & management of patients pre-and post-result

(5) the extra yield of MRSA from routine screening compared to checklist activated or high-risk specialty screening

(6) cost effectiveness in each trust type at 3 prevalence levels of the following strategies:

- no screening,
- screening all admissions
- screening all admissions to high-risk specialty wards
- screening only those with a risk factor for MRSA carriage (checklist activated screening)
- screening high-risk specialty & checklist activated screening of low-risk specialty admissions
- screening all admissions with pre-emptive isolation of previously known MRSA positives.

METHODS:

Study design: a national one-week prevalence audit of MRSA screening with transmission dynamic and health economic modelling.

National prevalence audit: A questionnaire sent to infection control teams in all 167 English NHS acute, teaching & specialist trusts for completion between 9-15th May 2011 requesting data on (1) number of emergency, elective & day-case patients admitted & screened in one week, and the numbers of those previously known or newly identified as MRSA positive, (2) local screening, isolation, decolonisation & laboratory practices, (3) point prevalence of MRSA, (4) clinical details, pre-/post-result management & presence of known risk factors for MRSA (on an itemised checklist) for all new admission screen positive patients that week and for a random sample of 5-10 patients screening negative.

Sentinel site audit: Detailed retrospective data from 7 trusts for 2010-11 on 30-day readmission rates of MRSA positive & negative patients and discharge/inter-ward transfers.

Health economic and dynamic transmission modelling: An existing individual based stochastic dynamic transmission health economic model created for the DH funded "MECAMIP" project (Robotham et al 2011) was further developed and extended to evaluate different screening strategies at the whole hospital level. The extended model incorporated proportions of high-& low-risk specialty patients, specialty specific patient movement patterns within the hospital plus specialty specific readmission, infection & death rates. Each identified MRSA positive patient was isolated & decolonised until trust isolation capacity exceeded, when decolonisation & contact precautions were used. Three models were developed to evaluate the effectiveness & cost effectiveness of 6 screening strategies in acute, teaching & specialty trusts, populated by audit data & by economic data (NHS reference costs for bed-days (DH, 2008), Scottish Pathfinder for infection control costs (Health Protection Scotland 2011b) & MECAMIP for treatment costs (Robotham et al 2011)). Probabilistic sensitivity analyses, incorporating uncertainty in model parameters, were conducted. Incremental cost-effectiveness ratios [ICERs] (i.e. cost per Quality Adjusted Life Year [QALY] gained for one screening strategy, compared to another) were calculated. Strategies costing less than £30,000 (€35,500) per QALY gained were defined as cost effective (£30,000 being the upper limit of the usual National Health Service "willingness to pay" for health benefits).

RESULTS: ***Response rate:*** 144/167 (86.2%) trusts responded (23/26 [89%] teaching, 19/20 [95%} specialist and 98/121 [81%] acute). Risk factor & patient management information was received for 760 newly MRSA-positive & 951 MRSA negative patients.

*I**mplementation**:* Sixty-one per cent of emergency admissions (IQR 47.5%-85.8%), 81% elective and 47% (23-79%) of day-cases were screened overall.

Admission prevalence: Overall MRSA admission prevalence was 1.5%, (emergency admissions 2.1%, elective admissions 0.9% and day case admissions 0.7%) . Approximately half of cases were newly identified MRSA positives. Numbers needed to screen in order to identify one new MRSA positive were high: 102 emergency admissions, 180 elective admissions or 186 day case admissions. Numbers were highest for acute trust day cases (462) and lowest for electives in specialist trusts (80).

Local policies: nearly all trusts (92%) used Chromogenic agar for detection. Routine use of PCR was rare. Most trusts (77%) pre-emptively isolated previously known MRSA; nearly half (44%) did so for high-risk specialty patients.

***Patient management**:* Mean time to result was 2.9 days for MRSA positives, 1.75 days for negatives. A third of MRSA positive & negative patients alike were discharged before results available. Only half (55%) of new MRSA positives who were still in hospital when results were available were isolated.

Point prevalence: Overall point prevalence on 13th May was 3.3% (3.6% acute, 3% teaching & 2% specialist). A third of MRSA positive patients were not in isolation on the audit day. This number was lower in specialist trusts.

***Extra yield of routine admission**:* For all trust types, checklist activated screening would detect 80% of MRSA positives detected by routine screening (an average of 2 less/week/trust) whilst halving the number of screens. Results were similar for checklist activated screening of low-risk specialty admissions and screening all admissions to high-risk specialties. Screening just high-risk specialty admissions would detect only 10% of MRSA positive patients (24% in specialty trusts) whilst reducing screening by 90%.

Cost effectiveness of 6 screening strategies (see table 1): Screening admissions to "high-risk" specialties was the only cost-effective option at current admission prevalence in acute trusts

(£9,964/QALY), and was also cost-effective in specialist trusts (£10,777/QALY). This reflects the fact that most infections (which carry the largest determinants of cost and health benefits) occur in this patient group. In teaching trusts, high-risk specialty screening was only slightly more costly than the £30,000 willingness to pay threshold, with an ICER of £31,077/QALY. The differences in cost-effectiveness between acute and teaching trusts reflected the overall greater costs per admission in teaching trusts (causing any strategy to have a higher cost/QALY). In specialist trusts, screening all admissions with pre-emptive isolation of those known to be previously MRSA positive was cost-effective (£14,324/QALY).

In acute and specialist trusts high-risk specialty screening remained cost-effective at both high and low MRSA admission prevalence levels and became a cost-effective strategy in teaching trusts at a high prevalence (£20,715/QALY). These results therefore indicate that the cost-effectiveness of high risk specialty screening would be robust if MRSA prevalence were to rise again, to twice the current prevalence level. At lower MRSA prevalence, screening of high-risk specialties combined with checklist-activated screening of all other admissions became cost-effective in acute trusts (£23,196/QALY) as did screening all admissions in specialist trusts (£14,224/QALY). No screening strategy was deemed optimal (cost-effective) at lower prevalence in teaching trusts. At current prevalence levels none of the decisions changed for any Trust type if the willingness to pay for a QALY gained was reduced to £20,000

However, for all scenarios and settings there was substantial uncertainty (due to inherent uncertainty in the effectiveness of isolation and decolonisation), with the probability of any one strategy being the most cost effective not exceeding 30% (except at higher prevalence).

Table 1 : Cost-effectiveness of screening strategies by trust type and MRSA admission prevalence. Cost per QALY gained is shown for strategies considered both cost-effective† and non-cost-effective. Any remaining strategies for each prevalence scenario were dominated††.

Trust type	MRSA prevalence on admission	Cost-effective strategies	Non cost-effective strategies
ACUTE	High (2.80%)	Strategy 3(£8,650/QALY)	Strategy 5 (£45,257/QALY)
	Baseline (1.40%)	Strategy 3 (£9,964/QALY)	Strategy 5 (£33,806/QALY)<br>Strategy 6 (£35,796/QALY)
	Low (0.70%)	Strategy 3 (£9,796/QALY)<br>Strategy 5 (£23,196/QALY)	Strategy 6 (£87,517/QALY)
TEACHING	High (2.60%)	Strategy 3 (£20,715/QALY)	Strategy 5 (£78,258/QALY)<br>Strategy 6 (£308,839/QALY)
	Baseline (1.30%)	None of the alternative strategies were considered cost-effective.	Strategy 3 (£31,077/QALY)<br>Strategy 5 (£55,742/QALY)<br>Strategy 6 (£374,070/QALY)
	Low (0.65%)	None of the alternative strategies were considered cost-effective.	Strategy 3 (£61,710/QALY)<br>Strategy 5 (£98,634/QALY)<br>Strategy 6 (£102,797/QALY)
SPECIALIST	High (2.08%)	Strategy 3 (£9,745/QALY)	Strategy 6 (£309,264/QALY)
	Baseline (1.04%)	Strategy 3 (£10,777/QALY)<br>Strategy 6 (£14,324/QALY)	-
	Low (0.52%)	Strategy 3 (£10,566/QALY)<br>Strategy 2 (£14,224/QALY)	-

†An ICER of less than £30,000 per QALY is considered cost-effective. An ICER of more than £30,000 is not considered cost-effective. £30,000 is the upper limit of the usual NHS willingness to pay range.

††Dominated strategies are those that are more costly **and** provide less benefit than one other strategy or a combination of two other strategies. Since it can never be cost-effective to pay more for less benefit, ICERs were not calculated for these strategies.

Effect of switching to screening admissions high-risk specialties only*:* At current admission prevalence such a change would reduce total average annual costs by £1,592,000 per acute trust, £1,864,000 per teaching and £438,000 per specialist trust, at the expense of one extra colonisation/week and less than two extra infections/year in acute and teaching trusts. Specialist trusts would have even fewer colonisations but slightly more infections (2.6 more/ trust/year).

Overall the results indicate that persisting with the current policy of routine screening of all admissions, does not appear to be cost effective. Reverting to the previous targeted screening strategy of screening only admissions to high-risk specialties may generate substantial savings (on average £250m per year) across the NHS for a very minimal rise in infections (approximately two per year per trust) and colonisations (approximately one per week per trust). The cost-effectiveness of this strategy was maintained even if prevalence increased to twice the current levels.

CORE REPORT

INTRODUCTION:

Following government and public concern at reported high levels of meticillin resistant *Staphylococcus aureus* over 2001-2004 [HPA, 2004] many national infection control interventions were introduced. These included the clean**your**hands campaign (from December 2004[NPSA 2004]), the Saving Lives campaign [DH,2005] (from July 2005, a programme of implementing infection control care bundles within a clinical governance framework) , setting a national MRSA target (from November 2005), publication of the Health Act in October 2006 [DH,2006] (legislation of statutory criteria for infection control) and visits from April 2006 onwards to trusts by Department of Health improvement teams to reinforce delivery of the Saving Lives campaign. Mandatory reporting showed annual MRSA bacteraemia rates had fallen from 17.6 (April 2004-March 2005) to 7.8 cases per 100, 000 bed days (April 2008-March 2009), with evidence that the reduction was associated with specific national interventions, within the context of a high profile political drive (Stone et al 2012).

Until April 2009 national guidelines for MRSA screening recommended targeted screening of patients in “High-Risk” specialties (Nephrology, Neurosurgery, Orthopaedics and Trauma, Haematology and Oncology, Vascular Surgery and Cardiothoracic Surgery) where infections were likely to be deep-seated and difficult to treat. There was no clear clinical evidence, however, from the limited studies that had been performed, that this was more effective than routine screening of all admissions or using a check-list of clinical risk factors for MRSA carriage to assess all admissions and screening those with at least one risk factor (check-list activated screening). Modelling studies, including a Scottish Health Technology Assessment (Ritchie et al 2007), suggested that, although there was little to choose between long term prevalence levels achieved by different screening strategies, the actual costs varied substantially. A Department of Health (England) impact assessment (appendix 1) using modelling suggested that the most cost-effective strategy would be mandatory screening of all appropriate elective admissions (except for paediatric, maternity and same day case admissions) and all appropriate emergency admissions. This policy was introduced in April 2009 for elective admissions and in December 2010 for emergencies. However, as these modelling studies were populated by limited data from the literature, which were unrepresentative of NHS hospitals overall, the impact assessment committed the Department to reviewing the implementation, impact on patients and their management, and the effectiveness and cost-effectiveness of this policy.

The current study was, therefore, commissioned to perform a national prevalence audit of MRSA screening providing representative clinical and economic data which would be used to populate an individual-based transmission dynamic model evaluating different screening strategies. It was also able

to incorporate findings from the Scottish Pathfinder study (Health Protection Scotland 2011 a, b, c, d), which published a prevalence model in 2011, evaluating different screening strategies, using data from three trusts that had been screening all admissions for a year. They explicitly recommended that future models should be individual-based to develop more complex, powerful tools for predictive modelling to inform decisions on costed management of healthcare associated infection. This we have done.

STUDY AIMS: There were six objectives:-

Objective 1. To report how widely the policy of screening Emergency and Elective admissions was implemented across the NHS in the three different trust types (acute, teaching , specialist)

Objective 2: To report the prevalence of MRSA carriage on admission in Emergency and Elective admissions in different types of trust and the proportion of carriage that was previously unknown.

Objective 3: To report on screening, isolation and decolonisation policies and practices, laboratory methods and costs

Objective 4: To report on how patients were managed, how soon results were available, how many patients were isolated and / or decolonised pre-emptively and after the result was known, and how many were treated for infection.

Objective 5: To determine the extra yield of MRSA positive patients achieved by routine admission screening for the NHS as a whole and for each of the three types of trust (acute, teaching and specialist) compared to:

a) checklist activated screening of all patients (ie screening only those with one or more risk factors for MRSA carriage,
b) screening all admissions to “high-risk” specialties only
c) screening all admissions to high-risk specialties plus checklist activated screening of all other admissions.

Objective 6: To use the audit data and reliable cost data to populate an existing model of hospital MRSA transmission to provide predictions of the effectiveness and cost-effectiveness for each type of trust for six different screening strategies

1. no screening
2. screening all admissions (Emergency and Elective)

3. screening admissions to “high-risk” specialties only
4. checklist activated screening of all admissions
5. strategy 3 plus checklist activated screening all other admissions
6. strategy 2 (screening all admissions) plus pre-emptive isolation of those known to be previously MRSA positive.

METHODS

Study design: a national prevalence audit of MRSA screening with transmission dynamic and health economic modelling.

National prevalence audit: A national one-week prevalence audit of MRSA screening was carried out through a questionnaire (Appendix 2) sent to infection control teams in all 167 English NHS acute hospital trusts for completion between 9 and15th May 2011 requesting data on (1) the number of Emergency, Elective, and day-case patients admitted and screened in one week, with numbers of new and previously known MRSA positive screens (2) local screening, isolation, decolonisation practices, laboratory methods (3) point prevalence of colonised and infected MRSA patients on a given day (4) clinical data on all MRSA patients newly identified by admission screen that week, and on a random sample of 5-10 patients screening negative, including time to result, pre- and post-result management. The presence of any risk factors for MRSA was evaluated for MRSA positives using a five-item checklist, and for MRSA negatives using the same checklist with a sixth risk factor of ever having been MRSA positive.

Sentinel site audit: Data were collected in the autumn/winter of 2011 from seven trusts who volunteered to provide retrospective data for the financial year 2010-2011. Data were collected on proportions of MRSA positive and negative patients readmitted within 30 days, number of ward stays per patient admission, discharge and transfer destinations of intensive care units (ICU) patients and numbers of ICU wards, ICU beds and ICU isolation rooms (Appendix 4).

Health economic and dynamic transmission modelling: An existing individual based stochastic dynamic transmission model created for the Department of Health funded “MECAMIP” project (Robotham 2011), was developed further to evaluate the effects of screening, isolation and decolonisation policies on MRSA transmission at the whole hospital level. Model extensions included incorporation of high-risk and low-risk specialty classification, realistic patient movement between specialties and between the hospital and community populations, Elective and Emergency admission

routes, as well as specialty specific infection, death and discharge rates (Appendices 5 & 6). Individual patients were classified as either MRSA negative (or susceptible), colonised with MRSA or having an MRSA infection, and could move between these categories on a daily basis. Patients' (actual and perceived) MRSA status could be tracked over time, each MRSA positive patient being isolated and decolonised until the trust's isolation capacity was exceeded, after which decolonisation and contact precautions were used. Three distinct models were developed for the three trust types, accounting for size, distribution of high and low-risk specialties, numbers of isolation beds, admission prevalence, proportions of patients checklist positive, previously known to be MRSA positive and admitted as Electives or Emergencies. The effect of different screening strategies was modelled in each trust type for three MRSA prevalence levels. Sensitivity analyses for transmission and death rate were also performed.

The models were populated by audit questionnaire data. Economic data for costs of isolation, screening, swabbing, decolonisation, and contact precautions were taken from the Scottish Pathfinder study (Health Protection Scotland 2011b), using NHS reference costs for bed days (DH 2008). Treatment costs, estimates for transmission, death and intervention effectiveness came from robust analyses carried out for the DH-funded MECAMIP project model (Robotham et al 2011). Probabilistic sensitivity analyses, incorporating uncertainty in intervention effectiveness parameters, were conducted. Incremental cost-effectiveness ratios [ICERs] (i.e. cost per Quality Adjusted Life Year [QALY] gained for one screening strategy, compared to another) were calculated. Strategies costing less than £30,000 (€35,500) per QALY gained were defined as cost effective (£30,000 being the upper limit of the usual National Health Service "willingness to pay" for health benefits).

RESULTS

Response rate: 144/167 (86.2%) trusts responded (23/26 [89%] teaching, 19/20 [95%} specialist and 98/121 [81%] acute) although not all provided full sets of data. Risk factor information was received for 760 new MRSA positive and 951 MRSA negative patients.

Objective 1: To report how widely the policy of screening Emergency and Elective admissions was implemented across the NHS in the three different trust types (acute teaching and specialist)

Screening was performed on 61% IQR (47.5%-85.8%) of emergency-, 81% of elective-, and 47% (IQR 23-79%) of day-case admissions. Significant differences between trust types were that 88% of

electives were screened in acute trusts and 74% of day cases were screened in specialist trusts. High-risk specialties accounted for 7.3% of emergency admissions and 14.75% of elective admissions.

Objective 2*: To report the prevalence of MRSA carriage on admission in Emergency and Elective admissions in different types of trust and the proportion of carriage that was previously unknown.*

The overall, prevalence of MRSA on admission screening was 1.5%, (emergency 2.1%, elective 0.9% , and day case 0.7%). Prevalence of newly identified MRSA was approximately half of these numbers: 0.8% (overall), 1% (emergency); 0.6 % (elective) and 0.5% (day case). The number needed to screen to detect one new case of MRSA was 102 (emergency), 180 (elective) and 186 (day case) overall. The number needed to screen was higher (462) for day cases in acute trusts and for emergencies in specialist trusts (163) but lower (80) for electives in specialist trusts.

Objective 3: To report on screening, isolation and decolonisation policies and practices, laboratory methods and costs

Screening policies: 10% of trusts (all specialist) screened all patients. Over 70% followed DH advice for exemptions from screening.

Isolation policies: Pre-emptive isolation was used by 77% of trusts for patients with a past history of MRSA, and by 44% for patients in high-risk specialties, by 8% for other patient groups and by 1.5% for all patients.

Decolonisation: Pre-emptive decolonisation was used by 35% of trusts for patients with a past history of MRSA, by 41% for high-risk specialty patients, by 10% for all patients and was not used at all in 34% of trusts. Ninety-two per cent used nasal mupirocin with a chlorhexidine (50%) or octenidine (36%) wash as the main decolonisation regime.

Sites swabbed: All trusts performed nasal swabbing with 92% also swabbing wounds/devices plus or minus groin or perineum.

Laboratory methods: Chromogenic agar was used for detection of MRSA in 82% of trusts for Emergencies (with broth enrichment in a further 10%) and by 87% for Electives. PCR was used routinely in very few trusts (5% for all Emergencies, by 1% for all Electives, by 16% for selected Emergencies, and by 11% for selected Electives).

Costs: laboratories charged a mean (sd) of £5.68 (£4.44) for chromogenic agar, and £25.86 (£9.64) for PCR. Most trusts (85%) processed the swabs themselves rather than sending to another trust with less than 2% sending to a private laboratory.

Objective 4: ***To report on how patients were managed, how soon were results available, and how many patients were isolated and / or decolonised pre-emptively and after the result was known, and how many were treated for infection.***

Turn round time for processing and reporting swab results: This was a mean of 2.87 days for MRSA positive results and 1.75 days for negative results. A third of patients (both MRSA positive and negative) were discharged before the result became available.

Pre-emptive isolation and decolonisation/suppression: Sixteen per cent of MRSA positive admissions were pre-emptively isolated and 6% pre-emptively decolonised, compared to 6% and 10% of negative admissions.

Post result isolation and decolonisation/suppression: Fifty-five per cent of new MRSA positives who were still in hospital were isolated. Decolonisation was started in 73% of all MRSA positives.

Point prevalence: On the audit day of 11th May, 3.3% of in-patients had MRSA (2% in specialist Trusts, 3% in teaching and 3.6% in Acute) and 11% of these were receiving systemic antibiotics for any MRSA infection. A third of these were not isolated. In specialist trusts nearly all were isolated.

Objective 5: ***To determine the extra yield of MRSA positive patients achieved by routine admission screening for the NHS as a whole and for each of the three types of trust (acute, teaching and specialist) compared to (a) check list activated screening of all patients (b) screening "high-risk" specialties only (c) and screening all admissions to high-risk specialties plus checklist activated screening of all admissions to low-risk specialties***.

For the NHS as a whole, checklist activated screening would reduce the numbers of screens from 790 to 398 a week per trust, whilst identifying 9 out of an average of 11 MRSA positive admissions (new and previously known). In other words, halving the number of screens, whilst detecting just over 80% of MRSA. This did not vary significantly between trust types.

Screening of all admissions to high-risk specialties plus checklist activated screening of all other admissions, would yield similar results. Just over half (55%) of the admitted population would be screened (448 screens/week/trust). This varied little between the three trust types, except that the detection rate was 90% in acute trusts.

In contrast, if only high risk specialties were screened, there would be a 90% reduction in screening (to 87/week/trust), but only 9% of MRSA positives would be detected. Although acute trusts followed this pattern, detection rates were higher in teaching (16%) and specialist trusts (24%), as the proportions of high-risk specialty patients were higher. Reductions in screening were less marked (80% reduction in teaching and 70% reduction in specialist).

Objective 6: ***To use audit data and reliable cost data to populate an existing model of hospital MRSA transmission to provide predictions of the effectiveness and cost-effectiveness for each type of trust of the above six different screening strategies.***

ACUTE TRUSTS (Appendix 7a):

At current MRSA admission prevalence (1.4%), only strategy 3 (screening admissions to "high-risk" specialties) was cost effective, at £9,964/QALY. Strategy 5 (screening admissions to high risk specialties plus checklist activated screening for low risk specialties) was slightly too costly and above the £30,000 willingness to pay threshold (£33,806/QALY) (table 1).

Results were very similar using the Scottish Pathfinder checklist to determine checklist activated screening (strategy 3 costing £9,731/QALY and strategy 5 (£33,206/QALY) (table 2). There was substantial uncertainty, however, with the probability that any one strategy was the most cost-effective within a willingness to pay range of £20,000-30,000/QALY clustered together, and not exceeding 30%.

In higher prevalence settings (2.8%), strategy 3 became better value for money at £8,650/QALY (table 1). Although the probability that it was the most cost-effective option was approximately 50% at lower

willingness to pay values (around £10,000/QALY), there was substantial uncertainty within the usual NHS range of £20,000-£30,000/QALY.

At low prevalence settings (0.7%), strategy 3 remained cost effective at £9,796/QALY (table 1). However, a move to strategy 5 from 3 also came below the threshold at £23,196/QALY. There was substantial uncertainty as above.

In a setting of reduced transmission in high risk specialties, strategy 3 was the most cost-effective option and, despite having a greater cost per QALY (at £26,551/QALY), it remained optimal in a setting of reduced death rates (table 2).

For acute trusts, compared to universal screening, restricting screening to high-risk specialty patients would reduce total annual costs by an average of £1,592,000 per acute trust, (£619 per admission), at the expense of 56.5 more transmission events (i.e. one extra colonisation per week per trust) and 1.7 more infections per year. At higher prevalence levels this equated to savings of £1,766,148 (£670 per admission), 63.5 more colonisations (1.2 extra per trust/week) and 2.9 more infections per year.

TEACHING TRUSTS (Appendix 7b):

At current MRSA admission prevalence (1.3%), strategy 3 (screening admissions to "high-risk" specialties only) was not cost-effective, but was only just above the £30,000 willingness to pay threshold at £31,077/QALY (table 1). However, at higher prevalence levels (2.6%), this strategy became cost-effective at £20,715/QALY (table 1), with a probability of cost-effectiveness exceeding 40%. At low prevalence levels (0.65%), no screening strategy was considered cost-effective, with strategy 1 (no screening) and isolation only of clinical cases being optimal (table 1).

For teaching trusts, restricting screening to high-risk specialty patients would reduce total annual costs per trust by £1,864,000 (£576 per admission), at the price of 47 more colonisations (less than one extra/week/ trust) and 1.2 more infections per year.

SPECIALIST TRUSTS (Appendix 7c):

At a current admission prevalence of 1.04%, strategies 3 and 6 (screening all admissions) plus pre-emptive isolation of those known to be previously MRSA positive) were cost-effective at £10,077/QALY and £14,324/QALY respectively (table 1). There was substantial uncertainty, with the probabilities that any one strategy was the most cost-effective clustering at less than 30%. **At higher prevalence levels**

(2.1%), strategy 3 remained cost-effective at £9,745/QALY (table 1) and was the optimal option with a 40% probability of cost-effectiveness. **In low prevalence settings (0.5%),** both strategies 3 and 2 (screening all patients) were cost-effective at £10,566/QALY and £14,224/QALY respectively (table 1), although there is substantial uncertainty with almost all screening strategies having an approximately 25% probability of being cost-effective.

For specialist trusts, restricting screening to high-risk specialty patients would reduce total annual costs per trust by £438,000 (£600 per admission), at the price of 18.3 more colonisations (one extra/every three weeks/ trust) and 2.6 more infections per year.

Table 1 : Cost-effectiveness of screening strategies by trust type and MRSA admission prevalence. Cost per QALY gained is shown for strategies considered both cost-effective† and non-cost-effective. Any remaining strategies for each prevalence scenario were dominated††.

Trust type	MRSA prevalence on admission	Cost-effective strategies	Non cost-effective strategies
ACUTE	High (2.80%)	Strategy 3(£8,650/QALY)	Strategy 5 (£45,257/QALY)
	Baseline (1.40%)	Strategy 3 (£9,964/QALY)	Strategy 5 (£33,806/QALY)<br>Strategy 6 (£35,796/QALY)
	Low (0.70%)	Strategy 3 (£9,796/QALY)<br>Strategy 5 (£23,196/QALY)	Strategy 6 (£87,517/QALY)
TEACHING	High (2.60%)	Strategy 3 (£20,715/QALY)	Strategy 5 (£78,258/QALY)<br>Strategy 6 (£308,839/QALY)
	Baseline (1.30%)	None of the alternative strategies were considered cost-effective.	Strategy 3 (£31,077/QALY)<br>Strategy 5 (£55,742/QALY)<br>Strategy 6 (£374,070/QALY)
	Low (0.65%)	None of the alternative strategies were considered cost-effective.	Strategy 3 (£61,710/QALY)<br>Strategy 5 (£98,634/QALY)<br>Strategy 6 (£102,797/QALY)
SPECIALIST	High (2.08%)	Strategy 3 (£9,745/QALY)	Strategy 6 (£309,264/QALY)
	Baseline (1.04%)	Strategy 3 (£10,777/QALY)<br>Strategy 6 (£14,324/QALY)	-
	Low (0.52%)	Strategy 3 (£10,566/QALY)<br>Strategy 2 (£14,224/QALY)	-

†An ICER of less than £30,000 per QALY is considered cost-effective. An ICER of more than £30,000 is not considered cost-effective. £30,000 is the upper limit of the usual NHS willingness to pay range.
††Dominated strategies are those that are more costly **and** provide less benefit than one other strategy or a combination of two other strategies. Since it can never be cost-effective to pay more for less benefit, ICERs were not calculated for these strategies.

Table 2. Cost-effectiveness of screening strategies in alternative scenarios (simulations performed for an Acute Trust setting). Cost per QALY gained is shown for strategies considered both cost-effective† and non-cost-effective. Any remaining strategies for each prevalence scenario were dominated††.

Scenario	Cost-effective strategies	Non-cost-effective strategies
Lower transmission in high-risk specialties*	Strategy 3(£12,382/QALY)	Strategy 6 (£85,713/QALY)
Reduced probability of death in high-risk specialties**	Strategy 3 (£26,511/QALY)	Strategy 6 (£296,859/QALY)
Use of the Scottish Pathfinder checklist to identify those at risk of MRSA carriage***	Strategy 3 (£9,731/QALY)	Strategy 5 (£33,206/QALY)<br>Strategy 6 (£87,517/QALY)

†An ICER of less than £30,000 per QALY is considered cost-effective, An ICER of more than £30,000 is not considered cost-effective. £30,000 is the upper limit of the usual NHS willingness to pay range.

††Dominated strategies are those that are more costly **and** provide less benefit than one other strategy or a combination of two other strategies. Since it can never be cost-effective to pay more for less benefit, ICERs were not calculated for these strategies.

* MRSA transmission rates in high-risk specialties were reduced (to be midway between values for high-risk and low-risk specialties in the baseline model (see appendix 4, table 6).

** The probability of death in high-risk specialties was reduced to the level of that in low-risk specialities (see appendix 4, table 4).

***Assumes that 26% of all admissions have a risk factor compared to the assumption used in the baseline model that 56% of all admissions have a risk factor (see appendix 4, table 1).

DISCUSSION

Summary of findings:

There was an excellent response to the study with 86% of all trusts providing data needed to inform the modelling. It also yielded seven important findings.

Firstly, implementation of Emergency screening was poor at 61% (IQR 47.5%-85.8%) and even worse for eligible day case admissions (47%) (IQR 23-79%) although better for electives (81%).

Secondly, admission prevalence of MRSA was low at 1.5% (overall), 2.1% (Emergency), 0.9% (Electives) and 0.7% (day cases), with approximately half being newly identified MRSA. This meant that numbers needed to screen in order to identify one new positive were high, especially for Elective (180) and day case (186) admissions, but were lower (80) for Electives in specialist trusts.

Thirdly, over three quarters of trusts pre-emptively isolated those with previous MRSA and nearly half pre-emptively isolated high-risk specialty patients.

Fourthly, a third of MRSA positive and negative patients alike were discharged before the results of screening were available, as the mean turn round time was 2.87 days for positives and 1.75 days for negatives. Decolonisation was started in three-quarters of all patients screened and found to be newly MRSA positive. Just over half of new positives who were still in patients were isolated. Very few were using PCR test methodologies that could potentially produce faster results.

Fifthly, the point prevalence survey showed that, although the overall prevalence of MRSA was 3.3% (3.6% acute, 3% teaching and 2% specialist), a third of MRSA patients were not isolated. In specialist trusts nearly all MRSA positive patients were isolated.

Sixth, for the NHS as a whole, checklist activated screening would detect 80% of the MRSA positive patients detected by routine screening (an average of 2 less/week/trust) and would halve the number of screens required. Screening high-risk specialty admissions would detect only 10% of MRSA positive

patients (although this proportion would be higher in teaching and specialist trusts) whilst reducing screening by 90%.

Finally, the cost effectiveness of different screening strategies, evaluated at a willingness to pay threshold of £30,000 per QALY gained, showed screening admissions to “high-risk” specialties performed best overall in different scenarios and settings. In the base case scenario none of the decisions changed for any Trust type if the willingness to pay for a QALY gained was reduced to £20,000. However, there was substantial uncertainty, with the probability that any one strategy was the most cost-effective generally not exceeding approximately 30%. This low probability of cost-effectiveness associated with each of the screening strategies is due to the inherent uncertainty in the effectiveness of the accompanying intervention (isolation and decolonisation). If we had assumed that identifying MRSA positive patients led to placing them under an intervention that was 100% effective and prevented any further colonisations or infections, this uncertainty would be reduced. However, our estimates of the effectiveness of isolation and decolonisation represent the best available evidence, and we considered it to be important for decision makers to visualise this uncertainty *within* the decision making process.

At current admission MRSA prevalence levels, moving from the current strategy of routine screening to targeted screening of high-risk specialty patients would reduce total average annual costs by £1,592,000 per acute trust, £1,864,000 per teaching and £438,000 per specialist trust. This would be at the expense of one extra colonisation per week per trust and less than two extra infections per year in acute and teaching trusts. For specialist trusts there would be even fewer colonisations but slightly more infections (an extra 2.6 per trust per year).

Strengths

The first strength of the study was the high response rates from trusts. This enabled audit data representative of current NHS settings to populate the model. The availability of good quality economic data from the Pathfinder (Health Protection Scotland 2011b) and MECAMIP studies (Robotham et al 2011) also contributed to the model and ensured that it was relevant to, and representative of, current NHS settings, practice and prevalence levels.

The use of a powerful sophisticated individual based model, as suggested by the Scottish Pathfinder study as the next appropriate direction for modelling studies was a particular strength. The incorporation of dynamic transmission models, with robust estimates of transmission, into economic evaluation enabled consideration of population–level effects of screening strategies, which benefited

not only the person screened but other patients. This avoided underestimating the effects of screening. Use of a stochastic model, with 1000 simulations per parameter set, for each strategy, minimises the uncertainty due to chance effects, which were dominant in the small populations seen in NHS trusts.

Other strengths included simulation of the time to result delays, and the capture of more realistic patient movements between specialities and between hospital and community. Long-term effects were considered by adjusting the quality adjusted life expectancy of infected patients with long-term detriments to health. The additional length of stay and additional risk of mortality due to infection were dominant economic parameters, and the way in which these were modelled, specifically by determining each patient's daily probability of discharge and death, adjusted according to their infection status make this model more relevant to the real world.

Limitations

There were the assumptions that both MRSA transmission and probability of death in high-risk specialties were equivalent to that in Intensive Care Units (ICUs), and that in low risk specialties they were equivalent to general medical wards. However, this was adjusted within sensitivity analyses, assuming that transmission in high-risk settings was intermediate between ICUs and general medical wards and that probabilities of death for the whole patient population were equivalent to those in general medical wards. Results were robust to these parameter changes.

The second main limitation, was that transmission was modelled at a specialty level, with homogenous mixing assumed within specialties, which might have especially affected large teaching trusts. A greater level of 'granularity' i.e. including a ward-based structure, and inclusion of ICUs in particular, would enhance the model. Further modelling, incorporating data from the sentinel sites on inter-ward transfers and readmission rates of MRSA patients would lead to more realistic transmission dynamics, and thus more reliable evaluation of screening strategies. Given that screening of admissions to high-risk specialties was only just above the willingness to pay threshold for teaching trusts, and that in low prevalence teaching hospital settings, no strategy appeared to be cost effective, it will be important to re-run the models, considering transmission at a more detailed ward level. In addition, modelling of the effects of excluding day-case or elective screening and of the effects of pre-emptive isolation of all patients with a history of MRSA (which audit data showed that most trusts attempt) would also have enhanced the applicability of the model to the current NHS. Other potential limitations were assumptions that isolation had no adverse effects and that there was no increased mupirocin resistance due to its widespread use in decolonisation.

Comparison with other modelling studies:

The most important comparisons are with the Department of Health impact assessment (appendix 1) the Scottish HTA model (Ritchie et al 2007) and the subsequent Scottish Pathfinder study (Health Protection Scotland 2011b). Non-UK or Republic of Ireland modelling (Hubben et al 2012, Murthy et al 2010, Lee et al 2009) or clinical studies (Harbath et al 2008) are not directly compared to this study due to differences in setting, their concentration on a single or limited number of specialties, rather than considering a whole hospital, or their evaluation of universal PCR screening, which is little used in the UK, with a much smaller range of alternative screening policies than those considered in the NOW project.

The DH impact assessment (appendix 1) did not account for transmission, considering only patient level events limited to those patients colonised on admission, evaluated health benefits using deaths avoided (each death having a value of £250,000) and had a much higher estimation of the effectiveness of isolation and decolonisation at 90%, whereas our model estimated reduction in transmission for primary isolation at 64% (SD 14%), and at 24% (SD 12%) for contact precautions and decolonisation. These parameters have previously been found to exert the greatest influence, and this may explain why the DH policy of screening all admissions rarely proved cost-effective in our models, which incorporated full uncertainty of these parameters in order to maximise robustness of results.

The Scottish HTA prevalence model (Ritchie et al 2007)was limited, as its authors acknowledged, by substantial uncertainty in parameter estimation, which they did not attempt to adjust for. Our model included full uncertainty distributions for intervention parameters and used national audit data whenever possible.

The subsequent Scottish Pathfinder study (Health Protection Scotland 2011 a b c d) provided an excellent review and valuable general model of MRSA screening and associated cost-effectiveness. The authors explicitly recommended that the next step for modelling should be an individual-based approach (see introduction above), as conducted here, with stochastic modelling calculating each patient's probability of colonisation or infection on a daily basis, which depended on how many such patients they were surrounded by, and what screening, isolation and decolonisation interventions these were receiving (which might also change on a daily basis). Other key differences included

extensive parameterisation of transmission which could change daily, the modelling of uncertainty, and incorporation of real patient movement data and patient level differences in probabilities of discharge and mortality.

The cost effectiveness results

The relative cost-effectiveness of screening admissions to high-risk specialties probably derives from the fact that most infections occur in this population, who have a higher probability of progressing from colonisation to infection and it is infections that have the largest impact on length of stay and mortality, the largest cost and health benefit determinants. The reduction of these infections, combined with lower screening and isolation costs associated with a strategy of screening only admissions to high risk specialties, makes this a more cost-effective strategy, especially in higher prevalence settings. The differences in cost-effectiveness between acute and teaching trusts reflected the overall greater costs per admission in teaching trusts (causing any strategy to have a higher cost/QALY) and lower transmission and MRSA admission prevalence in the high risk specialty population (meaning the screening strategies had less ability to reduce transmission and infections, and therefore less ability to generate health benefits in teaching trusts). The differences in cost-effectiveness between specialist and acute hospitals reflected higher transmission rates in specialist hospitals meaning that strategies could better prevent transmission and infections and thus generate greater QALY gains, making all strategies better value for money (even the most costly ones, including screening all admissions). The small population size of specialist trusts in particular also meant that stochastic (i.e. chance) effects had a greater impact on the transmission dynamics.

It should also be noted that there existed substantial uncertainty, with the probability of any one strategy being a better option than any other of around only 30% (at willingness to pay values of £20-30,000/QALY).

Overall the results indicate that persisting with the current policy of routine screening of all admissions, does not appear to be cost effective. Reverting to the previous targeted screening strategy of screening only admissions to high-risk specialties may generate substantial savings (on average £250m per year) across the NHS for a very minimal rise in infections (approximately two per year per trust) and colonisations (approximately one per week per trust). The cost-effectiveness of this strategy was maintained even if prevalence increased to twice the current levels.

Generalisability:

Wherever possible model inputs came from audit data to reflect current status of trusts in England, with extensive sensitivity analyses undertaken demonstrating the robustness of findings to trust type, MRSA prevalence, and assumptions regarding transmission and mortality probability parameters. This enabled the model to have substantial generalisability. However, the certainty in choosing between strategies was low and changes in costs and effects were clustered.

FULL REPORT

BACKGROUND

Following government and public concern at reported high levels of meticillin resistant *Staphylococcus aureus* over 2001-4 (HPA 2004) many national infection control interventions were introduced. These included the clean**your**hands campaign (NPSA 2004, Stone et al, 2012) (from December 2004), the Saving Lives campaign [DH2005] (a programme of implementing infection control care bundles within a clinical governance framework (from July 2005), setting a national MRSA target (from November 2005), publication of the Health Act in October 2006 [DH 2006] (legislation of statutory criteria for infection control) and visits from April 2006 onwards to trusts by Department of Health improvement teams to reinforce delivery of the Saving Lives campaign. Mandatory reporting showed annual MRSA bacteraemia rates had fallen from 17.6 (April 2004-March 2005) to 7.8 cases per 100, 000 bed days (April 2008-March 2009), with evidence that the reduction was associated with specific national interventions, within the context of a high profile political drive (Stone et al 2012).

Reducing the burden of health care associated infection remained a top priority for the Department of Health (The Health Act 2008). Reduction of meticillin resistant *Staphylococcal aureus (*MRSA) to an unavoidable minimum is a major requirement in the NHS Operating Framework 2010-2011 (DH 2009) in view of its historically high levels and associated infection, mortality and cost (Coia et al 2006; HPA 2012). The basis of MRSA reduction is screening for asymptomatic carriers, isolation of those found to be MRSA positive (MRSA+ve), and suppression/decolonisation therapy (Cooper et al 2004). Epidemiological and health economic modelling, largely based on data from the literature, suggests that increasing either the intensity of screening or the isolation capacity of a hospital is effective, provided neither is limited (Cooper et al 2003, 2004). There are no randomised controlled trials to provide guidance on the most effective and cost-effective screening strategies and clinical studies in the United Kingdom vary in the patient group screened (Rao et al 207; Hardy et al 2009, Jeyaratnam et al 2008, Creamer et al 2010, Smyth et al J 2008) and reported effectiveness of different screening strategies.

National guidance, published in 2006 (Coia et al 2006), recommended targeted screening of patients in high risk specialties (Nephrology, Neurosurgery, Orthopaedics and Trauma, Haematology and Oncology, Vascular Surgery and Cardiothoracic Surgery) where infections were likely to be deep-seated and hard to treat and/or targeted screening of individual patients with known risk factors for MRSA

carriage, and/or of patients . Hospitals had discretion to implement these guidelines according to local circumstance.

A Scottish Health Technology Assessment systematic review (Ritchie et al 2007) produced a prevalence model, populated by data from the literature, which showed that routine screening of all admissions was effective, especially when combined with pre-emptive isolation of high-risk specialty patients. Long term differences in prevalence levels differed little between screening strategies, although there were substantial variations in five-year costs. It appeared possible that teaching hospitals could save up to £2M and non-teaching hospitals more than £1M over five years by using other risk based screening strategies instead of routine admission screening. On the basis of its own impact assessment (Appendix 1) which modelled the cost effectiveness of different screening and decolonisation strategies in preventing MRSA bacteraemias, wound infections and deaths, the Department of Health decided to introduce mandatory screening of all appropriate elective admissions from April 2009 (by which time MRSA bacteraemia rates had fallen to 7.8/100,000 beddays) and of all appropriate emergency admissions from December 2010 (by which time rates had fallen further to 4.2/100,000 beddays). Certain day cases (ophthalmology, endoscopy, dental and minor dermatology), paediatrics (unless in a high risk speciality) and maternity/obstetric cases were to be excluded from routine screening because the model concluded screening these groups was not cost effective. The impact assessment committed the Department to review the effectiveness of this policy in the future, a commitment reflected in calls by both the National Audit Office (NAO 2009) and Public Accounts Committee (Public accounts committee 2009) for a robust review of the implementation of the policy, its effectiveness and cost-effectiveness, and its impact on patients and their management.

The NOW Study

This research commissioned by the Department and whose aims, methods, results and implications for research and policy are described in this report, took the form of a national prevalence audit of MRSA screening providing representative clinical and economic data to populate an individual-based transmission dynamic model evaluating the effectiveness and cost effectiveness of different screening strategies. The study was able to incorporate key findings of the Scottish Pathfinder study (Health Protection Scotland 2011b, Stewart et al 2011) which reported during the study. Pathfinder produced a prevalence model of the effectiveness and cost effectiveness of routine admission screening of all admissions, populating their model by clinical and economic data taken from three NHS Boards (equivalent to three English trusts), where routine screening had been implemented for a year. In purely economic terms the most cost effective strategies were, in order:

1. checklist activated screening of all admissions (using a check-list of clinical risk factors for MRSA carriage to assess all admissions and screening those with at least one risk factor),
2. two swab (nasal and perineal) screening of all admissions to high-risk specialities combined with universal (routine) check-list activated screening of all other admissions,
3. universal nasal swab screening.

Their overall conclusion was that, taking public acceptability and the economic climate into account, the second strategy (two swab screening of all admissions to high-risk specialities combined with universal check-list activated screening of all other admissions) “appeared to offer the best clinical return for a similar level of financial investment to universal screening of all admissions.”. They explicitly recommended that future models should move from prevalence models to individual-based ones, to facilitate development of more complex, powerful tools for predictive modelling to inform decisions on costed management of healthcare associated infection.

OBJECTIVES

The current study had six objectives:-

Objective 1. To report how widely the policy of screening Emergency and Elective admissions was implemented across the NHS in the three different trust types (acute teaching and specialist)

Objective 2: To report the prevalence of MRSA carriage on admission in Emergency and Elective admissions in different types of trust and the proportion of carriage that was previously unknown.

Objective 3: To report on screening, isolation and decolonisation policies and practices, laboratory methods and costs

Objective 4: To report on how patients were managed, how soon were results available, and how many patients were isolated and / or decolonised pre-emptively and after the result was known, and how many were treated for infection.

Objective 5: To determine the extra yield of MRSA positive patients achieved by routine admission screening for the NHS as a whole and for each of the three types of trust (acute, teaching and specialist) compared to (a) check list activated screening of all patients (b) screening “high-risk” specialties only (c) and screening all high-risk specialty patients with checklist activated screening of all low-risk patients.

Objective 6: To use these data and reliable cost data to populate an existing model of hospital MRSA transmission to provide predictions of the effectiveness and cost-effectiveness for each type of trust for six different screening strategies:

1) no screening

2) screening all admissions (emergency and elective)

3) screening admissions to "high-risk" specialties only

4) checklist activated screening of all admissions

5) strategy 3 plus checklist activated screening all other admissions

6) strategy 2 (screening all admissions) plus pre-emptive isolation of those known to be previously MRSA positive.

METHODS

Methodology audit questionnaire.

A national one-week prevalence audit of MRSA screening with modelling was deemed to be the most practical way to evaluate routine admission screening in a timely manner. Questionnaires were sent to infection control teams in all English NHS acute trusts for completion in May 2011, allowing time for acute admission screening to bed down nationally.

Ethics and Research Governance

Since the study used anonymised, confidential patient data and effected no change in clinical management, the National Research Ethics Service considered that it did not require formal ethical approval.

Study Design

A national one-week prevalence audit of MRSA screening with epidemiological and health economic modelling data was carried out through a questionnaire sent to infection control teams in all English NHS acute trusts for completion in May 2011

Questionnaire design and piloting

The questionnaire was designed by the research team in December 2010 and piloted in 10 trusts in early 2011 to check for understanding (face validity) of individual items and to assess for the feasibility of data collection. Further changes were made following meetings in March/April 2011 to discuss participation in the audit with representatives from approximately 125 hospital infection control teams in nine regional meetings around the country. The steering group was closely involved in the conduct of the research and gave input into the questionnaire design. A paper copy of the questionnaire was sent to infection control teams in all 167 NHS acute trusts at the end of April 2011.

The questionnaire (see appendix 2) was divided into 5 sections as follows:

Sections 1a: Trust level data on the number of emergency, elective, and day-case admissions for the week 11th -17th April 2011. Data was broken down into "high-risk" specialty admissions (Nephrology, Neurosurgery, Orthopaedics and Trauma, Haematology and Oncology, Vascular Surgery and

Cardiothoracic Surgery) and "low-risk" specialty admissions (all other specialties). Trusts were defined as Acute, Teaching or Specialist according to standard HPA definitions used in mandatory reporting.

Section 1 b: The number of MRSA screens performed on emergency, elective and day case admissions that week with the numbers screening positive for each of these three groups, divided into new and previously known MRSA. These data were requested retrospectively, to facilitate questionnaire completion and rapid return as data for the audit week in May would not be available from trust informatics for two to three weeks, and it is a reasonable assumption this admission data would be similar to admission data for the audit week in May.

Section 2: Trust level data were also collected on local screening practice and other policies including:

- Screening including exclusion criteria
- Sites routinely swabbed for emergency and elective screens
- If swabs were pooled or not
- Routine laboratory tests used for emergency and elective patients
- How much the laboratory charged for screening
- Where swabs were processed (in the trust, at another trust or in private laboratory)
- Categories of emergency and elective patients for whom PCR was routine
- Pre-emptive decolonisation/suppression and isolation policies for emergency and elective patients.
- First line decolonisation regimes

Section 3: Trust level Point-prevalence data for Wednesday 11th May 2011.

- the number of inpatients in the trust
- the number of high risk and low risk speciality wards
- the number of side rooms or isolation ward beds
- the number of MRSA positive patients in the trust as a whole and in the ITU
- the number of MRSA patients isolated in side rooms, isolation wards, or cohorts or not isolated

- the number receiving anti-MRSA systemic antibiotics .

Section 4: Anonymised, confidential individual patient level data was requested for **all** newly identified MRSA positive patients who were detected on admission or pre-admission screening during the audit week (9th May 2011 to 15th May 2011). Infection control teams were asked to make all reasonable efforts to interrogate relevant data bases, review the medical and nursing notes and, if possible, the patient, and discuss with nursing staff. If there was no documented, clinical or database evidence of these risk factors it was assumed they were not present.

- Age and sex.
- Dates of admission and discharge (if applicable), date swab was taken and date result became available.
- Whether it was an acute or elective screen.
- Admitting specialty (categorised by the research team as high-risk or low-risk. (High risk specialties = Nephrology, Haematology/Oncology, Neurosurgery, Cardiothoracic surgery, Trauma/Orthopaedics and Vascular Surgery.)
- Patient management – if and how the patient was isolated, if and when they received decolonisation/suppression therapy.
- Presence of risk factors for MRSA carriage (**1**. Care-home resident. **2**. Transferred from another hospital. **3**. Hospitalised within the last year in this trust **or** another trust. **4**. Presence of skin breaks. **5.** Presence of indwelling devices). If there was a positive record of the presence of one of these risk factors, the risk factor was considered present. If there was no documented or data base evidence of these risk factors then it was assumed the factor was not present. In this way it was hoped to mimic routine clinical practice

Section 5: The same individual patient level data was sought for a randomly selected sample of 5-10 MRSA negative patients who were admitted or screened in that week . Respondents were asked to identify all relevant patients for that week, number them consecutively and identify a random sample using an online research randomiser tool (http://www.randomizer.org/form.htp). As well as the five risk factors for MRSA carriage mentioned above, trusts were also requested to record whether the patient was known previously to be MRSA positive (MRSA +ve).

Methodology: Modelling & Cost-Effectiveness.

In order to address objective 6, the above data were used to inform mathematical models of MRSA transmission in hospital populations. Three models were developed to represent different Trust types; Acute, Teaching and Specialist. Each of the models simulated patient movement within the hospital and between hospital and community populations, transmission of MRSA within the hospital, as well as alternative screening and control strategies.

The models were used to simulate MRSA transmission under each of these alternative strategies in order to compare both the effectiveness and cost-effectiveness of control.

The models (see appendix 3)

An existing dynamic model developed for the DH funded MECAMIP project [Robotham et al 2011], which simulated MRSA transmission within a single hospital ward, was further advanced. In order for the models to be appropriate for evaluation of hospital-level screening policies the following major extensions were performed:

1. The development of a whole hospital model.
2. The inclusion of realistic patient movements (ward transfers and readmissions).
3. Stratification of the admissions into elective or emergency admissions
4. The development of three distinct models representing the different Trust types.

Model structure

Models were stochastic, individual-based and discrete-time and simulated the transmission of MRSA in a whole hospital setting. The models were individual-based allowing the MRSA status of individual patients to be tracked over time.

The model structure is represented schematically in Figure 1, and model assumptions listed below.

Figure 1. Schematic of whole-hospital and community transmission model structure. S=susceptible, or MRSA negative, patient; C=colonised; I = infected. Black lines represent movements, where solid lines are admissions and discharges, dashed lines readmissions and dotted line transfers. Red lines represent transmission events, and green lines death events.

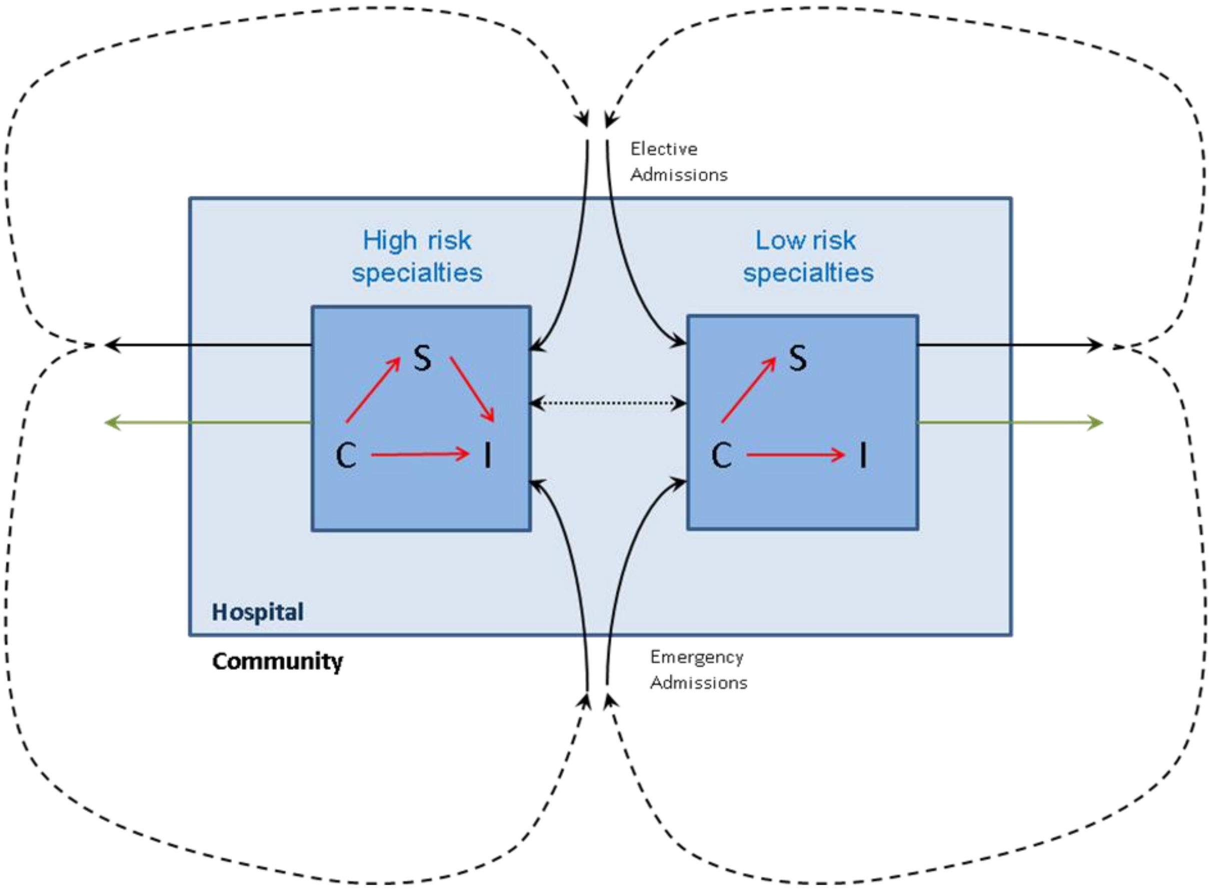

Summary of model assumptions:

- Admissions may be colonised or susceptible according to prevalence (but not infected).
- Prevalence on admission is dependent on whether the patient is categorised as checklist positive (ie having at least one risk-factor for MRSA carriage on the six-item checklist) as well as whether they are admitted via an Elective or Emergency admission route
- No specific assumptions about transmission routes are made.
  - The instantaneous risk of a susceptible patient becoming colonised increased linearly with the ward-level MRSA prevalence.
- Colonised and infected patients are equally infectious.

- Transmission parameters (infectiousness of colonised/infected individuals, probability of progression and susceptibility to colonisation/infection) are specialty dependent.
- Direct infection from a susceptible state cannot occur in low risk specialty settings and patients must first become colonised.
- Once MRSA positive, patients remain so for the duration of their stay.
- All infected patients are suspected to be so, with a delay of 1 day before a clinical specimen is taken.
- Recovery may occur in the community.
- At any time patients may belong to either high-risk (HR) or low-risk (LR) specialties
  - Parameters may differ between specialties
  - No transmission can occur between specialties.
- Length of stay of colonised and susceptible patients is modelled using the same daily probabilities of discharge, only infection increases length of stay
- Similarly, additional mortality is associated with infection only
- Daily probability of discharge and death is dependent on whether the patient is in a high-risk or low-risk specialty as well as their infection status.
- 100% bed occupancy.

Uncertainty

For parameters determining effectiveness of the intervention method used, values were defined as probability distributions rather than point estimates. These distributions were chosen to represent the uncertainty in each of the parameters and are assumed independent. In each model simulation run, a parameter value was sampled from these distributions. Since different simulations draw different parameter values from these distributions, the model outcomes also vary between simulations. In this way, uncertainty could be propagated through the model.

In addition to the uncertainty in the parameter values, chance also enters the model due to the stochastic nature of the transmission process. While it is important to account for such stochastic effects when evaluating different strategies, uncertainty in model outcomes should reflect only parameter and structural uncertainties in the models. Therefore, for each sample of parameter values we performed a large number of runs and recorded the mean value of the outcomes of interest. Specifically, we selected 50 parameter sets (each with a different value pulled from the probability distribution for intervention effectiveness) and ran the model 1000 times for each parameter set. Therefore for each strategy, we performed a total of 50,000 model runs.

The model was programmed using the C++ language and open-source libraries. As hundreds of thousands of model runs were required to compare the strategy options; these were performed on a high performance cluster. Analysis and graphical representation of the large amount of generated output was performed in R 2.10.1 (www.r-project.org).

Cost-effectiveness analysis

Health economic data were incorporated into the model to explore the direction and size of changes in economic costs and health benefits due to interventions, through a cost-effectiveness analysis (Graves et al, 2004), allowing comparison of each screening policy. Incorporation of economic parameters into a transmission dynamic model (as opposed to a static model) allows population-level effects to be accounted for, such effects are important since preventing infection in one individual directly benefits that individual and indirectly benefits others by preventing transmission. Analyses failing to take account of indirect effects may underestimate benefits of interventions (Roberts et al 2004). Health benefits are described using quality adjusted life years (QALYs). The theory by which health benefits may be evaluated using a dynamic simulation process is outlined in Figure 2.

Figure 2: Representation of how a transmission dynamic model can evaluate the impact of an intervention policy on health benefits (measured in QALY).

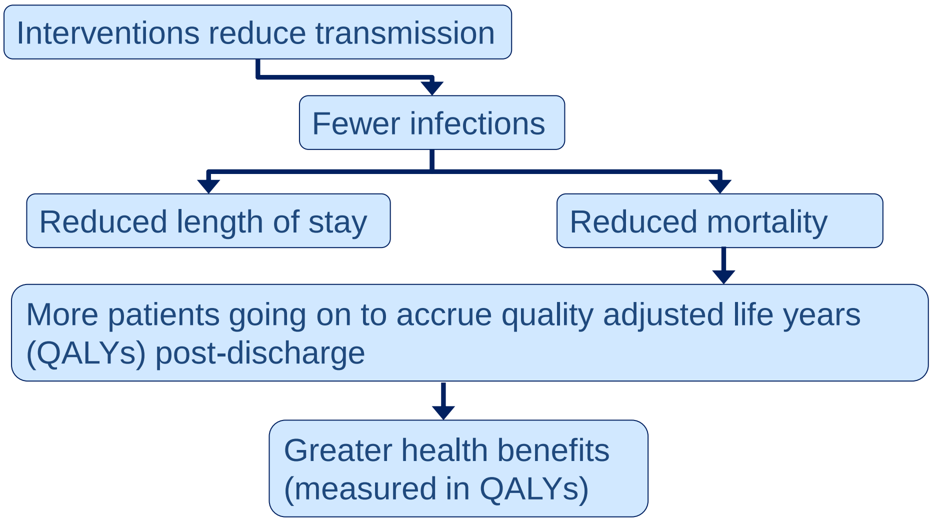

Costs: The transmission dynamic model is used to estimate the number of occurrences (over the simulation period) of each event that incurs costs. Important parameters are the monetary valuation for these events and of associated resources, expressed as unit costs.

Three types of costs incurred were considered in our analyses:

- Infection related costs per day.
- Cost of a bed day
- Intervention related costs.

Health Benefits: Health benefits are described using quality adjusted life years (QALYs). Three measurements were required for analysis of health outcomes:

- bed days accrued
- number of deaths
- number of successful patient episodes (number of patients discharged alive)

This estimate was further adjusted to account for long term effects of MRSA infections.

Cost-effectiveness outputs

We conducted a health-economic evaluation to predict outcomes of each strategy in terms of costs and health benefits, measured in Quality Adjusted Life Years (QALYs). The perspective for this analysis represents the healthcare decision maker at a regional or national level.

Competing interventions were compared against a baseline scenario in terms of their (ICER) incremental cost-effectiveness ratios (the ratio of the change in costs to the change in health outcome compared to the alternative). Strategies were considered cost-effective if they generate an ICER) that is less than the current NHS decision makers willingness to pay threshold of £30,000 per QALY.

Using the economic transmission model we compared policies in different scenarios and settings, firstly in terms of the clinical effectiveness of each policy (in terms of appropriateness of resource use, and number of transmission, infection and death events) followed by the costs of each policy. These effectiveness and costs results are then combined and depicted on cost-effectiveness planes and presented as mean ICERs, allowing direct comparison of alternative strategies.

We also present results in the form of cost-effectiveness acceptability curves (CEACs) and cost-effectiveness acceptability frontiers (CEAFs) which show, respectively, the probability of each strategy having the highest net monetary benefit (NMB) for different values of the willingness to pay per unit of health benefit gained, and the strategy returning the greatest expected NMB.

Model Parameterisation (see appendix 6)

Model parameters were estimated using data from the prevalence audit wherever possible, readmission data from the sentinel audit (see appendices 4a and 4b) with others estimated as in MECAMIP (Robotham et al 2011) or from previously published data.

Where uncertainty associated with model parameters was included, parameters were described using probability distributions. Where NOW audit data were used for parameter estimation, data were stratified according to Trust type.

1) **Population parameters**, the characteristics and sizes of populations modelled.
2) **Movement parameters**, ie patient transfers, discharge and readmission, the additional length of stay and mortality associated with MRSA infection.

3) **Transmission parameters**, describing the susceptibility of individual patients to colonisation and infection.
4) **Intervention parameters**, describing the characteristics and effectiveness of interventions ie isolation and decolonisation.
5) **Health economic parameters**, ie intervention costs, infection costs, additional length-of –stay, quality adjusted life expectancy post discharge.

Methodology sentinel questionnaire(See appendix 5a.)

Data were collected from a sample of trusts to allow more accurate parameterisation of the model for the present study and for future work. All English acute NHS trusts that had responded to the questionnaire were asked whether they would be willing to take part in the Sentinel study. In total 8 trusts returned data – 3 teaching and 5 acute trusts. They represented a range of sizes and covered a wide geographic spread.

Data were collected in the autumn/winter of 2011 for the following parameters:

Section A. Proportions of patients readmitted within 30 days. Data were collected retrospectively for the financial year 2010-2011 for MRSA positive patients and all patients.

Section B. Number of ward stays per patient admissions. Data were collected retrospectively for the financial year 2010-2011

Section C. Discharge and transfer destinations of ITU patients. Data were collected retrospectively for the financial year 2010-2011

Section D. Numbers of ITU wards per trust, number of ITU beds and number of ITU isolation rooms

Section E. Estimated extra resources that would be required to introduce routine PCR admission screening in the trust (staff and capital)

Section F. Annual antibiotic spend for antibiotics commonly used in MRSA infection. Data were collected retrospectively for the financial year 2010-2011

RESULTS

Objective 1

To report how widely the policy of screening Emergency and Elective admissions is implemented across the NHS in different hospitals and patient groups.

Response rates to the questionnaire were excellent, 144 (86.2%) of trusts returned a questionnaire, of which 143 (85.6%) returned a response to sections 1-3 of the questionnaire (Table 1). Where data are presented broken down by trust type (acute, teaching and specialist), data from two trusts, for which it was not possible to identify the trust type are included under the "All" category.

Table 1: Proportion of trusts returning a questionnaire

	All	Acute	Specialist	Teaching
Proportion	144/167 (86.2%)	100/121 (82.6%)	19/20 (95%)	23/26 (88.5%)

<u>Questionnaire section 1:</u> Numbers of acute & elective admissions and admission screens for the week beginning 11th April 2011

In total there were 93021 reported emergency admissions in 136 trusts and 129 692 elective admissions (including day-cases) in 140 trusts. Elective admissions were more likely than emergency admissions to be admitted to "high-risk" specialties (haem/onc, nephrology, cardiothoracic surgery, neurosurgery and trauma/orthopaedics (7.3% and 14.75% of all admissions respectively).

The proportion of electives, emergencies and day-cases screened for MRSA on admission is summarised in Table 2. Proportions were highest among patients admitted as electives (81.8%) and lowest in those admitted as day-cases (47.9%).

Table 2: Proportion of admissions screened for MRSA *

Admission category	All	Acute	Specialist	Teaching
Emergency	52788/87165 (61%) Median 67.3% IQR (47.5%-85.8%) (n= 132 trusts)	38127/63577 (60%) Median 67.1% IQR (47.4-85.8% n=91	657/1166 (56%) Median 85.9% IQR (68.3-100%) n=16	13736/21988 (62.5%) Median 59.4% IQR (48.9-89.2%) n=22
Elective (not including day-cases)	22773/27838 (81.8%) Median 90% (IQR 58-118%) (n= 115 trusts)	14477/16497 (87.7%) Median 92% IQR (59-136%) n=77	1652/2191 (75.4%) Median 86% IQR (62-100%) n=16	6569/9044 (72.6%) Median 73% IQR (30-102%) n=20
Day-cases (not including dermatology, endoscopy, ophthalmic and paediatrics)	22416/46777 (47.9%) Median 41.4% IQR (23.2-78.9%) (n =110 trusts)	14255/32927 (43.3%) Median 36.5% IQR (17.4-73.9%) n=77	1153/1568 (73.5%) Median 67.3% IQR (42.6-100%) n=13	6894/11927 (57.8%) Median 48.3% IQR (36.1-77.7%) n=19

*Since a significant proportion of the elective screens taken in the audit week were for patients not yet admitted, and the denominator was the numbers admitted in that week, proportions of screens in some trusts was greater than 100%..

Differences in proportions of patients screened on admission were calculated comparing Emergency with Elective admissions and Emergency with Day-Case admissions. Emergency admissions were significantly less likely to be screened than Elective admissions (z-ratio=-65.007, p=<0.002) and significantly more likely to be screened than Day-Cases (z-ratio=44.444, p=<0.0002.) Elective admissions were significantly more likely to be screened than Day cases (z-ratio=91.594, p=<0.0002).

Objective 2.

To report the prevalence of MRSA carriage on admission in Emergency and Elective admissions in different types of hospital and the proportion of carriage that was previously unknown.

In those trusts for which data on numbers of admissions *and* admission screens were available, only 2.1% of emergency screens and less than 1% of elective and day-case screens were MRSA +ve (Table 3). Just 1%, 0.6% and 0.5% of emergency, elective and day-case screens were from patients who were newly identified as MRSA +ve (Table 4). In order to identify one new MRSA +ve patient, therefore, 102, 180 and 186 screens would need to be taken respectively for emergency, elective and day-cases (Table 5*)*.

Table 3: Proportion of admission screens MRSA +ve

Admission category	All	Acute	Specialist	Teaching
Emergency	1075/52064 (2.1%) Median 1.6% IQR (1.1-2.7%) (n= 129 trusts)	836/37408 (2.2%) Median 2% IQR (1.2-2.7) n=90	5/652 (1%) Median 0% IQR (0-0.2%) n=16	230/13736 (1.7%) Median 1.7% IQR (1.1-2.4%) n=22
Elective (not including day-cases)	188/20798 (0.9%) Median 0.7% IQR (0-1.9%) (n= 101 trusts)	110/13532 (0.8%) Median 0.7% IQR (0-1.8%) n=68	25/1488 (1.7%) Median 0.7% IQR (0-2.5%) n=15	53/5703 (0.9%) Median 0.5% IQR (0.3-1.5%) n=14
Day-cases	150/21501 (0.7%) Median 0% IQR (0-1%) (n= 112 trusts)	58/13509 (0.4%) Median 0% IQR (0-0.6%) n=76	6/1062 (0.6%) Median 0% IQR (0-1.1%) n=16	85/6816 (1.2%) Median 0.7% IQR (0.3-1.2%) n=19

Differences in proportions of patients found to be MRSA +ve on admission were calculated comparing Emergency with Elective admissions and separately comparing Emergency with Day-Case admissions. Emergency admissions were significantly more likely to be MRSA +ve than Elective admissions (z-ratio=-10.843, p=<0.0002) and Day-Cases (z-ratio=13.179, p=<0.0002). Elective admissions were significantly more likely to be MRSA +ve than day-cases (z-ratio=2.382, p=0.0172).

Table 4: Proportion of admission screens newly positive for MRSA

**Admission category**	**All**	**Acute**	**Specialist**	**Teaching**
Emergency	498/50739 (1.0%) Median 0.9 % IQR (0.4-1.3%%) (n = 127 trusts)	374/36083 (1.0%) Median 1.0 % IQR (0.5-1.5%%) n=88	4/652 (0.6%) Median 0% IQR (0-0%) n=16	119/13736 (0.9%) Median 0.8% IQR (0.5-1.3%) n=22
Elective (not including day-cases)	107/19283 (0.6%) Median 0.4% IQR (0-1.2%) (n = 98 trusts)	68/12953 (0.5%) Median 0.4% IQR (0-1.7%) n=68	16/1346 (1.2%) Median 0.4% IQR (0-1.5%) n=14	23/4909 (0.5%) Median 0.5% IQR (0-1.4%) n=15
Day-cases	79//20461 (0.4%) Median 0% IQR (0-0.1%%) (n = 110 trusts)	27/12469 (0.2%) Median 0% IQR (0-0.2%) n=74	5/1062 (0.5%) Median 0% IQR (0-0%) n=16	47/6816 (0.7%) Median 0.1% IQR (0-0.7%) n=19

Differences in proportions of patients found to be newly MRSA +ve on admission were calculated comparing Emergency with Elective admissions and separately comparing Emergency with Day-Case admissions. Emergency admissions were significantly more likely to be newly MRSA +ve than Elective admissions (z-ratio=-5.449, p=<0.0002) and Day-Cases (z-ratio=5.825, p=<0.0002). The number of newly MRSA +ve was not significantly different when Electives were compared to Day cases (z-ratio= 0.234, p=0.815).

Table 5: Number needed to screen (to identify one new MRSA positive)

	All	Acute	Specialist	Teaching
Emergency	101.9	96.5	163	115
Elective (not including day-cases)	180.2	190.5	84.1	145.0
Day-cases	186.0	461.8	212.4	145.0

Objective 3.

To report on screening, isolation and decolonisation policies and practices, laboratory methods and costs.

<u>Questionnaire section 2:</u> Trust practice around MRSA admission screening

Screening:

In section 2 of the questionnaire trusts were asked to report screening protocols. Following DH advice, more than two thirds of trusts did not screen dermatology-, ophthalmic-, and dental day-cases, low risk paediatrics or endoscopy patients (Table 6). Acute trusts were significantly more likely to exclude groups from admission screening than specialist trusts. Fifty per cent of specialist trusts excluded no patients from screening compared to 5% of acute trusts (z-ratio=-5.435, p=<0.0002). Acute trusts were no more likely to exclude paediatric (z-ratio=1.723, p=0.0849) or emergency maternity admissions (z-ratio=0.911, p=0.3623) compared to teaching trusts. Statistically significant differences for other categories not calculated.

Table 6: Which groups do you routinely exclude from MRSA admission screening? (more than one response possible**)**

	All trusts= 143	Acute trusts=100	Specialist trusts=18	Teaching trusts=23
None	14 (9.8%)	5 (5%)	9 (50%)	0 (0%)
Dermatology day-cases	101 (70.6%)	79 (79%)	2 (11.1%)	18 (78.3%)
Ophthalmic day-cases	105 (73.4%)	81 (81%)	3 (16.7%)	19(82.6%)
Endoscopy	109 (76.2%)	84 (84%)	3 (16.7%)	20 (86.9%)
Paediatrics (low risk)	111 (77.6%)	88 (88%)	4 (22.2%)	17 (73.9%)
Emergency Maternity	68 (47.6%)	54 (54%)	2 (11.1%)	10 (43.5%)
Dental day-cases	106 (74.1%)	82 (82%)	3 (16.7%)	19 (82.6%)
Other	37 (25.9%)	26 (26%)	4 (22.2%)	6 (26.1%)

All 143 trusts reported that nasal swabs were taken as part of admission screening, with the majority also screening groin and/or perineum plus another site (Table 7).

Table 7: Which sites are routinely swabbed as part of MRSA admission screening?

	Emergency trusts =143	Elective trusts =142
Nose only	8 (5.6%)	11 (7.8%)
Nose and groin/perineum only	3 (2.1%)	7 (4.9%)
Nose and groin/perineum and any other *site.	108 (75.5%)	98 (69%)
Nose and any other site (excluding groin and perineum)	24 (16.8%)	26 (18.3%)

*"Other" includes wounds, indwelling devices, throat etc.

Laboratory Testing:

The most common laboratory technique for processing both elective and emergency admission swabs was chromogenic agar plating with more than 80% of trusts using this as their main technique (Table 8). Only a very small number of trusts (7: 4.9%) were *routinely* using PCR for the processing of emergency admission screens and just one trust used PCR as the routine method of screening elective admissions. Reported costs for laboratory processing per swab are also reported in Table 8. As might be expected, the most commonly used technique (chromogenic agar plating) was also one of the cheaper options.

Table 8: Routine microbiological tests for emergency and elective admission screens

	Emergency n =142 trusts	Elective n =141 trusts	Mean cost per swab
PCR	7 (4.9%)	1 (0.7%)	£25.86 (sd £9.64)
Rapid enrichment	0 (0%)	0 (0%)	£14.25 (sd £3.54)
Chromogenic agar	116 (81.7%)	123 (87.2%)	£5.68 (sd £4.44)
Broth enrichment	15 (10.6%)	14 (9.9%)	£4.77 (sd £2.52)
Other	4 (2.8%)	3 (2.1%)	-

Chromogenic agar plating was the most commonly used laboratory method for admission and elective screens. There was no statistically significant difference between the proportions of trusts using this method for emergency and elective admission screening (z-ratio=-1.287, p=0.1981). Although more trusts stated they would use PCR for routine processing of emergency admission screens compared to elective screens, the difference was not significant (Fisher's Exact test, p=0.066).

Admission swabs were generally tested separately [85/143 trusts: 59%] rather than pooled, and the overwhelming majority [122/143: 85%] processed samples within the trust with a further 18 [13%] sending samples to another trust.

Although PCR was not the routine test for most admission screens, a minority of trusts used PCR routinely for admission screening in selected patient groups. PCR was used by22/141 [15.6%] trusts some emergency screening and in 15/142 [10.5%] trusts for at least some elective screening. In these cases, PCR screens were generally processed in a central laboratory [25/30 trusts: 83%]. A further 2 trusts processed samples using point of care testing and one used a hot-laboratory. PCR tests were run daily in 8/20 [40%] trusts: twice daily in 8 trusts [40%] and more than twice daily in the remaining four trusts.

Patient Management – isolation/contact precautions/decolonisation:

The majority of trusts initiate some form of action on admitted patients aimed at preventing the spread of MRSA before confirmation of screen results. Most trusts reported that previously known MRSA patients or "high risk" patients (i.e. those admitted to locally-defined high risk areas or those with particular risk factors for MRSA carriage) are either given suppression/decolonisation therapy and/or undergo contact precautions and/or are isolated (Table 9). Whereas only a small number of trusts (10/143 [7%]) do not routinely isolate at least some patients before results are available, a significant number (49/143 [34%], do not initiate suppression/decolonisation pre-emptively on any admitted patients. By way of contrast, 121/143 (84.6%) of trusts offer suppression/decolonisation to patients identified as MRSA +ve at pre-admission clinics.

Table 9: Trusts initiating precautions on *admitted* patients before MRSA results available. (more than one response possible)

	Suppression/decolonisation trusts =143	Contact precautions trusts =143	Isolation trusts =143
All patients	14 (9.8%)	30 (21.0%)	2 (1.4%)
All previous MRSA +ves	50 (35.0%)	85 (59.4%)	110 (76.9%)
High risk patients *	59 (41.3%)	33 (23.1%)	63 (44.1%)
Other	3 (2.1%)	5 (3.5%)	12 (8.4%)
None	49 (34.3%)	20 (14%)	10 (7.0%)

* locally defined (see text above)..

First line decolonisation/suppression regimes appear to be relatively consistent across trusts. All but 11 trusts reported the use of nasal Mupirocin ointment as a part of their suppression/decolonisation regime. Most trusts combined this with chlorhexidine (72trusts), octenidine (51 trusts) or triclosan body-washes (17 trusts) (Table 10).

Table 10: Numbers of trusts reporting first line suppression/decolonisation regimes for MRSA +ve patients (more than one response possible)

	Trusts =143
Mupirocin only	1 (0.7%)
Mupirocin + Chlorhexidine wash	72 (50.3%)
Mupirocin + Triclosan wash	17 (11.9%)
Mupirocin + Octenidine wash	51(35.7%)
Mupirocin + other	16(11.2%)
Neomycin (nasal) + chlorhexidine	6(4.2%)
Other	5 (3.5%)

Objective 4 (point prevalence data)

To report on how patients were managed- how soon were results available, and how many patients were isolated and / or decolonised pre-emptively and after the result was known, and how many were treated for infection.

Questionnaire section 3: Point-prevalence data for Wednesday 11th May 2011

Data requested included bed and ward numbers, isolation capacity/numbers of MRSA +ve patients and MRSA patient management/treatment.

On the audit day 140 trusts reported a total of 3076 MRSA +ve inpatients (3.3% of all reported inpatients). Acute trusts had significantly higher proportions of MRSA +ve patients than both specialist (z-ratio=4.599, p=<0.0002) and teaching trusts (z-ratio=3.989, p=<0.0002). Teaching trusts had significantly higher proportions of MRSA +ve patients than specialist trusts (z-ratio=-3.132, p=0.0017).

Of the 3033 patients for whom data were available 1837 (60.5%) were isolated in side-rooms, 82 (2.7%) in a designated ward and 107 (3.5%) in a cohort. The remaining third were not isolated (Table 11). Specialist trusts were significantly more likely than acute (Fisher's p<0.0001) and teaching trusts (Fisher's p<0.0001) to isolate MRSA+ve patients.

In addition, 127 (88.8%) of responding trusts were able to supply data on the numbers of MRSA +ve inpatients receiving antibiotic treatment for any MRSA infection on the audit day. They reported that 286/2680 (10.7%) of MRSA positive patients were receiving antibiotic treatment (vancomycin, teicoplanin, doxycycline, linezolid, rifampicin or fusidic acid). Acute trusts reported a higher proportion of patients receiving antibiotic therapy than teaching trusts (z-ratio=-2.339, p=0.0193).

Table 11: Numbers and proportions of MRSA +ve inpatients, patients in isolation and patients receiving antibiotic therapy for MRSA on 11th May 2011

	All	Acute	Specialist	Teaching
% patients with MRSA	3076/92619 (3.3%) Median 2.9% IQR (1.8-4%) (trusts =140)	2191/61628 (3.6%) Median 3% IQR (2.1-4.8%) n=98	65/3205 (2.0%) Median 1.4% IQR (0.9-3.4%) n=18	758/25146 (3.0%) Median 3.1% IQR (1.9-3.8%) n=22
% isolated	2026/3033 (66.7%) Median 77.8 IQR (53.5-100%) (trusts =139)	1459/2191 (66.5%) Median 75% IQR (50-95.4) n=98	64/65 (98.5%) Median 100 IQR (100-100%) n=17	453/715 (63.4%) Median 73.1% IQR (51.9-93.8)- n=22
% receiving antibiotic therapy	286/2680 (10.7%) Median 9.1% IQR (0-16.7%) (trusts =127)	188/1923 (9.8%) Median 9.1% IQR (0-16.5%) n=90	8/63 (12.7%) Median 0% IQR (0-42.5%) n=17	90/694 (13.0%) Median 12.5% IQR (7.1-71.1%) n=20

Objective 4 contd (patient level data)

Questionnaire sections 4 and 5: Individual patient level data for newly identified MRSA positives and MRSA -ve patients during the audit week (09/05/2011 – 15/05/2011)

Total numbers

MRSA +ves: A total of 130 trusts returned forms with details for MRSA +ve patients newly identified during the audit week. A further 14 trusts did not have any patients who met the inclusion criteria. Data were received for 760 patients (mean per trust 5.3 [sd 4.96], median 4.5 [IQR 2-7].

MRSA –ves: A total of 141 trusts returned forms with details of MRSA -ve patients who were admitted *or* screened during the audit week. Each trust selected a random sample of 5-10 relevant patients. Data were received for 951 patients.

Patient Characteristics:

Overall proportions of female patients were 50.1% (MRSA +ve) and 54% (MRSA –ve) (Table 12). Differences between the two groups were not significantly different (z-ratio = 1.525, p=0.1273).

Table 12: Patient gender (newly identified MRSA +ves and MRSA -ves: audit week)

	MRSA +ves n=760	MRSA -ves n=951
Female	381 (50.1%)	512 (54%)
Male	376 (49.5%)	437 (45.8%)
Missing	3 (0.4%)	2 (0.2%)

Mean age was, however, greater in the MRSA +ves (67.43 sd21.2) compared to MRSA –ves (58.53, sd 21.8) (Table 13). This difference was found to be statistically significant (t=8.44, df 1689, p=<0.0001). Patients admitted to specialist trusts were generally younger than those admitted to acute and teaching trusts (50.1 years: MRSA-ves, 51.9 years: MRSA+ves)

Table 13: Age in years (newly identified MRSA +ves: audit week)

	MRSA +ves n=760	MRSA -ves n=951
Mean (sd)	67.43 (21.2)	58.53 (21.8)
Median (IQR)	72 (55-85)	62 (43-76)
Missing data	14	6

Patient Admission:

Of the 760 MRSA+ve patients (547: 71.9%) were admitted as emergencies. The proportion of MRSA-ve patients admitted as emergencies was lower (53.9%). This difference was statistically significant (z-ratio = 7.633, p=<0.0002).

In total, 206/760 (27.2%) of the MRSA +ve patients were screened electively (pre-admission screens plus elective on admission screens) and the remaining 547 (71.9%) were admitted as emergencies (Table 14). The proportion of MRSA -ve patients admitted electively was higher, at just under 45%.

Table 14: Method of admission (newly identified MRSA +ves and MRSA -ves screened in the audit week)

	MRSA +ves n=760	MRSA -ves n=951
Emergency on admission	547(71.9%)	513(53.9%)
Elective on admission	49(6.5%)	127(13.4%)
Preadmission screen	157(20.7%)	300(31.5%)
Missing data	7(0.9%)	11(1.2%)

The proportion of MRSA +ve patients and MRSA –ve patients admitted to “high risk” specialties was 17.6% and 24.2% respectively) (Table 15). This difference was found to be statistically significant (z-ratio = -3.291, p=<0.001).

Table 15: Numbers of patients admitted to “high risk” and “low risk” specialties (newly identified MRSA +ves: audit week)

	MRSA +ves n=760	MRSA -ves n=951
High risk**	134 (17.6%)	230 (24.2%)
Med Other	442(58.2**%)**	406 (42.7%)
Surg Other	174(22.9**%)**	307 (32.3%)
Missing data	10(1.3**%)**	8 (0.8%)

**= Haem/onc, nephrology, trauma and orthopaedics, neurosurgery, cardiothoracic surgery, vascular surgery.

Patient Management:

Sample turnaround time (the time between swabbing and the result becoming available) was calculated by subtracting the date that the result was available from the date that the swab was taken. Mean turnaround time was 2.87 days (median 3 days) for MRSA +ve results. The corresponding mean turnaround time for MRSA -ve results was 1.75 days, (median 2 days) (Table 16). This difference was statistically significant.

Table 16: Mean turnaround time (days) for processing of emergency on admission screens (newly identified MRSA +ves and MRSA -ves: audit week)

	MRSA +ves n=534	MRSA -ves n=482
Mean (sd)	2.87 (sd 1.33)	1.75 (sd0.9)
Median (IQR)	3 (sd 2-4)	2 (sd 1-2)

A substantial proportion (219/596:36.6%) of admitted patients who were subsequently found to be MRSA +ve were already discharged before results were available (see Table 17). Despite the faster turnaround time for MRSA -ve samples, a similar proportion of MRSA -ve patients (213:640: 33.3%) had been discharged before the result was available.

There was no significant difference between the two groups (z-ratio = 0.471, p=<0.6376).

Table 17: Number of admitted patients that were still inpatient when result available (newly identified MRSA +ves and MRSA -ves: audit week)

	MRSA +ve n=596	MRSA -ve n=640
Inpatient	376 (62.8%)	412(64.4%)
Discharged	219 (36.6%)	213(33.3%)
Missing data	1 (0.2%)	15 (2.3%)

Of the 596 admitted MRSA +ve patients 15.6% were pre-emptively isolated compared to 5.9% of MRSA –ve patients (table 18). MRSA +ve patients were less likely to be pre-emptively decolonised than MRSA-ves (36/596: 6% versus 59/640: 9.2% respectively).

These differences were found to be statistically significant for the probability of isolation (z-ratio= 5.517, p<0.0002), and decolonisation (z-ratio = -2.096, p=0.0361).

Table 18: Proportion of admitted patients isolated or decolonised pre-emptively (newly identified MRSA +ves and MRSA -ves: audit week)

	MRSA +ve n=596	MRSA -ve n=640
**Isolated**	93 (15.6%)	38(5.9%)
Missing data	55 (9.3%)	90(14.1%)
**Decolonised**	36 (6%)	59 (9.2%)
Missing data	64 (10.7%)	85 (13.3%)

MRSA +ve patients were more likely to have been isolated before the screen result was available than MRSA –ves (z-ratio= 5.517, p<0.0002), but less likely to have received decolonisation (z-ratio = -2.096, p=0.0361).

Once MRSA colonisation had been confirmed 205/376 (54.5%) MRSA +ve patients who were still inpatients were isolated. Decolonisation/suppression therapy was known to have been started in

551/760 (72.5%) patients (including those screened in pre-admission clinics) either before or after confirmation of MRSA status. Data were missing for a further 100 patients (13%).

Proportion of patients checklist positive

Questionnaire section 4: Individual patient level data for those newly identified as MRSA positive on admission screening during the audit week (09/05/2011 – 15/05/2011)

Checklist items MRSA positive patients:

Checklists for risk factors for MRSA colonisation were completed (or partially completed) for 760 patients. Five items were identified (whether the patient had previously been an inpatient in *that* trust or *another* trust was combined into one item). (table 19)

Table 19: Numbers of MRSA +ve patients positive for individual checklist items (760 patients) - (All trusts: 760 patients)

	1.Nursing home	2.Transfer	3a.Previous admission this trust	3b.Previous admission another trust	4.Wound	5.In-situ device
Checklist positive	133 (17.5%)	19 (2.5%)	314 (41.3%)	34 (4.5%)	118 (15.5%)	90 (11.8%)

Any patient who was checklist positive to at least one of the items was defined as "checklist positive". All other patients (including those for whom one or more checklist items were not completed) were defined as "checklist negative". A total of 458/760 (60.3%) patients were found to be "checklist positive". A higher proportion of emergency screens were "checklist positive" (376/542: 69%) compared to those screened electively (72/201: 35.8%) (Table 20). Those admitted via an emergency route to HR specialties were most likely to be "checklist positive".

MRSA +ve patients admitted electively were less likely to be checklist positive than those admitted as emergencies (z-ratio = -8.303, p=<0.0002). High-risk specialty admissions were no more likely to be checklist positive than low-risk admissions (z-ratio = -1.521, p=0.1283).

Table 20: Proportion of MRSA new positives with at least one risk factor for MRSA colonisation (All trusts: 760 patients)

	"High risk" specialty	"Low-Risk" specialty
Elective	23/67 (34.3%)	49/134 (36.6%)
Emergency	50/67 (74.6%)	326/475(68.6%)

Questionnaire section 5: Patient level data for MRSA negative patients screened or admitted during the audit week (09/05/2011 – 15/05/2011)

Checklist items MRSA negative patients: Checklists for risk factors for MRSA colonisation were completed (or partially completed) for 951 patients. The checklist was similar to that completed for the MRSA positive patients, but also included a sixth item (whether the patient had previously been MRSA positive) (Table 21).

Table 21: Numbers of MRSA -ve patients positive for individual checklist items. (All trusts [951 patients])

	1.Nursing home resident	2.Transfer from another trust	3a.Previous inpatient-this trust	3b.Previous inpatient-another trust	4.Wound or skin break	5.In-situ device present	6.Previously MRSA +ve
Checklist positive	33 (3.5%)	24(2.5%)	383(40.3%)	53(5.6%)	49(5.2%)	77(8.1%)	33(3.5%)

Checklist positivity was defined as for MRSA +ve patients. In total 481/951 (50.5%) of MRSA -ve patients were checklist positive. A greater proportion of those screened as emergencies were found to be checklist positive (28/501: 57.3%) than those screened electively (185/426: 43.4%) (Table 22).

MRSA +ve patients were more likely to be checklist positive compared to the MRSA –ves for the following items: Previously resident in a nursing home (z-ratio=9.742, p=0.0002), presence of a wound (z-ratio = 7.184, p=0.0002), presence of an in-situ device (z-ratio = 2.594, p=0.0095). No statistically significant difference was found between the two groups for the following checklist items: transfer from another trust (z-ratio = -0.031, p=0.9753), previous admission to the trust (z-ratio = 0.436, p=0.6628), previous admission to another trust (z-ratio = -1.029, p=0.03035).

Table 22: Proportion of MRSA negatives with at least one risk factor for MRSA colonisation (All trusts: 951 patients)

	"High risk" specialty	"Low-Risk" specialty
Elective	68/159 (42.3%)	117/267 (43.8%)
Emergency	41/66 (62.1%)	246/441 (55.7%)

Checklist positivity using Pathfinder checklist

The checklist used in the Scottish Pathfinder study (Stewart et al 2011), unlike the one used here, did not include a checklist item for patients that had been previously admitted.

As would be expected proportions of patients that were found to be checklist positive were lower for both MRSA +ve and MRSA -ve admissions. (see tables 23 and 24). These data were used to inform a sub analyses looking at the cost-effectiveness of checklist activated screening in acute trusts to allow comparison with results derived from the Pathfinder study.

Table 23: Proportion of MRSA new positives with at least one risk factor for MRSA colonisation (All trusts)

	"High risk" specialty	"Low-Risk" specialty
Elective	15/67: 22.4%	18/134: 13.4%
Emergency	37/67: 55.2%	197/475: 41.5%

Total = 267/743: 35.9%

Table 24: Proportion of MRSA negatives with at least one risk factor for MRSA colonisation (All trusts)

	"High risk" specialty	"Low-Risk" specialty
Elective	17/159: 10.7%	27/267: 10.1%
Emergency	22/66: 33.3%	100/441: 22.7%

Total =166 /933: 17.8%

Objective 5a:

To determine the extra yield of MRSA+ve patients achieved by routine admission screening for the NHS as a whole compared to checklist activated screening

Table 25 reports the numbers of patients that the average trust might be expected to screen per week using current practice (routine screening of all relevant admissions. This represents 100 333.9 screens from 127 trusts.

Numbers reported in the tables were derived from data provided in section 1b in the questionnaire (numbers of MRSA screens [A], numbers MRSA +ve [B] and MRSA –ve [C], numbers of new MRSA positives [D] and previously known MRSA +ves [E]). Numbers of those patients that were likely to be

checklist positive or checklist negative [F-J] were calculated using proportions derived from sections 4 and 5 of the questionnaire (see Table 21 and Table 22) [see also Appendix 6 Tables 1-4 for summary of numbers].

Using routine screening, the average trust would, therefore, carry out 790 admission screens [row A] in a week and identify 5.51 patients [row D] who were not previously known to be MRSA positive (Table 25). Of these 790 patients 397.69 [rows F+H+I] would be checklist positive and 9.1 [rows F+H] MRSA +ve. A small number of MRSA positives (2.23) would be checklist negative [row G]. Routine screening would, therefore, identify 11.33 MRSA +ve patients per week.

The use of checklist activated screening, therefore, would almost halve the numbers of laboratory samples required in a week (from 790 to 397.69) and identify 81% (9.10/11.33) of MRSA +ve admissions.

Table 25: Average numbers of patients per week per trust.

**Screening Categories**	**All patients**
A. Total number of MRSA screens	790
B. Total no of screens MRSA positive	11.33
C. Total no of screens MRSA negative	778.7
D. Total of MRSA +ves newly positive	5.51
E. Total of MRSA +ves previously positive	5.82
F. Total no of new positives, checklist positive	3.28
G. Total no of new positives checklist negative	2.23
H. Total of previous positives checklist positive	5.82
I. Total no of MRSA negatives checklist positive	388.59
J. Total no of MRSA negatives checklist negative	390.11

The use of checklist activated screening, therefore, would almost halve the numbers of laboratory samples required in a week (from 790 [A] to 397.69 [F+H+I]) whilst still identifying 9.10 [F+H]/11.33 [F+G+H] (81%) of MRSA +ve admissions. Equivalent numbers for different trust types are presented in table 26.

Table 26: Average numbers of patients identified per week using checklist activated MRSA admission screening [CAS] by trust type

	All	Acute	Specialist	Teaching
Total no. of MRSA Screens (Routine screening)	790	769.34	182.86	1342.77
Total no. of MRSA Screens (CAS)	397.67	390.31	96.83	672.04
Total no. of MRSA+ves (Routine Screening)	11.33	11.72	2.16	17.22
% of MRSA+ves identified. (CAS)	9.10/11.33 (81%)	9.74/11.72 (83%)	1.62/2.16 (75%)	13.39/17.22 (78%)

Sensitivity and specificity of the screening tool were calculated for the average trust. Sensitivity, i.e. the proportion of MRSA +ves who were checklist positive, was 81%: 9.10/11.33 and specificity, i.e. the proportion of MRSA –ves who were checklist negative, was 50.1%: 390.11/778.70.

Objective 5b.

To determine the extra yield of MRSA positive patients achieved by routine admission screening compared to screening selected "high-risk" specialty admissions.

The average weekly number of patients per trust admitted to high risk specialties is reported in Table 27. Numbers were derived as for objective 5a above. Since infection control teams were not asked to report what proportion of admission screens were collected from patients in high risk specialties, it is assumed that proportions were the same as the proportion of *admissions* to these specialties reported in section 1a of the questionnaire (i.e. 7.3% and 14.75% for emergency and electives respectively).

Table 27: Average numbers of patients screened per week per trust by admissions to High-risk and Low-risk specialties.

Screening Categories	1. High Risk	2. Low Risk
A. Total number of MRSA screens	86.76	703.27
B. Total no of screens MRSA positive	1.05	10.28
C. Total no of screens MRSA negative	85.71	692.99
D. Total of MRSA +ves newly positive	0.52	5
E. Total of MRSA +ves previously positive	0.52	5.29
F. Total no of new positives, checklist positive	0.29	3
G. Total no of new positives checklist negative	0.22	2
H. Total of previous positives checklist positive	0.52	5.29
I. Total no of MRSA negatives checklist positive	42.34	352.68
J. Total no of MRSA negatives checklist negative	43.8	346.31

Compared to routine screening, the number of screens required if only high risk specialty patients were screened would reduce from 790, to 87.76 (Table 27 row A column 1). In all, 1.05 (row B, column 1) MRSA +ve admissions would be identified out of a total of 11.33 (row B column 1+2) representing a pick-up rate of 9.1% . Equivalent numbers for different trust types are presented in table 28.

Table 28: Average numbers of patients identified per week using screening of admissions to "High-Risk" specialties only (by trust type).

	All	Acute	Specialist	Teaching
Total no. of MRSA Screens (High-risk specialties)	86.76	80.85	51.38	238.00
Total no. of MRSA+ves (Routine Screening)	11.33	11.72	2.16	17.22
Proportion of MRSA+ves identified. (High-Risk specialties)	1.05/11.33 (9.1%)	1.00/11.72 (8.5%)	0.52/2.16 (24.1%)	2.69/17.22 (15.6%)

Objective 5c.

To determine the extra yield of MRSA positive patients achieved by routine admission screening compared to screening all admissions to high risk specialties plus checklist activated screening of admissions to other specialties.

Combining the two strategies outlined above (laboratory screening for all admissions to high-risk" specialties and checklist activated screening for all admissions to "low risk specialties would require more laboratory screening for these trusts and would increase the yield slightly compared to screening only those who were checklist positive.

Using this strategy, a total of 86.76 screens would be taken from "high-risk" specialty patients [row A] plus a further 361 from "low-risk" checklist positive patients [rows F+H+I]. In total 9.34 screens would be MRSA +ve (1.05 from "high-risk" specialties and 8.29 from "low-risk" specialties [F+H]). This strategy would require 447.73 laboratory screens per week and, compared to checklist activated screening of all admissions, would increase the number of MRSA +ves detected from 9.10/11.33 (81%)to 9.34/11.33 (82.4%) per week, approximately equivalent to one high risk specialty patient per month. Equivalent numbers for different trust types are presented in table 29.

Table 29: Average numbers of patients identified per week using screening of all admissions to "High-Risk" (HR) specialties and checklist activated screening (CAS) of all other admissions (by trust type).

	All	Acute	Specialist	Teaching
Total no. of MRSA Screens<br>(HR + CAS)	447.73	430.92	124.26	795.25
Total no. of MRSA+ves<br>(Routine Screening)	11.33	11.72	2.16	17.22
Proportion of MRSA+ves identified.<br>(HR + CAS)	9.34/11.33<br>(82.4%)	10.51/11.72<br>(89.7%)	1.75/2.16<br>(81%)	14.07/17.22<br>(81.7%)

For the NHS as a whole table 29a compares the different numbers of screens that would be done each week and the number of positive patients identified using each of the four screening strategies.

Table 29a. Average numbers of patients across the NHS (167 trust) screened and identified per week by screening strategy.

	Routine screening	CAS (all admissions)	HR specialty screening	HR and CAS screening
Total no of MRSA screens	131,930	66,411	14,488	74,771
Total no of MRSA positives	1,892	1,519	175	1,556

Objective 6:

To use data from the audit questionnaire and reliable cost data to populate an existing model of hospital MRSA transmission to provide predictions of the effectiveness and cost-effectiveness for each type of trust for six different screening strategies:

1) no screening

2) screening all admissions (emergency and elective)

3) screening admissions to "high-risk" specialties only

4) checklist activated screening of all admissions

5) strategy 3 plus checklist activated screening all other admissions

6) strategy 2 (screening all admissions) plus pre-emptive isolation of those known to be previously MRSA positive.

For each Trust type, results are presented results in terms of the *effectiveness* of each strategy, followed by the *costs* of each strategy (split into cost components). Costs and effects are then combined and the results presented as cost-effectiveness planes and as mean incremental cost-effectiveness ratios (ICERs). Probabilistic sensitivity analyses are then presented to include the impact of uncertainty (namely the effectiveness of interventions, which has previously been shown to be the dominating parameter [Robotham et al 2011]), as the plots of multiple model runs under multiple parameter values, showing the full extent of uncertainty in the model outputs.

The uncertainty is considered in the comparison of policies in the cost-effectiveness acceptability curves (CEACs), which show the *proportion of simulations* in which each strategy is cost-effective i.e. how likely each strategy is to be suboptimal for each willingness to pay. Finally, results are presented as cost-effectiveness acceptability frontiers (CEAFs) which show the probability that the strategy with the highest net monetary benefit (NMB) is cost-effective for a given willingness to pay.

Results are also presented for several scenarios:

- For each Trust type we compare strategies under baseline prevalence for each trust type, as well as a prevalence of twice (high prevalence) and half this value (low prevalence).
- For Acute Trusts only (assuming baseline prevalence values) strategies are evaluated where :
    - Transmission parameters in high risk settings were reduced to the midpoint between ICU and general medical ward estimates.
    - Daily death probabilities in high risk specialties were reduced to be equivalent to those in low risk specialties.
- Finally, for Acute Trusts only, under baseline prevalence, strategies are evaluated using the Scottish Pathfinder definition of ‘checklist positive’, which is slightly different to that used in the audit study, to determine who is screened in “checklist activated screening”.

A summary of the cost-effectiveness of the various strategies for different scenarios is given in tables C5 and C6 at the end of the results section.

ACUTE TRUSTS

1. Baseline analyses

Effectiveness

Figure A1 shows the degree to which each of the strategies applied the primary intervention (i.e. use of side rooms accompanied by patients decolonisation) appropriately (i.e isolating MRSA positive patients), inappropriately (i.e. isolating MRSA negative patients) and the number of unisolated days (i.e. days spent by MRSA positive patients out of side room isolation.)

Strategies 2 (screening all admissions) and strategy 6 (screening all admissions with pre-emptive isolation of those known to be previously MRSA positive) do particularly well in terms of appropriate isolation use, but also give most inappropriate isolation days (simply due to a greater number of patients being isolated).

Strategy 6 reduces unisolated MRSA positive bed days only slightly compared to simply screening all patients. This is because those who are infected typically have long stays, so catching a small proportion of the MRSA positive population 'early' through pre-emptive isolation makes little overall difference to unisolated days. This finding would be missed if infection status dependent lengths of stay were not included within the model.

Strategy 3 (screening admissions to high risk specialties) appropriately (and inappropriately) isolates very few patients, with the majority remaining unisolated. This is due to the relatively small proportion of admissions to high risk specialties (16% of beds in Acute Trusts belong to high risk specialties).

Strategies 4 (checklist-activated screening) and 5 screening all high risk specialty patients plus 'checklist activated screening of admissions to low risk specialties) perform between the two extremes described above. However, strategy 4, whilst reducing the amount of appropriate isolation ~30% (compared to screening all patients) reduced inappropriate isolation usage by over 50%. This was because the prevalence in the checklist positive group was approximately 2.6% compared to 1.4% of the overall Acute admission population. Therefore, if isolation capacity is a limiting factor, screening only checklist positive patients may be an option to 'free up' 50% of the isolation capacity.

Figure A1. Primary isolation usage under each screening strategy,

showing appropriate isolation (isolation of patients who are MRSA positive), inappropriate isolation (isolation of MRSA negative patients) and unisolated bed days of MRSA positive patients.

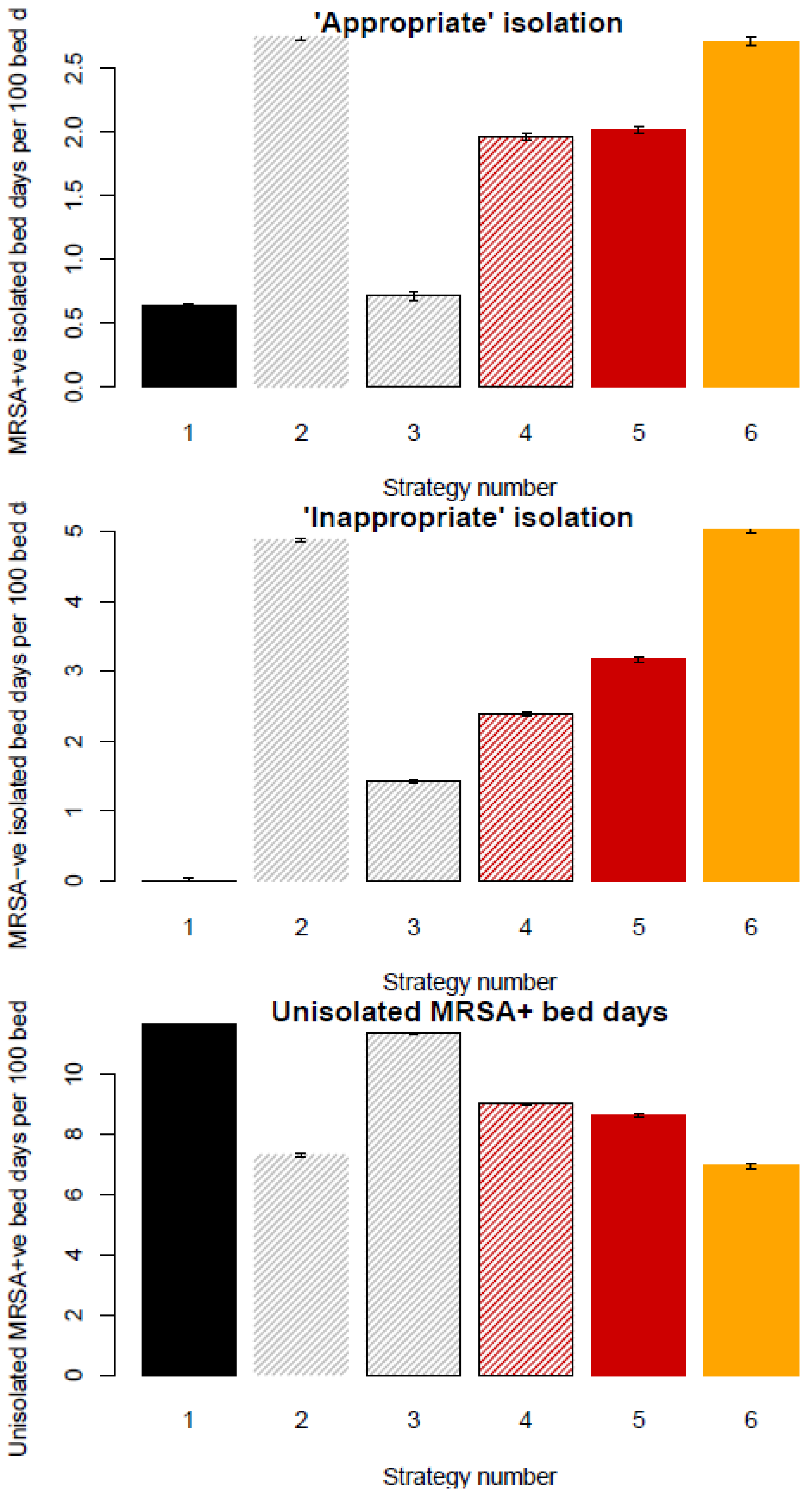

Figure A2 shows the extent to which the ability of each strategy to identify patients for control translates into reduction in transmission, infections and deaths. Strategies 2 and 6 which appropriately isolated the greatest number of patients reduced transmission to the greatest extent. However, overall reduction in transmission did not translate directly into reduction in MRSA infections; this was because some strategies concentrate on high risk specialty patients in whom the probability of acquiring infection was greater in the model than in low risk specialties. Thus, small reductions in transmission in high risk settings had a greater effect on the number of infections and therefore deaths, as the probability of death is directly related to infection status. Strategies with a greater impact on high risk specialties will do most to reduce infections and deaths. Thus strategy 3 (screening all admissions to high risk specialties) only marginally reduced transmission, but reduced infection and death more than strategy 4 (checklist activated screening) although the latter reduced overall hospital transmission more. Strategy 6 (screening all plus pre-emptive isolation of those known to be previously MRSA positive) reduced infections and deaths the most .

Costs

Figure A3 shows total costs of each of the strategies, broken down into their component parts. Aside from Strategy 1 (no screening), Strategy 3 is the cheapest because it screens and isolates the fewest patients. Total costs per admission are dominated by bed day costs.

Figure A2. Patient outcomes under each screening strategy,

showing new acquisitions of MRSA by hospital patients, total MRSA infections in the hospital, and total deaths (all per 100 admissions).

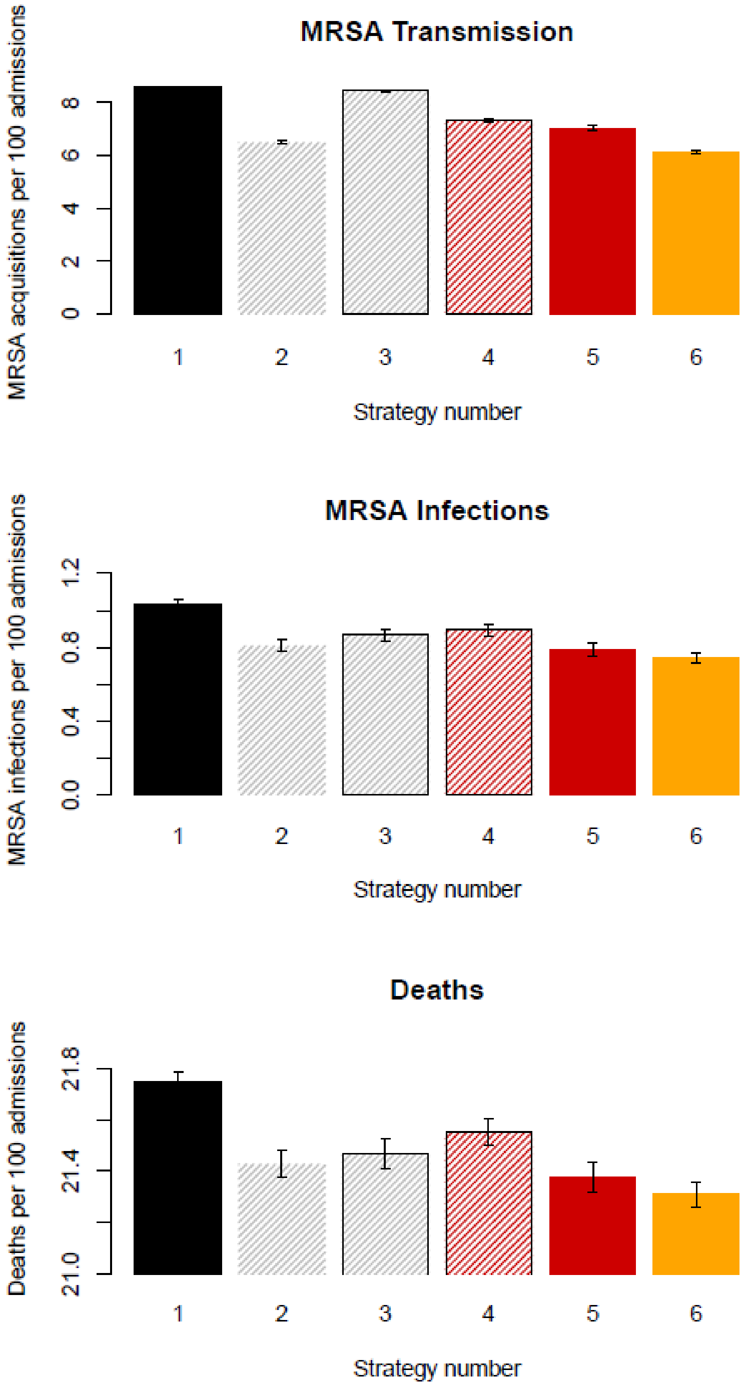

Figure A3. Cost per admission for each strategy,
presented as total costs and broken down into component parts (note different scales of sub graphs).

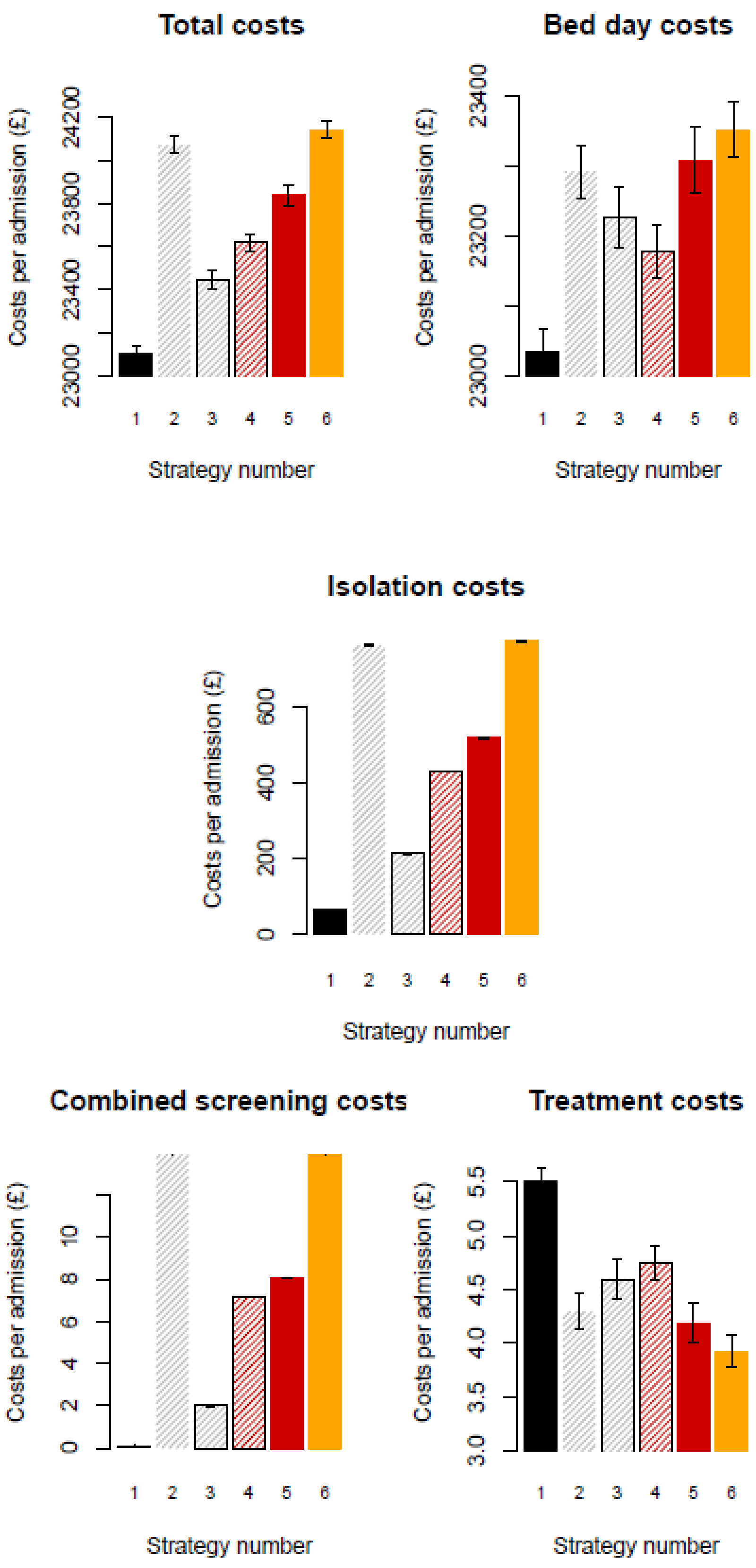

Cost-effectiveness

Each strategy was evaluated on a cost-effectiveness plane where the effectiveness of each strategy was measured in terms of health-benefits (measured in QALYs) per admission. A reduction in the number of infections decreases length of stay (hence cost per admission) and numbers of deaths, resulting in improvements in cost per QALYs gained.

Model results (Figure A4) confirmed that any investment in screening, compared to no screening, was likely to lead to increases in health benefits. Strategy 6 (screening all plus pre-emptive isolation of those known to be previously MRSA positive) gave the highest health benefits, but was associated with the greatest costs.

Figure A4.

Incremental cost-effectiveness plot comparing each of the screening strategies.

Numbers refer to strategies Error bars represent random error brought about by stochasticity and parameter uncertainty, and corresponded to +/- one standard error.

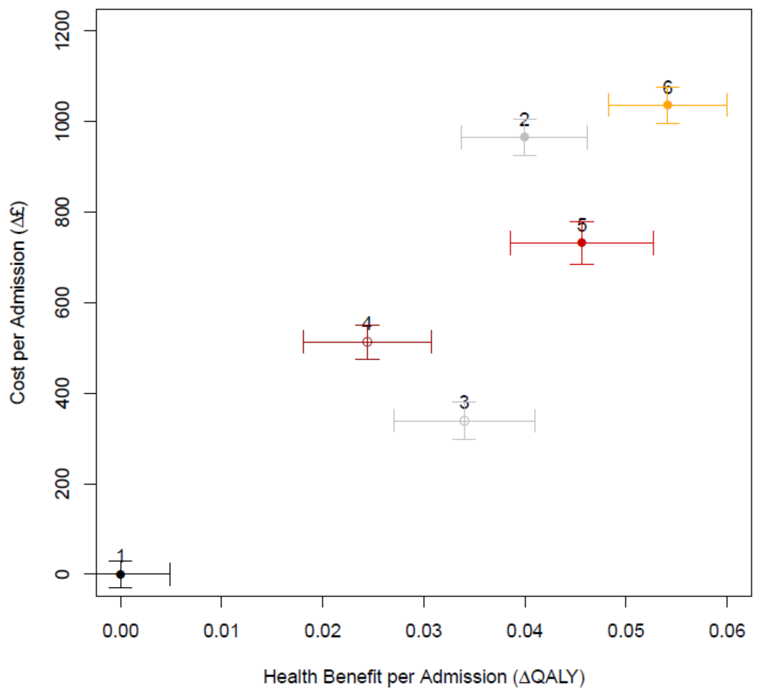

Table A1 describes all strategies in terms of mean change in costs and mean change in health benefits compared to baseline (strategy 1), and then combines these in terms of a mean cost per QALY gained by changing strategy from the baseline ‘do nothing’ approach. Each strategy was also evaluated using the techniques of dominance and extended dominance, allowing some strategies to be eliminated from further evaluation. Dominated strategies were those both more costly and providing less benefit than at least one other strategy. An extendedly dominated strategy was one that is more costly and provides less benefit than a combination of another two strategies. Since it would never be cost-effective to pay more for less benefit, these strategies (2 and 4) were excluded from any further evaluation.

Table A1. Cost-effectiveness evaluation of screening strategies for an Acute Trust.

Screening strategy (ranked by cost)	Cost per admission	QALY per admission	Change in costs, ΔC (compared to baseline)	Change in effects, ΔE (compared to baseline)	Difference in Costs / Difference in Effects	Option evaluation
1	£23,106	8.05123	-	-	-	Remains
3	£23,445	8.08524	£338.86	0.03401	£9,964	Remains
4	£23,619	8.07565	£513.30	0.02441	£21,025	Dominated
5	£23,838	8.09687	£731.94	0.04564	£16,038	Remains
2	£24,071	8.09119	£965.06	0.03996	£24,149	Dominated
6	£24,142	8.10537	£1,036.14	0.05413	£19,140	Remains

Incremental cost-effectiveness evaluations were then applied to the remaining strategies, which form the ‘cost-effectiveness frontier’. Each option was evaluated along the cost-effectiveness frontier, starting at the baseline, strategy 1, to determine whether it was cost-effective (in terms of some maximum threshold of willingness to pay a unit of health benefits, such as a QALY) to move from one strategy to the next most costly strategy on the frontier. This process was iterated, calculating the change in costs and health benefits in moving to the next strategy on the frontier, stopping when no move to a new strategy was cost-effective within the chosen threshold, which for the purposes of this study was the upper threshold of the £20-30,000 per QALY that NHS decision makers tend to use as a willingness to pay threshold (Rawlins et al 2004) This provided mean incremental cost-effectiveness

ratios (ICERs) (Table A2). By this analysis strategy 3 (screening all admissions to high risk specialties), at a mean incremental cost per QALY of £9,964/QALY, would be considered the optimal approach (under the specific model parameters and assumptions used). Although strategy 5, (checklist activated screening) was not cost-effective, it was only marginally above the£30, 000 threshold at £33,806/QALY. Consideration of this option depends on decision-makers willingness to pay for health benefits.

Table A2 Evaluation of cost-effectiveness frontier of strategies for an Acute Trust.

Move between policies	Cost per admission	QALY per admission	Change in costs, ΔC (compared to previous policy)	Change in effects, ΔE (compared to previous policy)	Difference in Costs / Difference in Effects (ICER, cost/QALY)	Option evaluation
Stay at policy 1	£23,106	8.051232	-	-	-	-
Or move:						
to 3	£23,445	8.085241	£338.86	0.03401	£9,964	Cost effective
to 5	£23,838	8.096869	£393.07	0.011627	£33,806	Not cost effective
to 6	£24,142	8.105367	£304.20	0.008498	£35,796	Not cost-effective

Consideration of uncertainty (Figure A5)

When sources of parameter uncertainty (namely the effectiveness of the intervention) are accounted for, choosing between competing strategies becomes more complicated. On the cost-effectiveness plane (Figure A5) we can see that the costs and health benefits for each strategy are highly variable and show considerable overlap. This suggests that no single strategy is likely to be preferred over the entire range of plausible parameter values. The importance of this is clear when considering the cost-effectiveness acceptability curves (CEACs), showing the proportion of simulations in which each strategy is cost-effective (Figure A6) i.e. the probability of each intervention being cost-effective *accounting for the variation in cost/QALY outcome for each model run* at each value of willingness to pay. This shows substantial uncertainty as at willingness to pay values between £20,000 to £30,000 the probability that any one strategy is the most cost-effective option does not exceed 30%, with the probabilities that any one strategy was cost effective clustered together.

Figure A5. **Simulation results comparing each strategy on a cost-effectiveness plane for an Acute Trust setting.** Each dot represents the mean of 1000 simulation runs for each parameter set. The results of 50 parameter sets for each strategy are plotted, where each parameter set is obtained by taking the mean value for all parameters apart from the effectiveness of the intervention, which is sampled from its probability distribution.

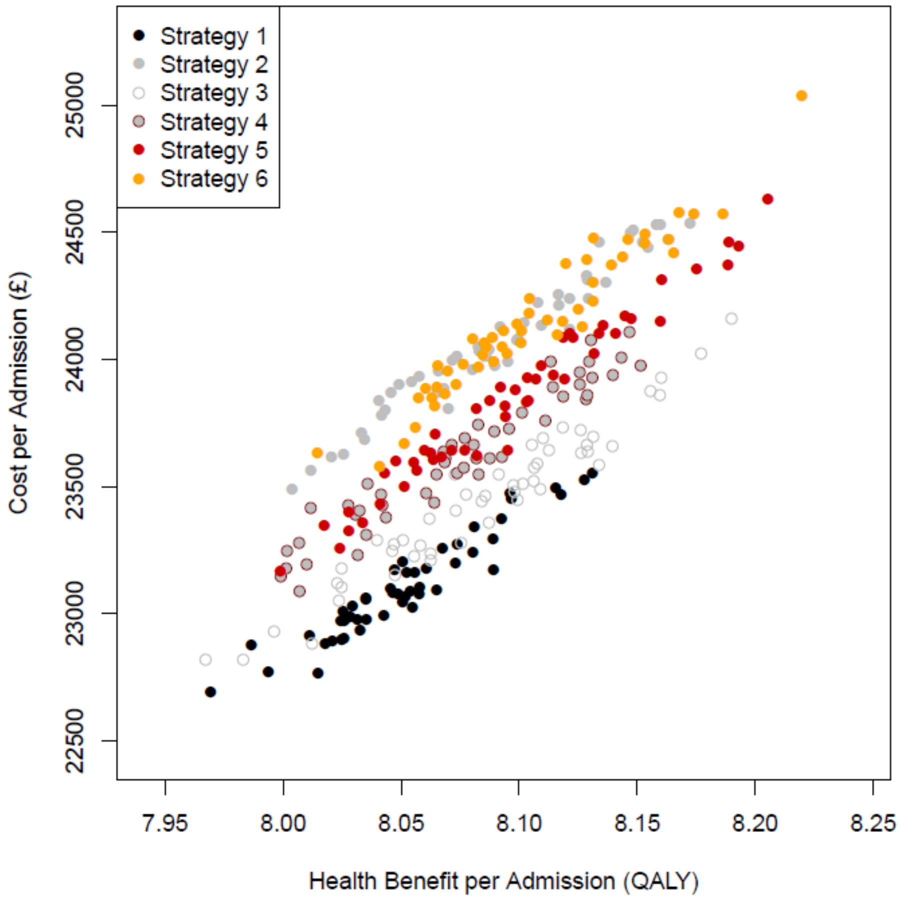

Figure A6. Cost-effectiveness acceptability curves.

Each line represents the proportion of simulations, for a particular strategy, that are cost-effective, as a function of willingness to pay for health benefits.

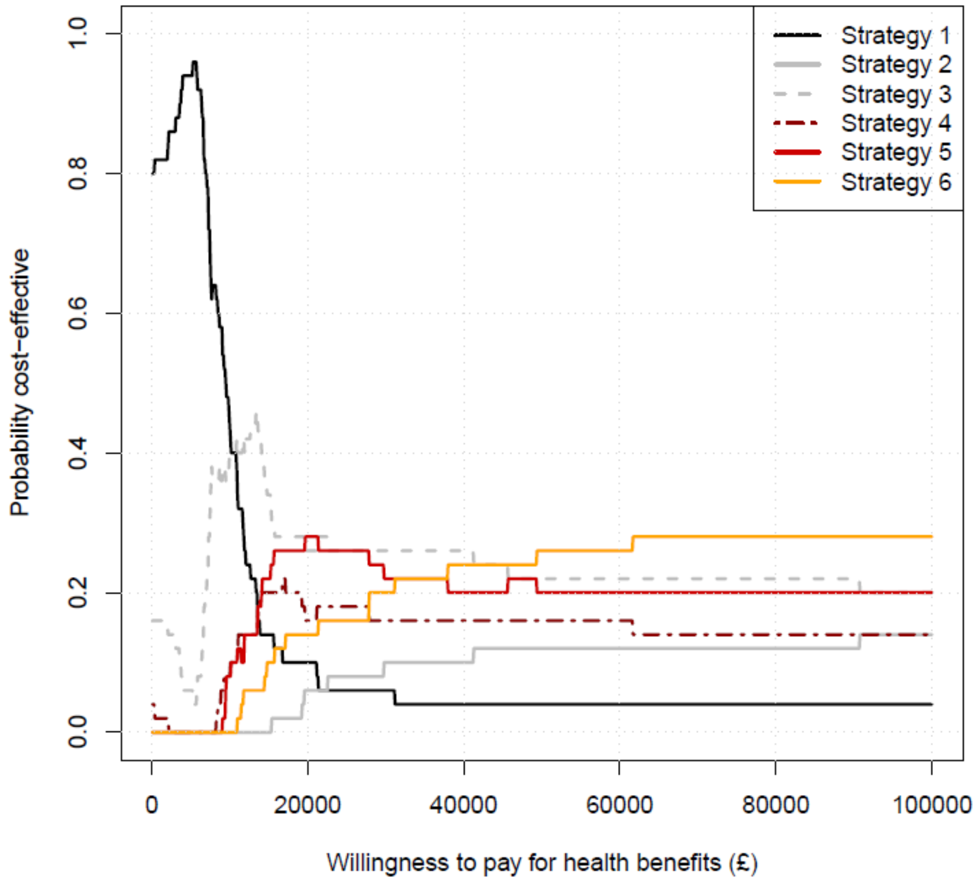

The decision is made slightly clearer by the CEAF (Figure A7), which shows only the strategy with the highest *expected* NMB over the full range of parameter uncertainty, and describes the probability that this strategy is cost-effective for a given willingness to pay. The CEAF therefore shows which strategy the model suggests we should choose for each willingness to pay. It can be seen that Figure A7 is split into 4 sections, meaning that the optimal strategy changes according to the willingness to pay for health benefits. At a willingness to pay of less than approximately £10,000/QALY, strategy 1 (the 'no screening' approach) is optimal. However above this, and up to the NHS willingness to pay threshold of £30,000/QALY, strategy 3 (screening of only high risk specialty patients) is the optimal option.

Figure A7. Cost-effectiveness acceptability frontier.

Lines depict the strategies with the highest expected net monetary benefit, dependent on the willingness to pay for health benefits, while dotted vertical lines the willingness to pay values at which the decision changes.

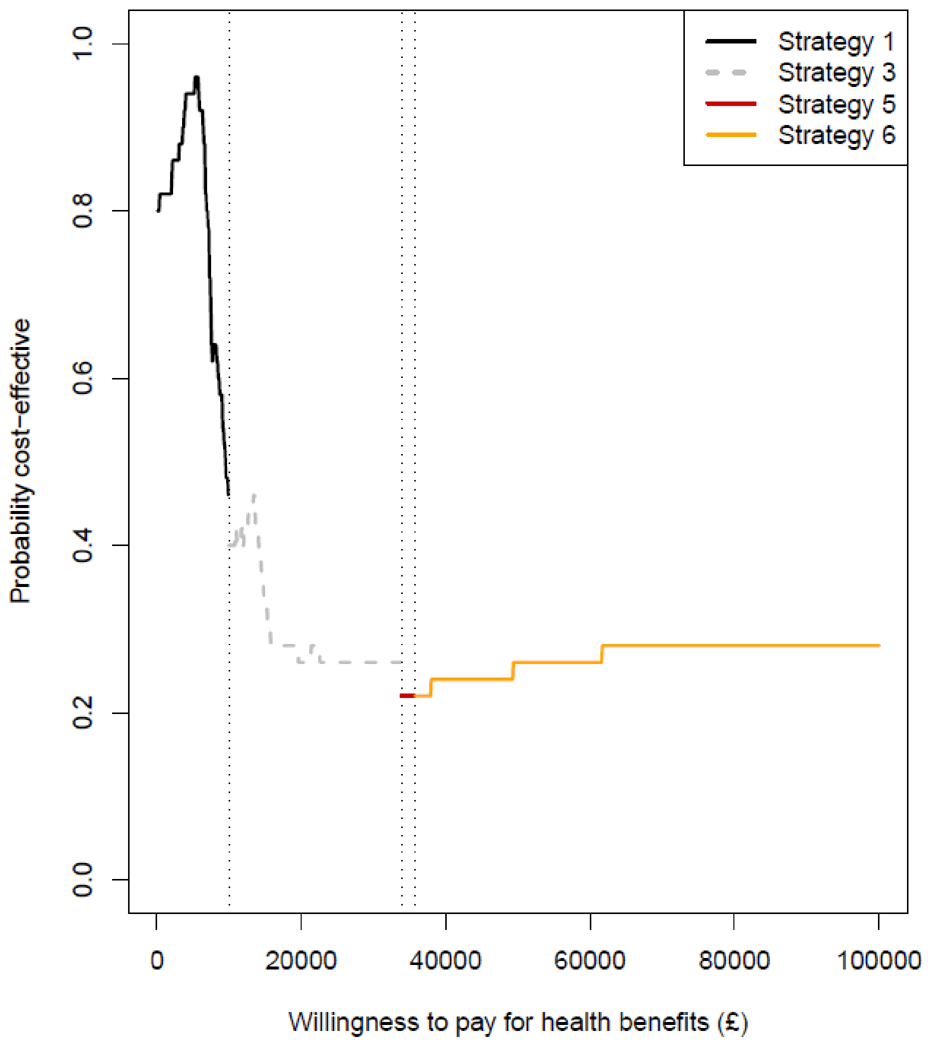

2. Scenario analyses

Scenario a. High MRSA Prevalence*:*

In a high prevalence setting (with admission prevalence twice that found in the audit for Acute Trusts: 2.8% compared to 1.4%) the number of MRSA positive isolated and unisolated bed days are higher as there are more positive patients, not all of whom can be accommodated in isolation facilities. The pattern of differences between strategies is the same as at baseline prevalence (Figure A8 in Appendix 7). The greater prevalence leads to slightly more transmission (more infectious imports to the hospital) and thus slight increases in absolute numbers of infections and deaths (Figure A8). Differences in costs between higher and baseline prevalence settings are minimal (Figure A10 in Appendix 7) however.

The cost-effectiveness plane shows similar overall health benefits per cost accrued as at baseline prevalence (Figure A9) although health benefits were slightly greater as more transmission events and therefore slightly more infections and deaths were prevented. The error bars show overlap between strategies, which is reflected in the uncertainty demonstrated in the CEAC and CEAF plots (Figures A10 and A11).

Evaluation of the cost-effectiveness frontier (Table A3) shows that despite the ICERs shifting slightly, the decision remains the same as in the baseline prevalence setting, with Strategy 3 (screening all admissions to high-risk specialties) the only cost-effective option (at a willingness to pay threshold of £30,000/QALY gained).

Table A3. Evaluation of cost-effectiveness frontier of strategies for an Acute Trust in a high prevalence setting, using a willingness to pay threshold of £30,000/QALY gained.

Move between policies	Cost per admission	QALY per admission	Change in costs, ΔC (compared to previous policy)	Change in effects, ΔE (compared to previous policy)	Difference in Costs / Difference in Effects (ICER, cost/QALY)	Option evaluation
Stay at policy 1	£22,858	7.990682	-	-	-	-
Or move:						
to 3	£23,227	8.033346	£369.03	0.042664	£8,650	Cost -effective
to 5	£23,897	8.048153	£670.14	0.014807	£45,257	Not cost-effective

The CEAC and CEAF figures show very similar levels of uncertainty in the decision between strategies in a higher prevalence setting as in a baseline prevalence setting (Figures A10 and A11) with clustering

around the 20% probability of cost effectiveness for several stategies at the usual NHS willingnes to pay threshold of £20-30,000. However this rose above 50% for strategy 3 below a £20,000 threshold. Strategy 2, screening all patients, has a greater probability of being cost-effective only at higher willingness to pay values (above approximately £45,000/QALY) when it becomes the optimal strategy.

For acute trusts, compared to screening all admissions, restricting screening to high-risk specialty patients would reduce total annual costs by £1,592,000 per acute trust, (£619 per admission), at the expense of 56.5 more transmission events (i.e. one extra colonisation per week per trust) and 1.7 more infections per year. (See table A4) At higher prevalence levels this equated to savings of £1,766,148 (£670 per admission), 63.5 more colonisations (1.2 extra per trust/week) and 2.9 more infections per year. (see table A5)

Table A4 Total costs, colonisations and infections per trust/year (Acute trusts, current prevalence)

	Total admissions	Total bed days	Total costs	Cost per admission	Colonisation events	Colonisation per 100 bed days	Colonisation per admission	Infections	Infection per 100 beddays	Infections per admission
Strategy 2	14427.38	1009225	347020000	24052.88	923.9872	0.091554	0.064044	117.0882	0.011602	0.008116
Strategy 3	14469	1009225	339060000	23433.55	1206.456	0.119543	0.083382	125.6946	0.012455	0.008687
Difference			7960000	619.33	-282.469	-0.02799	-0.01934	-8.6064	-0.00085	-0.00057
Per year			1592000		-56.4937			-1.72128		

Table A5 Total costs, colonisations and infections per trust/year (Acute trusts, high prevalence)

	Total admissions	Total bed days	Total costs	Cost per admission	Colonisation events	Colonisation per 100 bed days	Colonisation per admission	Infections	Infection per 100 beddays	Infections per admission
Strategy 2	14576.31	1009225	3.48E+08	23899	1017.616	0.100831	0.069813	170.5608	0.0169	0.011701
Strategy 3	14616.33	1009225	3.40E+08	23229	1335.175	0.132297	0.091348	185.147	0.018345	0.012667
Difference			8.83E+06	670	-318	-0.031	-0.022	-15	-0.001	-0.001
Per year			1766148		-63.512			-2.91724		

Figure A8. Patient outcomes under each screening strategy, new acquisitions of MRSA by hospital patients, total MRSA infections in the hospital, total deaths (all per 100 admissions)

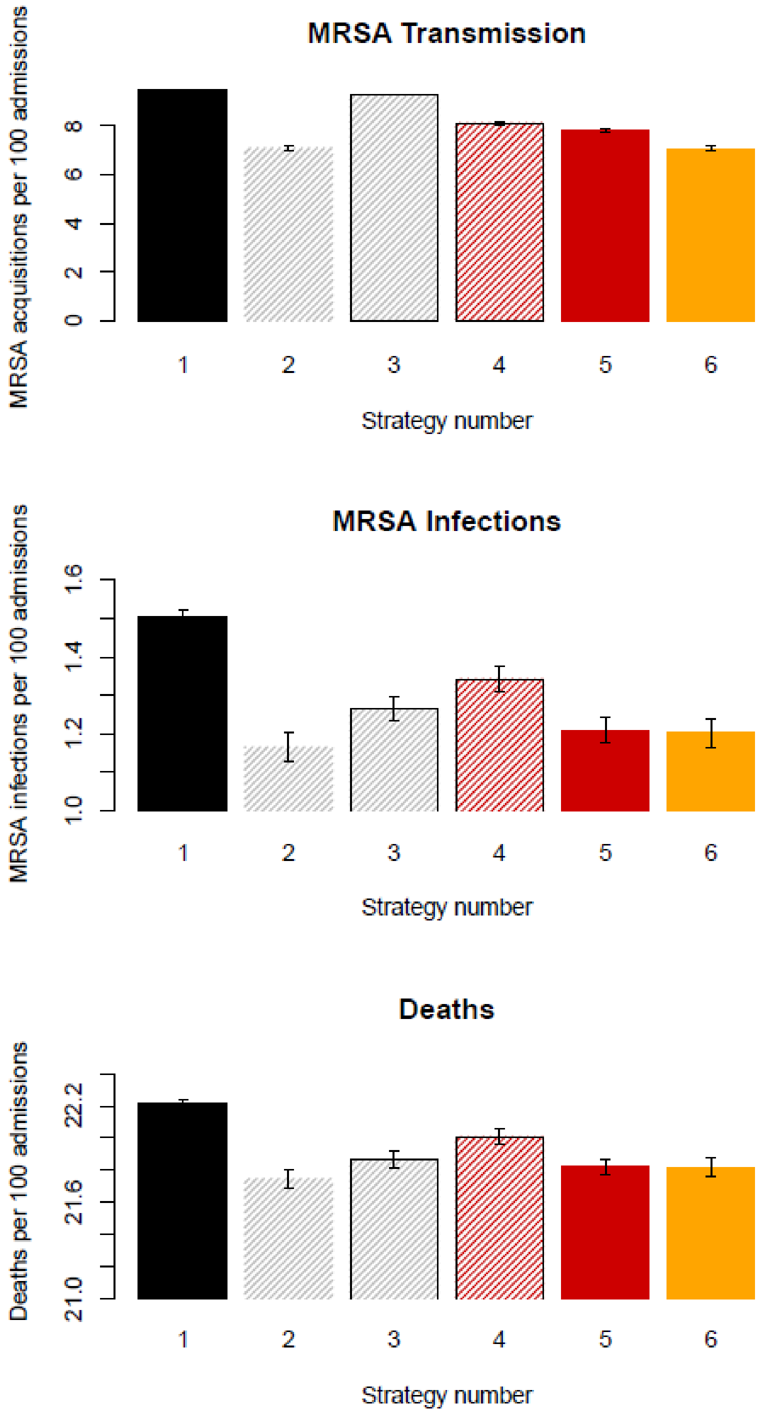

Figure A9. Incremental cost-effectiveness plot comparing each of the screening strategies. Numbers indicate strategy numbers as outlined above. Error bars represent random error brought about by stochasticity in the model and parameter uncertainty, and correspond to plus or minus one standard error.

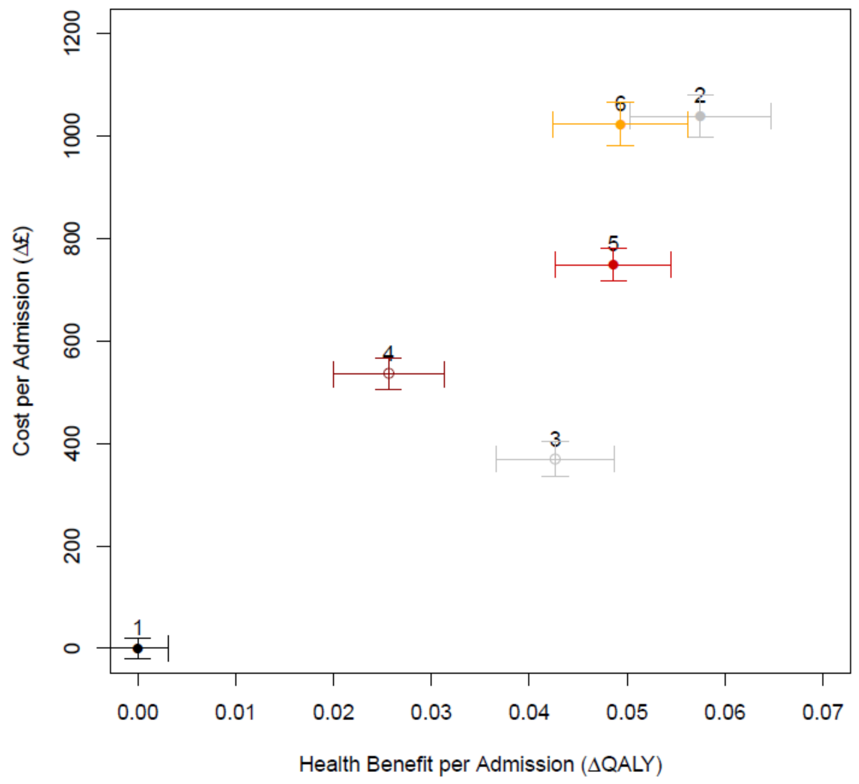

Figure A10. Cost-effectiveness acceptability curves.

Each line represents the proportion of simulations, for a particular strategy, that are cost-effective, as a function of willingness to pay for health benefits

.

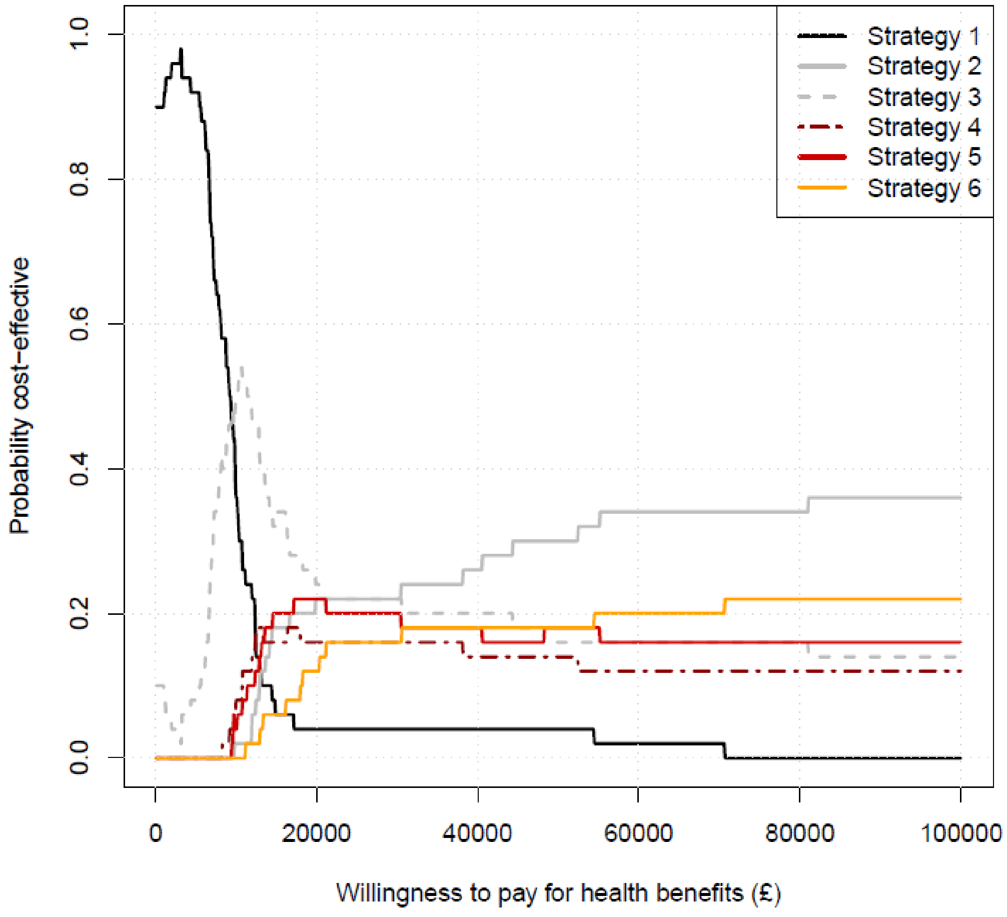

Figure A11. Cost-effectiveness acceptability frontier. Lines depict the strategies with the highest expected net monetary benefit, dependent on the willingness to pay for health benefits, while dotted vertical lines the willingness to pay values at which the decision changes.

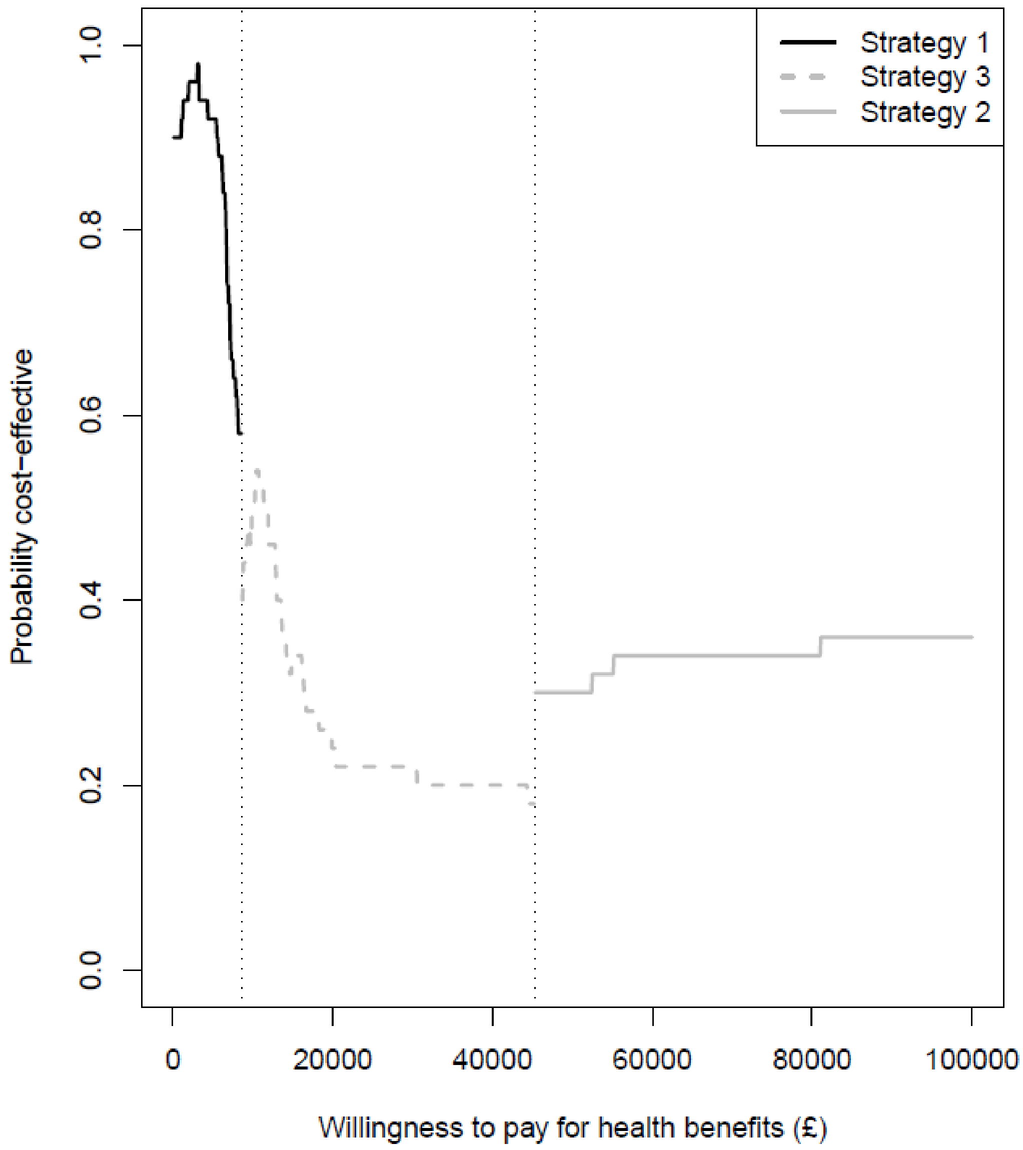

Scenario b. Low MRSA Prevalence:

In a low prevalence setting (with admission prevalence half (0.7%) that found in the audit for Acute Trusts) there are less MRSA positive isolated and unisolated bed days as there are fewer positive patients. The pattern of differences between strategies is the same as at baseline prevalence (Figure A14 in Appendix 7). The lower prevalence leads to less transmission, infections and deaths (due to less infectious imports into hospital), whilst comparison between policies remains the same as at baseline prevalence (Figure 15 Appendix 7) The costs however are very slightly higher (Figure A16 in Appendix 7), as for the same level of screening effort the screening costs are not offset to the same extent by cost savings through reductions in infections.

The cost-effectiveness plane (Figure A12) shows the ordering of strategies remains the same under a lower prevalence. Evaluation of the cost effectiveness frontier shows the ICERs change only slightly at lower prevalence, strategy 3 (screening of patients admitted to high risk specialties) remaining cost-effective (Figure A9, Table A6) at a willingness to pay threshold of £30,000/QALY. However, Strategy 5 (checklist activated screening of all admissions plus screening all admissions to high risk specialties) also becomes a cost-effective option if moving from strategy 3 to 5.

Table A6. Evaluation of cost-effectiveness frontier of strategies for an Acute Trust in a low prevalence setting, with a willingness-to-pay threshold of £30,000/QALY gained

Move between policies	Cost per admission	QALY per admission	Change in costs, ΔC (compared to previous policy)	Change in effects, ΔE (compared to previous policy)	Difference in Costs / Difference in Effects (ICER, cost/QALY)	Option evaluation
Stay at policy 1	£23,292	8.090747	-	-	-	-
Or move:						
to 3	£23,670	8.12928	£377.47	0.038533	£9,796	Cost effective
to 5	£24,090	8.147422	£420.83	0.018142	£23,196	Cost effective
to 6	£24,364	8.150547	£273.41	0.003124	£87,517	Not cost effective

The CEAC and CEAF figures (Figures A13and A14) show very similar levels of uncertainty in the decision between strategies in a lower prevalence setting as in a baseline prevalence setting with clustering around the 30% probability of cost effectiveness for several stategies at the usual NHS willingnes to pay threshold of £20-30,000. However this rose to nearly 50% for strategy 3 below a £10,000 threshold, where it was the optimal strategy. Adding checklist activated screening of other admissions to this (Strategy 5) was optimal at the upper end of the usual willingness to pay threshold (figure A14).

Figure A12. Incremental cost-effectiveness plot comparing each of the screening strategies. Numbers indicate strategy numbers. Error bars represent random error brought about by stochasticity in the model and parameter uncertainty, and correspond to plus or minus one standard error.

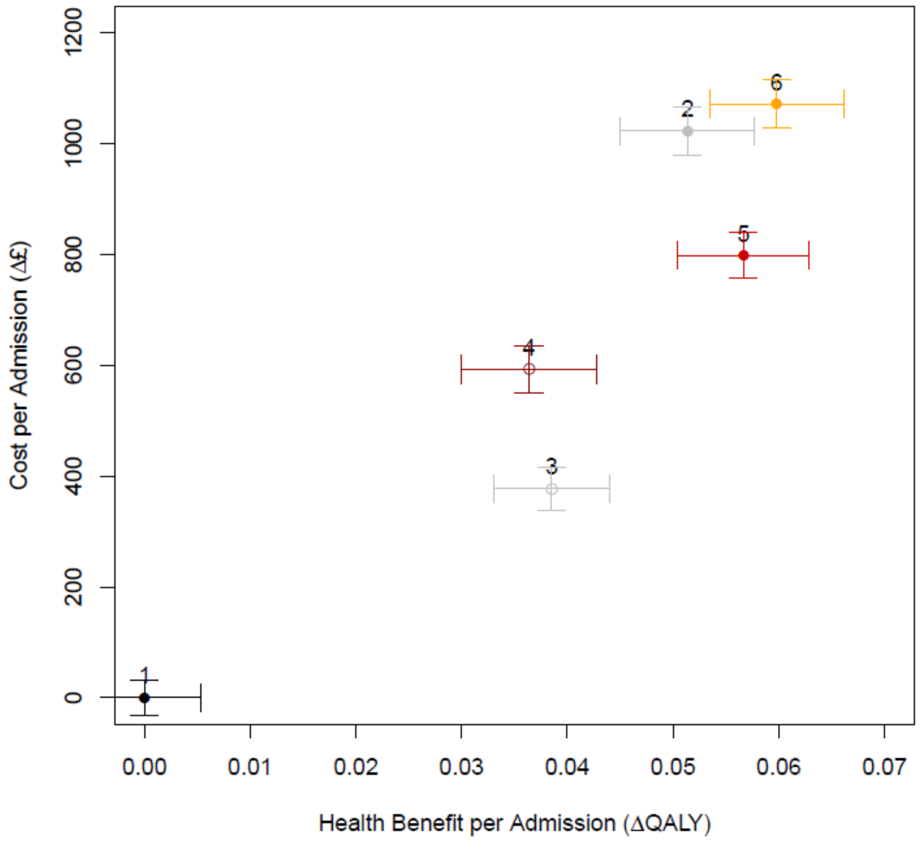

Figure A13. Cost-effectiveness acceptability curves.

Each line represents the proportion of simulations, for a particular strategy, that are cost-effective, as a function of willingness to pay for health benefits.

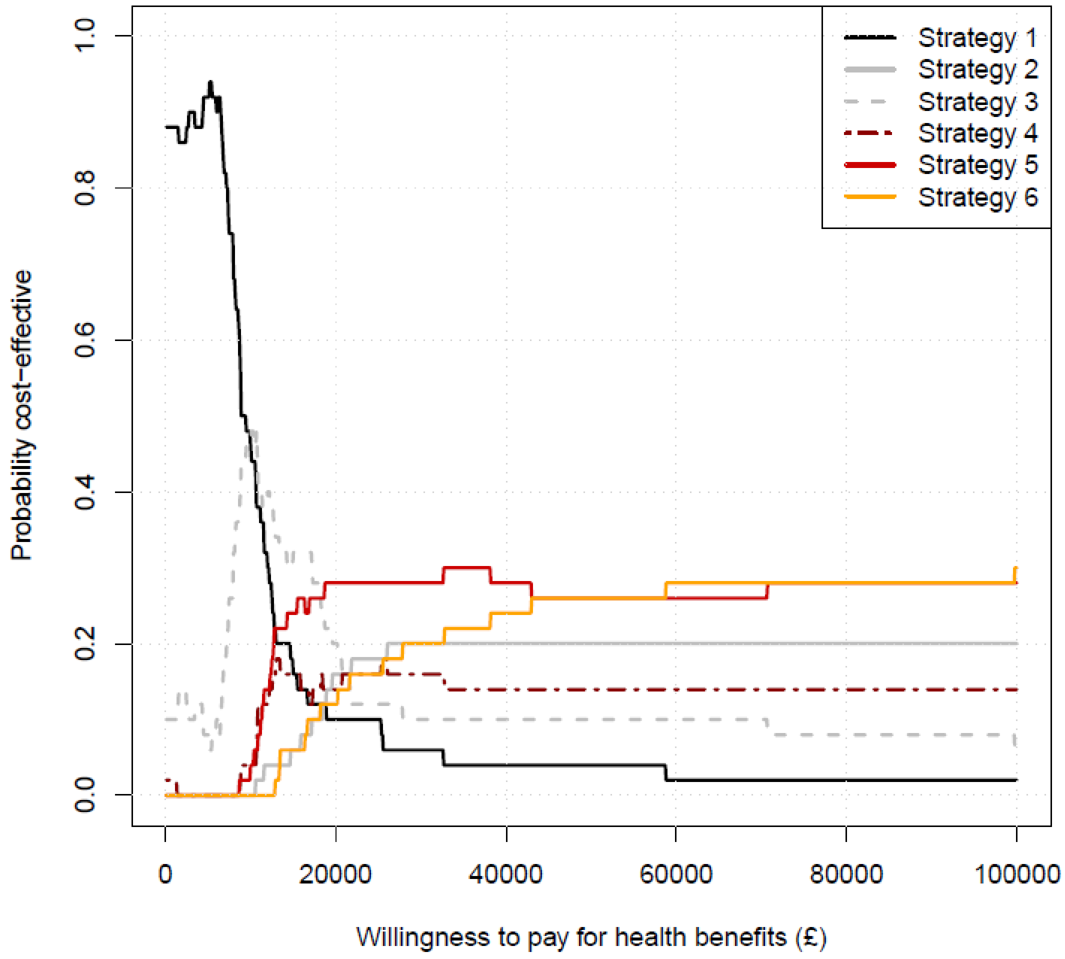

Figure A14. Cost-effectiveness acceptability frontier.

Lines depict the strategies with the highest expected net monetary benefit, dependent on the willingness to pay for health benefits, while dotted vertical lines the willingness to pay values at which the decision changes.

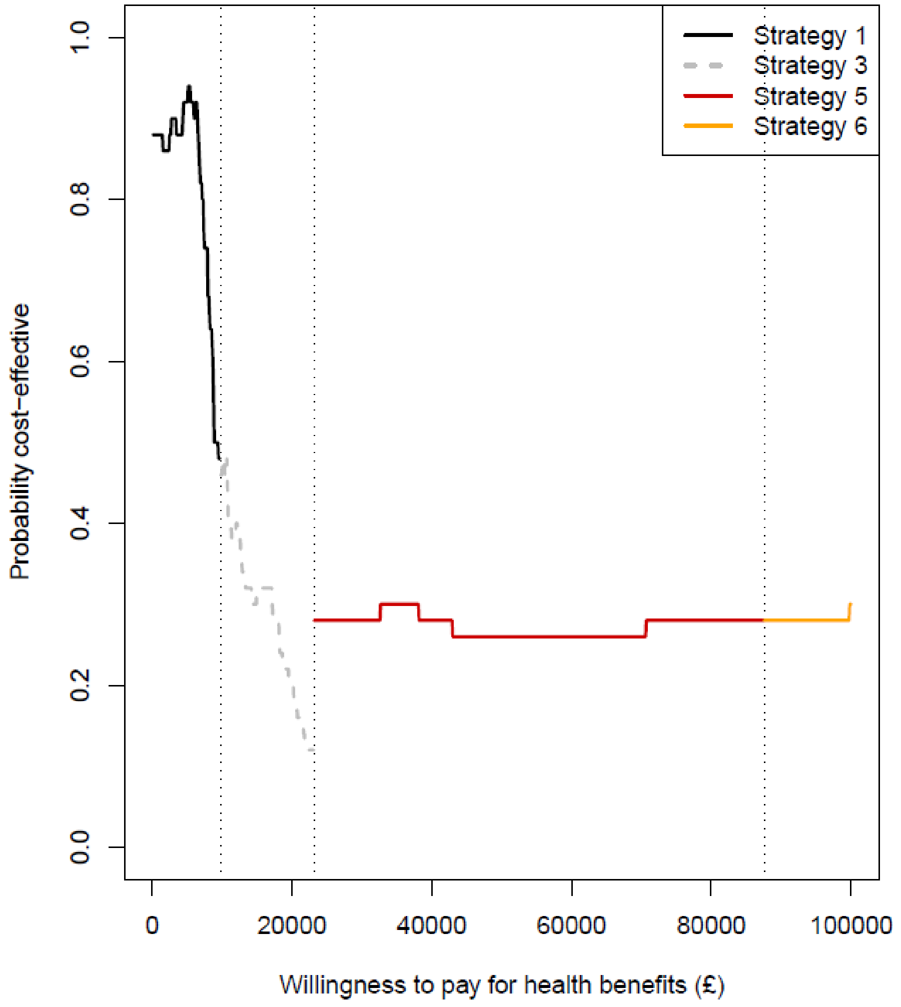

Scenario c. Low transmission in high-risk specialties:

If transmission in high risk specialties was reduced to be midway between ICU and general medical ward transmission probabilities this had very little effect on the ability of each strategy to effectively isolate MRSA positive patients but there was half the number of deaths and therefore infections. Total costs therefore were greater than under baseline transmission parameters because costs of screening were not offset to the same degree by savings through reductions in infections (Figures A 20-22 in Appendix 7)

The cost effectiveness plane (Figure A23 in Appendix 7) shows no strategy provides the same gains in health benefits at this lower transmission rate but despite a higher ICER, Strategy 3 (screening all patients admitted to high risk specialties) is still cost-effective at £12,382/QALY (Table A7).

There is much less uncertainty around this decision (Figure A15) with Strategy 3 having a probability of almost 60% of being cost effective, compared to other strategies. This is reflected in the CEAF (Figure A16) where strategy 3 is the optimal option from willingness to pay values of just over £10,000/QALY to nearly £90,000/QALY.

Table A7. Evaluation of cost-effectiveness frontier of strategies for an Acute Trust (low transmission in high risk specialties, using a willingness to pay threshold of £30,000/QALY gained.

Move between policies	Cost per admission	QALY per admission	Change in costs, ΔC (compared to previous policy)	Change in effects, ΔE (compared to previous policy)	Difference in Costs / Difference in Effects (ICER, cost/QALY)	Option evaluation
Stay at policy 1	£23,733	8.159388	-	-	-	-
Or move:						
to 3	£24,054	8.185329	£321.22	0.025942	£12,382	Cost effective
to 6	£24,712	8.193005	£657.88	0.007675	£85,713	Not cost effective

Figure A15. Cost-effectiveness acceptability curves. Each line represents the proportion of simulations, for a particular strategy, that are cost-effective, as a function of willingness to pay for health benefits.

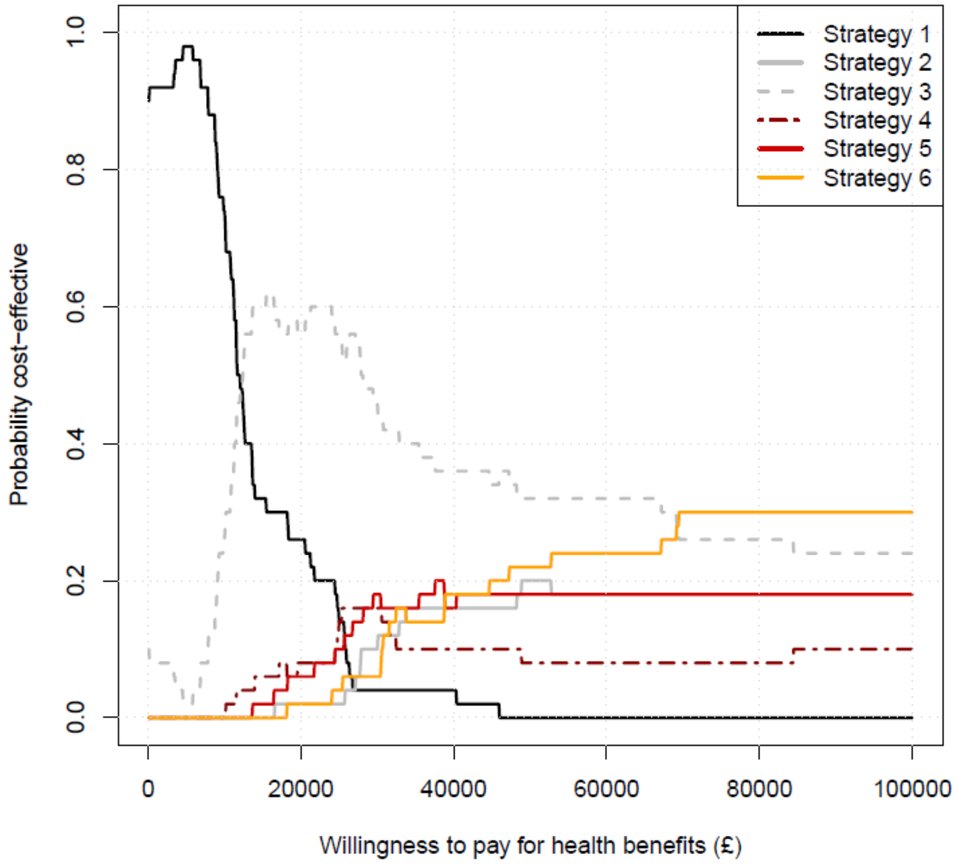

Figure A16. Cost-effectiveness acceptability frontier.

Lines depict the strategies with the highest expected net monetary benefit, dependent on the willingness to pay for health benefits, while dotted vertical lines the willingness to pay values at which the decision changes

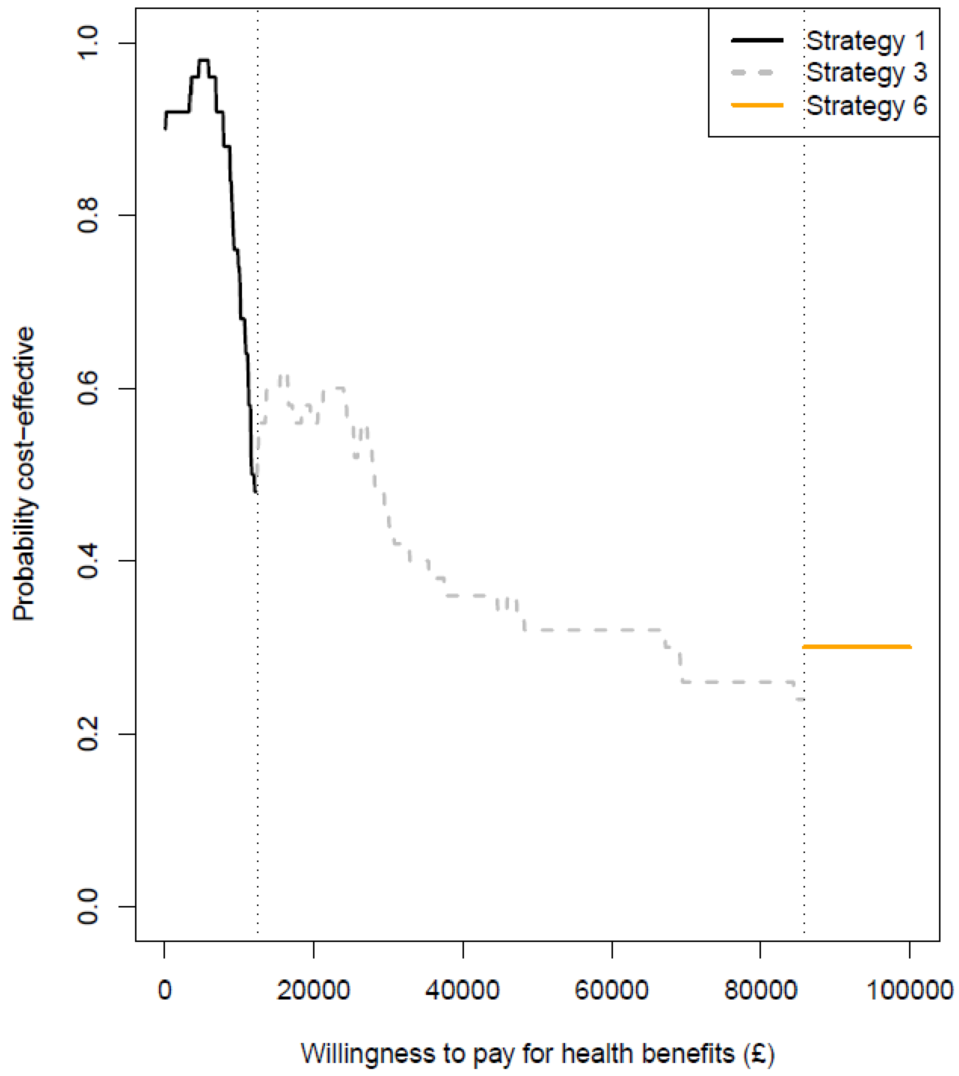

.

Scenario d. Probability of death is homogenous across the hospital*:*

In a setting where the daily probability of death in high risk specialties is reduced to that in low risk specialties, there is little change in reduction in transmission or infection events but there are differences in long term QALY accrual, as death events prevent patients being discharged and going on to accrue QALYs . Results and policies are therefore compared only in terms of costs per QALY gained.

In this scenario each screening strategy has reduced ability to prevent deaths and thus gain health benefits (QALYs). The cost-effectiveness plane shows a 10-fold reduction in health benefits gained under each strategy (Figure A17) compared to settings with the baseline probability of death. Evaluation of the cost-effectiveness frontier (Table A8) shows ICER values (cost/QALY) are much higher. Even so, Strategy 3 remains cost-effective at £26,551/QALY. The CEAC (Figure A18) shows the probability of this being cost-effective within the NHS willingness to pay threshold is about 60%. The CEAF (Figure A19), which accounts for both the magnitude of the potential benefit and its probability of cost-effectiveness, shows the decision changes dependent on willingness to pay. The “no screening” strategy is optimal, up to approximately £25,000/QALY, while strategy 3 becomes optimal above this.

Table A8 Evaluation of cost-effectiveness frontier of strategies.

Move between policies	Cost per admission	QALY per admission	Change in costs, ΔC (compared to previous policy)	Change in effects, ΔE (compared to previous policy)	Difference in Costs / Difference in Effects (ICER, cost/QALY)	Option evaluation
Stay at policy 1	£22,321	8.245049	-	-	-	-
Or move:						
to 3	£22,452	8.249984	£131.03	0.004935	£26,551	Cost effective
to 6	£22,969	8.251726	£517.07	0.001742	£296,859	Not cost effective

Figure A17. Incremental cost-effectiveness plot comparing each of the screening strategies. Numbers indicate strategy numbers as outlined above. Error bars represent random error brought about by stochasticity in the model and parameter uncertainty, and correspond to plus or minus one standard error.

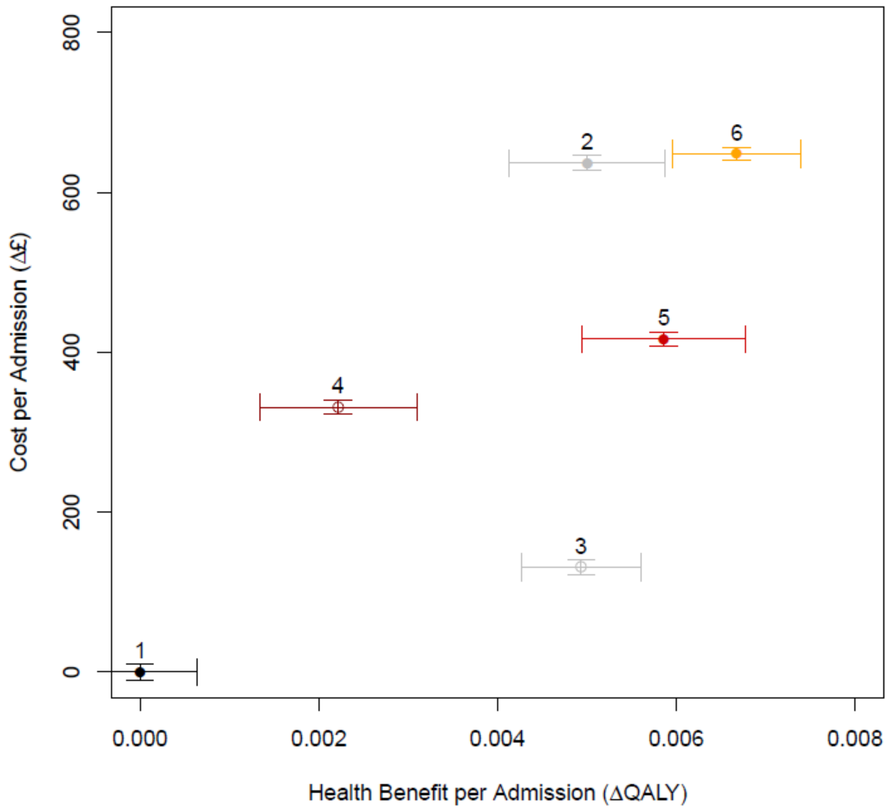

Figure A18. Cost-effectiveness acceptability curves.

Each line represents the proportion of simulations, for a particular strategy, that are cost-effective, as a function of willingness to pay for health benefits

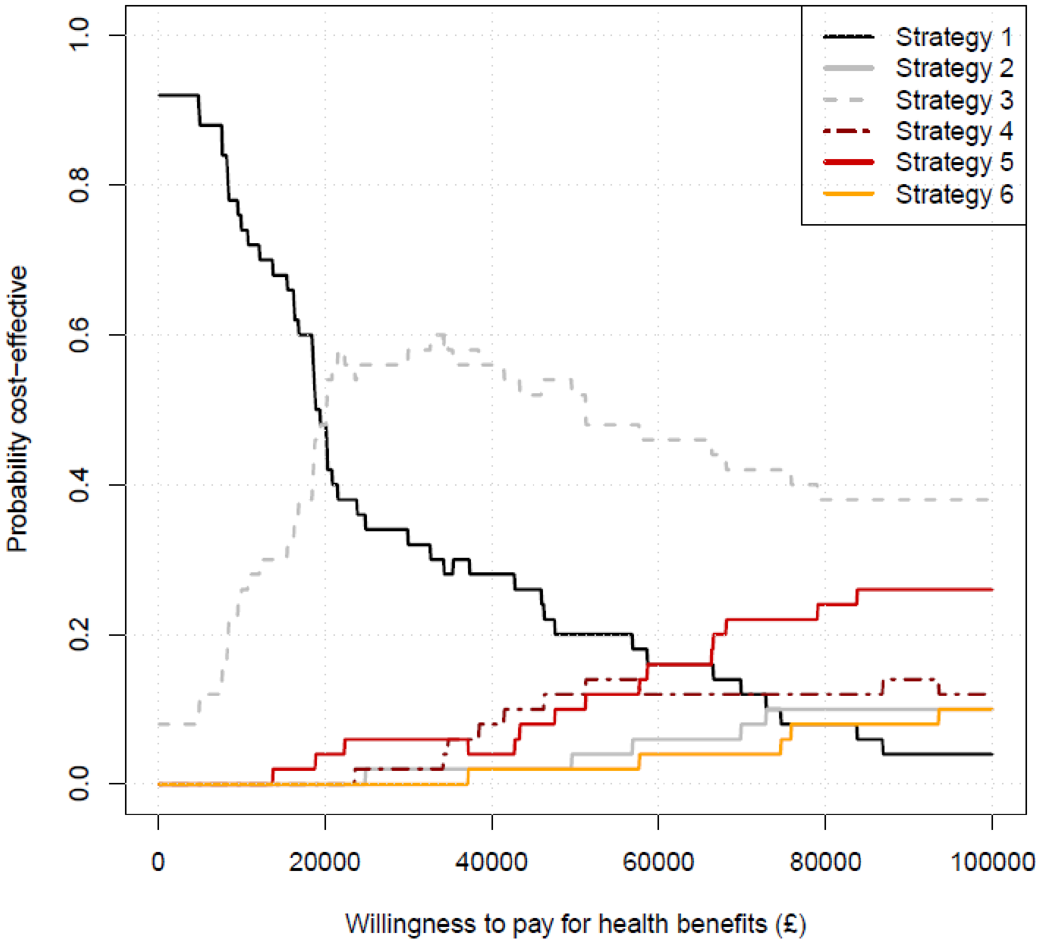

.

Figure A19. Cost-effectiveness acceptability frontier.

Lines depict the strategies with the highest expected net monetary benefit, dependent on the willingness to pay for health benefits, while dotted vertical lines the willingness to pay values at which the decision changes

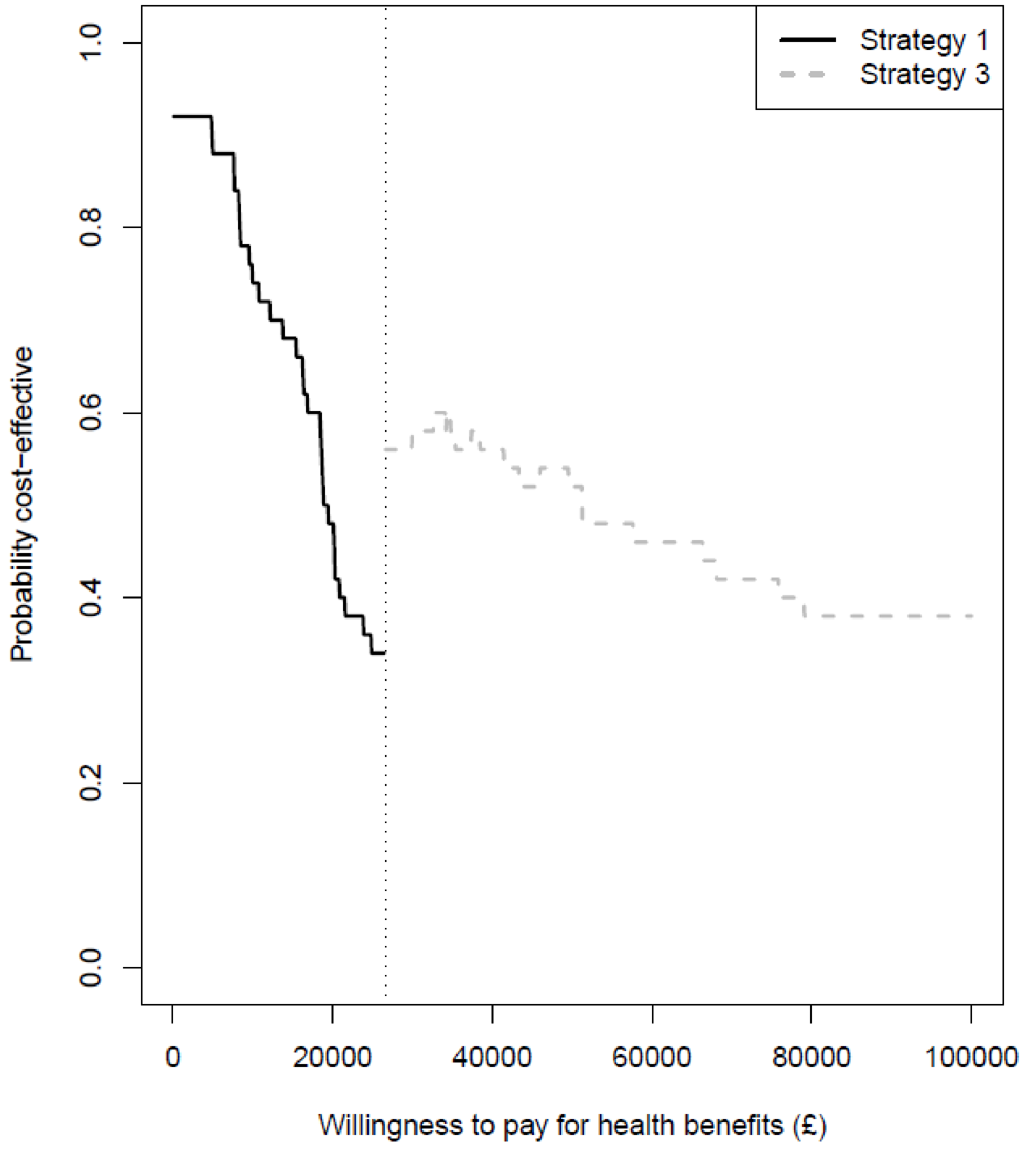

.

Scenario e. Checklist activated screening using Scottish Pathfinder checklist*:*

The Scottish Pathfinder study (Stewart et al 2011) had found that both checklist activated strategies (Strategies 4 and 5) were cost effective but used a slightly different checklist of risk factors for MRSA carriage from that used in the NOW study. Strategies 4 and 5 were therefore re-evaluated for their cost effectiveness in acute trusts at current admission prevalence levels (1.4%) using the Pathfinder checklist. This increased the prevalence of MRSA in the checklist positive group to 5.6% (from 2.6% using the NOW checklist), and decreasing the percentage of admissions screened (from 44% to 10.5% for elective patients, and from 57% of 25% of emergency admissions).

The incremental cost-effectiveness plot (Figure A29 in Appendix 7) shows, as expected, that the incremental costs for Strategies 4 (in particular) and 5 were markedly reduced because of the reduction in numbers screened. However, gains in health benefits were also reduced as more MRSA positive patients were "missed" using the Pathfinder definition because of the reduction in numbers screened. The overall incremental cost-effectiveness evaluations of the cost-effectiveness frontier (Table A9) were therefore very similar to those found using the NOW checklist. Strategy 3, screening admissions to high risk specialties, remained the only cost-effective strategy at £9,731 /QALY gained.

Table A9. Evaluation of cost-effectiveness frontier of strategies.

Move between policies	Cost per admission	QALY per admission	Change in costs, ΔC (compared to previous policy)	Change in effects, ΔE (compared to previous policy)	Difference in Costs / Difference in Effects (ICER, cost/QALY)	Option evaluation
Stay at policy 1	£22,859	7.99248	-	-	-	-
Or move:						
to 3	£23,197	8.02719	£337.77	0.034711	£9,731	Cost effective
to 5	£23,405	8.033475	£208.70	0.006285	£33,206	Not cost effective
to 6	£23,946	8.049186	£541.02	0.015711	£34,437	Not cost effective

The levels of uncertainty (Figures A30 and A31 in Appendix 7) were similar to those achieved when using the NOW checklist (Figures A6 and A7) as at willingness to pay values between £20,000 to £30,000 the probabilities that any one strategy was the most cost-effective option clustered together and did not exceed 30%.

TEACHING TRUSTS

1. Baseline analyses at current admission prevalences

Effectiveness: In teaching trusts, admission prevalence and transmission parameters are slightly lower than in acute trusts which results in fewer isolated and unisolated bed days (Figure B1 in Appendix 7), with less infections and deaths (Figure B1), although the relative differences between screening strategies are the same.

Costs: Two effects lead to teaching trusts having greater costs per admission for each screening strategy (Figure B3 in Appendix 7). These are their larger size (median of 1113 beds compared to 553) leading to greater numbers of bed days over the simulation period and the larger number of patients to be screened (acute trusts have 22% less admissions with 50% less overall bed day costs over the five-year simulation period) . Teaching hospitals also have 44% more high-risk specialty beds, which alters movement parameters within hospital and between hospital and community.

Cost-effectiveness: The incremental cost-effectiveness plot (Figure B2), shows that each strategy generates fewer health benefits (compared to baseline 'no screening') than are achieved in Acute settings due to the lower transmission, infections and deaths. Strategy 3 (screening patients admitted to high risk specialties) is the most cost-effective option on the cost-effectiveness frontier (Table B1) at just over £1,000 above the NHS willingness to pay threshold of £30,000/QALY, at £31,077 /QALY

Table B1. Evaluation of cost-effectiveness frontier of strategies.

Move between policies	Cost per admission	QALY per admission	Change in costs, ΔC (compared to previous policy)	Change in effects, ΔE (compared to previous policy)	Difference in Costs / Difference in Effects (ICER, cost/QALY)	Option evaluation
Stay at policy 1	£36,207	8.225403	-	-	-	-
Or move:						
to 3	£36,737	8.242465	£530.26	0.017063	£31,077	Not cost effective
to 5	£37,094	8.248859	£356.39	0.006394	£55,742	Not cost effective
to 6	£37,366	8.249589	£273.18	0.0073	£374,070	Not cost effective

Consideration of uncertainty: The degree of uncertainty in this decision is large (Figure B5 in Appendix 7), the CEAC (Figure B3) demonstrating that no strategy has a greater than 30% chance of cost-effectiveness within the NHS willingness to pay range of £20,000-£30,000/QALY. The CEAF (Figure B4) shows the optimal option is the isolation and decolonisation of clinical cases only (Strategy 1). This decision only changes at willingness to pay values slightly over £30,000/QALY when Strategy 3 would become cost-effective but cost-effectiveness of strategies would shift under only slightly different assumptions for transmission parameters.

For teaching trusts, restricting screening to high-risk specialty patients instead of continuing the current policy of routine screening of all admissions, would reduce total annual costs per trust by £1,864,000 (£576 per admission), at the price of 47 more colonisations (less than one extra/week/ trust) and 1.2 more infections per year. (See table B2)

Table B2 Total costs, colonisations and infections per trust/year (Teaching trusts)

	Total admissions	Total bed days	Total costs	Cost per admission	Colonisation events	Colonisation per 100 bed days	Colonisation per admission	Infections	Infection per 100 beddays	Infections per admission
Strategy 2	18532.63	2031061	6.92E+08	37339.55	400.8482	0.019736	0.021629	66.7478	0.003286	0.003602
Strategy 3	18569.3	2027306	6.83E+08	36763.91	634.81	0.031313	0.034186	72.6356	0.003583	0.003912
Difference			9320000	575.6446	-233.962	-0.01158	-0.01256	-5.8878	-0.0003	-0.00031
Per year			1864000		-46.7924			-1.17756		

Figure B1. Patient outcomes under each screening strategy,

showing new acquisitions of MRSA by hospital patients, total MRSA infections in the hospital, and total deaths (all per 100 admissions).

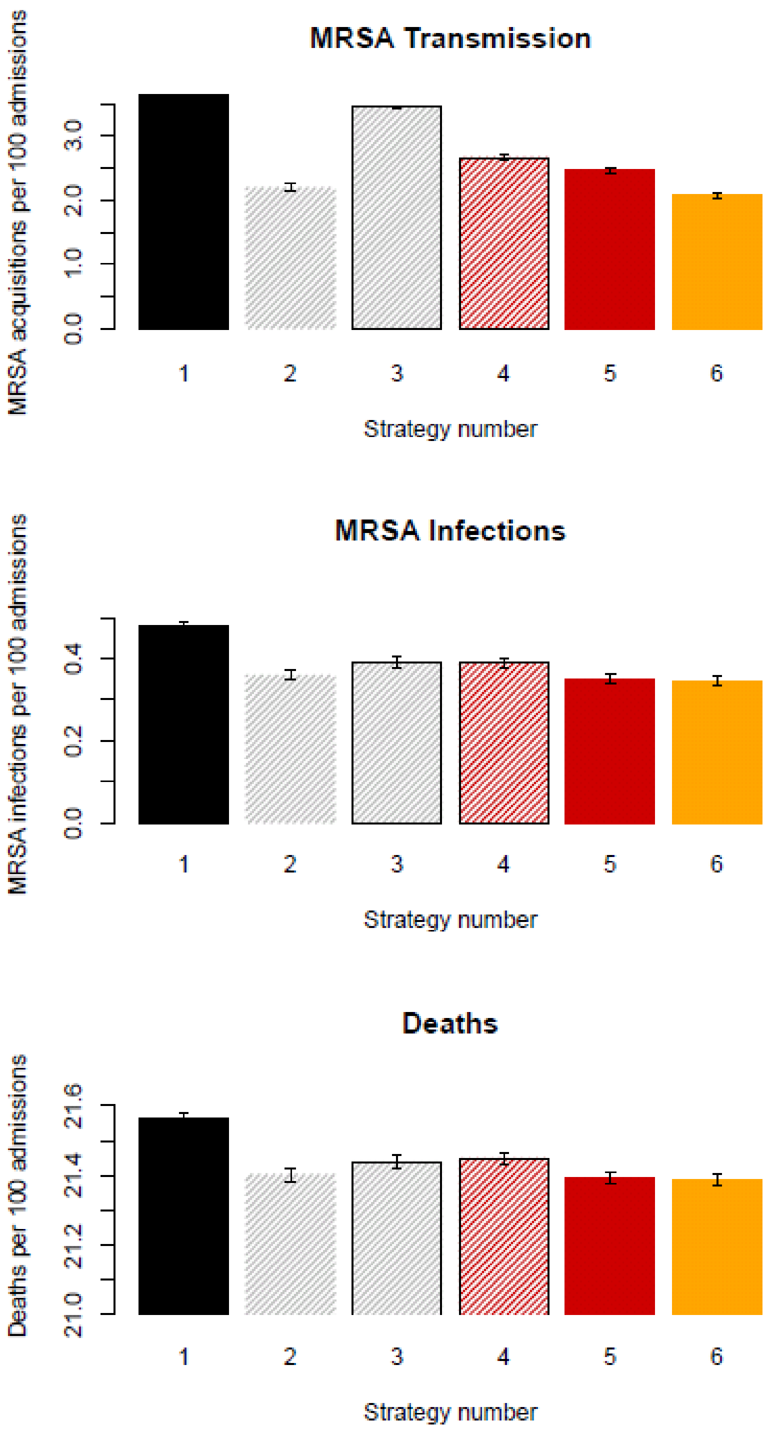

Figure B2. Incremental cost-effectiveness plot comparing each of the screening strategies. Numbers indicate strategy numbers as outlined above. Error bars represent random error brought about by stochasticity in the model and parameter uncertainty, and correspond to plus or minus one standard error.

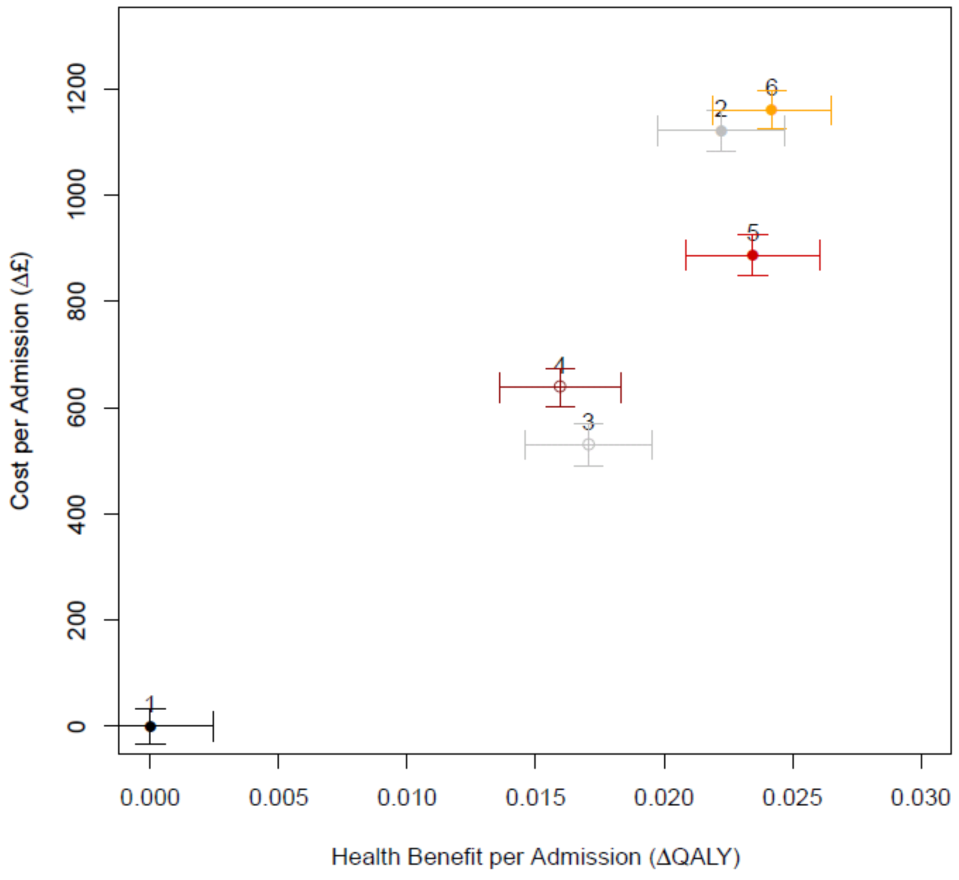

Figure B3. Cost-effectiveness acceptability curves.

Each line represents the proportion of simulations, for a particular strategy, that are cost-effective, as a function of willingness to pay for health benefits.

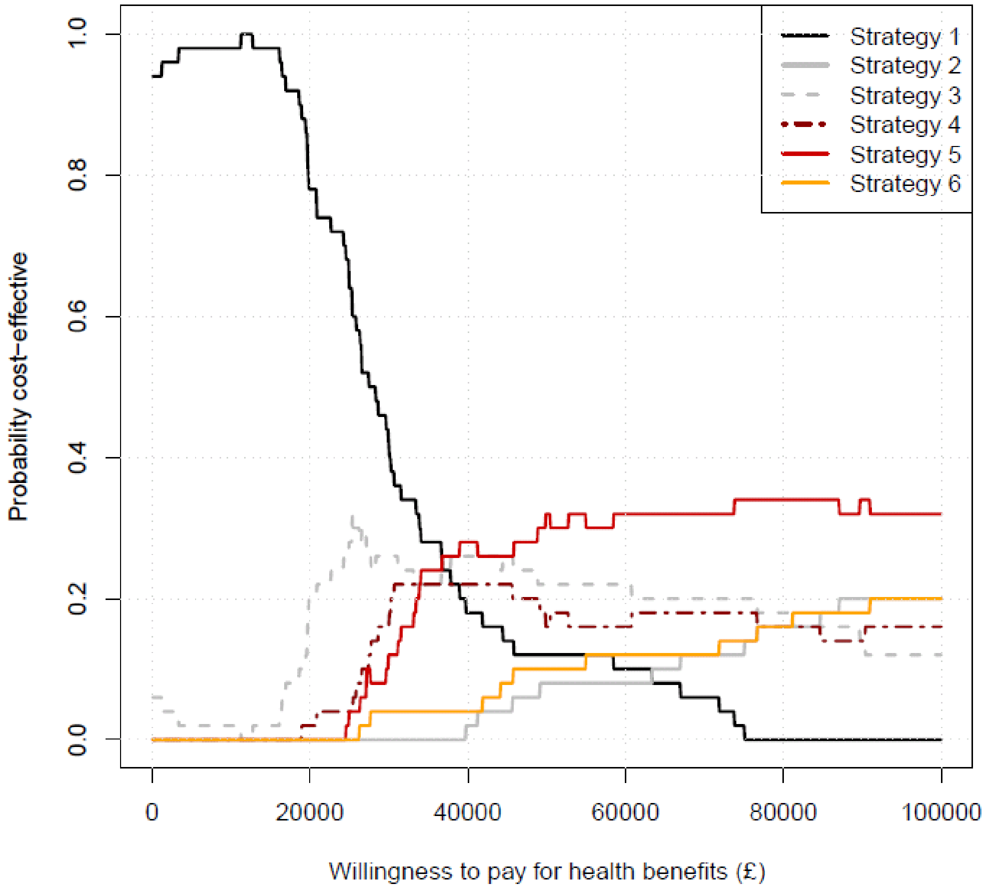

Figure B4. Cost-effectiveness acceptability frontier.

Lines depict the strategies with the highest expected net monetary benefit, dependent on the willingness to pay for health benefits, while dotted vertical lines the willingness to pay values at which the decision changes.

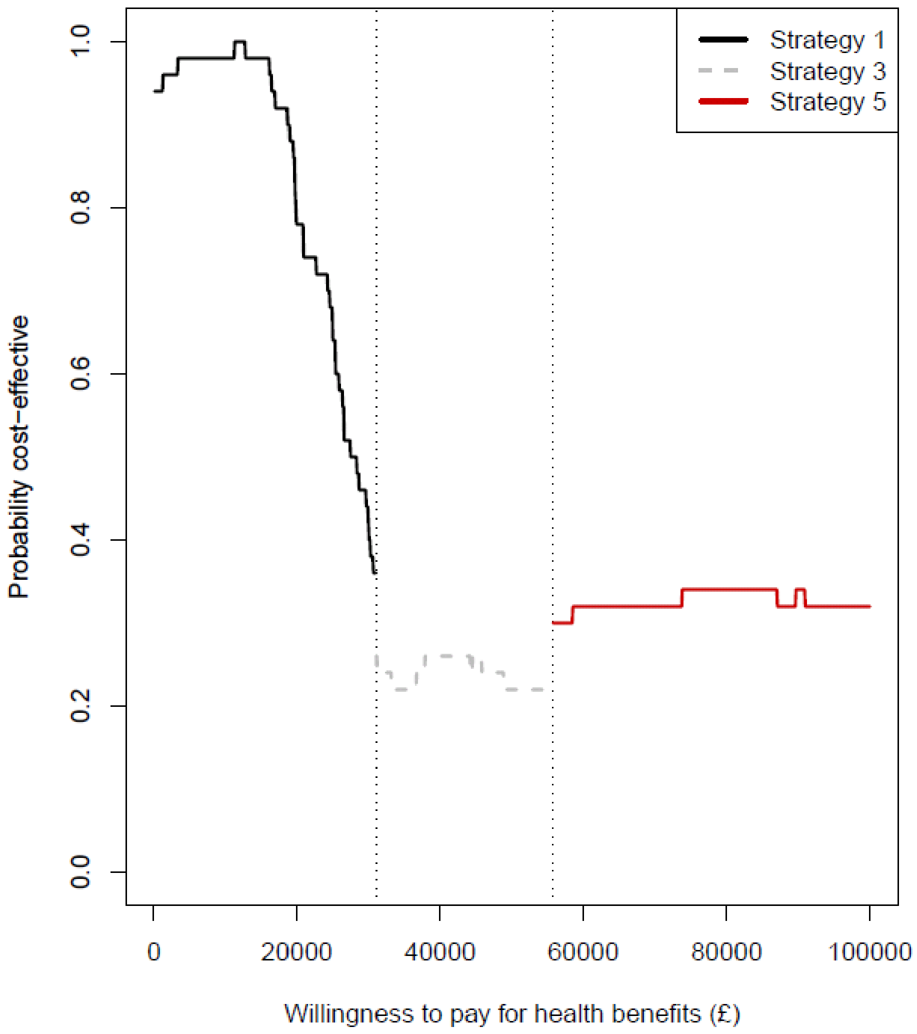

Scenario Analyses

Scenario a. High MRSA prevalence:

When prevalence is twice that of current prevalence (2.6% compared to 1.3%) both appropriate and inappropriate isolation increase (Figure B8 in Appendix 7) as do transmissions, infections and deaths (Figure B9 in Appendix 7). Total costs reduce slightly (Figure B10 in Appendix 7) as screening strategies achieve cost savings through their impact on infections leading to cost savings. The incremental cost-effectiveness plot shows each strategy provide greater gains in health benefits in this setting (Figure B5) making, screening patients admitted to high risk specialties (strategy 3), the only cost-effective strategy at £20,715/QALY (Table B3).

Table B3. Evaluation of cost-effectiveness frontier of strategies.

Move between policies	Cost per admission	QALY per admission	Change in costs, ΔC (compared to previous policy)	Change in effects, ΔE (compared to previous policy)	Difference in Costs / Difference in Effects (ICER, cost/QALY)	Option evaluation
Stay at policy 1	£35,728	8.177714	-	-	-	-
Or move:						
to 3	£36,368	8.208617	£640.15	0.030903	£20,715	Cost effective
to 5	£36,766	8.213712	£398.69	0.005095	£78,258	Not cost effective
to 6	£37,032	8.213712	£266.04	0.000861	£308,839	Not cost effective

Although both the CEAC (Figure B6) and CEAF (Figure B7) still exhibit considerable uncertainty, strategy 3 is the optimal strategy throughout the NHS willingness to pay range, with a 40% to nearly 50% probability of cost-effectiveness.

Figure B5. Incremental cost-effectiveness plot comparing each of the screening strategies. Numbers indicate strategy numbers as outlined above. Error bars represent random error brought about by stochasticity in the model and parameter uncertainty, and correspond to plus or minus one standard error.

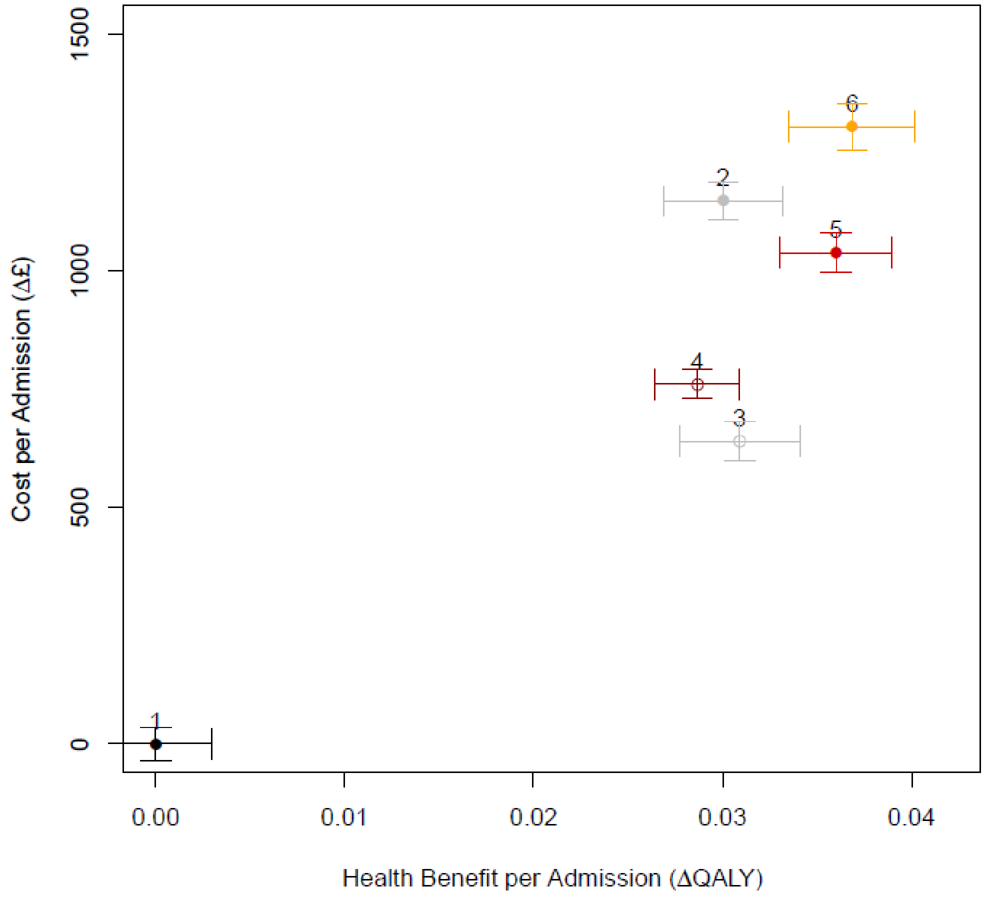

Figure B6. Cost-effectiveness acceptability curves.

Each line represents the proportion of simulations, for a particular strategy, that are cost-effective, as a function of willingness to pay for health benefits.

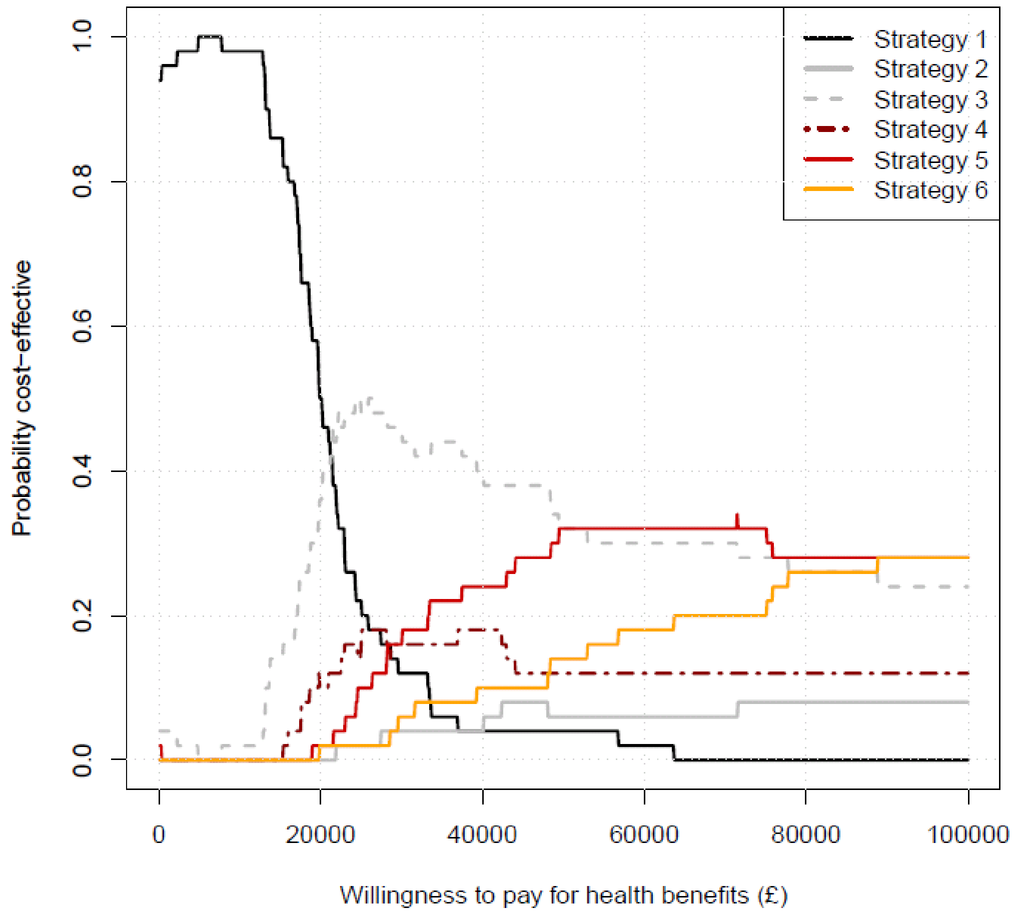

Figure B7. Cost-effectiveness acceptability frontier.

Lines depict the strategies with the highest expected net monetary benefit, dependent on the willingness to pay for health benefits, while dotted vertical lines the willingness to pay values at which the decision changes.

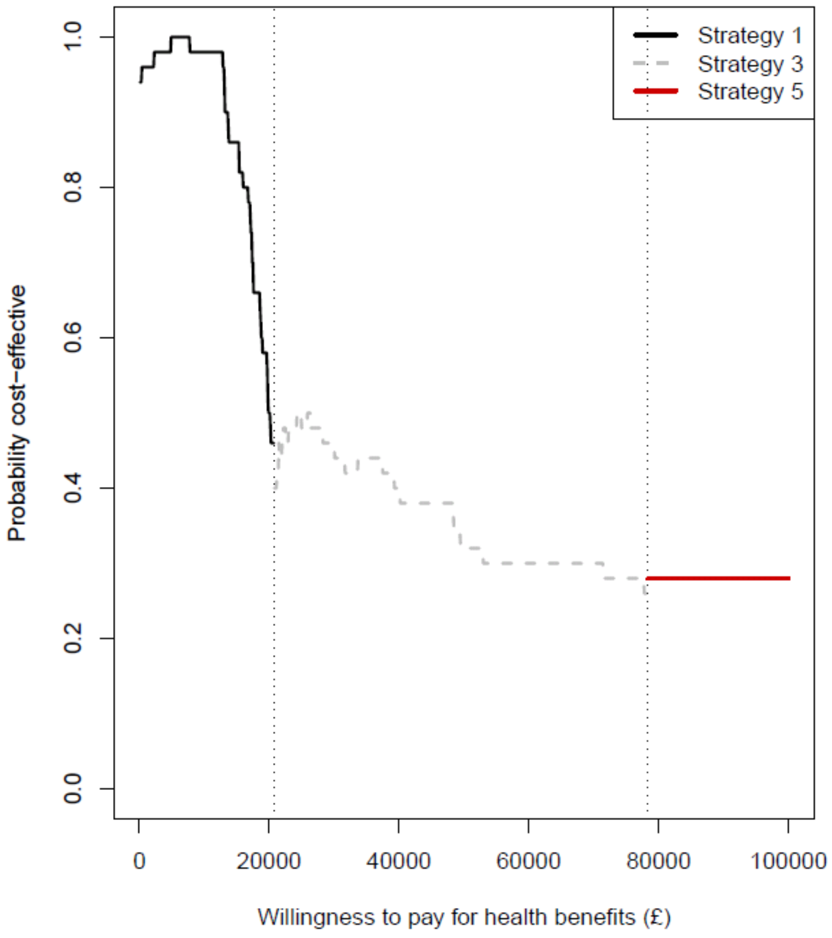

Scenario b. Low MRSA prevalence:

Where the prevalence is half that found from the audit for Teaching Trusts 0.65% compared to 1.3%) there is less use of isolation facilities (Figure B14 in Appendix 7), less transmission, infection and death (Figure B15 in Appendix 7), due to the lower infectious assault on the hospital. Costs are very slightly higher, as the same amount of screening takes place but has less impact in a setting with fewer infections (Figure B16 in Appendix 7). The incremental cost-effectiveness plot (Figure B8) shows that incremental gains in health benefits were much lower than in an average prevalence setting especially for strategy 2 (screening all admissions). The cost-effectiveness frontier showed that all strategies had ICERs well above the usual NHS willingness to pay threshold of £30,000/QALY and were therefore not cost-effective (Table B4). The CEAC (Figure B9) and CEAF (Figure B10) show that even up to a willingness to pay of £40,000/QALY no screening strategies is cost-effective. The baseline Strategy 1 (no admission screening and isolating only clinical cases) is in fact the optimal strategy, with a probability of being cost effective of 60-80% at the conventional willingness to pay threshold.

Table B4. Evaluation of cost-effectiveness frontier of strategies.

Move between policies	Cost per admission	QALY per admission	Change in costs, ΔC (compared to previous policy)	Change in effects, ΔE (compared to previous policy)	Difference in Costs / Difference in Effects (ICER, cost/QALY)	Option evaluation
Stay at policy 1	£36,627	8.261082	-	-	-	-
Or move:						
to 3	£37,052	8.267965	£424.79	0.006884	£61,710	Not cost effective
to 5	£37,371	8.271209	£319.92	0.003243	£98,634	Not cost effective
to 6	£37,655	8.273961	£282.89	0.002752	£102,797	Not cost effective

Figure B8. Incremental cost-effectiveness plot comparing each of the screening strategies. Numbers indicate strategy numbers as outlined above. Error bars represent random error brought about by stochasticity in the model and parameter uncertainty, and correspond to plus or minus one standard error

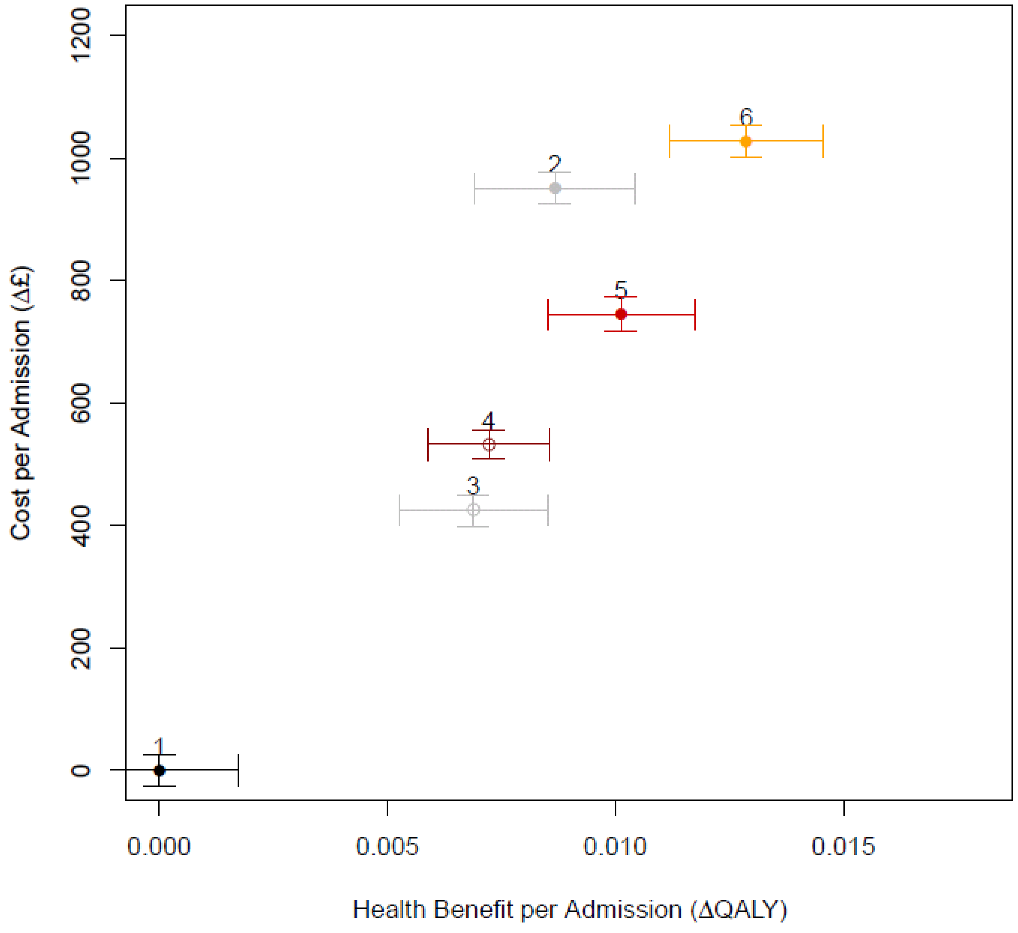

Figure B9. Cost-effectiveness acceptability curves.

Each line represents the proportion of simulations, for a particular strategy, that are cost-effective, as a function of willingness to pay for health benefits.

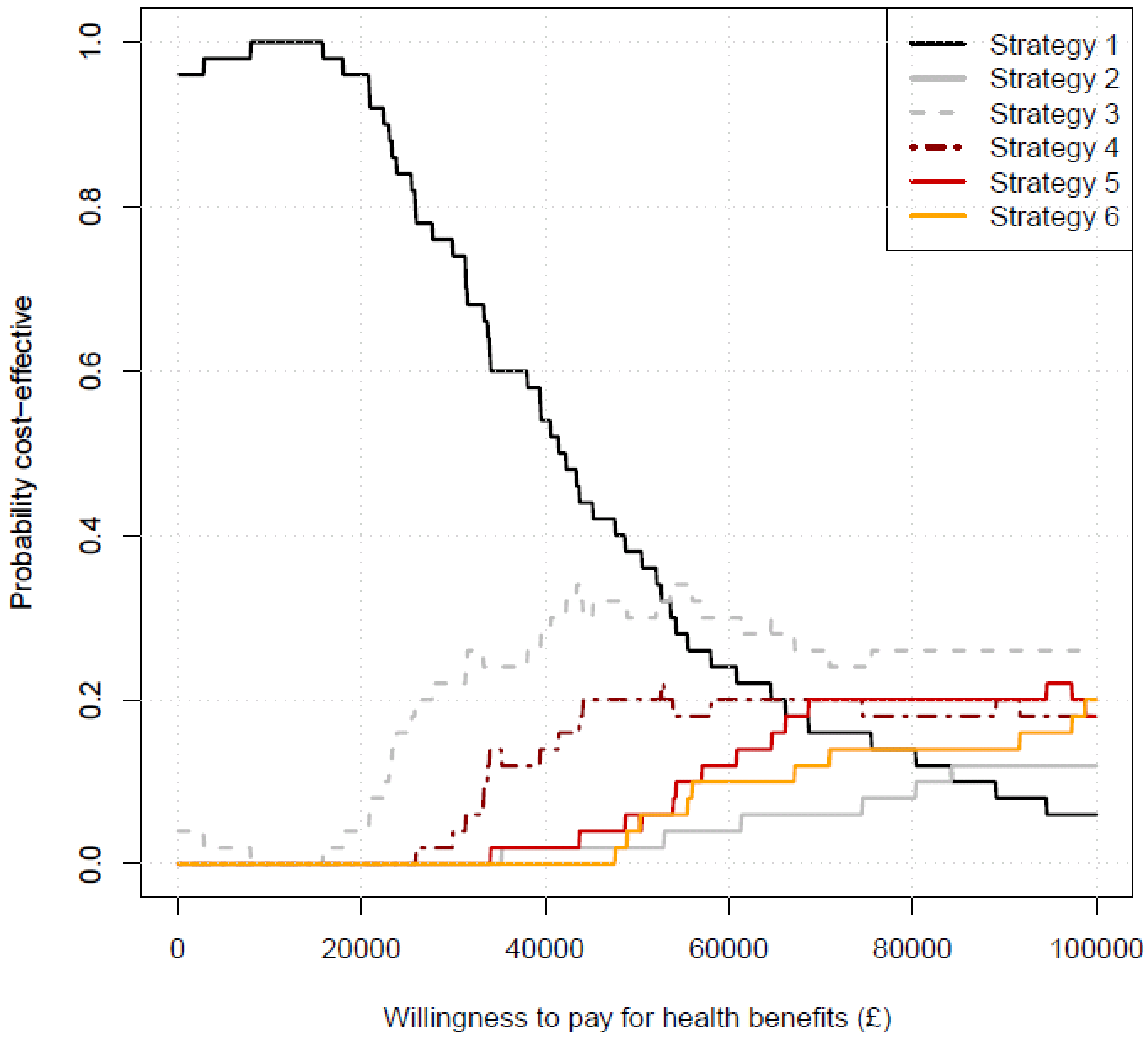

Figure B10. Cost-effectiveness acceptability frontier.

Lines depict the strategies with the highest expected net monetary benefit, dependent on the willingness to pay for health benefits, while dotted vertical lines the willingness to pay values at which the decision changes.

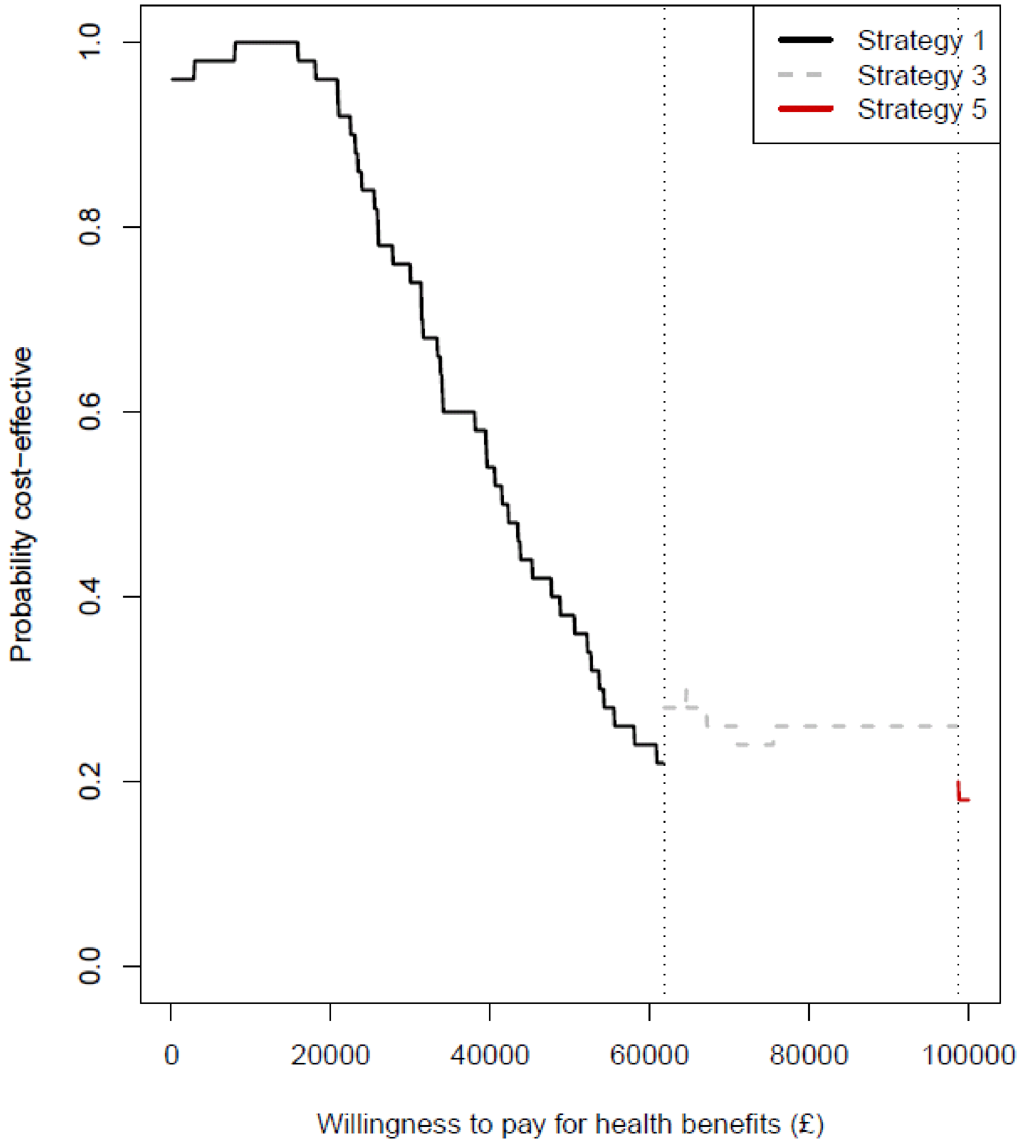

SPECIALIST TRUSTS

Baseline analyses at current admission prevalence (1.04%)

Effectiveness:

Key characteristics of specialist trusts which impact on the effectiveness and costs of screening strategies are their smaller size (72% smaller than acute trusts), which is not proportionally matched by the reduction in numbers of admissions (specialist trusts have 59% fewer admissions), and their higher proportions of high risk specialty beds (44% compared to 16%), which affects patient movement parameters and gives the population higher probabilities of transmission, infection and progression from colonisation to infection.

All strategies isolate greater numbers of patients per 100 bed days in a Specialist setting (Figure C1 in Appendix 7) , with greater appropriate and less inappropriate isolation because of the increased isolation of clinical cases owing to the higher numbers of infections arising from higher transmission in this setting. This results in higher numbers of unisolated MRSA positive cases, as those missed by individual strategies go on to generate more cases.

While the ordering of strategies remains the same as in Acute Trusts in terms of effectiveness at reducing transmission, there are more infections and a 22% increase in deaths per 100 admissions than in Acute trusts (Figure C1) despite the slightly lower admission prevalence. Costs are less than in acute settings due principally to the lower bed costs in the smaller specialist trusts (figure C3 in Appendix 7) .

Cost-effectiveness

Comparing incremental costs (Figure C2) shows that while costs remain broadly similar to an Acute setting, the ordering of strategies shifts slightly, with Strategy 3, screening admissions to high risk specialties, becoming a more costly option (compared to the baseline) due to the greater proportion of patients admitted to high risk specialties. Incremental health benefits gained for all screening strategies (compared to the baseline 'no screening' strategy, strategy 1) are at least twice as great in a Specialist setting (Figure C2), leading to ICERs less costly than the cost-effectiveness threshold of £30,000. Strategy 6, which also has the greatest gains in health benefits (screening all patients on admission plus

pre-emptive isolation of those patients known to have been previously MRSA positive) is also cost effective at £14,324/QALY.

Table C1. Evaluation of cost-effectiveness frontier of strategies.

Move between policies	Cost per admission	QALY per admission	Change in costs, ΔC (compared to previous policy)	Change in effects, ΔE (compared to previous policy)	Difference in Costs / Difference in Effects (ICER, cost/QALY)	Option evaluation
Stay at policy 1	£15,536	7.408665	-	-	-	-
Or move:						
to 3	£16,601	7.507464	£1,064.77	0.098798	£10,777	Cost effective
to 6	£17,451	7.566808	£850.08	0.059344	£14,324	Cost effective

Consideration of uncertainty

There is substantial uncertainty in costs and QALYs as represented in Figure C3, with the CEAC (Figure C4) showing very tight clustering of screening strategies at probabilities of only 20% that any one strategy is the most cost-effective at willingness to pay values of £20-30,000/QALY. This is reflected in the CEAF (Figure C5) where even though strategy 6 appears optimal for willingness to pay values over £15,000/QALY, the certainty in this decision does not exceed 25% for any willingness to pay.

For specialist trusts, moving from the current policy of screening all admissions to restricting screening to high-risk specialty patients would reduce total annual costs per trust by £438,000 (£600 per admission), at the price of 18.3 more colonisations (one extra/every three weeks/ trust) and 2.6 more infections per year. (see table C2)

Table C2. Total costs, colonisations and infections per trust/year (Specialist trusts)

	Total admissions	Total bed days	Total costs	Cost per admission	Colonisation events	Colonisation per 100 bed days	Colonisation per admission	Infections	Infection per 100 beddays	Infections per admission
Strategy 2	5710.521	277399.6	97836000	17132.59	555.5038	0.200254	0.097277	133.5398	0.04814	0.023385
Strategy 3	5785.037	277399.9	95644000	16533	647.0232	0.233246	0.111844	146.4204	0.052783	0.02531
Difference			2192000	599.5879	-91.5194	-0.03299	-0.01457	-12.8806	-0.00464	-0.00193
Per year			438400		-18.3039			-2.57612		

Figure C1. Patient outcomes under each screening strategy,

showing new acquisitions of MRSA by hospital patients, total MRSA infections in the hospital, and total deaths (all per 100 admissions).

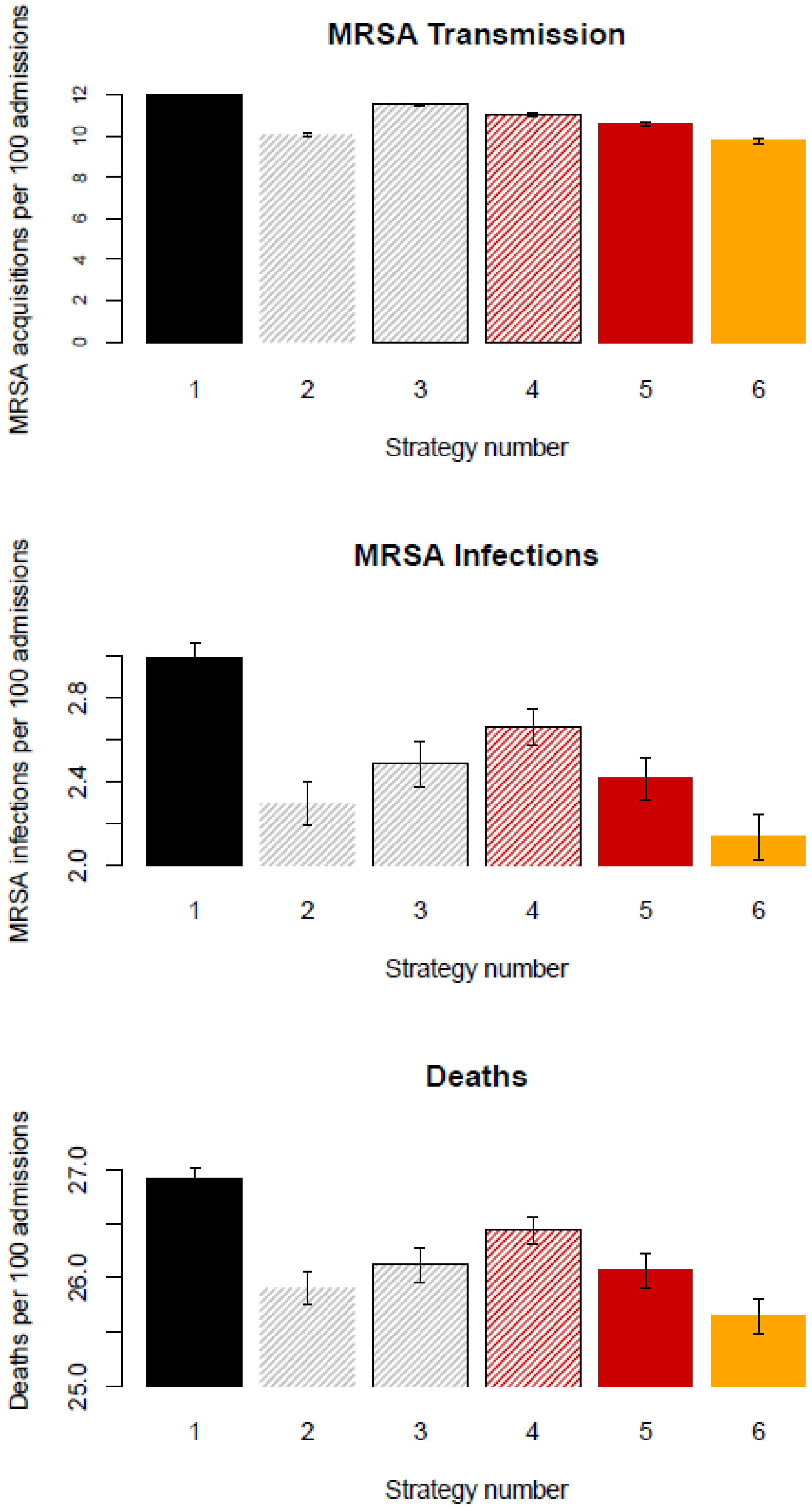

Figure C2. Incremental cost-effectiveness plot comparing each of the screening strategies. Numbers indicate strategy numbers as outlined above. Error bars represent random error brought about by stochasticity in the model and parameter uncertainty, and correspond to plus or minus one standard error.

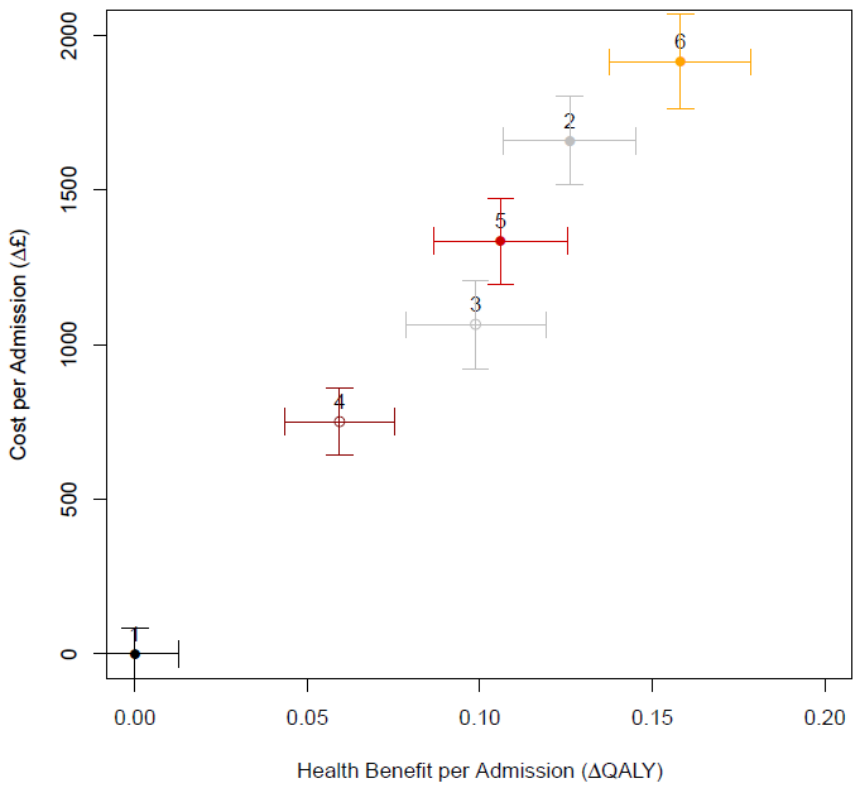

Figure C3. Simulation results comparing each strategy on a cost-effectiveness plane for a Specialist Trust setting.

Each dot represents the mean of 1000 simulation runs for each parameter set. The results of 50 parameter sets for each strategy are plotted, where each parameter set is obtained by taking the mean value for all parameters apart from the effectiveness of the intervention, which is sampled from its probability distribution.

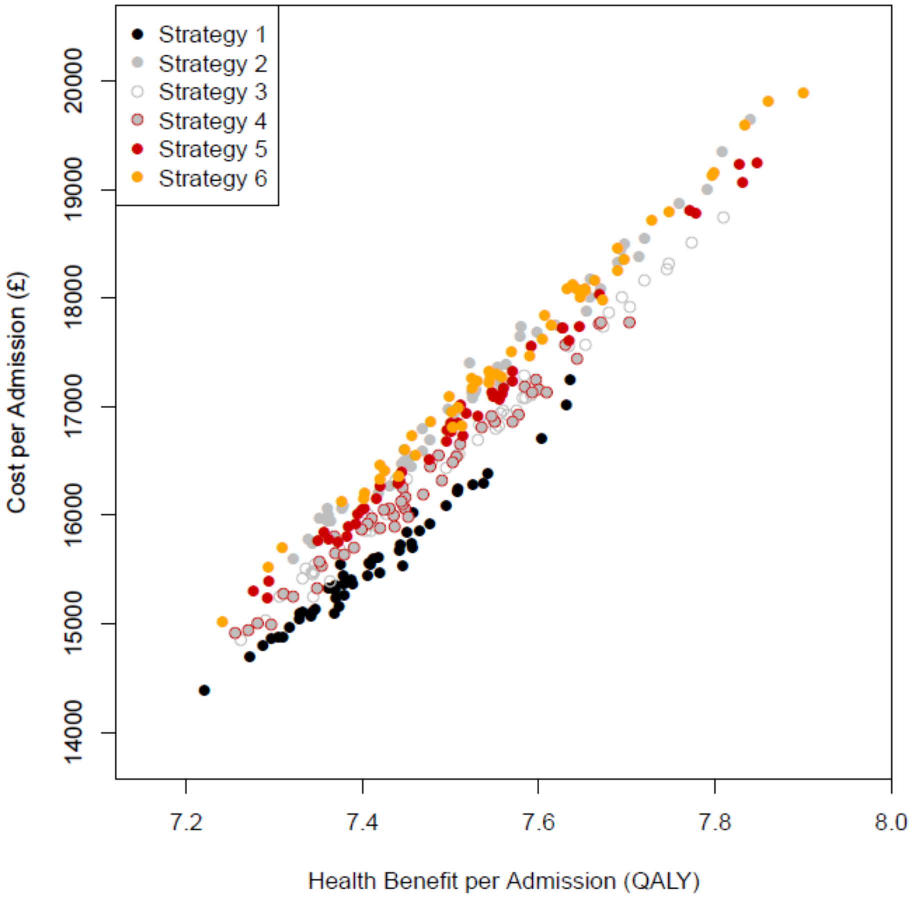

Figure C4. Cost-effectiveness acceptability curves.

Each line represents the proportion of simulations, for a particular strategy, that are cost-effective, as a function of willingness to pay for health benefits

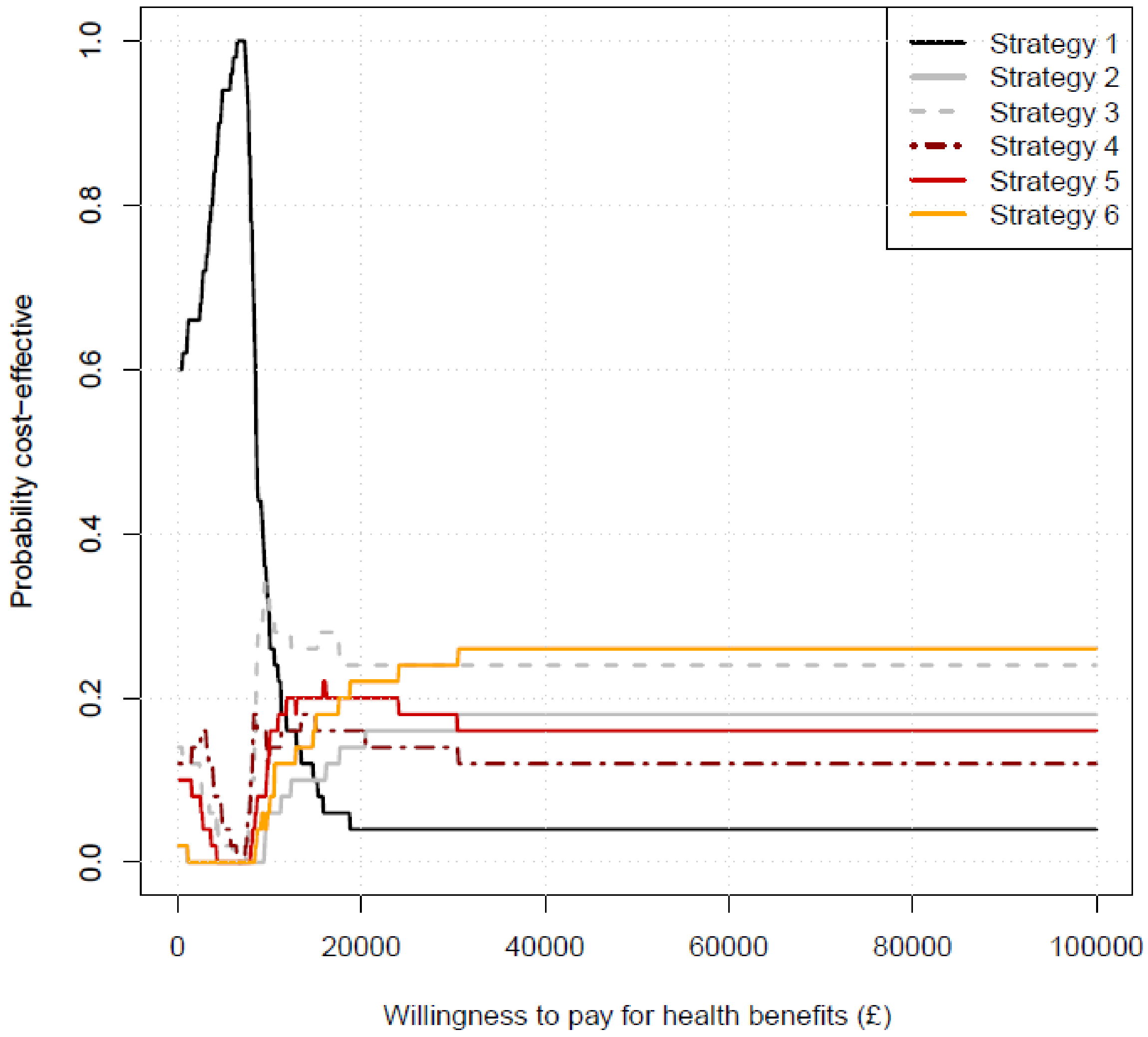

.

Figure C5. Cost-effectiveness acceptability frontier.

Lines depict the strategies with the highest expected net monetary benefit, dependent on the willingness to pay for health benefits, while dotted vertical lines the willingness to pay values at which the decision changes.

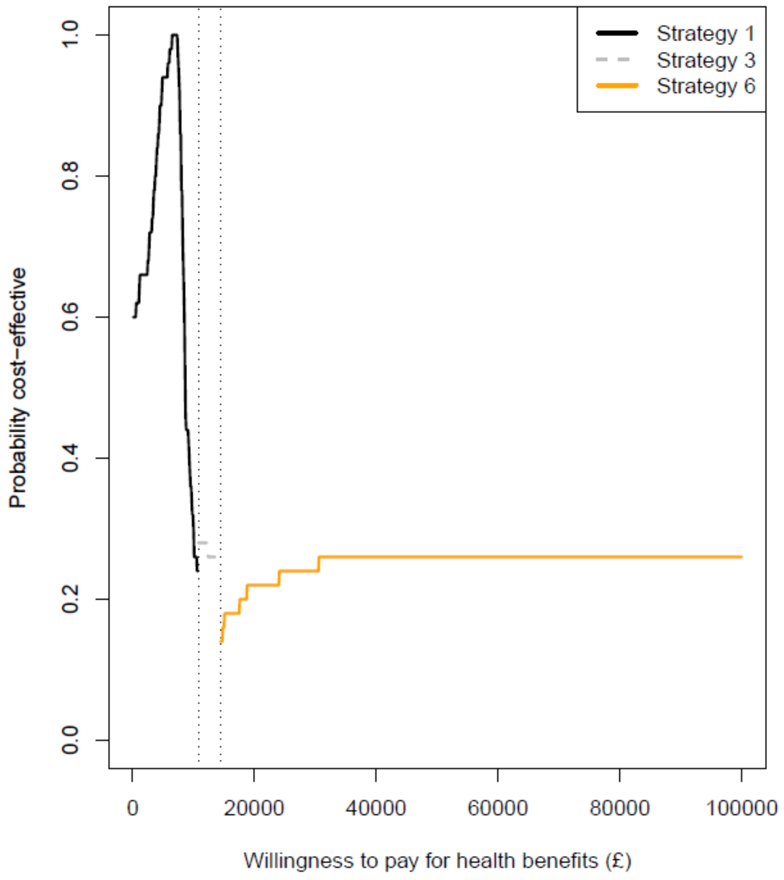

Scenario Analyses

Scenario a. High MRSA prevalence:

Effectiveness:

When prevalence is twice that of current prevalence, appropriate isolation increases and inappropriate isolation decreases, as there are more cases of MRSA (Figure C8 in Appendix 7). Unisolated MRSA positive bed days slightly increase as there are more missed cases and more onwards transmission. The relative ability of the strategies to effectively identify and isolate (and decolonise) MRSA positive patients remains the same. There are very slight increases in transmission and thus infection and deaths due to the higher admission prevalence. Total costs per admission are slightly lower (Figure C10 in Appendix 7) as all strategies prevent more infections and produce cost-savings.

Cost-effectiveness*:*

The incremental cost-effectiveness plot (Figure C6) shows the strategies are much more tightly clustered for both incremental costs and incremental health benefits, making the choice between strategies more difficult. Evaluation of the cost-effectiveness frontier (Table C3) shows only strategy 3, screening patients admitted to high risk specialties, is cost-effective at £9,745/QALY. The CEAC (Figure C7) demonstrates the uncertainty in this decision. The CEAF (Figure C8) shows this is the optimal policy, yielding the greatest monetary net benefits for all values of willingness to pay above approximately £10,000/QALY.

Table C3. Evaluation of cost-effectiveness frontier of strategies.

Move between policies	Cost per admission	QALY per admission	Change in costs, ΔC (compared to previous policy)	Change in effects, ΔE (compared to previous policy)	Difference in Costs / Difference in Effects (ICER, cost/QALY)	Option evaluation
Stay at policy 1	£14,930	7.306292	-	-	-	-
Or move:						
to 3	£16,094	7.425706	£1,163.70	0.119414	£9,745	Cost effective
to 6	£16,492	7.426992	£398.00	0.001285	£309,624	Not cost effective

Figure C6. Incremental cost-effectiveness plot comparing each of the screening strategies. Numbers indicate strategy numbers as outlined above. Error bars represent random error brought about by stochasticity in the model and parameter uncertainty, and correspond to plus or minus one standard error.

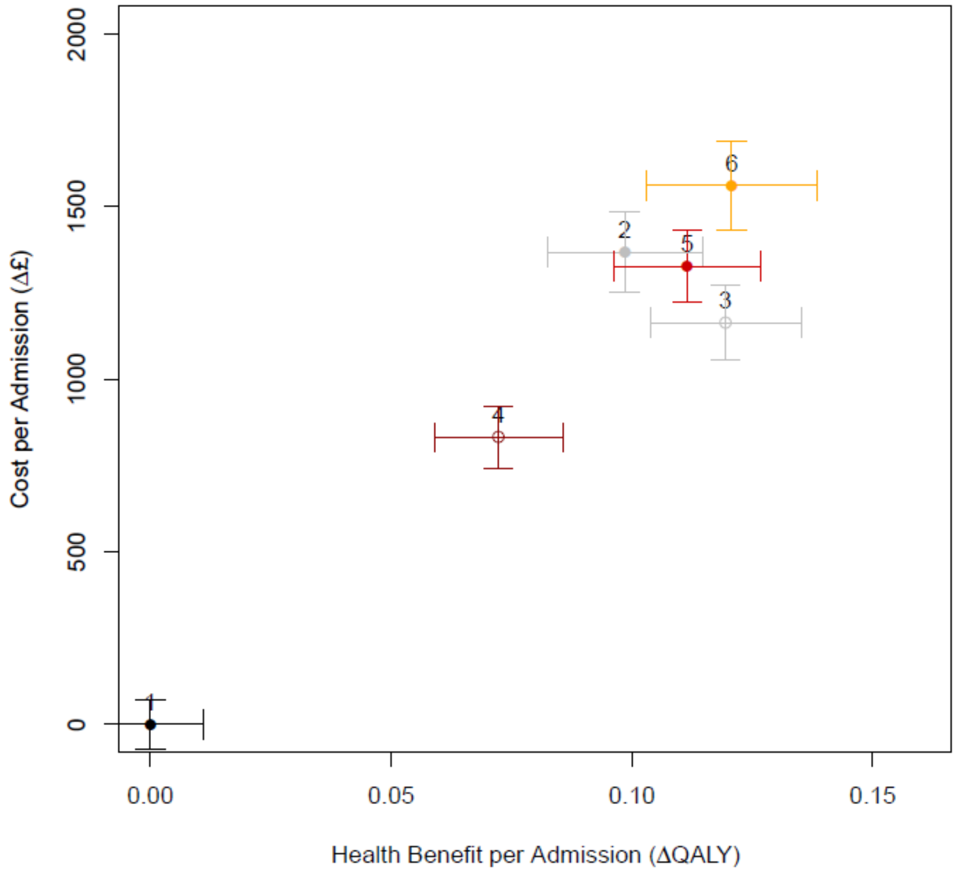

Figure C7. Cost-effectiveness acceptability curves.

Each line represents the proportion of simulations, for a particular strategy, that are cost-effective, as a function of willingness to pay for health benefits

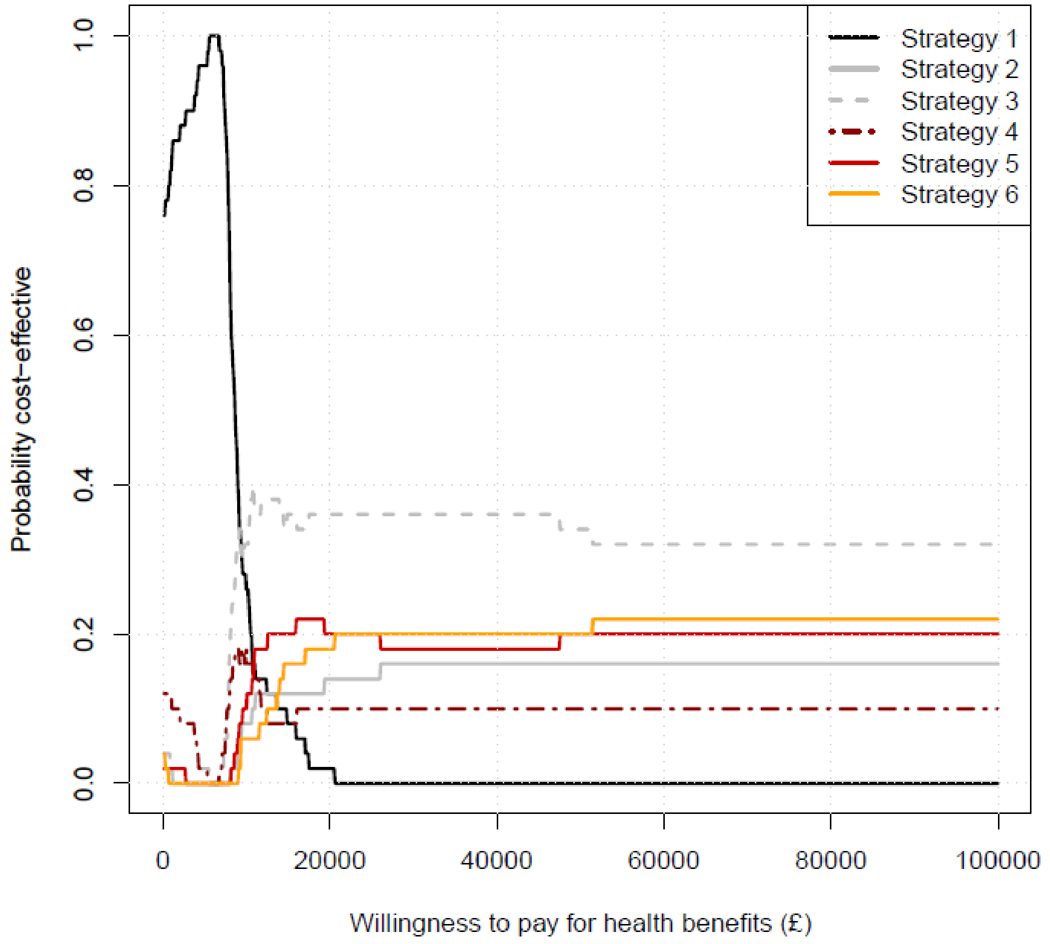

.

Figure C8. Cost-effectiveness acceptability frontier.

Lines depict the strategies with the highest expected net monetary benefit, dependent on the willingness to pay for health benefits, while dotted vertical lines the willingness to pay values at which the decision changes.

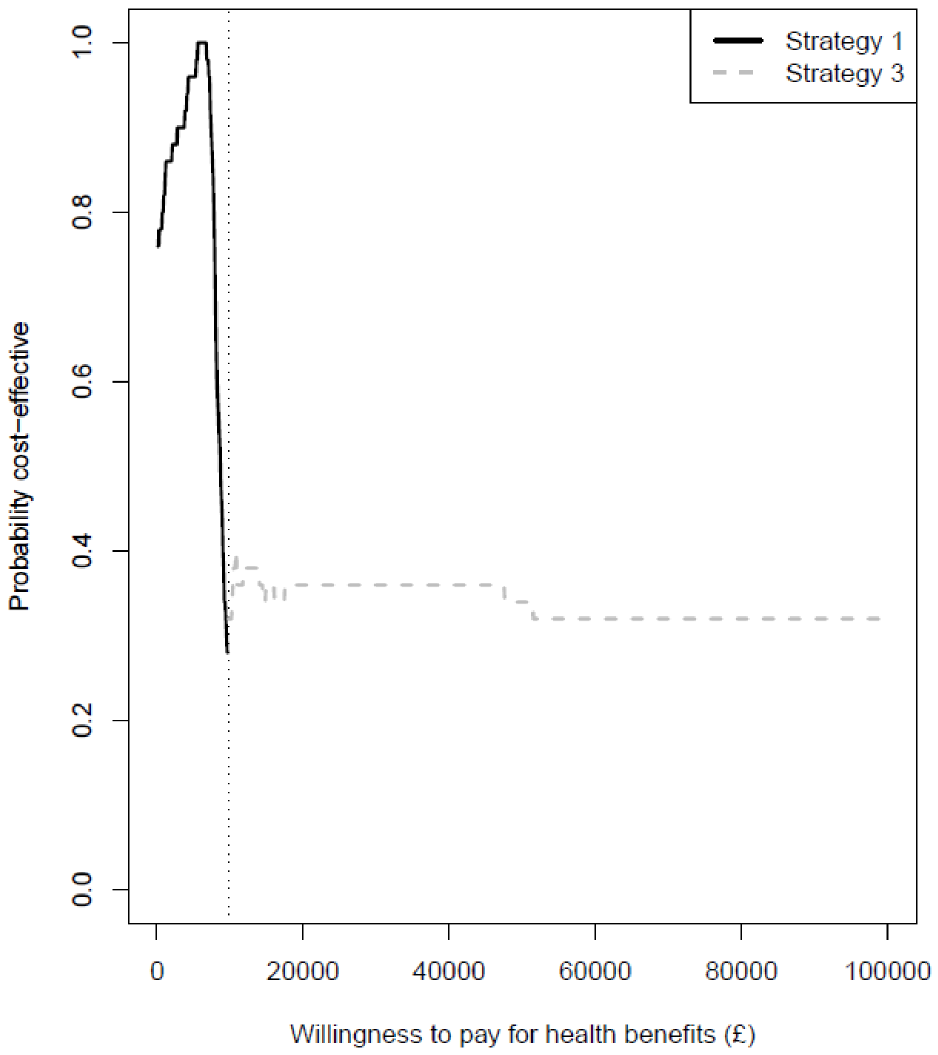

Scenario b. Low MRSA prevalence (0.52%)

In a low prevalence setting, half that of current admission prevalence, appropriate isolation and unisolated MRSA positive bed days reduce, whilst inappropriate isolation increases marginally (Figure C14 in Appendix 7). Transmissions, infections and deaths are less (Figure C15 in Appendix 7), whilst the relative ordering of strategies to reduce these remains the same. The lower prevalence leads to higher costs as the screening effort remains the same, but identifies fewer MRSA positive patients and has reduced ability to prevent infections and provide cost-savings (Figure C16 in Appendix 7). The incremental cost-effectiveness plot values (Figure C9) are similar to those in an average prevalence setting, although there is a slight re-ordering of the screening strategies. Evaluation of the cost-effectiveness frontier (Table C4). shows that strategy 3, screening of high risk specialty admissions, remains cost-effective at £10,566/QALY but that Strategy 2, screening of all patients, also becomes cost-effective at £14,224/QALY.

Table C4. Evaluation of cost-effectiveness frontier of strategies.

Move between policies	Cost per admission	QALY per admission	Change in costs, ΔC (compared to previous policy)	Change in effects, ΔE (compared to previous policy)	Difference in Costs / Difference in Effects (ICER, cost/QALY)	Option evaluation
Stay at policy 1	£16,205	7.51243	-	-	-	-
Or move:						
to 3	£17,457	7.630957	£1,252.36	0.118527	£10,566	Cost effective
to 2	£18,304	7.690535	£847.43	0.059578	£14,224	Cost effective

There is substantial uncertainty however, with the CEAC (Figure C10) showing strategies 2, 3, 5 or 6 had a 25% probability of being the most cost-effective within the usual NHS willingness to pay range (£20,000-£30,000/QALY). The CEAF (figure C11), shows Strategy 2 is optimal for all willingness to pay values above £15,000/QALY and Strategy 3 below that, but with probabilities of cost-effectiveness no more than 30%.

Figure C9. Incremental cost-effectiveness plot comparing each of the screening strategies. Numbers indicate strategy numbers as outlined above. Error bars represent random error brought about by stochasticity in the model and parameter uncertainty, and correspond to plus or minus one standard error.

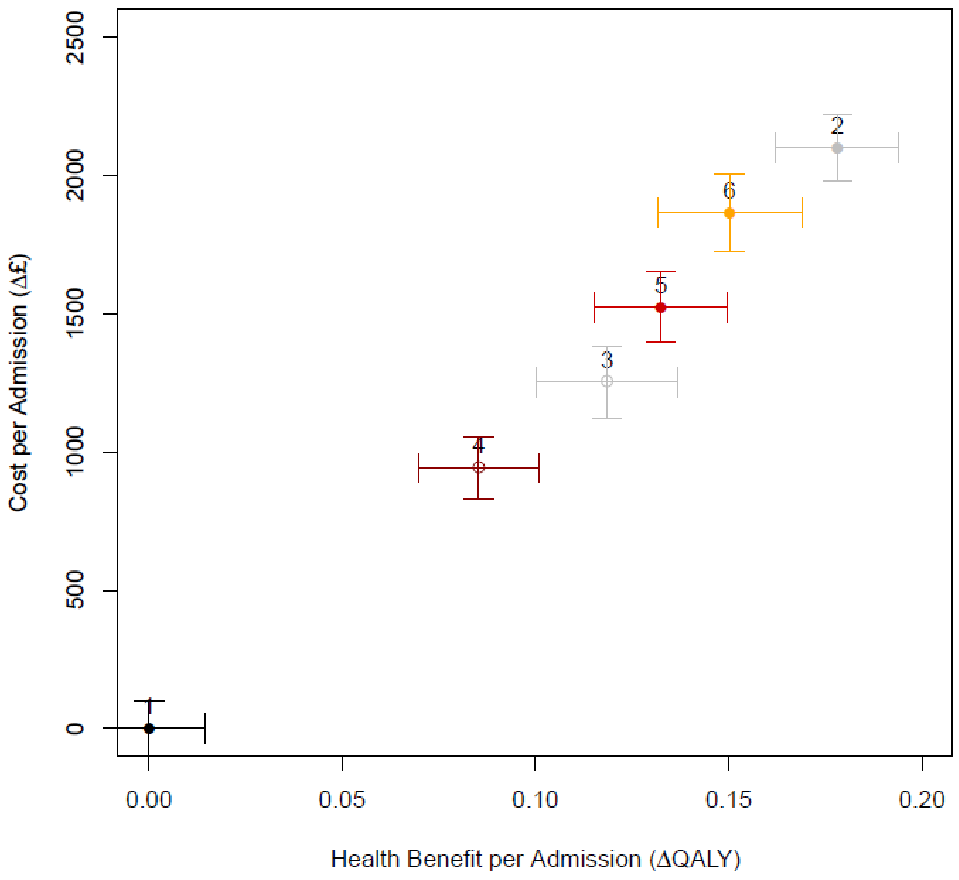

Figure C10. Cost-effectiveness acceptability curves.

Each line represents the proportion of simulations, for a particular strategy, that are cost-effective, as a function of willingness to pay for health benefits.

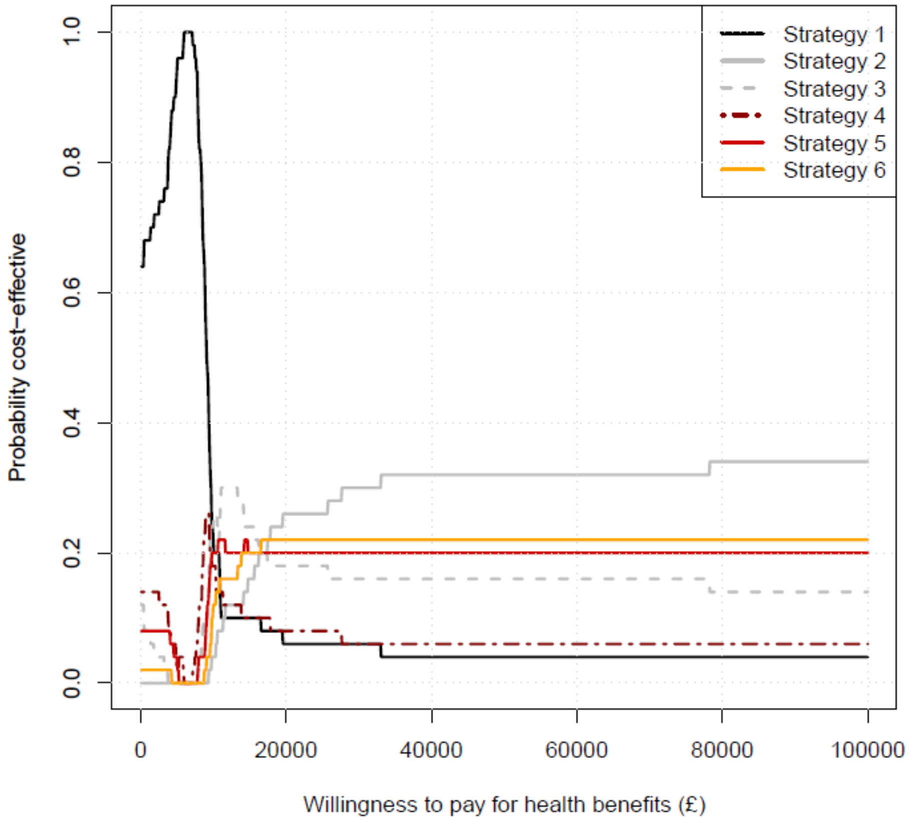

Figure C11. Cost-effectiveness acceptability frontier.

Lines depict the strategies with the highest expected net monetary benefit, dependent on the willingness to pay for health benefits, while dotted vertical lines the willingness to pay values at which the decision changes.

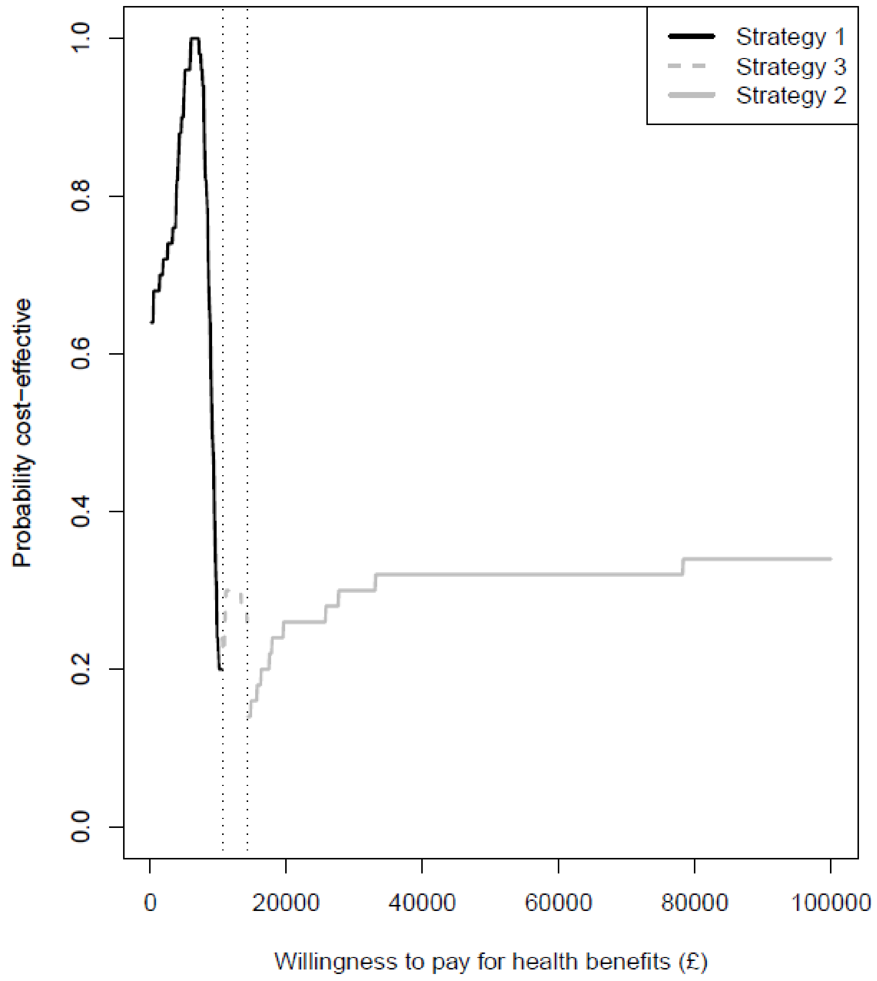

Table C5 : Cost-effectiveness of screening strategies by trust type and MRSA admission prevalence. Cost per QALY gained is shown for strategies considered both cost-effective† and non-cost-effective. Any remaining strategies for each prevalence scenario were dominated††.

Trust type	MRSA prevalence on admission	Cost-effective strategies	Non cost-effective strategies
ACUTE	High (2.80%)	Strategy 3(£8,650/QALY)	Strategy 5 (£45,257/QALY)
	Baseline (1.40%)	Strategy 3 (£9,964/QALY)	Strategy 5 (£33,806/QALY)<br>Strategy 6 (£35,796/QALY)
	Low (0.70%)	Strategy 3 (£9,796/QALY)<br>Strategy 5 (£23,196/QALY)	Strategy 6 (£87,517/QALY)
TEACHING	High (2.60%)	Strategy 3 (£20,715/QALY)	Strategy 5 (£78,258/QALY)<br>Strategy 6 (£308,839/QALY)
	Baseline (1.30%)	None of the alternative strategies were considered cost-effective.	Strategy 3 (£31,077/QALY)<br>Strategy 5 (£55,742/QALY)<br>Strategy 6 (£374,070/QALY)
	Low (0.65%)	None of the alternative strategies were considered cost-effective.	Strategy 3 (£61,710/QALY)<br>Strategy 5 (£98,634/QALY)<br>Strategy 6 (£102,797/QALY)
SPECIALIST	High (2.08%)	Strategy 3 (£9,745/QALY)	Strategy 6 (£309,264/QALY)
	Baseline (1.04%)	Strategy 3 (£10,777/QALY)<br>Strategy 6 (£14,324/QALY)	-
	Low (0.52%)	Strategy 3 (£10,566/QALY)<br>Strategy 2 (£14,224/QALY)	-

†An ICER of less than £30,000 per QALY is considered cost-effective. An ICER of more than £30,000 is not considered cost-effective. £30,000 is the upper limit of the usual NHS willingness to pay range.
††Dominated strategies are those that are more costly **and** provide less benefit than one other strategy or a combination of two other strategies. Since it can never be cost-effective to pay more for less benefit, ICERs were not calculated for these strategies.

Table C6. Cost-effectiveness of screening strategies in alternative scenarios (simulations performed for an Acute Trust setting). Cost per QALY gained is shown for strategies considered both cost-effective† and non-cost-effective. Any remaining strategies for each prevalence scenario were dominated††.

Scenario	Cost-effective strategies	Non-cost-effective strategies
Lower transmission in high-risk specialties*	Strategy 3(£12,382/QALY)	Strategy 6 (£85,713/QALY)
Reduced probability of death in high-risk specialties**	Strategy 3 (£26,511/QALY)	Strategy 6 (£296,859/QALY)
Use of the Scottish Pathfinder checklist to identify those at risk of MRSA carriage***	Strategy 3 (£9,731/QALY)	Strategy 5 (£33,206/QALY)<br>Strategy 6 (£87,517/QALY)

†An ICER of less than £30,000 per QALY is considered cost-effective, An ICER of more than £30,000 is not considered cost-effective. £30,000 is the upper limit of the usual NHS willingness to pay range.

††Dominated strategies are those that are more costly **and** provide less benefit than one other strategy or a combination of two other strategies. Since it can never be cost-effective to pay more for less benefit, ICERs were not calculated for these strategies.

* MRSA transmission rates in high-risk specialties were reduced (to be midway between values for high-risk and low-risk specialties in the baseline model (see appendix 4, table 6).

** The probability of death in high-risk specialties was reduced to the level of that in low-risk specialities (see appendix 4, table 4).

***Assumes that 26% of all admissions have a risk factor compared to the assumption used in the baseline model that 56% of all admissions have a risk factor (see appendix 4, table 1).

DISCUSSION

Summary of findings: There was an excellent response to the study with 86% of all trusts providing data to inform the modelling. It also yielded seven important findings. Firstly, implementation of emergency screening was poor at 61% (median 67% IQR 48%-85%) and even worse for eligible day case admissions (47%) (median 41%, IQR 23%-79%) although better for electives (81%) (median IQR 58%-100%)

Secondly, admission prevalence of MRSA was low at 1.5% (overall), 2.1% (emergency), 0.9% (electives) and 0.7% (day cases), with approximately half being newly identified MRSA. This meant that numbers needed to screen in order to identify one new positive were high, especially for elective (180) and day case (186) admissions, but were lower (80) for electives in specialist trusts.

Thirdly, over three quarters of trusts pre-emptively isolated those with previous MRSA with nearly half pre-emptively isolating high-risk specialty patients.

Fourthly, a third of MRSA positive and negative patients alike were discharged before the results of screening were available, as the mean turn round time was 2.87 days for positives and 1.75 days for negatives. . Decolonisation was started in ¾ of all patients screened and found to be newly MRSA positive Just over half of new positives who were still in patients were isolated. Chromogenic-agar plating was used for routine admission screening in most (82%) trusts and broth enrichment in a further 10%. Very few were using PCR test methodologies that could potentially produce faster results.

Fifthly, the point prevalence survey showed that although the overall prevalence of MRSA was 3.3% (3.6% acute, 3% teaching and 2% specialist), a third of MRSA patients were not isolated, except for specialist trusts.

Sixth, for the NHS as a whole, checklist activated screening would detect 80% of the MRSA positive patients detected by routine screening (an average of 2 less/week/trust) whilst halving the number of screens. Screening high-risk specialty admissions would detect only 10% of MRSA positive patients (although higher in teaching and specialist trusts) whilst reducing screening by 90%.

Finally, the cost effectiveness of different screening strategies, evaluated at a willingness to pay threshold of £30,000 per QALY gained, showed screening admissions to “high-risk” specialties performed best overall in different scenarios and settings. In the base case scenario none of the

decisions changed for any Trust type if the willingness to pay for a QALY gained was reduced to £20,000. However, there was substantial uncertainty, with the probability that any one strategy was the most cost-effective generally not exceeding approximately 30%. This low probability of cost-effectiveness associated with each of the screening strategies is due to the inherent uncertainty in the effectiveness of the accompanying intervention (isolation and decolonisation). If we had assumed that identifying MRSA positive patients led to placing them under an intervention that was 100% effective and prevented any further colonisations or infections, this uncertainty would be reduced. However, our estimates of the effectiveness of isolation and decolonisation represent the best available evidence, and we considered it to be important for decision makers to visualise this uncertainty *within* the decision making process.

At current admission MRSA prevalence levels, moving from current universal screening to targeted screening of high-risk specialty patients would reduce total average annual costs by £1,592,000 per acute trust, £1,864,000 per teaching and £438,000 per specialist trust, at the expense of one extra colonisation per week and less than two extra infections per year in acute and teaching trusts. For specialist trusts there would be even fewer colonisations but slightly more infections (an extra 2.6 per trust per year).

Strengths and limitations

The analyses presented have a number of strengths which are important to emphasise when comparing the findings with other model-based economic analyses. The first strength is the high response rates from trusts which enabled audit data representative of current NHS settings to populate the model. The availability of good quality economic data from the Pathfinder (Health Protection Scotland b) and MECAMIP (Robotham et al 2011) studies also contributed to the model and ensured that it was relevant to, and representative of, current NHS settings, practice and prevalence levels. The use of a powerful sophisticated individual based model, as suggested by the Pathfinder study as the next appropriate direction for modelling studies, is a particular strength. The incorporation of dynamic transmission models, with robust estimates of transmission, into economic evaluation enables consideration of population–level effects of screening strategies, which benefit not only the person screened but other patients. This avoided underestimating the effects of screening. Use of a stochastic model, with 1000 simulations per parameter set, for each strategy, minimises the uncertainty due to chance effects, which are dominant in the small populations seen in NHS trusts. Transmission parameters from the MECAMIP model were robust, being estimated using multi-state modelling

techniques to analyse detailed individual-level patient data from hospital wards in NHS hospitals (see Appendix 6).

Other strengths include simulation of the time to result delays, and the capture of more realistic patient movements, than seen in other individual based models, between specialities and between hospital and community based on detailed hospital data. Long-term effects are considered by adjusting the quality adjusted life expectancy of a proportion of infected patients who suffered long-term detriments to health associated with an MRSA infection.

The additional length of stay and additional risk of mortality due to infection are dominant economic parameters, a strength of this work being the way in which these are modelled. Each patient's daily probability of discharge and death, adjusted according to their infection status, was determined using estimates derived from previous literature, employed robust individual level data, using state of the art longitudinal Markov models [Barnett et al 2009, Robotham et al 2011). Finally the effectiveness of side-room isolation and decolonisation, and of contact precautions and decolonisation, in terms of reduction in transmissibility, was estimated from robust individual-level patient in general hospital wards (Worby et al in submission).

However, there are two important potential limitations, which must be borne in mind when using the results to inform policy and research recommendations. Firstly, there were the assumptions that both transmission and probability of death in high-risk specialties were equivalent to that in Intensive Care Units (ICUs), and that in low risk specialties was equivalent to general medical ward patients. However this was adjusted within sensitivity analyses, assuming that transmission in high-risk settings was intermediate between ICUs and general medical wards and that probabilities of death for the whole patient population are equivalent to those in general medical wards. Results were robust to these parameter changes and this is important since differences in transmission parameters had a far greater impact on the strategy evaluation than differences in MRSA admission prevalence.

The second main limitation was that transmission was modelled at a specialty level, with homogenous mixing assumed between all patients within the high-risk specialties and all patients within the low-risk specialties, which might have especially affected large teaching trusts. A greater level of 'granularity' i.e. including a ward-based structure, and inclusion of ICUs in particular, would enhance the model, as patients in different wards and geographic locations will have different abilities for cross -transmission. Further modelling, incorporating data from the sentinel sites on inter-ward transfers from ICUs to the

rest of the hospital (ICUs being potential reservoirs of MRSA, acting as a “carousel” to spread the organism round the hospital), data on inter-ward transfers in general throughout a patient’s hospital journey and data on readmission rates of MRSA positive and negative patients, would have led to still more realistic transmission dynamics, and thus more reliable evaluation of screening strategies. Incorporating these unique data, whose collection had not been envisaged at the start of the NOW study, into the current transmission models to consider transmission at a more detailed ward level would have been helpful. This would be especially relevant in teaching trusts, since, in this setting high-risk specialty screening was only slightly more costly than the willingness to pay threshold, and since under conditions of low MRSA prevalence, no strategy appeared to be cost effective. In addition, modelling of the effects of excluding day case or elective screening and of the effects of pre-emptive isolation of all patients with a history of MRSA , which the audit data showed most trusts tried to do, would have also enhanced the applicability of the model to the current NHS. Other potential limitations were assumptions there was no increased mupirocin resistance due to its widespread use in decolonisation and that isolation had no adverse effects, although some evidence suggests isolated patients have fewer contacts with healthcare workers (Kirkland & Weinstein 1999, Saint et al 2003, Evans , et al 2003, Stelfox et al 2003), may receive suboptimal care for some conditions (Morgan et al 2011) or be more depressed (Tarzi et al 2001). Despite these limitations, however, it should be recognised that the extended MECAMIP model is now more sophisticated and powerful than any comparable model in this field and in its entirety represents 5 years-worth of modelling.

Comparison with other prevalence studies

The overall proportions (~1.5%), of MRSA positives identified on admission screening was lower than reported in recent years for routine admission screening in whole hospitals (2.4-8.6%) (Health Protection Scotland 2011a, Rao et al 2007). The likely reason for this is that levels of MRSA are lower, the English mandatory reporting system (HPA 2011) having shown that bloodstream MRSA infections decreased by 38% between April 2009 and June 2011, as multiple national infection control interventions took effect (Stone et al 2012). The Scottish Pathfinder Study also [Health Protection Scotland 2011a] described a decline in prevalence of MRSA colonisation from 5.5% to 3.5% in a one year period (2008-2009 and, although limited to acute care of the elderly wards an Irish teaching hospital study reported a decline from 9% to 2% in 2007-10 (Creamer et al 2012).

Our point prevalence estimate for any MRSA infection (0.31%), although based on a proxy measure of prescription of anti-MRSA antibiotics and not standard criteria for Healthcare associated infection, was similar to the overall annual incidence of 0.52% reported in the Pathfinder study (Health Protection

Scotland 2011a) which used more formal criteria. This is lower than the 1.5% reported in the 2006 Hospital Infection Society's Health Care Associated Infection national prevalence survey (Smyth et al 2008), which used similar case ascertainment criteria to Pathfinder and higher than a more recent national prevalence survey (HPA 2012) which reported that <0.1% of patients surveyed had an MRSA infection. Just 1% of HCAI detected named MRSA as the causative organism.(HPA 2012)

The lower figures in NOW and Pathfinder and the 2011 point prevalence survey probably reflect the same temporal and infection control trends that govern admission prevalence. Both Pathfinder and NOW reported that approximately half the admission prevalence was made up of newly identified MRSA positives (Health Protection Scotland 2011 a, b). It is not possible to compare admission prevalence in specific clinical specialities with those reported in these other studies as this was not part of the NOW study's remit. Infection control teams returning data for the NOW audit were not asked to link admission screens to individual admissions (except for the newly identified MRSA patients and the randomly selected sample of MRSA negative patients), but to provide total numbers of screens and admissions in the audit week. In contrast, Pathfinder had a team of research nurses to provide the wealth of detail on patients and patient populations detailed in their report.

The proportion of MRSA positive patients who would be identified by checklist activated screening (81%) was virtually the same as that reported in the three Scottish Pathfinder Health Boards [Smith et al 2011] and from Rao et al's study (2007) in an English hospital (84.5%), although we found a slightly lower proportion of admissions would be checklist positive (51% v 57.6% (Rao et al 2007)] v 57% (Smith et al 2011) and require screening. There were some differences between studies in that Rao's (2007)checklist was virtually the same as ours whereas the full Pathfinder checklist was much larger (12 items versus 6).

Pathfinder also examined use of a three item checklist (previous history of MRSA, presence of wounds or indwelling devices, and admissions not from home), which in fact covers five of the six NOW checklist risk factors, (excluding admission to hospital within the last year). They found that only 10% of their admissions would be checklist positive, but that the checklist would detect 50.4% of MRSA positive patients. When we applied the three-item checklist to our data we found that 26% of admissions were checklist positive and that 68% of MRSA+ve admissions would be identified. This difference between the studies is not easy to explain but may reflect differences in sampling and the types of hospitals studied. The NOW study was based on data collected from nearly all acute English Trusts, whereas the Pathfinder study is based on 6 hospitals, in three Boards, where the annual

number of admissions (81,438 between them) is much lower than in the average teaching or acute trust in England (nearly 100,000 and 60,000 respectively).

Implementation of screening

The proportion of patients screened on admission was lower In the NOW study than in Pathfinder [Health Protection Scotland 2011a] where 85% of emergency admissions and 98% of elective admissions were screened. This may reflect the fact that data collected for NOW is representative of English rather than Scottish hospitals, but it could also reflect the fact that the Pathfinder hospitals were enrolled in a specific study, with a team of research nurses following admissions and reminding wards to screen if they had overlooked individual patients. The relatively low compliance that we report in England, with a national mandatory universal admission screening programme, which should be the easiest of all screening policies to routinise and implement, should give considerable pause for thought in respect of how well any policy of checklist activated screening would be implemented. The Pathfinder study was able to use research nurses to administer the checklist whereas in the NOW study, infection control nurses used the standard prevalence surveillance techniques of interrogation of hospital data bases, review of notes and examination or interview of patients, and entered more don't-know responses, which may be more representative of actual clinical practice. This indicates that implementation of a checklist by admitting nurses on wards is likely to be poor and should not be considered as a feasible option.

Turn round time for the processing of samples appears shorter in Pathfinder (Health Protection Scotland 2011b) with a median (IQR) of 28 hours (24-43) for negative results and of 48 hours (25-63). The proportions of MRSA positive patients discharged before the result was available is similar in both studies but a much smaller proportion of positive patients were decolonised (47%) in Pathfinder (Health Protection Scotland 2011b) although the proportions of patients isolated and some stage in their admission appears similar, isolation capacity per ward being very similar.

Comparison with other modelling studies:

The most important comparisons are with the Department of Health impact assessment (Appendix 1) the Scottish HTA model (Ritchie et al 2007)and the subsequent Scottish Pathfinder study (Health Protection Scotland 2011 a,b,c,d) . Non-UK or Republic of Ireland modelling (Hubben et al 2012, Murthy et al 2010, Lee et al 2009) or clinical studies (Harbath et al 2008) are not directly compared to

this study due to differences in setting, their concentration on a single or limited number of specialties, rather than considering a whole hospital, or their evaluation of universal PCR screening, which is little used in the UK, with a much smaller range of alternative screening policies than those considered in the NOW project.

The Department of Health impact assessment did not account for transmission, considering only patient level events limited to those patients colonised on admission, evaluated health benefits using deaths avoided (each death having a value of £250,000) and had a much higher estimation of the effectiveness of isolation and decolonisation at 90%, whereas our model estimated reduction in transmission for primary isolation at 64% (SD 14%), and at 24% (SD 12%) for contact precautions and decolonisation (Worby et al in submission) These parameters have previously been found to exert the greatest influence and this may explain why the DH policy of screening all admissions rarely proved cost-effective in our models, which incorporated full uncertainty of these parameters in order to maximise robustness of results.

The Scottish HTA prevalence (Ritchie et al 2007) model was limited, as its authors acknowledged, by substantial uncertainty in parameter estimation, which they did not attempt to adjust for. A key assumption of the HTA model and therefore incorporated into the Pathfinder model, is that isolation reduces transmission to 0%. Our model included full uncertainty distributions for intervention parameters and used national audit data whenever possible. The subsequent Pathfinder study (Health Protection Scotland 2011 a, b,c ,d), published during planning stages of our study, provided an excellent review and valuable general model of MRSA screening and associated cost-effectiveness. The authors explicitly recommended that the next step for modelling should be an individual-based approach (see introduction above), as conducted in this study, so that patient movements, MRSA transmission and control brought about through screening, isolation and decolonisation were modelled at an individual-patient level. The stochastic modelling used in this study calculated each patient's probability of colonisation or infection on a daily basis, which depended on how many such patients they were surrounded by, and what screening, isolation and decolonisation interventions these were receiving (which might also change on a daily basis). Other key differences include extensive parameterisation of transmission which could change daily, the modelling of uncertainty, and the use of the MECAMIP model to estimate the effectiveness of decolonisation and isolation (Robotham et al 2011) using Markov state modelling techniques. For instance a key assumption of the Scottish HTA study (Ritchie 2007) and the subsequent Pathfinder report (Health Protection Scotland 2011b) was that

isolation reduces transmission to zero, this contrasts with the assumption used here that isolation is 64% effective.

Final important differences are incorporation of real patient movement data and patient level differences in probabilities of discharge and mortality , according to whether they are in a high or low risk specialty, their length of hospital stay and whether they were infected or not . Readmissions were modelled at an individual patient level, with probabilities of readmission calculated from real hospital data and dependent on previous admission history. Length of time between readmissions was modelled from a full distribution based on real hospital data.

These comparisons between modelling studies probably underlie the differences in results. The HTA model (Ritchie et al 2007) suggests routine screening of all admissions was effective, especially when combined with pre-emptive isolation of high-risk specialty patients. However, long term differences in prevalence levels differed little between screening strategies, but there were substantial variations in five-year costs, with the possibility that that teaching hospitals could save up to £2M and non-teaching hospitals more than £1M over five years by using other risk based screening strategies (checklist activated or specialty based) instead of routine admission screening. The NOW study reports not only that screening all admissions to high risk specialties is in general more cost effective, the savings to be made are considerably greater than estimated by the Scottish HTA model (Ritchie et al 2007).

It is harder to compare Pathfinder and NOW cost effectiveness modelling for two main reasons. Firstly their definition of high-risk specialties was much broader so that 68% of their admissions came under this category (Health Protection Scotland 2011a). If they had used the NOW definition of high-risk specialties the proportion would have been 21% for elective and emergencies combined. This is still nearly twice the proportion in the NOW audit where 11.75% of admissions were to high risk specialties across the entire population of the three trust types (13% if day cases, which are not specifically mentioned in Pathfinder, are excluded) and suggests a difference in clinical practice between England and Scotland.

Secondly, although modelling of screening admission to high risk specialties was undertaken (Health Protection Scotland 2011b, Stewart et al 2011), no cost effectiveness plane, CEAC or CEAF results are presented. Pathfinder found that although routine screening of all admissions reduced infections and acquisitions more than any other strategy, although the difference was not statistically significant. The

most cost-effective strategy was checklist activated screening of all admissions (using the three item checklist, with nasal and perineal swabbing of those with at least one risk factor), equivalent to NOW Strategy 4, followed by routine screening of all high-risk specialty patients combined with checklist activated screening of all low-risk admissions (equivalent to NOW Strategy 5), followed by routine screening by nasal swab of all admissions (equivalent to NOW Strategy 2, except that in England screening by nasal swab only is done in less than 10% of trusts). The ICERs quoted all came below the conventional willingness to pay threshold of £30, 000 although the study set no such threshold arguing that this is for policy makers to decide (Stewart et al 2011) . Pathfinder's overall conclusion was that, taking public acceptability and the economic climate into account, combining two swab screening of all admissions to high-risk specialties combined with universal check-list activated screening of all other admissions "appeared to offer the best clinical return for a similar level of financial investment to universal screening of all admissions."

The NOW cost effectiveness results

The relative cost-effectiveness of screening admissions to high-risk specialties probably derives from the difference in transmission parameters in high and low-risk specialties, and the fact that most infections occur in this population (high-risk specialties), who have a higher probability of progressing from colonisation to infection. It is infections that have the largest impact on length of stay and mortality, the largest cost and health benefit determinants. The reduction of these infections combined with lower screening and isolation costs associated with a strategy of screening only admissions to high risk specialties, makes this a more cost-effective strategy, especially in higher prevalence settings.

The differences in cost-effectiveness between acute and teaching trusts reflected the overall greater costs per admission in teaching trusts (causing any strategy to have a higher cost/QALY) and lower transmission and MRSA admission prevalence in the high risk specialty population (meaning the screening strategies had less ability to reduce transmission and infections, and therefore less ability to generate health benefits in teaching trusts). The differences in cost-effectiveness between specialist and acute hospitals reflected higher transmission rates in specialist hospitals meaning that strategies could better prevent transmission and infections and thus generate greater QALY gains, making all strategies better value for money (even the most costly ones, including screening all admissions). The small population size of specialist trusts in particular also meant that stochastic (i.e. chance) effects had a greater impact on the transmission dynamics.

It should also be noted that there existed substantial uncertainty, with the probability of any one strategy being a better option than any other of around only 30% (at willingness to pay values of £20-30,000/QALY).

Overall the results indicate that persisting with the current policy of routine screening of all admissions, does not appear to be cost effective. Reverting to the previous targeted screening strategy of screening only admissions to high-risk specialties may generate substantial savings (on average £250m per year) across the NHS for a very minimal rise in infections (approximately two per year per trust) and colonisations (approximately one per week per trust). The cost-effectiveness of this strategy was maintained even if prevalence increased to twice the current levels.

Generalisability:

Wherever possible model inputs came from audit data to reflect current status of trusts in England, with extensive sensitivity analyses undertaken demonstrating the robustness of findings to Trust type, MRSA prevalence, and assumptions regarding transmission and mortality probability parameters. Parameterisation of the model was also undertaken using audit data wherever possible, thus maximising the reflection of the current status of Trusts in England. This enabled the model to have substantial generalisability. Reassuringly, application of our model under these different analyses indicated that, for the most part, decisions were robust to parameter changes. However, the certainty in choosing between competing strategies was low and changes in costs and effects were clustered.

For the cost-effectiveness evaluations we had taken the perspective of a decision maker who manages resources at a regional or national level, and who seeks to improve the economic efficiency of healthcare services – therefore placing value on bed days freed and QALYs gained. However, from the point of view of clinical or nursing directors for example, the prompter discharge of patients, who have fewer MRSA infections, may facilitate more admissions into the bed base, increasing cost and workload. Factors such as bed management and staffing may be considered more important, and thus change the decision. Conversely, the opportunity to perform contracted work in beds no longer occupied by infected patients may be considered important and provide further support for the decision.

REFERENCES

Barnett AG, Batra R, Graves N et al ,(2009). Using a longitudinal model to estimate the effect of methicillin-resistant Staphylococcus aureus infection on length of stay in an intensive care unit. **Am J Epidemiol**. 170:1186-1194.

Coia J, Duckworth G, Edwards D et al (2006). Guidelines for control and prevention of MRSA. **J Hosp Infection**. 63S, S1-S44

Cooper BS, Medley GF, Stone SP, et al (2004). Methicillin-resistant Staphylococcus aureus in hospitals and the community: stealth dynamics and control catastrophes. **Proc Natl Acad Sci USA** , 101:10223-10228.

Cooper, G F Medley, B Cookson, G Duckworth, C C Kibbler, J A Roberts, S Ebrahim, S Stone 2004.Methicillin-resistant Staphylococcus aureus in hospitals and the community: Stealth dynamics and control catastrophes. **Proceedings National Academy of Sciences** 2004;101:10223–10228

Creamer E, Dolan A, Sherlock O et al (2010). The Effect of Rapid Screening for Methicillin-Resistant *Staphylococcus aureus* (MRSA) on the Identification and Earlier Isolation of MRSA-Positive Patients **Infect Control Hosp Epidemiol** 31:374–381;

DH (2005). Saving Lives: a delivery programme to reduce health care associated infection (HCAI) including MRSA. www.clean-safe-care.nhs.uk

DH (2006): Code of practice for the prevention and control of healthcare associated infections. London: Department of Health.www.dh.gov.uk/en/Publicationsandstatistics/Publications/PublicationsPolicyAndGuidance/DH_081927

DH (2008). Department of Health. NHS reference costs 2006-07. Published: 1st February 2008. http://www.dh.gov.uk/en/Publicationsandstatistics/Publications/PublicationsPolicyAndGuidance/DH_082571

DH 2009 The Operating framework for 2010/11 for the NHS in England http://www.connectingforhealth.nhs.uk/systemsandservices/infogov/links/operatingframework2010-2011.pdf last accessed 18/07/2012

Drummond MF, Sculpher MJ, Torrance GW, et al.(2005), Methods for the Economic Evaluation of Health Care Programmes, third edition. Oxford: Oxford University Press.

Evans HL, Shaffer MM and Hughes MG et al. (2003) Contact isolation in surgical patients: a barrier to care? **Surgery** 134:180–188.

Graves N (2004). Economics of preventing hospital infection**. Emerg Infect Dis** [serial online] 2004 Apr Available from: http://wwwnc.cdc.gov/eid/article/10/4/02-0754.htm DOI:10.3201/eid1004.020754

Harbarth S; Fankhauser C; Schrenzel J; et al. 2008 Universal Screening for Methicillin-Resistant Staphylococcus aureus at Hospital Admission and Nosocomial Infection in Surgical Patients **JAMA**.;299(10):1149-1157

Hardy K, Price C, Szczepura A et al (2009). Reduction in the rate of methicillin-resistant Staphylococcus aureus acquisition in surgical wards by rapid screening for colonization: a prospective, cross-over study. **Clin Microbiol Infect** 16:333-339.

Health Protection Scotland (2011a), National Services Scotland, NHS Scotland MRSA Screening Pathfinder Programme Final Report Volume 1: An investigation of the Clinical Effectiveness of MRSA Screening, Health Protection Scotland [Report]

Health Protection Scotland. (2011b) NHS Scotland MRSA Screening Pathfinder Programme. Final report volume 2: An assessment of the Economics, Implementation and Modelling of Universal MRSA Screening. Edinburgh:

Health Protection Scotland (2011c), National Services Scotland, Final Report Volume 3: Staff Patient Acceptability, Health Protection Scotland [Report]

Health Protection Scotland (2011d). National Services Scotland, Final Report Volume 4: To Evaluate the Feasibility and Potential for Rollout of the MRSA Screening Programme 2011, Health Protection Scotland [Report]

HES (2010). 2009/2010 Provisional linked HES-ONS mortality data aggregated by diagnosis/procedure. http://www.hesonline.org.uk/Ease/ContentServerSite last accessed 06/07/2012

HPA (2004) Staphylococcus aureus bacteraemia: England, Wales, and Northern Ireland: October to December 2003. CDR Weekly Volume 14 No 12, 18 March 2004 www.hpa.org.uk/cdr/archives/2004/staph_1204.pdf

HPA (2011). Quarterly Epidemiological Commentary: Mandatory MRSA bacteraemia, *Clostridium difficile* infection & MSSA bacteraemia (up to April – June 2011) http://www.hpa.org.uk/webc/HPAwebFile/HPAweb_C/1317131910576 last accessed 11/07/2012

HPA (2012) English National Point Prevalence Survey on Healthcare Associated Infections and Antimicrobial Use, 2011: Preliminary Data] http://www.hpa.org.uk/webw/HPAweb&HPAwebStandard/HPAweb_C/1317134306322 Last accessed 20-07-2012

Hubben G, Bootsma M, Luteijn M, Glynn D, Bishai D, et al. (2011) Modelling the Costs and Effects of Selective and Universal Hospital Admission Screening for Methicillin-Resistant Staphylococcus aureus. PLoS ONE 6(3). Available from: http://www.plosone.org/article/info%3Adoi%2F10.1371%2Fjournal.pone.0014783

Jeyaratnam D, Whitty C, Phillips K et al (2008). Impact of rapid screening tests on acquisition of methicillin resistant Staphylococcus aureus: cluster randomised crossover trial. **BMJ**; 336: 927;

Kirkland KB and Weinstein JM. (1999) Adverse effects of contact isolation**, Lancet** 354:1177–1178.

Lee B, Tsui Y, Bailey R et al (2009). Should Vascular Surgery Patients be screened pre-operatively for Methicillin-Resistant Staphylococcus Aureus? **ICHE**, 30: 1158

Morgan D, Day H, Harris D et al (2011) the impact of contact isolation on the quality of inpatient hospital care. PLOS One 6(7): e22190.doi 10.1371/journal.pone.0022190

Murthy A, De Angelis G, Pittet D et al (2010). Cost-effectiveness of universal screening on admission to surgery. **CMI**; 16:1747-1753

NAO (2009). Report by the comptroller and auditor general HC 560 session 2008-9 12 June 2009. Reducing Healthcare Associated Infections in Hospitals in England. HMSO: London.

www.nao.org.uk/publications/0809/reducing_healthcare_associated.aSpx (accessed 21st September 2009)

NPSA (2004). Ready, steady, go. The full guide to implementing the clean**your**hands campaign in your Trust. www.npsa.nhs.uk/EasySiteWeb/GatewayLink.aspx?alId=5923
Ng M, Gakidou E, Levin-Rector A, et al 2011. Assessment of population-level eff ect of Avahan, on HIV-prevention initiative in India. **Lancet** published online Oct 11. DOI:10.1016/S0140-6736(11)61390-1.)

Public accounts Committee 2009. Reducing healthcare associated infections in hospitals in England. Fifty-second report of session 2008-9. London. The Stationary Office.
www.publications.parliament.uk/pa/cm/cmpubacc.htm
PublicationsPolicyAndGuidance/DH_081927 (last accessed 18th January 2009

Rao GP. Michalczyk P, Nayeem L et al (2007). Prevalence and risk factors for meticillin-resistant *Staphylococcus aureus* in adult emergency admissions – a case for screening all patients? **J Hosp Infect** 66: 15–21

Rawlins MD, Culyer AJ. (2004). National Institute for Clinical Excellence and its value judgments. **BMJ** 329:224-227.

Ritchie K, Bradbury I, Craig J, et al (2007) Health Technology Assessment Report 9: The clinical and cost effectiveness of screening for meticillin-resistant *Staphylococcus aureus* (MRSA)

Roberts T, Robinson S, Barton P. (2004) The correct approach to modelling and evaluating chlamydia screening **Sex Transm Infect** 80:324-5

Robotham JV, Graves N, Barnett AG, et al (2011). Model-based evaluation and cost-effectiveness analysis of MRSA intervention policies. DH Report;. [DH reference number PR-IP-0807-0410026]

Saint S, Higgins LA, Nallamothu BK and Chenoweth C. (2003) Do physicians examine patients in contact isolation less frequently? A brief report. **Am J Infect Control** 31:354–356.

Smith A, Christie P, Stari T (2011) Health Protection Scotland, National Services Scotland, NHS Scotland. MRSA Screening; The Value of Nasal Swabbing versus Full Body Screening or Clinical Risk Assessment to Detect MRSA Colonisation at Admission to Hospital. Health Protection Scotland [Report]

Smyth E , McIlvenny G, Enstone J. (2008) Four Country Healthcare Associated Infection Prevalence Survey 2006: overview of the results **J Hosp Infect** 69: 230-248

Stelfox, Bates DW and Redelmeier DA (2003). Safety of patients isolated for infection control, **J Am Med Assoc** 290:1899–1905.

Stewart S, Taylor J, Marshall M, Christie P. Health Protection Scotland, National Services Scotland, NHS Scotland. MRSA Screening: Economic Analyses.2011.Health Protection Scotland ISBN 978-1-873772-42-3

Stevenson M, McClure R (2005). Use of ecological study designs for injury prevention**. Injury Prevention** ;11:2–4.)

Stone S, Fuller C, Savage J et al (2012) Evaluation of the national Cleanyourhands campaign to reduce *Staphylococcus aureus* bacteraemia and *Clostridium difficile* infection in hospitals in England and Wales by improved hand hygiene: four year, prospective, ecological, interrupted time series study. **BMJ** 344:e3005 doi: 10.1136/bmj.e3005344:e3005

Tarzi S, Kennedy P, Stone S et al (2001) Methicillin-resistant *Staphylococcus aureus*: psychological impact of hospitalization and isolation in an older adult population. **J Hosp Infect** 49: 250-254

The Health Act 2008: Code of Practice for the prevention & control of HCAIs

Worby C, Robotham J, Kypraios T, (in submission). Estimating the effectiveness of isolation and decolonisation measures in reducing MRSA transmission in general hospital wards: a model-based analysis.

APPENDICES

Appendix 1. DH impact assessment document

Summary: Intervention & Options		
Department /Agency: Department of Health	Title: Impact Assessment of screening elective patients for MRSA	
Stage: Final	Version: 0.3	Date: 6 November 2008
Related Publications: MRSA screening operational guidance		

Available to view or download at:
http://www.tinyurl.com/59nwuu

Contact for enquiries: Robin Feline **Telephone:** 020 2792 1247

What is the problem under consideration? Why is government intervention necessary?

MRSA stands for methicillin resistant Staphylococcus aureus. It is a highly contagious strain of the Staphylococcus aureus family of bacteria, which cause a number of infections, such as boils, carbuncles, infected wounds and bloodstream infection (or bacteraemia), which can be fatal. Bacteraemia can lead to septicaemia, the kind of MRSA infection that has the highest death rate. MRSA is resistant to common antibiotics.

What are the policy objectives and the intended effects?

The policy objective is to reduce the risk of infection for MRSA, and ultimately the number of infections, by screening elective inpatients for MRSA, and decolonising those found to be carriers. By issuing NHS best practice guidance on screening patients for MRSA the aim is to prevent many of the infections that might otherwise have occurred.

What policy options have been considered? Please justify any preferred option.

The following options have been considered

1. Do nothing
2. Preferred option: implement screening for elective inpatients, but excluding maternity/obs, day cases in low risk specialties, children and paeds, minor dermatology. Screening for emergency cases to follow by 2011.
3. Implement screening for all elective inpatients and day cases.
4. As option 2, but also include screening for emergency cases immediately.

When will the policy be reviewed to establish the actual costs and benefits and the achievement of the desired effects? Post implementation review will be conducted over time as we monitor the number of bacteraemias, and will also be informed by PMDU review (due November 2008).

Ministerial Sign-off For final proposal/implementation stage Impact Assessments:

I have read the Impact Assessment and I am satisfied that (a) it represents a fair and reasonable view of the expected costs, benefits and impact of the policy, and (b) the benefits justify the costs.

Signed by the responsible Minister:

[illegible] .... Date: 3-12-08

1

Summary: Analysis & Evidence

Policy Option: 2	Description: MRSA screening for all except those in low risk groups, with implementation for emergencies by March 2011

COSTS

ANNUAL COSTS		Description and scale of **key monetised costs** by 'main affected groups' Cost of screening plus decolonisation totals £1.22bn in cash terms, £1.01bn PV over 10 years. However, there is a cash saving in treatment costs of MRSA bacteraemia and wound infections of £1.19bn cash, £987m PV. Typical annual outlay is £130m per year (cash terms) from 2010-11 onwards.
**One-off** (Transition)	**Yrs**	
**£ nil**		
**Average Annual Cost** (excluding one-off)		
**£ 2.6m (cash)**		**Total Cost** (PV) **£ 19m (PV)**

Other **key non-monetised costs** by 'main affected groups'

BENEFITS

ANNUAL BENEFITS		Description and scale of **key monetised benefits** by 'main affected groups' Key benefit recorded here is 'avoided deaths'. These do not count as a saving to the NHS, but a wider societal benefit.
**One-off**	**Yrs**	
**£ nil**		
**Average Annual Benefit** (excluding one-off)		
**£ 274.6m (cash)**		**Total Benefit** (PV) **£ 2.53bn**

Other **key non-monetised benefits** by 'main affected groups'

Key Assumptions/Sensitivities/Risks Levels of risk for each patient group estimated from SA septicaemia data in HES. Also, some of the data on costs and efficacy of tests is based on current expert view - no direct link to primary evidence has been identified.

Price Base Year 2008	Time Period Years 10	**Net Benefit Range** (NPV) **£ subj to sensitivity test**	**NET BENEFIT** (NPV Best estimate) **£ 2.51bn**

What is the geographic coverage of the policy/option?			England	
On what date will the policy be implemented?			2008	
Which organisation(s) will enforce the policy?			PCTs	
What is the total annual cost of enforcement for these organisations?			£ nil	
Does enforcement comply with Hampton principles?			Yes	
Will implementation go beyond minimum EU requirements?			No	
What is the value of the proposed offsetting measure per year?			£ N/a	
What is the value of changes in greenhouse gas emissions?			£ nil	
Will the proposal have a significant impact on competition?			No	
Annual cost (£-£) per organisation (excluding one-off)	Micro	Small	Medium	Large
Are any of these organisations exempt?	No	No	N/A	N/A

Impact on Admin Burdens Baseline (2005 Prices) (Increase - Decrease)

Increase of £ Decrease of £ **Net Impact** £

Key: Annual costs and benefits: Constant Prices (Net) Present Value

Evidence Base (for summary sheets)

[Use this space (with a recommended maximum of 30 pages) to set out the evidence, analysis and detailed narrative from which you have generated your policy options or proposal. Ensure that the information is organised in such a way as to explain clearly the summary information on the preceding pages of this form.]

Background – what is the policy problem that needs to be solved?

The Staphylococcus aureus family of bacteria, to which MRSA belongs, is a very common cause of bacterial infections such as boils, carbuncles, infected wounds, deep abscesses and bloodstream infection (or bacteraemia). When penicillin was introduced in the 1940s, it helped tackle these infections, but after a while some strains of the bacteria began to become resistant to the antibiotic and by 1959, about 90-95% of S.aureus strains isolated from patients with clinical infections were resistant to penicillin. Methicillin (and, later, cloxacillin and flucloxacillin) was therefore developed, from penicillin, to treat these new strains with some success. Although the first case of MRSA was reported in England within a year of the launch of meticillin, MRSA was relatively uncommon through the 1960s and 1970s, and only a few more cases appeared in the 1980s.

In the mid -1990s, however, "epidemic" strains of MRSA became established in hospitals throughout the UK. These strains are easily transmissible (passing between and colonising both patients and hospital staff easily) and have the capacity to cause serious disease.

There is a high level of public concern about healthcare associated infections, and political and NHS determination both to reduce the number of MRSA bacteraemias and to bolster public confidence.

Most MRSA bacteraemias occur in people over 65, and primarily in men. In 2006/07 across England, 6,383 cases were reported. This impact assessment assesses the evidence without assuming that any existing public commitment is the right way to proceed. However we note that there is a public commitment to reduce MRSA infections, and PSA 19 includes a commitment to DH, "implementing best practice, for example through developing best practice guidance and spreading knowledge........". By examining the available evidence on costs and benefits this impact assessment provides an evidence base to support the preferred option, which is to implement screening now for all elective inpatients (with certain exceptions) and to implement screening for emergency admissions by March 2011.

Why is Government intervention necessary?

Existing guidance leaves it open to NHS organisations to implement MRSA screening regimes. However, despite encouraging progress in reducing levels of infection there is scope to reduce the number of MRSA bacteraemia further.

MRSA infection is distressing to patients and their families, and the perceived risk of infection is also worrying to patients. In reality, the numerical risk of MRSA bacteraemia is very low, affecting around 0.04% of all inpatients. Comprehensive screening would involve screening large numbers of patients who did not have MRSA, and also decolonising a large number of patients who would not, in any case, have developed a bacteraemia despite being colonised on the skin or in the nose.

In essence, we argue in this IA that NHS organisations are operating on the basis of imperfect information (in an economic sense). A reasoned assessment of the costs and benefits indicates that the high costs of treating MRSA, together with the cost (in terms of lost life years) for the small number who die from MRSA infection, can outweigh the cost of a comprehensive screening regime. Government intervention provides a means to share information on the scale of relative risks and costs, and to define a cost effective testing regimen.

Although some Trusts do screen for MRSA, it is apparent that the NHS have not implemented a consistent and comprehensive screening regime to date. This supports the argument that the NHS is operating on imperfect information, and that Government intervention is needed.

Broad options for reducing infection – an outline case for screening and decolonisation

The broad policy objective is to reduce the risk of transmission from skin-borne MRSA on patients (on admission to hospital) to blood stream infections in patients after NHS treatment. The preferred option explored in this IA is for a programme of screening for MRSA. There are a number of pre-existing testing techniques. Testing is followed by decolonisation for those who test positive for MRSA. Decolonisation includes a body wash, shampoo and nasal cream. (the latter with an antibiotic cream).

When considering the costs and benefits of such an approach it is sensible to consider alternative strategies, including decolonisation for all patients in the particular 'relevant' group. Some clinicians favour this approach, and in some circumstances start decolonisation whilst awaiting test results. However, use of the antibiotic cream for very large numbers of NHS patients would run the risk of developing resistance. This may be counter-productive as a strategy to tackle antibiotic resistant infection and we rule it out for that reason.

We could consider the scope for 'partial decolonisation', including the full body wash and shampoo for all patients but without the nasal cream. This approach would fail to tackle one of key routes of infection (from colonisation of the nose) and so cannot be used as a comprehensive solution. It would also be poorly targeted, requiring partial decolonisation for large numbers of patients who were not colonised. We rule out this approach for those reasons.

The preferred approach is the only appropriate means to use screening and decolonisation as a means to reduce infection. There is a theoretical enhancement to this approach, requiring partial decolonisation (without the nasal cream) for those who are negative screens (i.e. the test shows they are not colonised). However, the specificity of all the available tests is high, the number of avoided cases would therefore be very low and it is quickly apparent without a detailed assessment of the figures that this approach would not be cost effective.

We have therefore narrowed down potential solutions to variations of a screening-decolonisation strategy. The cost effectiveness of such a strategy rests on the scale of any benefits (in terms of reduced number of infections, measured in terms of quality of life and life years gain for the patient) against the direct costs of the screening and decolonisation programme itself. This will depend, in large part, on the relative risk of infection for patients in different groups.

Assumptions/ baseline data

Expert opinion is that it is generally true that colonisation precedes infection, and so that transmission from skin-borne infections to bloodstream infections is the primary infection route. However, there is potential for direct transmission in other ways, such as lapses in hand hygiene and aseptic practice. Expert opinion is that these would not, typically, represent more than 10% of cases. For the calculations presented here, we concentrate on patients identified on admission as MRSA positive. Decolonisation then removes the risk of transmission to the bloodstream. To avoid over-stating the benefits of the screening regime we assume that 10% of bloodstream infections occur by other routes and are therefore unaffected by a screening regime.

Other key assumptions and evidence used in this impact assessment are as follows:

- There is sound evidence to suggest that around 7% of admitted patients are colonised with MRSA. We assume that this figure applies equally to all admissions.
- Wound infections with MRSA are 4 times as likely as MRSA bacteraemia (supported by current figures)
- 23% of patients with MRSA bacteraemia will die as a result.
- There are no deaths from MRSA wound infections (already counted in the bacteraemia deaths).
- Currently, Trusts screen 25% of inpatients.

- Treating one case of MRSA costs £4999 at 2008-09 prices (source: Plowman et al, "The Socioeconomic Burden of Hospital Acquired Infection", using standard HCHS deflators up to 2006-07, and assuming 4.2% inflation beyond that).
- Treating one MRSA wound infection costs the same as treating MRSA bacteraemia
- A life lost from MRSA has a value of £250,000, valued in terms of QALY's (assumes 10 yrs of life at quality of 0.7, life year valued at £38.8k with 1.5% discount rate). This is a modest assumption, as average (mean) life expectancy after hospital admission is higher than 10 years for both electives and emergencies.
- The decolonisation process costs £7 at 2006-07 prices. Allowing for 4.2% price inflation puts this at £7.60 in 2008-09
- A standard medium based Chromogenic agar plating test costs £5.70 at 2006-07 prices (£6.20 at 2008-09 prices).
- A rapid PCR test costs £16.35 at 2006-07 prices (£17.75 at 2008-09 prices)
- Unless we have data to the contrary, we assume that NHS admissions grow by 3.2% per annum.
- All tests have a typical sensitivity of 90% (probability of +ve test given that patient is colonised), a specificity of 95% (probability of –ve test given that patient is negative).
- De-colonisation itself is 90% effective in the timescales required for NHS treatment (we assume that elective patients are typically tested one week before admission).

Relating the costs and benefits to level of risk

Before assessing the costs and benefits for different options, we rehearse the case for or against MRSA screening. The following calculations are not based on precise figures, but are designed to test 'orders of magnitude'. They show that the case for screening depends on the relative risk of a bacteraemia infection, given that the patient is already colonised.

The overall number of MRSA cases suggests an infection rate of around 0.05% of all patient admissions. As 7% of patients are 'colonised' on admission, this is equivalent to approximately 1 in every 140 'colonised' patients. To simplify calculations in this illustration, we assume that there are no bloodstream infections through other routes. We also assume that existing testing has already had an impact and we calculate a 'baseline' level of risk, without any screening, of about 1 in 120.

The benefits of screening are reduced incidence of bacteraemia, reduced incidence of wound infection and reduction in deaths from bacteraemia. With existing screening regimes (in which we assume that 25% of inpatients are screened, with resulting risk of 1 in 140), the costs of these undesirable outcomes are calculated as follows:

i) 7% are colonised, and of these 1 in 140 acquire a bacteraemia, 7500 cases, each costing £5k to treat.

ii) Four times that many acquire a wound infection, 30,000 cases at £5k each to treat.

iii) Of those with bacteraemia, 23% die. 1725 deaths at £0.25m each.

iv) Total baseline cost is therefore £618.75m.

If we assume that all admitted patients should be tested, with electives using the cheaper test and emergencies using PCR, we find illustrative costs as follows:

Number of admissions:	approx 15 million, of whom 4 million are emergencies
Cost of screening:	£6.20 times 11m + £17.75 times 4 million = £139.2m
Number decolonised:	7% x (sensitivity of test) + 93% x (1-specificity of test)

= 1.6425 million patients

Cost of decolonisation: £12.48m

Total cost: £151.68m

Introducing this screening regime for all elective and emergency cases would reduce the number of infections. Infections would still arise in two groups:

i) those for whom the test was insufficiently sensitive, and therefore no decolonisation takes place.

ii) Those who are colonised, but for whom the test is positive, but decolonisation is ineffective.

iii) The proportion infected is therefore:

7% *(1-sensitivity) * risk + 7% * (sensitivity) * (1-effectiveness of decolonisation) * risk.

= 7% x 0.1 x (1/120) + 7% x 0.9 x 0.1 x (1/120) = 0.0095%

iv) This implies 1425 bacteraemias, 5700 wound infections and 328 deaths.

v) The total cost of these negative impacts would be £117.625m

Hence a full screening programme would reduce the negative impacts of MRSA from £618.75m to £117.25m, a benefit of £501m, at a cost of just £151.68m.

This outline calculation suggests that the benefits of screening are an order of magnitude larger than the costs, and there is therefore a prima facie case for considering comprehensive MRSA screening. However, this conclusion depends primarily on the level of risk. Decolonisation itself is a relatively small cost and therefore the costs of screening are virtually constant, whilst the benefits depend on the level of infection risk. If the baseline risk is 1 case in 120 colonised patients, there is a net benefit. If the risks are lower, the benefits reduce. If risk falls to around 1 case in 700 colonised patients (1 in every 10,000 admissions), the cost-benefit calculation becomes neutral.

The following diagram illustrates the effect of screening on infection risk:

1. Without screening

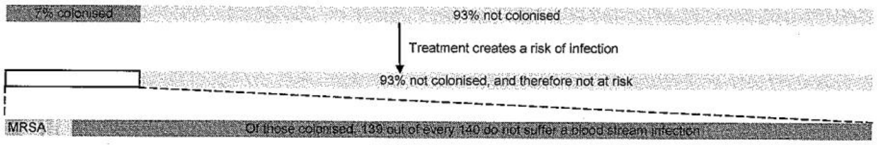

Proportion infected = proportion colonised * risk of infection for those colonised
=7% x 1/140 = 0.05%

2. With screening

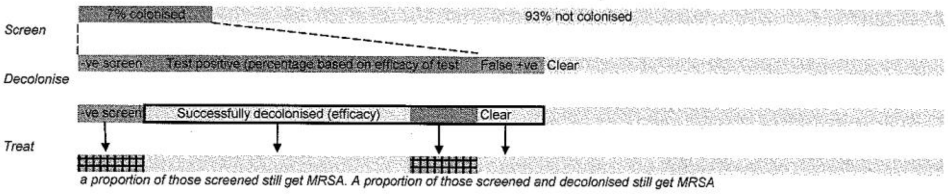

a proportion of those screened still get MRSA. A proportion of those screened and decolonised still get MRSA

Prportion infected = proportion colonised * (1- efficacy of test) * risk of infection for those colonised
+ proportion colonised * (efficacy of test) * (1- efficacy of decolonisation) * risk of infection for those colonised

= 0.07 * (0.1) * (1/140) + 0.07 * 0.9 * 0.1 * (1/140)
= 0.0095%

i.e 81% of the risk has been removed, given 90% efficacy of test and 90% efficacy of decolonisaiton

Number decolonised = proportion colonised * efficacy of test + proportion colonised * probability of false negative
= 0.07*0.9 + 0.93 * 0.05
= 0.1095

i.e Assuming 5% of clear individuals receive 'false positives and 10% of colonised individuals get false negs, 11% of patients are decolonised in total

The options – for which groups of patients does the risk justify the cost?

For the remainder of this impact assessment, we assess the cost and benefits of MRSA screening for different groups of patients. The methodology is exactly as set out above, but calculated on precise figures and with a different level of risk calculated for patients in different groups. We also factor in an assumption that up to 10% of cases are caused by other infection routes.

We know that the level of risk varies for patients in different groups. The risks are higher for those who undertake an invasive procedure. They are also higher for men than for women, and higher for those in older age groups. Routine monitoring data collected by HPA suggest that the number of patient episodes with a reported MRSA septicaemia does vary considerably by specialty. Around 1 in 27 colonised nephrology patients goes on to develop a SA septicaemia. The equivalent figure for urology is around 1 in 370.

For this IA, we have considered the evidence for the following groups of patients:

Nephrology inpatient + day case
Neurosurgery inpatient + day case
Paediatric high risk inpatient (assume 10% of all IPs)
Day case ophthalmology
Day case dental
Day case endoscopy
Dermatology day cases for minor procedures
Paediatrics (IP + DC +emergency)
Maternity/obstetrics/ births
Mental health IP+DC +emergency
Other inpatient elective admissions
Other day case elective admissions
regular day attenders
Other emergency + other

The first two groups are included as illustrative examples of high risk groups, we expect these to show much higher benefit than cost. Expert opinion is that paediatric cases are low risk except for a few in high risk categories, we therefore include 'high risk' paediatrics as a separate group. The next 7 categories have all been identified by experts as potentially low risk, and therefore potentially not justifying screening. The remaining groups capture all remaining types of admission. In most cases we assume that all admissions would be tested once. The exception is for regular day attenders, for whom we assume that approximately one in every five admissions would require a test (22% based on calculations elsewhere).

From this list of categories, we form 4 broad options:

Option 1 – do nothing. Leave the screening regime as it is now.

Option 2 – Introduce MRSA screening immediately for those groups for which benefits outweigh the costs, with the exception of 'other emergency' cases, for which we propose implementation by March 2011.

Option 3 – screen all groups

Option 4 – as option 2, but introduce screening for emergencies immediately.

Under each option, we assume that emergencies would require the faster PCR test. For elective patients there are several tests that could be used. Whilst there is some variation in sensitivity or specificity, most tests seem to have sensitivity of around 90% and specificity of around 95%. It makes sense, therefore, to leave it open to Trusts to choose the most appropriate test suitable for local needs (and probably based on cost). For this IA, we assume a cost of £6.19, based on the cost of Chromogenic agar plate test at £5 at 2006-07 prices, with two years of 4.2% inflation to give a 2008-09 price.

Costs and benefits

Option 1 – Do nothing

The costs and benefits of option 1 are, by definition, zero. But in assessing the impact of other options, we need to consider the level of testing implicit in the do nothing option, and the extent to which this impacts on any latent risk of MRSA bacteraemia. The costs for other options are therefore the costs of additional testing required for 100% coverage. The benefits are the additional benefits, achieved over and above the benefits of any existing MRSA screening.

Option 2 – Screening for those groups where benefits outweigh costs, except for emergencies for which we introduce screening by March 2011.

In assessing costs and benefits for this and subsequent options, we need some measure of risk for each patient group. To estimate this, we use HES data to determine the risk of a diagnosis of A41.0 SA septicaemia. This is not a direct proxy for MRSA (since it includes non-resistant strains), but it gives an indication of the relative risk of bacteraemia. The figures are then re-based to match the total number of cases (6383) in 2006-07. For some groups (eg day case ophthalmology) there are no cases of SA septicaemia, but for the calculations in this paper we assume a nominal number of cases.

If we assume instantaneous implementation, these calculations result in the following figures for net benefit in each group:

Table 1: balance of costs and benefits in a single year

	Total direct costs	Net benefit	Baseline number of cases
Nephrology inpatient	£664,619.45	£9,226,721.64	168
Neurosurgery	£353,183.54	£3,578,207.05	67
Paeds high risk	£440,626.97	£5,734,349.57	105
Day case ophthalmology	£3,208,689.47	-£2,947,984.90	4
Day case dental	£1,406,176.26	-£1,278,706.03	2
Day case endoscopy	£9,082,721.21	-£8,746,082.07	5
Minor dermatology electives	£1,229,330.25	-£1,063,531.97	3
Paediatrics	£8,433,027.12	-£7,019,142.74	22
Maternity/obstetrics/ births	£7,766,276.54	-£5,207,819.09	40
Mental health	£1,209,484.62	-£132,239.38	17
Other inpatient elective admissions	£16,407,376.26	£82,930,161.47	1691
Other day case elective admissions	£16,517,357.28	£40,426,748.53	889
regular day attenders	£1,663,549.67	£4,741,327.39	100
emergency + other	£80,236,802.82	£129,182,120.93	3270
Total	£148,619,221	£249,424,130	6383

Thus, MRSA screening has a total potential cost (initial outlay) of £148m per year, but shows a net benefit for most groups. For the low risk groups identified above, the costs appear to outweigh the benefits. However for one group, Mental Health, this is a very marginal result: £132k net cost on an overall spend of £1.2m. There is a degree of uncertainty in the baseline counts of MRSA cases in each group, and just two more cases in the assumed baseline figures would make net benefits for this group positive. For option 2, therefore, we include Mental Health in the initial tranche. Option 2 then implies MRSA screening in 2009-10 to include all except the 'low risk' groups and emergencies. In 2010-11 it is extended to include emergencies (the reasons for this are discussed below).

For technical reasons, the avoided cost of treating bacteraemia and wound infections count as a 'negative cost' rather than a benefit. Avoided deaths count directly as a benefit. With this proviso, the costs and benefits of this option are shown in the following table:

Table 2: Costs and benefits over 10 years of a screening programme

**Cash terms £millions**									
**Costs**	2008-09	2009-10	2010-11	2011-12	2012-13	2013-14	2014-15	2015-16	2016-17
Additional costs of screening (above opt 1) (£m)	£0	£39	£122	£126	£130	£134	£138	£143	£147
Cost of decolonisation (£m)	£0	£5	£9	£10	£10	£10	£10	£11	£11
Avoided treatment costs (£m)	£0	-£57	-£127	-£131	-£135	-£139	-£144	-£149	-£153
Total	£0	-£13	£4	£5	£5	£5	£5	£5	£5
**Benefits**									
Value of avoided deaths £m	£0	£132	£292	£301	£311	£321	£331	£342	£353
Net Benefit	£0	£145	£288	£297	£306	£316	£326	£337	£347
**NPV terms £millions**									
**Costs**									
Additional costs of screening (above opt 1) (£m)	£0	£38	£114	£113	£113	£112	£112	£111	£111
Cost of decolonisation (£m)	£0	£5	£9	£9	£9	£9	£8	£8	£8
Avoided treatment costs (£m)	£0	-£55	-£118	-£118	-£117	-£117	-£116	-£116	-£115
Total	£0	-£13	£4	£4	£4	£4	£4	£4	£4
**Benefits**									
Value of avoided deaths £m	£0	£130	£283	£288	£293	£297	£302	£307	£312
Net Benefit	£0	£143	£279	£284	£289	£293	£298	£303	£308

Thus this option has a total out-lay cost of just over £1bn in NPV terms over 10 years, with direct costs of around £130m per year in cash terms from 2010-11 onwards, increasing in line with increases in admissions. However, the net benefit of this option is substantial, at £2.5bn over 10 years.

Option 3 – screening for all groups.

A similar calculation for option 3 yields slightly higher out-lay costs, because it includes the cost of screening and decolonisation for low risk groups. The total out-lay cost is £1.36bn over 10 years (NPV). The net benefit is £2.4bn over 10 years.

The outlay costs are £164m cash in 2010-11, increasing gradually over time in line with increases in admissions (3.2% per year).

Option 4 – as for option 2, but screening for emergencies cases introduced in 2009-10 instead of 2010-11.

Under option 4, the total out-lay costs are slightly higher than option 2 because we factor in an extra year of screening for emergency cases. The total outlay is £1.086bn over 10 years (PV), with direct costs in cash terms being similar to option 2 except in 2009-10.

The net benefits of option 4 are £2.6bn over 10 years.

<u>Discussion and conclusion</u>

These calculations show that there is an outline case for introduction of comprehensive MRSA screening. Each of options 2,3 and 4 provide net benefits in comparison to the do nothing option.

Option 3 is not recommended, because it includes a number of patient groups for whom the risks of MRSA do not justify the costs. Although this option provides net benefits overall, there is a net cost implicit in screening each of the following groups:

Day case ophthalmology
Day case dental
Day case endoscopy
Dermatology day cases for minor procedures
Paediatrics (IP + DC +emergency)
Maternity/obstetrics/ births
Mental health IP+DC +emergency

The net cost for Mental health, however, is very small and within the margin of error for these calculations. We therefore do not include mental health in the list of exclusions under option 2.

Option 4 provides the greatest net benefit. However, there are two practical reasons for not recommending this option:

i) CSR funding has been secured to support this policy. This funding provides £70m in 2009-10 and £130m in 2010-11. Although this cost-benefit analysis suggests that there would be 'saved costs' from reductions in MRSA cases, this funding will not

necessarily be directly available at the point of testing. It makes sense, therefore, to constrain the outlay costs to be below these benchmarks. Since option 4 has an outlay of £127m in cash terms in 2009-10 it is not 'affordable' in the sense defined here. In practice, the NHS may be able to implement some screening for emergency cases as the benefits of elective screening reduce treatment costs.

ii) There are potential issues of practicality in introducing 11 million tests in 2009-10. Such an approach would put pressure on testing facilities. It is also likely to be more difficult administratively to implement testing for emergency cases (although the administration of testing is included in the costs described above).

Option 2 is therefore the preferred option. It has net benefits to the service, it avoids unnecessary testing for patients in very low risk groups and has the advantage of being deliverable in a practical sense.

Sensitivities

The costs of screening and decolonisation are not subject to substantial variation or error in calculation. The key sensitivities in the above analysis relate to estimates of the levels of risk within different patient groups and the extent to which the route of infection addressed by screening covers all, or virtually all, cases of bloodstream infection. In this section, we test these sensitivities.

Table 1 above shows the costs and net benefits for each patient group in a single year, assuming that a screening programme is in operation for all patient groups. This table is based on the assumption that baseline risk of infection can be estimated by looking at the number of infections in 2006-07. If we assume that baseline risk is much lower than this, for example 40% of the level suggested by 2006-07, then table 1 is amended as follows:

Table 1a: cost benefit calculations with baseline risk reduced to 40% of 2006-07 levels

	Total direct costs	Net benefit	Baseline number of cases
Nephrology inpatient	£664,619.45	£3,291,916.99	67
Neurosurgery	£353,183.54	£1,219,372.70	27
Paeds high risk	£440,626.97	£2,029,363.65	42
Day case ophthalmology	£3,208,689.47	-£3,104,407.64	2
Day case dental	£1,406,176.26	-£1,355,188.17	1
Day case endoscopy	£9,082,721.21	-£8,948,065.56	2
Minor dermatology electives	£1,229,330.25	-£1,163,010.94	1
Paediatrics	£8,433,027.12	-£7,867,473.36	9
Maternity/obstetrics/ births	£7,766,276.54	-£6,742,893.56	16
Mental health	£1,209,484.62	-£778,586.52	7
Other inpatient elective admissions	£16,407,376.26	£23,327,638.83	676
Other day case elective admissions	£16,517,357.28	£6,260,285.05	356
regular day attenders	£1,663,549.67	£898,401.15	40
emergency + other	£80,236,802.82	£3,530,766.68	1308
Total	£148,619,221	£10,598,119	2553

The broad pattern of the table is similar to our central model, in that most groups show a positive net benefit from screening. Lower risk groups show a net cost. We note here that for Mental Health patients the benefits of screening are negative, to the order of three quarters of a million pounds per year. However, as in the central model, these figures are based on very small numbers of cases. The total outlay on screening for mental health patients is small, at less

than 1% of the total out-lay cost, and it is sensible to include this group in the screening programme given:

a) uncertainty about the level of risk
b) very large potential benefits to the small number of individual patients who avoid MRSA infection.

The second area of sensitivity is the extent to which this transmission route describes all or virtually all cases of infection. In our central model we assume that 90% of cases are due to this transmission route (and it is possible, for example, that this figure is even higher in reality). Assuming that all cases are due to 'skin to blood' transmission makes virtually no difference to the substantive argument. The benefits are slightly larger for all groups, and for Mental Health patients there is a very small net cost (around £12k).

If we assume that only 80% of cases are due to this transmission route, the pattern is again as shown in tables 1 and 1a. The net cost for Mental Health patients is around £0.25m.

We would need to assume that as many as 65% of cases are caused by other mechanisms for any of the other groups included in the analysis to show a net cost. This would not be a realistic assumption.

What this sensitivity analysis shows is that the analysis is robust to variation in the key assumptions. The conclusion of this impact assessment makes qualitative arguments for the inclusion of Mental Health patients in the screening programme, despite a small net cost. Under certain assumptions this net cost is higher, but the substantive argument remains the same.

Specific Impact Tests: Checklist

Use the table below to demonstrate how broadly you have considered the potential impacts of your policy options.

Ensure that the results of any tests that impact on the cost-benefit analysis are contained within the main evidence base; other results may be annexed.

**Type of testing undertaken**	***Results in Evidence Base?***	***Results annexed?***
Competition Assessment	Yes/No	Yes/No
Small Firms Impact Test	Yes/No	Yes/No
Legal Aid	Yes/No	Yes/No
Sustainable Development	Yes/No	Yes/No
Carbon Assessment	Yes/No	Yes/No
Other Environment	Yes/No	Yes/No
Health Impact Assessment	Yes/No	Yes/No
Race Equality	Yes/No	Yes/No
Disability Equality	Yes/No	Yes/No
Gender Equality	Yes/No	Yes/No
Human Rights	Yes/No	Yes/No
Rural Proofing	Yes/No	Yes/No

Annexes

An Equality Impact Assessment has been carried out on the programme to reduce the number of MRSA infections, and which covers the screening aspects, is published on the Department of Health website

MRSA equality impact assessment : Department of Health - Publications

A full Health Impact Assessment has been considered unnecessary, the rationale for which is reproduced here.

MRSA Screening Health Impact Assessment

The MRSA screening programme set out in this Impact Assessment has additionally been considered in terms of the need for a full Health Impact Assessment. To determine whether a Health Impact Assessment is necessary the standard three screening questions have been considered as follows:

1. Will your policy have a significant impact on human health by virtue of its effects on the following wider determinants of health?

Income
Environment
Housing
Employment
Social cohesion
Crime
Transport
Education
Agriculture

The screening programme is concerned purely with its effectiveness in reducing MRSA in the healthcare environment – there appears to be little potential for significant (either positive or negative) impact in the areas described above.

2. Will there be a significant impact on any of the following lifestyle related variables?

Physical activity
Smoking, drugs, or alcohol use
Accidents and stress at home or work
Diet
Sexual behaviour

Similar to Q1 above, there appears to be little potential for significant (either positive or negative) impact in these areas.

3. Is there likely to be a significant demand on any of the following health and social care services?

Primary care
Hospital care
Accident or emergency attendances
Health protection and preparedness response
Community services
Need for medicines
Social services

There will be a demand for screening and decolonisation (where necessary) in hospital care; this has been funded through CSR (included in the general tarrif). The extent to which primary care are involved is a matter for local determination.

As the answers to two or more of these questions are "no", a full health impact assessment has not been conducted. That said, the cost benefit analysis included in the IA centres on the health benefits to patients against the cost to the NHS.

APPENDIX 2

The NOW Questionnaire

The National One Week Prevalence Audit of MRSA Screening

Trust

Dear Colleague

The NOW study- the National One Week prevalence audit of MRSA screening

The Department of Health (England) has commissioned an independent review of the implementation, clinical and cost-effectiveness, and impact on patient management of the national MRSA screening programme. This review is supported by the Department of Health, the British Infection Association, the Infection Prevention Society, the Hospital Infection Society, the NHS Confederation and the patients' group National Concern for Healthcare Infections.

As of December 2010, national policy is to screen all relevant elective and emergency hospital admissions for MRSA. This policy was based on the best available evidence at the time, but it is important that we understand how policy is being implemented and review it as new data becomes available. This will help the NHS achieve real patient benefits from screening that are appropriate to local circumstances.

This is a real opportunity for you to influence future policy.

Although participation is voluntary, the success of this review, whose primary output will be a report to the Department of Health, depends on your input and we hope that you will take part.

Yours sincerely

Dr Sheldon Stone (UCL)   Prof Barry Cookson (HPA)   Prof Brian Duerden CBE

CONTENTS

Instructions for completing the questionnaire

Definitions

Section 1a: Admission Data

Section 1b: MRSA Admission Screening Data

Section 2: Local Practice

Section 3: MRSA Point Prevalence Audit

Section 4: Individual Patient Audits (MRSA +ves)

Section 5: Individual Patient Audits (MRSA -ves)

Acknowledgements

INSTRUCTIONS FOR COMPLETING THE QUESTIONNAIRE

Thank you for taking the time to complete this audit. The questionnaire is divided into 6 sections each of which is likely to take approximately one hour to complete, depending on the size of your trust and your data collection systems.

If there are sections or individual questions that you cannot answer please leave them blank.

Sections 1a and 1b: Admissions and MRSA screening data. Retrospective data collection, 11th April – 17th April.

Section 2: Description of your local policy and practice.

Section 3: Point prevalence audit of all MRSA +ve patients in the trust on the 11th May 2011.

Section 4: Individual patient level audit. All new MRSA +ve patients identified on admission screening. Prospective data collection, 9th May – 15th May 2011.

Section 5: Individual patient level audit. A sample of MRSA -ve patients identified on admission screening. Prospective data collection, 9th May – 15th May 2011.

We have included 30 separate sheets for sections 4 & 5, but if you need more, you'll find them on the IDRN website:
www.idrn.org/audit.php

The DH has requested that we provide them with a report in August 2011, in order that the results can be used to inform policy for the following year. If you could return the questionnaire in the pre-paid envelope enclosed by **Friday 20th May** it would be much appreciated.

Christopher Fuller
Research Fellow
UCL Research Dept of Infection and Population Health
UCL (Hampstead Campus)
Royal Free Hospital
London
NW3 2PF

christopher.fuller@ucl.ac.uk

DEFINITIONS

Section 1a: Admission Data

Q1 – 5: All admissions (including paediatric).

Q1 – including emergency day-cases

Q3 – including elective day-cases

Section 1b: MRSA Admission Screening Data

Q10 – 12: Include admission screens for elective patients and pre-admission screens taken in that week. Include day-cases.

Q9, 12, 15: All patients identified as being **MRSA +ve** on admission who are flagged on your surveillance system as ***previously*** MRSA +ve.

Section 2: Local Practice

Q21 – 34: **Routinely**: We ask what you do routinely. If practice varies i.e. between hospitals, wards etc. record what happens in the majority of situations.

MRSA screening

Q18: **Emergency maternity**: i.e. unbooked, non-vaginal deliveries.

Q21: **Charge per swab**: If you pool nasal and groin/perineal swabs record the price for the pooled sample. If you test swabs separately record the price for each individual swab.

Patient Management

Q30, 31, 32: **Suppression/decolonisation**: the use of topical and/or or systemic antibiotics/antiseptics to achieve a temporary reduction in the bacterial load of MRSA or the use of topical and/or systemic antibiotics/antiseptics to clear colonised sites of MRSA.

Q33: **Contact precautions**: use of gloves and aprons for any contact with the patient or patient’s environment, in addition to standard infection control procedures.

Q34: **Isolation:** the physical separation of MRSA +ve patients and MRSA -ve patients. (i.e. in a sideroom/cohort bay/MRSA ward). *For the purposes of this audit MRSA +ve patients sharing a room/bay with MRSA -ve patients are not considered to be in isolation.*

Q30, Q31, Q33, Q34: **High risk categories**: Patients who are deemed to be at high risk of MRSA carriage (not including those who were previously known to be MRSA positive).

Q30, Q34: **High risk wards**: Areas where overall MRSA prevalence may be low, but the consequences of an MRSA infection are severe.

Section 3: MRSA Point Prevalence Audit

Q40. If wards share specialties, include only those wards for which more than 50% of patients belong to that specialty.

Q41: **Siderooms**: Include **all** rooms that are potentially available for isolating patients with MRSA (occupied and unoccupied). Do not include rooms that are currently out of commission i.e. for maintenance work.

Q45, 49: **MRSA ward**: A designated ward for isolation of MRSA patients only. May be purpose built or improvised, with or without controlled ventilation. **Isolation ward**: A designated ward for isolation of patients with infectious diseases including, but not exclusively used by, MRSA patients.

Q46 – Q52: Include all patients who have been confirmed MRSA +ve on this admission and have ***not*** had 3 consecutive negative swabs.

Q48: **Sideroom isolation**: The isolation of MRSA patients in a single bedded room. Includes the use of two bedded rooms for the isolation of one patient at a time. May or may not have en-suite facilities.

Q50: **Cohort bay**: The physical separation of MRSA patients from non-MRSA patients in a separate area of the same ward. This area may or may not be closed off from the rest of the ward. Staff may or may not be designated solely to care for MRSA +ve patients.

Q51: **Isolation**: The physical separation of MRSA +ve patients and MRSA -ve patients. (*See Q34 above*)

Sections 4 and 5: Individual Patient Audits

Q5: **Date swab taken**: Include only MRSA screen taken on admission to hospital (as day-case or inpatient) or as a pre-admission screen for elective patients (day-case or inpatient). Do **not** include screens taken after the beginning of day 3 of admission.

Q6: **Date first available**: Record date that screen first became available to the ICT for action.

Q9: **MAU**: Medical Assessment Unit. **SAU**: Surgical Assessment Unit.

Q11, 12: Include patients not yet admitted, i.e. those screened in pre-admission clinics.

Q15: **Wounds, pressure ulcers etc.**: Include any breaks to the skin (except for those created by in situ devices (see below)). Do **not** include skin breaks that occurred after admission.

Q16: **In situ devices**: Include any instrument that breaches the skin or has been introduced into a body cavity. Do **not** include any devices inserted on admission such as urinary catheters, CVP lines, IV cannulae inserted in A+E or admission ward.

Q18, 19, 20, 21: **Isolation:** The physical separation of MRSA +ve patients and MRSA -ve patients. (*see Q34 above*)

Q19, 21: **Sideroom**: The isolation of MRSA patients in a single bedded room. Include the use of two bedded rooms for the isolation of one patient. May or may not be en-suite.

Q19, 21: **Cohort bay on ward**: The physical separation of MRSA patients from non-MRSA patients in a separate area of the same ward. (*See Q50 above*)

Q19, 21: **MRSA ward**: A designated ward for isolation of MRSA patients only. May be purpose built or improvised, with or without controlled ventilation.

Q19, 21: **Isolation ward**: A designated ward for isolation of patients with infectious diseases including, but not exclusively used by, MRSA patients.

Q22: **Suppression/Decolonisation:** (*See Q 30-32 above*)

SECTION 1A: ADMISSION DATA
(For the week of 11/04/2011 – 17/04/2011)

Instructions

This section should be completed retrospectively for the week commencing 00.01hrs on Monday 11/04/2011 and ending at 23:59 hrs on Sunday 17/04/2011. This should allow time for your Trust information systems department to prepare and clean the data for the audit week.

In the week commencing **11/04/2011** (Monday to Sunday)

1. No. of **emergency** admissions…………………………………………………….
2. No. of emergency admissions to the following consultant specialties:

   Haematology/Oncology………………………………………………………………
   Nephrology ……………………………………………………………………………
   Trauma & Orthopaedics ……………………………………………………………….
   Neurosurgery …………………………………………………………………………
   Cardiothoracic surgery………………………………………………………………
   Vascular surgery………………………………………………………………………

3. No. of **elective** admissions………………………………………………………….
4. No. of elective admissions to the following consultant specialities:

   Haematology/Oncology………………………………………………………………
   Nephrology…………………………………………………………………………….
   Trauma & Orthopaedics……………………………………………………………..
   Neurosurgery………………………………………………………………………….
   Cardiothoracic surgery………………………………………………………………
   Vascular surgery……………………………………………………………………...

5. Total number of **day-case** admissions……………………………………………..
6. Total number of day-case admissions ***excluding*** the following areas: *Dermatology, Endoscopy, Ophthalmic, Paediatrics*.

………………………………………………………………………………………………

SECTION 1B: MRSA ADMISSION SCREENING DATA
(For the week of 11/04/2011 – 17/04/2011)

Instructions

In this section we ask you to provide data on Trust compliance with MRSA screening and proportions of patients found to be MRSA positive on admission/pre-admission screening. Individually named Trust data will not be passed on to the DH or SHAs.

This section should be completed retrospectively for the week commencing 00.01hrs on Monday 11/04/2011 and ending at 23:59 hrs on Sunday 17/04/2011. This should allow time for your microbiology laboratory to prepare and clean the data ready for the audit week.

7. No. of **emergency admissions** screened for MRSA……………………………

8. No. of emergency admissions MRSA +ve………………………………………...

9. No. of those MRSA +ve admissions previously known to have MRSA…………

10. No. of **elective admissions/pre-admissions** screened for MRSA …………

11. No. of elective admissions MRSA +ve…………………………………………….

12. No. of those MRSA +ve admissions previously known to have MRSA………..

13. No. of **day-case admissions** screened for MRSA………………………………

14. No. of day-case admissions MRSA +ve…………………………………………...

15. No. of those MRSA +ve admissions previously known to have MRSA ………..

SECTION 2: LOCAL PRACTICE

Instructions

In this section we ask you to provide data on Trust policy and practice around MRSA admission screening. If you are asked to tick one option only, please choose the option which is most common for your trust.

MRSA Screening

16. When did you start MRSA admission screening for all **emergency** admissions?

    MM/YYYY

17. When did you start MRSA admission screening for all **elective** admissions?

    MM/YYYY

18. Which groups do you routinely **exclude** from MRSA **admission** screening? (tick all that apply)

    None ☐
    Dermatology day-cases ☐
    Opthalmic day-cases ☐
    Endoscopy ☐
    Paediatrics (low risk groups) ☐
    Emergency maternity ☐
    Dental day-cases ☐
    Other (please state) ………………………………………………………………

19. Which sites are routinely swabbed as a part of MRSA **emergency** admission screening? (tick all that apply)

    Nose ☐
    Groin ☐
    Perineum ☐
    Axilla ☐
    Throat ☐
    CSU ☐
    Wounds, ulcers, sores ☐
    In situ devices (PEG, iv lines etc) ☐
    Other (please state) ………………………………………………………………

20. Which sites are routinely swabbed as a part of MRSA **elective** admission/pre-admission screening? (tick all that apply)

Nose ☐
Groin ☐
Perineum ☐
Axilla ☐
Throat ☐
CSU ☐
Wounds, ulcers, sores ☐
In situ devices (PEG, iv lines etc) ☐
Other (please state) ………………………………………………………………

Microbiological Testing

21. Are nose, groin/perineal swabs routinely pooled or tested separately? (tick one only)

Pooled ☐ Tested separately ☐

22. How much does your microbiology laboratory charge per swab for processing MRSA **admission** screens? (complete for all that apply)

PCR test £________________________________

Rapid enrichment £__________________________

Chromogenic Agar plating £___________________

Broth enrichment culture £____________________

Other £___________________________________

23. What is your routine microbiological test for **emergency** admissions? (tick one only)

PCR test ☐
Rapid enrichment and immunomagnetic/bioluminescent detection ☐
Chromogenic Agar plating (direct culture) ☐
Broth enrichment and agar subculture ☐
Other (please state) ………………………………………………………………

24. What is your routine microbiological test for **elective** admissions? (tick one only)

PCR test ☐
Rapid enrichment and immunomagnetic/bioluminescent detection ☐
Chromogenic Agar plating (direct culture) ☐
Broth enrichment and agar subculture ☐
Other (please state) ……………………………………………………………

25. Where are emergency and elective admission screens routinely processed?

In the trust ☐
In another trust ☐
In a private laboratory ☐

26. Do you routinely use PCR for ***emergency admission*** screens in the following areas? (*tick all that apply*)

Haematology/Oncology ☐
Nephrology ☐
Trauma & Orthopaedics ☐
Neurosurgery ☐
Cardiothoracic surgery ☐
Vascular surgery ☐
None of these ☐
Other (please state) ……………………………………………………………

27. Do you routinely use PCR for ***elective*** screens in the following areas? (*tick all that apply*)

Haematology/Oncology ☐
Nephrology ☐
Trauma & Orthopaedics ☐
Neurosurgery ☐
Cardiothoracic surgery ☐
Vascular surgery ☐
None of these ☐
Other (please state) ……………………………………………………………

28. If you use PCR tests for ***any*** admission screening:
Do you perform these? *(tick all that apply)*

Not applicable ☐
At the point of care ☐
In hot laboratory in your trust ☐
In a central laboratory in your trust ☐
In another trust ☐
In a private laboratory ☐

29. How often do you run PCR tests? (*complete all that apply*)

On weekdays ☐ *(state number of times per day)* ……………………………
At weekends ☐ *(state number of times per day)* ……………………………
As requested ☐
Other (please state) …………………………………………………………

Patient Management

30. Which patients routinely receive MRSA suppression/decolonisation following emergency admission screening *(**before result is known**)*? (*tick all that apply*)

All patients ☐
All previous MRSA positive patients ☐
All other patients in "high risk" categories (according to local definition) ☐
All patients in "high risk" wards (according to local definition) ☐
None ☐
Other (please state)…………………………………………………………………

31. Which MRSA +ve patients receive MRSA suppression/decolonisation **before admission** following pre-admission screening? (*tick all that apply*)

All patients ☐
All patients in "high risk" categories (according to local definition) ☐
All patients in "high risk" wards (according to local definition) ☐
None ☐
Other (please state)…………………………………………………………………

32. What is your **first line** suppression/decolonisation regime for MRSA patients? (*tick all that apply*)

Mupirocin (nasal) ☐
Chlorhexidine gargle ☐

Chlorhexidine bodywash ☐

Neomycin/chlorhexidine (nasal) ☐

Benzalkonium/chlorhexidine (i.e. Dermol 500®) ☐

Octenidine HCl (i.e. Octenisan®) ☐

Triclosan (ie Skinsan®) ☐

Other (please state) ……………………………………………………………

33. In which patients are contact precautions (gloves and aprons) routinely commenced **before** screening results are available?

All patients ☐

All previous MRSA +ve patients ☐

All *other* patients in "high risk" categories (according to local definition) ☐

None ☐

Other (please state)………………………………………………………………

34. Which patients are routinely isolated **before** screening results are available?

All patients ☐

All previous MRSA positive patients ☐

All *other* patients in "high risk" categories (according to local definition) ☐

All patients in "high risk" wards (according to local definition) ☐

None ☐

Other (please state)………………………………………………………………

Compliance with MRSA Screening

35. What was your most recent reported compliance for **emergency** screening to the SHA or Monitor?…………….%

36. What was your most recent reported compliance for **elective** screening to SHA or Monitor?……………...%

37. How do you calculate this?

Divide combined number of MRSA screens sent to lab over a given time period by total admissions in the same period ☐

Divide combined number of MRSA screens sent to lab over a given time period by total discharges in the same period ☐

Link MRSA screens sent to lab with patient level elective, emergency and day-case ***admissions*** to hospital ☐

Link MRSA screens sent to lab with patient level elective, emergency and day-case ***discharges*** from hospital ☐

Point prevalence audit ☐

Other (please state)…………………………………………………………………

SECTION 3: MRSA POINT PREVALENCE AUDIT (11th MAY 2011)

Instructions

This section should be completed at midday on Weds 11th May 2011. If it is not possible to do it on that date please complete the form on another day in that week.

38. How many inpatients in the trust today?...

Wards

39. How many inpatient wards does your trust have?...

40. How many wards for the following specialties does your trust have?

    Haem/Onc.................................

    Nephrology................................

    Trauma/Orthopaedics................

    Neurosurgery............................

    Cardiothoracic surgery.............

    Vascular surgery.......................

Isolation Capacity

41. How many side rooms are there in total in the trust?.......................................

42. How many side rooms on maternity wards?...

43. How many side rooms on paediatric wards?...

44. How many siderooms on ICU/HDU wards?...

45. How many beds are on a currently used and designated MRSA ward or on an isolation ward to which MRSA patients can be admitted? ....

MRSA +ve Patients

46. How many MRSA +ve patients are there in total in the trust today?................

47. How many MRSA +ve patients are there in ICU/HDU today?...........................

48. How many MRSA +ve patients are isolated in a sideroom today?...................

49. How many MRSA +ve patients are isolated on a currently used and designated MRSA/isolation ward today?...

50. How many MRSA +ve patients are isolated in cohort bay on a general ward today?..

51. How many MRSA +ve patients **are not** isolated today?.................................

Antibiotics

52. How many of the MRSA +ve patients are currently receiving one or more antibiotic for MRSA infection?
(e.g. *vancomycin, teicoplanin, doxycycline, linezolid, rifampicin, fusidic acid*)

53. If you are unable to provide the data requested in question 52 please state why...
....
....
....
....
....
....

SECTION 4:

INDIVIDUAL PATIENT AUDIT (MRSA +ve Patients)
(For the week of 09/05/2011 – 15/05/2011)

In this section we ask you to fill out a separate form for each patient.

Fill out a form for **each** patient (adult and paediatric) who meets **all three** of the following criteria:

1. MRSA admission or pre-admission swab taken between 00:01hrs on Monday **09/05/2011** and 23:59hrs on Sunday **15/05/2011**.
2. MRSA positive.
3. Never previously known to be MRSA positive.

Do **not** include;

- Elective patients admitted during that week, but screened in a pre-admission clinic *in a previous week.*
- Patients who were **not** screened as part of the admission screening (i.e. rescreens and clinical screens).
- Emergency and elective **admissions** that were not screened by the beginning of day 3 of admission

**PATIENT DETAILS**		**MRSA +VE PATIENTS**	**Patient 1**
1. Patient Gender?	Female ☐ Male ☐	2. Age?	
3. Admission date? (*leave blank if patient not yet admitted*)	**dd/mm/2011**	4. Discharge date? (*leave blank if patient not yet discharged*)	**dd/mm/2011**
**SAMPLE DETAILS**			
5. Date swab taken?	**dd/mm/2011**	6. Date result first available to ICT?	**dd/mm/2011**
7. Why was specimen taken?<br>(tick **ONE** only, include only swabs taken by the beginning of day 3 of admission)		☐ Pre-admission screen (**elective**) in outpatients/ pre-admission<br>☐ Elective **on admission** screen<br>☐ **Emergency** on admission screen	
**HOSPITAL DETAILS**			
8. Main consultant specialty for this admission?	Haem/Onc ☐ Nephrology ☐ Trauma & Orthopaedics ☐<br>Neurosurgery ☐ Cardiothoracic surgery ☐ Vascular surgery ☐<br>Other medical ☐ Other surgical ☐		
9. Ward specialty when screen taken?	ICU/HDU ☐<br>MAU ☐ SAU ☐<br>A&E ☐ Other ☐<br>Not admitted ☐	10. Was patient admitted as a day-case?	Yes ☐ No ☐<br>Not yet admitted ☐
**POTENTIAL RISK FACTORS FOR MRSA ACQUISITION**			
11. Was the patient admitted from a residential or nursing home?			Yes ☐ No☐<br>Don't know ☐
12. Was the patient admitted directly from another trust on this admission?			Yes ☐ No☐<br>Don't know ☐
13. Has the patient been an inpatient within last year in **this** trust?			Yes ☐ No☐<br>Don't know ☐
14. Has the patient been an inpatient within last year **another** trust?			Yes ☐ No☐<br>Don't know ☐
15. Did patient have any wounds, ulcers, pressure sores etc. **at the time of screening**?			Yes ☐ No☐<br>Don't know ☐
16. Did the patient have any IV lines, catheters, PEGs or other in situ devices **at the time of screening?** *(Not including any devices inserted on or since admission)*			Yes ☐ No☐<br>Don't know ☐
**HOW WAS PATIENT MANAGED?**			
17. Was patient an inpatient when result available?		Yes ☐ Not yet admitted ☐ Discharged ☐<br>Don't Know ☐	
18. Was patient isolated **before** result available?		Yes ☐ No ☐ Don't know ☐<br>Not yet admitted ☐	
19. If **yes** was patient isolated in:		Sideroom ☐ Cohort bay on ward ☐<br>MRSA ward☐ Isolation ward☐	
20. Was patient isolated **after** result available?		Yes ☐ No ☐ Don't Know ☐<br>Not yet admitted ☐	
21. If yes was patient isolated in:		Sideroom ☐ Cohort bay on ward☐<br>MRSA ward ☐ Isolation ward☐	
22. Was suppression/decolonisation started/advised? *(Include inpatients, discharged patients & those not yet admitted)*		Yes, **before** result available ☐ No ☐<br>Yes, **after** result available ☐ Don't know ☐	

SECTION 5:
INDIVIDUAL PATIENT AUDIT (MRSA -ve Patients)
(For the week of 09/05/2011 – 15/05/2011)

In this section we ask you to fill out a separate form for each patient.

Fill out a separate form for a random sample of **5-10** patients (adult and paediatric) who meet the following criteria

1. Patients screened on admission/preadmission *or* admitted between 00:01hrs on Monday **09/05/2011** and 23:59hrs on Sunday **15/05/2011**.
2. MRSA negative.

Do **not** include:

- Patients who were **not** screened as part of the admission screening (i.e. rescreens and clinical screens).
- Emergency and elective **admissions** that were not screened by the beginning of day 3 of admission

Further instructions on how to select your sample are included below.

Selecting MRSA negative patients

We ask you to randomly identify 5-10 patients who were found to be MRSA negative on admission or following a pre-admission screen and to complete a form for each of these patients.

It is important that the patients are chosen randomly to prevent bias. We explain the protocol for doing this below. We have given two options for doing this:

- Option A – randomising by screen date.
- Option B – randomising by admission date.

You can use whichever you find easiest.

Option A: Randomisation by Screen Date

STEP 1: Identify all MRSA admission, and pre-admission screens that were logged on Wednesday 11th May.

STEP 2: Number each screen consecutively on a master list.

STEP 3: Identify a sample of 5-10 using the online research randomiser tool. (http://www.randomizer.org/form.htm)

USING THE ONLINE RANDOMISER

Please fill in all the required fields as follows:

1) *How many sets of numbers do you want to generate*? **1**

2) *How many numbers per set*? **5** or **10** (depending on whether you are selecting 5 or 10 patients).

3) *Number range (e.g., 1-50) From:* **1 To**: **enter the total number of patients identified on your master list.**

4) Do you wish each number in a set to remain unique? **Yes**

5) Do you wish to sort your outputted numbers? **Yes least to greatest**

6) How do you wish to view your outputted numbers? **Place markers off**

Click on the **Randomize Now!** button. You will now see a list of 5 or 10 random numbers.

STEP 4: Match these numbers to your master list to identify 5-10 patients. And complete a form for each of these patients.

STEP 5: When results are available check which of the randomised patients have a result confirming that they are MRSA negative. If you have completed a form for a patient who is found to be MRSA positive or whose result is not available, please put a line through the form.

Option B: Randomisation by Admission Date

STEP 1: Identify all admissions (elective and emergency) for Wednesday 11th May.

STEP 2: Number each screen consecutively on a master list.

STEP 3: Identify a sample of 5-10 using the online research randomiser tool. (http://www.randomizer.org/form.htm)

USING THE ONLINE RANDOMISER

Please fill in all the required fields as follows:

1) *How many sets of numbers do you want to generate*? **1**

2) *How many numbers per set*? **5** or **10** (depending on whether you are selecting 5 or 10 patients).

3) *Number range (e.g., 1-50) From:* **1 To**: **enter the total number of patients identified on your master list.**

4) Do you wish each number in a set to remain unique? **Yes**

5) Do you wish to sort your outputted numbers? **Yes least to greatest**

6) How do you wish to view your outputted numbers? **Place markers off**

Click on the **Randomize Now!** button. You will now see a list of 5 or 10 random numbers.

STEP 4: Match these numbers to your master list to identify 5-10 patients. And complete a form for each of these patients.

STEP 5: On Friday 13th May (or when the results become available) check which of the randomised patients have a result confirming that they are MRSA negative. If you have completed a form for a patient who is found to be MRSA positive or whose result is not available, please put a line through the form.

PATIENT DETAILS MRSA -VE PATIENTS			Patient 1
1. Patient Gender?	Female ☐ Male ☐	2. Age?	
3. Admission date? (*leave blank if patient not yet admitted*)	**dd/mm/2011**	4. Discharge date? (*leave blank if patient not yet discharged*)	**dd/mm/2011**
**SAMPLE DETAILS**			
5. Date swab taken?	**dd/mm/2011**	6. Date result first available to ICT?	**dd/mm/2011**
7. Why was specimen taken? (tick **ONE** only, include only swabs taken by the beginning of day 3 of admission)		☐ Pre-admission screen (**elective**) in outpatients/ pre-admission<br>☐ Elective **on admission** screen<br>☐ **Emergency** on admission screen	
**HOSPITAL DETAILS**			
8. Main consultant specialty for this admission?	Haem/Onc ☐ Nephrology ☐ Trauma & Orthopaedics ☐<br>Neurosurgery ☐ Cardiothoracic surgery ☐ Vascular surgery ☐<br>Other medical ☐ Other surgical ☐		
9. Ward specialty when screen taken?	ICU/HDU ☐<br>MAU ☐ SAU ☐<br>A&E ☐ Other ☐<br>Not admitted ☐	10. Was patient admitted as a day-case?	Yes ☐ No ☐<br>Not yet admitted ☐
**POTENTIAL RISK FACTORS FOR MRSA ACQUISITION**			
11. Was the patient admitted from a residential or nursing home?			Yes ☐ No☐<br>Don't know ☐
12. Was the patient admitted directly from another trust on this admission?			Yes ☐ No☐<br>Don't know ☐
13. Has the patient been an inpatient within last year in **this** trust?			Yes ☐ No☐<br>Don't know ☐
14. Has the patient been an inpatient within last year **another** trust?			Yes ☐ No☐<br>Don't know ☐
15. Did patient have any wounds, ulcers, pressure sores etc. **at the time of screening**?			Yes ☐ No☐<br>Don't know ☐
16. Did the patient have any IV lines, catheters, PEGs or other in situ devices **at the time of screening?** *(Not including any devices inserted on or since admission)*			Yes ☐ No☐<br>Don't know ☐
16a. Was patient ever previously known to be MRSA positive?			Yes ☐ No☐<br>Don't know ☐
**HOW WAS PATIENT MANAGED?**			
17. Was patient an inpatient when result available?		Yes ☐ Not yet admitted ☐ Discharged ☐<br>Don't Know ☐	
18. Was patient isolated **before** result available?		Yes ☐ No ☐ Don't know ☐<br>Not yet admitted ☐	
19. If **yes** was patient isolated in:		Sideroom ☐ Cohort bay on ward ☐<br>MRSA ward☐ Isolation ward☐	
22. Was suppression/decolonisation started/advised? *(Include inpatients, discharged patients & those not yet admitted*)		Yes, **before** result available ☐ No ☐<br>Yes, **after** result available ☐ Don't know ☐	

ACKNOWLEDGEMENTS

Many thanks to Infection Control Teams from the following Trusts for help with the piloting of the questionnaire.

Royal Free Hospital NHS Trust
Barnet and Chase Farm Hospitals NHS Trust
University College London Hospitals NHS Trust
Brighton
Royal United Hospital Bath NHS Trust
Whittington Hospital NHS Trust
Mid Essex Hospitals NHS Trust
Harrogate and District Hospitals NHS Trust

When complete please return this questionnaire in the pre-paid envelope by **20th May 2011** to:

Christopher Fuller
UCL Research Department of Infection and Population Health
UCL Hampstead Campus
Royal Free Hospital
London
NW3 2PF

christopher.fuller@ucl.ac.uk

Appendix 3. Methodology: modelling.

Introduction

In order to address objective 6, the above data were used to inform mathematical models of MRSA transmission in hospital populations. Three models were developed to represent different Trust types; Acute, Teaching and Specialist. Each of the models simulated patient movement within the hospital and between hospital and community populations, transmission of MRSA within the hospital, as well as alternative screening and control strategies.

The screening strategies evaluated using each model were:

1. no screening,
2. routine screening all Elective and Emergency admissions,
3. screening only admissions to "high-risk" specialties,
4. patient level risk-based screening of all admissions (checklist activated screening {CAS]),
5. screening admissions to "high-risk" specialties plus checklist activated screening of admissions to "low-risk" specialties,
6. routine screening of all admissions with pre-emptive isolation of those known to be previously MRSA positive.

The models were used to simulate MRSA transmission under each of these alternative strategies in order to compare both the effectiveness and cost-effectiveness of control.

The models

An existing dynamic model developed for the DH funded MECAMIP project [Robotham et al 2011], which simulated MRSA transmission within a single hospital ward, was further advanced such that screening and control policies could be evaluated at the whole hospital level, including internal hospital structure and patient journeys through it.

Model Extensions

In order for the models to be appropriate for evaluation of hospital-level screening policies the following major extensions were performed:

5. The development of a whole hospital model comprising a specialty-level structure. This involved the inclusion of two specialty groups within one model and therefore required

parameterisation at a specialty level, as well as a whole hospital level, allowing, for example, discharge and death rates to differ between specialties - as well as to reflect whole hospital patterns.

6. The inclusion of realistic patient movements. This involved simulation of patient movement in and out of hospital, as well as between specialties, and inclusion of the readmission process (see 'Patient Movements').
7. Stratification of the admission process such that admissions were classified as Elective or Emergency. This distinction was important due to the differences in proportion of high-risk patients and proportion of colonised patients admitted in these two ways. Additionally, this model development could allow more flexibility in policy evaluation at a later stage e.g. questions such as 'is screening Elective or Emergency admissions a better use of limited resources?' can be addressed.
8. The development of three distinct models representing the different Trust types: defined by Trust size, distribution of high risk and low risk specialties, numbers of isolation beds, Trust specific prevalence on admission, proportions of patients admitted via elective and emergency routes, proportion of patients classified as having a risk factor for MRSA (via the checklist) and proportion of MRSA positive patients who were previously known to be positive.

Model structure

Briefly, the models were stochastic, individual-based and discrete-time and simulated the transmission of MRSA in a whole hospital setting. The stochastic nature of the models allowed chance effects to be considered, as they are likely to play an important role in the transmission dynamics in small populations such as a hospital. The models were individual-based allowing the MRSA status of individual patients to be tracked over time, as well as other individual characteristics such as any interventions they were undergoing and how likely they were to acquire colonisation/infection and their individual level of transmissibility. To realistically capture the effect of using imperfect screening tests both the true and perceived MRSA status of each patient was explicitly tracked.

<u>The transmission process</u>

Transmission was modelled by classifying patients into three states: susceptible (i.e. MRSA negative), MRSA colonised or MRSA infected. Patients could move between these states according to defined daily probabilities. The models used a time step of one day and were dynamic in the sense that the

number of colonised, infected and susceptible patients on each day depended (stochastically) on the number from the previous day. Infections could occur via two routes, a patient may either acquire infection through cross-transmission, or (more commonly) due to a progression or 'self-infection' event, where the patient is already colonised with MRSA and progresses to a clinically infected state. We assumed that the instantaneous risk of a susceptible patient becoming colonised or infected with MRSA through cross-infection increased linearly with the number of other MRSA positive patients in the ward.

Patient movements

Length of stay data were used directly to model patient movements in and out of the different specialties and from the hospital as a whole, using daily probabilities of discharge and death (see Parameterisation section for full description of the data). The specialty level daily probabilities of discharge and death were adjusted for infection status according to the method as applied in MECAMIP [Robotham et al 2011] from the approach developed by Barnett et al (2009) such that infected patients, on average, had longer stays in hospital and greater risk of death.

If a specialty-level discharge was scheduled, the probability of the discharged patient being transferred to another specialty, rather than being discharged fully from hospital, was calculated. If the patient was to be transferred they were placed in a transfer queue until a bed became available. Until the transfer occurred the patient remained in their original specialty and could be discharged at any point. If a transfer did not occur then the patient was discharged from the hospital into the community population. In this case, the probability of that patient being readmitted and the duration of time between discharge and readmission were calculated, and a readmission was scheduled at some point in the future if appropriate.

Under an assumption of 100% bed occupancy, each discharge or death event triggered an admission to the freed bed, with first preference given to a patient awaiting a transfer, second preference given to a patient scheduled for readmission at that time, or lastly, a new admission was randomly selected from the community population.

Interventions

Screening was performed using a chromogenic agar test (test characteristics described in 'Parameters') on patient admission to hospital. A number of different scenarios of who to screen were assessed: 1) no patients on admission, only clinical specimens of those patients suspected to have clinical infection during their stay; 2) all patients on admission; 3) admissions to only high risk

specialties; 4) admissions who were considered at risk of colonisation through checklist activated screening (‘checklist positive’); or 5) a combination of these.

Screening allowed identification of colonised or infected patients. Due to the inaccuracies in the screening tests, false negative results could lead to a patient’s actual state being either colonised or infected, whilst their believed state was susceptible, which (depending on the policy) had the potential result in no control measures being imposed upon them. Similarly false positives, whilst unlikely to result in increased transmission, could cause unnecessary resource use.

Once a patient was found MRSA positive, they were assumed to remain positive until three further negative screens had been taken, at which time their believed state was taken to be MRSA negative and any control measures that had been imposed were removed.

Alongside screening, control measures were modelled. Isolation in side rooms and decolonisation (with chlorhexidine and mupirocin) was the primary method of control. However, as side rooms were limited, once capacity was reached those patients identified as MRSA positive were placed under a secondary control method in which they were cohorted in the open ward with standard contact precautions (where healthcare workers used disposable aprons and gloves prior to patient contact) and decolonisation. Both intervention methods reduced the degree of transmissibility of patients undergoing them, but with different degrees of effectiveness (see table 7 appendix 4). A queuing system was simulated such that as soon as space became available in isolation rooms, due to either: a discharge, death or believed recovery event, a known MRSA positive patient undergoing secondary control were moved into the isolation room.

The model structure is represented schematically in Figure D1, and model assumptions listed below.

Figure D1. Schematic of whole-hospital and community transmission model structure. S=susceptible, or MRSA negative, patient; C=colonised; I = infected. Black lines represent movements, where solid lines are admissions and discharges, dashed lines readmissions and dotted line transfers. Red lines represent transmission events, and green lines death events.

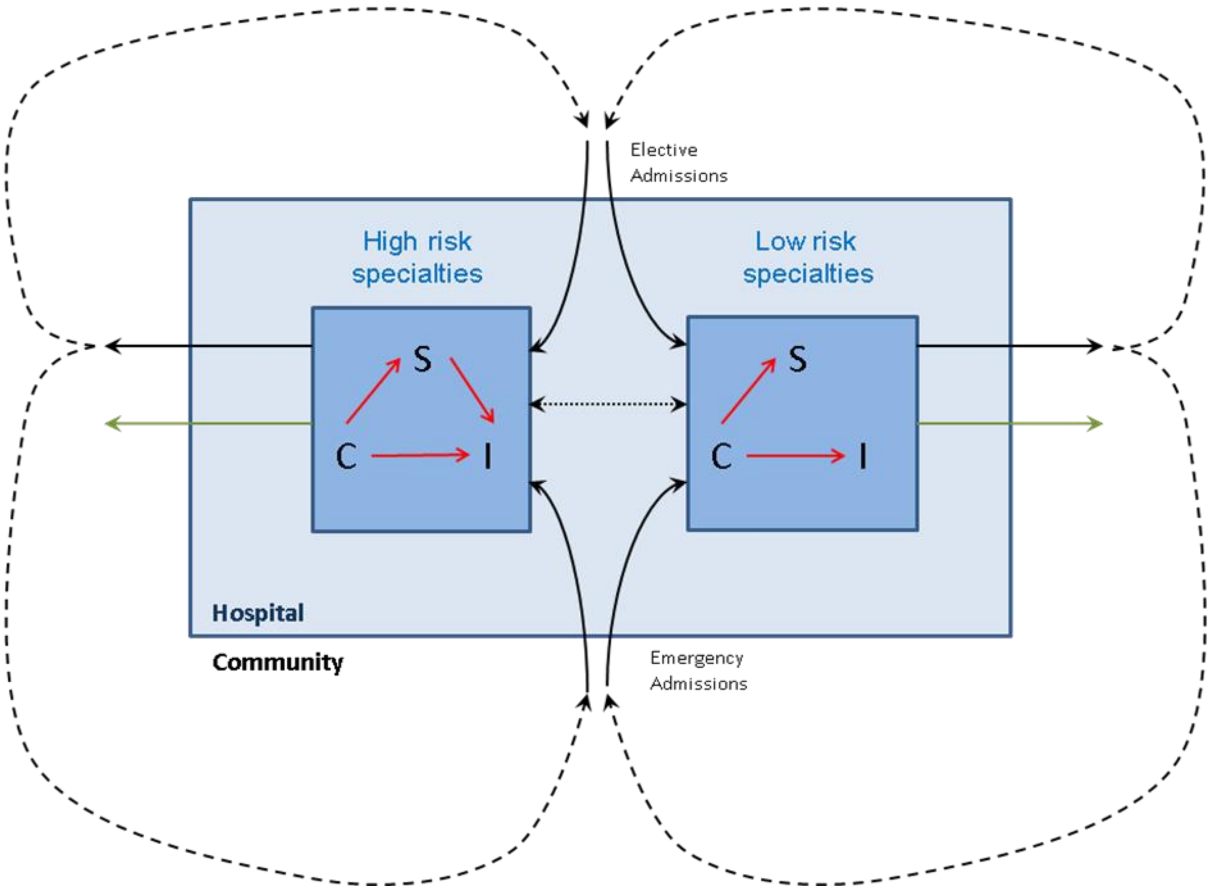

Summary of model assumptions:

- Admissions may be colonised or susceptible according to prevalence (but not infected).
- Prevalence on admission is dependent on whether the patient is categorised as at risk of MRSA colonisation (determined by risk-factor analysis i.e. the checklist), as well as whether they are admitted via an Elective or Emergency admission route
- No specific assumptions about transmission routes are made.
  - The instantaneous risk of a susceptible patient becoming colonised increased linearly with the ward-level MRSA prevalence.
- Colonised and infected patients are equally infectious.

- Transmission parameters (infectiousness of colonised/infected individuals, probability of progression and susceptibility to colonisation/infection) are specialty dependent.
- Direct infection from a susceptible state cannot occur in low risk specialty settings and patients must first become colonised.
- Once MRSA positive, patients remain so for the duration of their stay.
- All infected patients are suspected to be so, with a delay of 1 day before a clinical specimen is taken.
- Recovery may occur in the community.
- At any time patients may belong to either high-risk (HR) or low-risk (LR) specialties
  - Parameters may differ between specialties
  - No transmission can occur between specialties.
- Length of stay of colonised and susceptible patients is modelled using the same daily probabilities of discharge, only infection increases length of stay
- Similarly, additional mortality is associated with infection only
- Daily probability of discharge and death is dependent on whether the patient is in a high-risk or low-risk specialty as well as their infection status.
- 100% bed occupancy.

Uncertainty

For parameters determining effectiveness of the intervention method used, values were defined as probability distributions rather than point estimates. These distributions were chosen to represent the uncertainty in each of the parameters and are assumed independent. In each model simulation run, a parameter value was sampled from these distributions. Since different simulations draw different parameter values from these distributions, the model outcomes also vary between simulations. In this way, uncertainty could be propagated through the model.

In addition to the uncertainty in the parameter values, chance also enters the model due to the stochastic nature of the transmission process. While it is important to account for such stochastic effects when evaluating different strategies, uncertainty in model outcomes should reflect only parameter and structural uncertainties in the models. Therefore, for each sample of parameter values we performed a large number of runs and recorded the mean value of the outcomes of interest. Specifically, we selected 50 parameter sets (each with a different value pulled from the probability distribution for intervention effectiveness) and ran the model 1000 times for each parameter set. Therefore for each strategy, we performed a total of 50,000 model runs.

The model was programmed using the C++ language and open-source libraries. As hundreds of thousands of model runs were required to compare the strategy options; these were performed on a high performance cluster. Analysis and graphical representation of the large amount of generated output was performed in R 2.10.1 (www.r-project.org).

Cost-effectiveness analysis

Health economic data were incorporated into the model to explore the direction and size of changes in economic costs and health benefits due to interventions, through a cost-effectiveness analysis (Graves et al, 2004), allowing comparison of each screening policy. Incorporation of economic parameters into a transmission dynamic model (as opposed to a static model) allows population-level effects to be accounted for, such effects are important since preventing infection in one individual directly benefits that individual and indirectly benefits others by preventing transmission. Analyses failing to take account of indirect effects may underestimate benefits of interventions (Roberts et al 2004). Health benefits are described using quality adjusted life years (QALYs). The theory by which health benefits may be evaluated using a dynamic simulation process is outlined in Figure D2.

Figure D2: Representation of how a transmission dynamic model can evaluate the impact of an intervention policy on health benefits (measured in QALY).

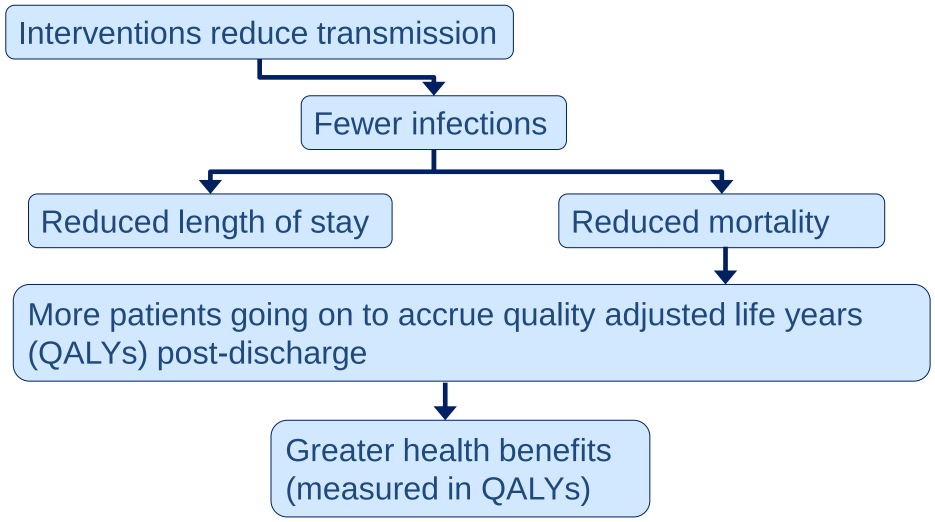

Costs

The transmission dynamic model is used to estimate the number of occurrences (over the simulation period) of each event that incurs costs. Important parameters are the monetary valuation for these events and of associated resources, expressed as unit costs.

Essentially, three types of costs incurred were considered in our analyses:

- Infection related costs each day whilst an individual is infected
- Cost of a bed day
- Intervention related costs determined by the particular strategy under consideration e.g. number of screens taken

Health Benefits

For estimating the total health benefits per admission (in terms of quality adjusted life years) a life year accrued by a patient following hospital discharge is the dominating factor. Therefore, an intervention which reduces the number of deaths and increased the number of successful patient episodes would result in a higher life expectancy and greater health benefits. Health benefits gained while in the hospital represent a very minor adjustment to this, but for completeness were considered in our analysis.

Therefore three measurements were required for analysis of health outcomes:

- bed days accrued
- number of deaths
- number of successful patient episodes (number of patients discharged alive)

Quality adjusted life years accrued following hospital discharge were taken from the MECAMIP report [Robotham et al 2011], where age and sex matched survival rates were adjusted for quality and discounted at a rate of 5% to give an average quality adjusted life expectancy for a discharged general patient (10 years).

For this evaluation, this estimate was further adjusted to account for long term effects brought about through long term detriment to quality of life in a proportion of MRSA infections.
The proportion of patients who acquired an infection during their hospital stay was calculated. Assuming that 2.4% of those patients with infections have long-term health loss amounting to a

further reduction of 30% (estimates from the Pathfinder study (Smith et al 2011), we then calculated the proportion of discharged patients with no long term health effects, and the proportion discharged with long-term health effects and adjusted the average quality adjusted life expectance on discharge accordingly.

Cost-effectiveness outputs

We conduct a health-economic evaluation to predict outcomes of each strategy in terms of costs and health benefits, measured in Quality Adjusted Life Years (QALYs) (Drummond et al 2005). Competing interventions are compared against a baseline scenario in terms of their incremental cost-effectiveness ratios (ICER) and their net monetary benefits (NMBs). ICERS are given by the ratio of the change in costs to the change in health outcomes (QALYs) compared to the alternative.

The ratio is interpreted in light of a decision-maker's maximum willingness to pay for a unit of health outcome, such as a QALY. Strategies are considered cost-effective if they generate an ICER) that is less than the current NHS decision makers willingness to pay threshold of £30,000 per QALY [Rawlins et al 2004].

The information used for the ICER can be re-arranged to estimate net monetary benefits (NMB), which are given by $\Delta E \text{ x}\lambda\text{-}\Delta C$. Where Δ is the difference in health benefits between the alternative and baseline strategy, λ the willingness to pay for health benefits, and ΔC the difference in costs between the alternative and baseline strategy. Therefore the NMB represent the value of the health benefits gained minus the amount the amount paid to achieve them.

The perspective for this analysis represents the healthcare decision maker who manages resources at a regional or national level, rather than the perspective of a manager of a single medical ward or hospital. We aim to represent the preferences of high level policy makers who seek to improve the economic efficiency of health care services.

Using the economic transmission model we compare policies in different scenarios and settings, firstly in terms of the clinical effectiveness of each policy (in terms of appropriateness of resource use, and number of transmission, infection and death events) followed by the costs of each policy. These effectiveness and costs results are then combined and depicted on cost-effectiveness planes and presented as mean ICERs, allowing direct comparison of alternative strategies. Together, these results are useful for understanding how and why costs and health benefits change with different

strategies. However, because these analyses ignore the uncertainty in model parameters the results are necessarily of limited value for directly informing policy decisions. Therefore, we also present results in the form of cost-effectiveness acceptability curves (CEACs) and cost-effectiveness acceptability frontiers (CEAFs) which show, respectively, the probability of each strategy having the highest NMB for different values of the willingness to pay for unit of health benefit gained, and the strategy returning the greatest expected NMB. The advantage of CEACs and CEAFs is that the decision is represented for a wide range of willingness to pay values. When a fixed value is assumed for the willingness to pay then the best decision according to the model is often considered the one that returns the highest expected NMB. Because these results account for what we believe to be fair representation of parameter uncertainty, they should be considered to be of more direct relevance for informing policy decisions. Therefore, decision makers should generally choose the strategy with the highest NMB, although because we do not know the model parameters with certainty, and because the best strategy depends on the values of these parameters, there is a chance that we will not choose the best strategy.

Appendix 4: Model Parameterisation

Introduction

Model parameters were estimated using data from the prevalence audit wherever possible, with others estimated as in MECAMIP (Robotham et al 2011) (from the literature, data analyses and formally elicited expert opinion). Where uncertainty associated with model parameters was included, parameters were described using probability distributions. Where NOW audit data were used for parameter estimation, data were stratified according to Trust type.

Model parameters can broadly be categorised into five main groups:

1. **Population parameters**, describing the characteristics and sizes of populations modelled.
2. **Movement parameters**, describing the movements of patients into, out of and between specialties, admissions and discharges to the hospital, the additional length of stay and mortality associated with MRSA infection, and probability of (and time until) readmission for discharged patients.
3. **Transmission parameters**, describing the susceptibility of individual patients to colonisation and infection, the infectiousness of colonised and infected patients, the probability of colonised patients progressing to an infected status, and the duration of colonisation and infection.
4. **Intervention parameters**, describing the characteristics and effectiveness of interventions.
5. **Health economic parameters**, detailing intervention related costs, infection related costs, and costs associated with additional stay due to infection as well as quality adjusted life expectancy post discharge.

All parameters are listed, along with their estimated value and source in Tables 1-5.

1. **Population parameters obtained from audit data (full tables of audit results and parameter estimations are given in Appendix 4a).**

Table 1.

**Parameter**	**Trust type (n values represent number of Trusts from which values were obtained, values in square brackets represent parameters for scenario analyses).**			Source/Notes
	**Acute**	**Teaching**	**Specialist**	
Hospital size	553<br>n=99	1113<br>n=22	152<br>n=18	Median numbers of occupied beds on the audit day (11th May 2011)
Number of high risk specialty beds (% of all beds)<br><br>Number of low risk specialty beds	89 (16%)<br><br>464<br>n=97	259 (23%)<br><br>854<br>n=22	67 (44%)<br><br>85<br>n=17	Median number of high risk specialty beds in trust on audit day<br>Median number of low risk specialty beds on audit day
Proportion of screened patients admitted via an:<br>Elective route<br><br><br>Emergency route	 0.464<br>n=87<br><br>0.536<br>n=88	 0.524<br>n=22<br><br>0.476<br>n=21	 0.822<br>n=22<br><br>0.178<br>n=21	 Total number of patients screened electively/total screens<br><br><br>Total no of patients screened as emergency/total screens
Proportion of patients classified as at risk for MRSA colonisation (or checklist positive) , admitted via:<br>Elective route<br><br><br><br><br><br>Emergency route	 <br>0.4377<br>n=87<br><br>[0.105]<br><br><br>0.5683<br>n=88<br><br>[0.245]	 <br>0.4364<br>n=22<br><br><br><br><br>0.5683<br>n=21	 <br>0.4381<br>n=18<br><br><br><br><br>0.5799<br>n=16	 <br>Total no of elective patients checklist positive/total no of patients checklist positive<br><br>Values obtained by applying the Scottish Pathfinder [Stewart et al 2011] definition of checklist positivity to the audit data.<br><br>Total no of emergency patients checklist positive/total no of patients checklist positive<br><br>Values obtained by applying the Scottish Pathfinder [Stewart et al 2011]<br>definition of checklist positivity to the audit data.
Proportion of colonised on admission:<br><br>Checklist negative patients	 <br>0.005<br>n=88<br><br>[0.0025, 0.01]	 <br>0.006<br>n=22<br><br>[0.003,0.012]	 <br>0.0048<br>n=18<br><br>[0.0024, 0.0096]	 <br>Total number of MRSA positive checklist negative patients/total no of checklist negative patients<br><br>Assumptions for low and high prevalence setting scenario analyses (half the audit value, and twice the audit value).

Checklist positive patients	[0.0055]<br><br>0.026<br>n-=88<br><br>[0.013, 0.052]<br><br>[0.0556]	0.0198<br>n=22<br><br>[0.0099, 0.0396]	0.0169<br>n=18<br><br>[0.00845, 0.0338]	Value for scenario analysis comparing NOW audit definition of checklist positivity with the Pathfinder [Stewart et al 2011] definition: obtained by applying the Pathfinder definition to the audit data.<br><br>Total number of MRSA positive checklist positive patients/total no of checklist positive patients<br><br>Assumptions for low and high prevalence setting scenario analyses (half the audit value, and twice the audit value).<br><br>Value for scenario analysis comparing NOW audit definition of checklist positivity with the Pathfinder [Stewart et al 2011] definition: obtained by applying the Pathfinder definition to the audit data.
Proportion MRSA positives previously known, admitted via:<br>Elective route	0.566<br>n=87	0.478<br>n=22	0.606<br>n=18	Total no of elective patients previously MRSA positive/total no of patients previously MRSA positive
Emergency route	0.546<br>n=88	0.483<br>n=21	0.196<br>n=16	Total no of emergency patients previously MRSA positive/total no of patients previously MRSA positive

0

2 Movement parameters

Daily probability of discharge or transfer

To parameterise and inform the model we analysed an anonymised dataset of all patients admitted to the Royal Free Hospital London between 29th October 2009 and 18th May 2011. Patient movement characteristics: daily probability of ward and subsequent hospital discharge, probability of changing specialty and probability of readmission, were calculated for high and low risk specialties (table 2).

In the dataset 2% of patients were admitted to a high risk specialty (Nephrology, Neurosurgery, Orthopaedics and Trauma, Haematology and Oncology, Vascular Surgery and Cardiothoracic Surgery) and accounted for 1,104 patient days. 98% of patients were admitted to a low risk specialty (General medicine, Geriatric medicine, Accident & Emergency, Cardiology, General surgery, Endocrinology, Hepatology, Neurology, Gastroenterology, Infectious diseases, Respiratory medicine, Rheumatology, Plastic surgery, Gynaecology, Urology, Anaesthetics, Hepatobiliary & Pancreatic surgery, Ophthalmology, Dermatology, Colorectal surgery, Ear, Nose) and accounted for 60,860 patient days.
As the proportion of patient days spent in an ICU was the same for high and low risk specialties (2.6% of patient days), we grouped ICU with non-ICU ward stays when calculating patient movement parameters. For each group of specialties we calculated the probability of discharge from the ward on their first, second and up to 22nd (and all subsequent) days of stay. The probability of ward discharge on day 1 for example, is calculated from the number of patients discharged from the ward on day 1, divided by the sum of patients discharged from the ward on day 1 and all subsequent days. Specialty dependent discharges from wards were adjusted to account for differences in lengths of stay between infection states according to expert opinion (as described in the MECAMIP report [Robotham et al 2011]), where experts believed that on average, daily probability of discharge for infected patients would be reduced by a mean of 25%. We assumed this adjustment applied across both high risk and low risk specialties.

We then calculated the proportion of patients who on discharge from the ward were subsequently discharged from the hospital for each day of stay. For those patients who

were discharged from the ward but not fully from the hospital, we calculated the daily probability that the patient would transfer from a high to low specialty (and vice versa). Overall discharge patterns were validated against data from the whole hospital (to ensure that using the specialty level estimates were accurately reflecting hospital level patient movements).

Furthermore, specialty dependent daily probabilities of ward discharge for susceptible/colonised patients derived from the individual-level data from the Royal Free hospital, and the adjusted values for daily probability of ward discharge for infected patients, were confirmed to provide comparable estimates to those used for the MECAMIP report (Robotham et al 2011) for general medical wards (Figure 1).

Figure 1. Daily probabilities of discharge by infection status for high and low risk specialties compared to those used in the MECAMIP model.

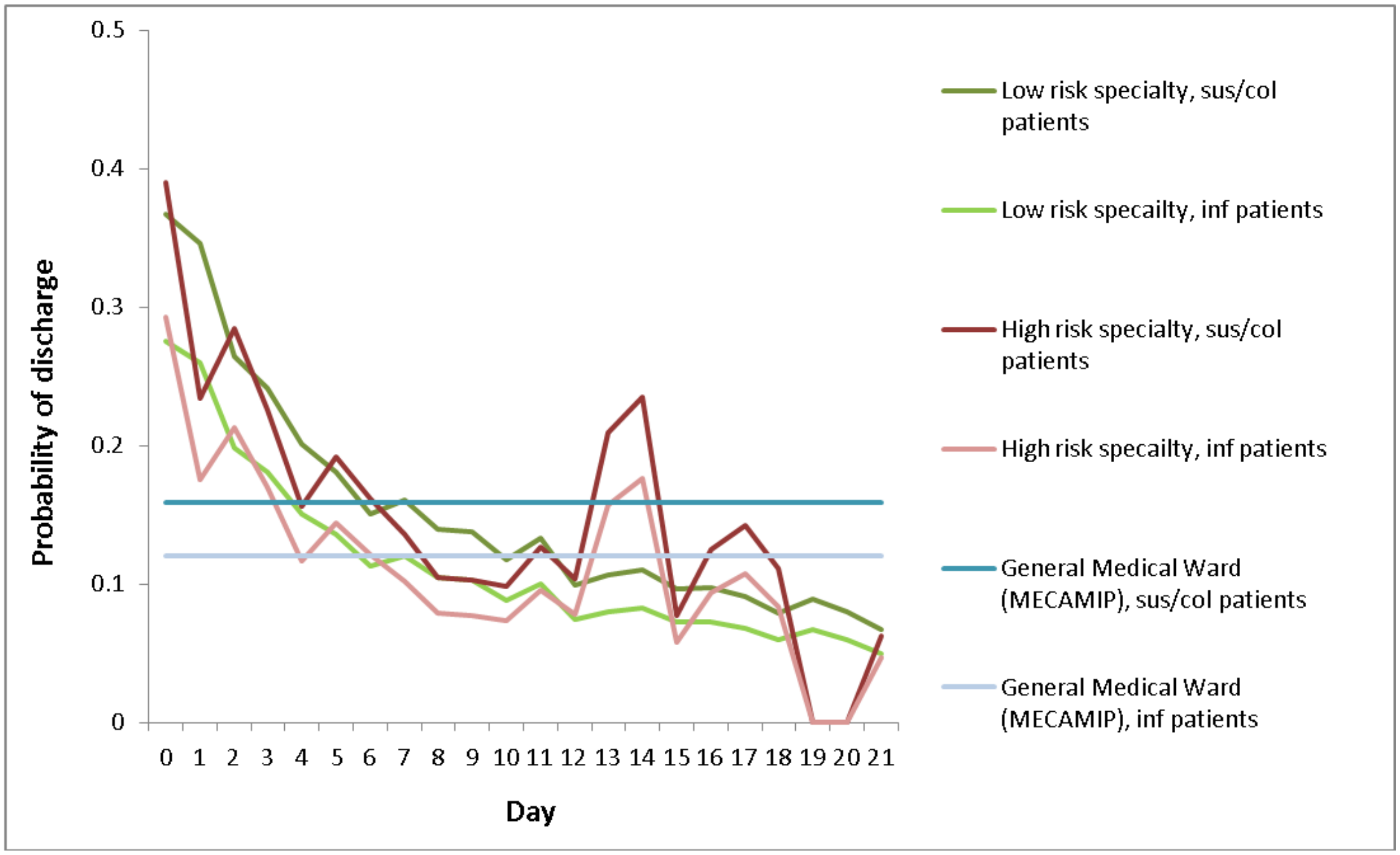

Table 2Daily probabilities of ward discharge (adjusted for infection status), hospital discharge, and specialty transfer calculated from individual level patient data.

Day	Low risk specialties				High risk specialties			
	Daily probability of discharge from ward (susceptible and colonised patients)	Daily probability of discharge from ward (infected patients)	Proportion of ward discharges discharged from the hospital	Proportion of ward discharges transferred to another speciality	Daily probability of discharge from ward (susceptible and colonised patients)	Daily probability of discharge from ward (infected patients)	Proportion of ward discharges discharged from the hospital	Proportion of ward discharges transferred to another speciality
0	0.367513	0.275635	0.621559	0.023384	0.389916	0.292437	0.41	0.31
1	0.346093	0.25957	0.368964	0.022564	0.23416	0.17562	0.35	0.38
2	0.264198	0.198149	0.417941	0.017045	0.284173	0.213129	0.37	0.40
3	0.241799	0.181349	0.459791	0.021591	0.226131	0.169598	0.29	0.53
4	0.200666	0.150499	0.466341	0.023766	0.155844	0.116883	0.46	0.38
5	0.180749	0.135562	0.501355	0.024457	0.192308	0.144231	0.36	0.31
6	0.150374	0.11278	0.518887	0.028926	0.161905	0.121429	0.35	0.73
7	0.16045	0.120338	0.552632	0.039216	0.136364	0.102273	0.50	0.50
8	0.139983	0.104987	0.473054	0.017045	0.105263	0.078947	0.50	0.75
9	0.137427	0.10307	0.475177	0.02027	0.102941	0.077206	0.57	0.33
10	0.118079	0.088559	0.521531	0.04	0.098361	0.07377	0.67	0.50
11	0.133248	0.099936	0.509615	0.009804	0.127273	0.095455	0.57	0.67
12	0.099039	0.074279	0.462687	0.027778	0.104167	0.078125	0.80	0.00
13	0.106645	0.079984	0.553846	0	0.209302	0.156977	0.22	0.57
14	0.110193	0.082645	0.633333	0.022727	0.235294	0.176471	0.63	0.33
15	0.097007	0.072755	0.542553	0.023256	0.076923	0.057692	0.50	1.00
16	0.097143	0.072857	0.552941	0.052632	0.125	0.09375	0.67	1.00
17	0.091139	0.068354	0.569444	0	0.142857	0.107143	0.67	0.00
18	0.079387	0.05954	0.561404	0	0.111111	0.083333	0.50	1.00
19	0.089259	0.066944	0.474576	0.032258	0	0	0.00	0.00
20	0.079734	0.059801	0.395833	0.034483	0	0	0.00	0.00
21	0.066787	0.05009	0.621622	0	0.0625	0.046875	0.00	0.00
22+	1	0.75	0	0	1	0.75	0.93	0.00

If ward discharge occurred, and given that a transfer event was chosen, the probability of which specialty patients were transferred to, given their specialty of discharge is presented in Table 3.

Table 3. Patient transfer events as proportions of which specialty they were transferred from.

		To	
		Low Risk	High Risk
From	Low Risk	0.93	0.07
	High Risk	0.96	0.04

Daily probability of death

We used an estimate of 0.007 for the daily probability of death for patients in low risk specialties. Using Dr. Foster data 2008/2009 (for East of England Trusts, from Eastern Region Public Health Observatory) the standardised mortality rate per 1000 discharges in general medicine was 44.7[1]. Taking a mean length of stay in general medicine of 6.3 days (Hospital Episode Statistics 2008/2009) gives a daily death probability of 0.007.

For comparison, in the MECAMIP report (Robotham et al 2011), daily risks of mortality estimated from intensive care unit data were adjusted according to expert opinion on the difference in mortality between ICUs and general medical wards. The mean daily probability of death in ICUs was 0.021. Adjusting this according to expert opinion (which gave a 65%

---

[1] Risk-adjusted hospital standardised mortality ratio is the rate calculated by Dr Foster Intelligence published as part of the Good Hospital Guide, by NHS Choices and accessed through some of DFIs on line tools. It is adjusted for age-sex, deprivation, case-mix, emergency admission and palliative care episodes and is based on about 80% of deaths in hospital. The methodology and risk ratios are available from the NHS Choices web site [

NHS website:
http://www.nhs.uk/NHSEngland/Hospitalmortalityrates/Documents/090424%20MS(H)%20-%20NHS%20Choices%20HSMR%20Publication%20-%20Presentation%20-20Annex%20C.pdf

NHS website:
http://www.nhs.uk/NHSEngland/Hospitalmortalityrates/Documents/HSMRLogodds_DH_Apr2009.xls
].

less chance of death in general medical wards compared to ICUs) gave a daily death probability of 0.0073.

For high risk specialties it was assumed that the daily probability of death was equivalent to that in ICUs, derived from robust individual-level data (as described in the MECAMIP report [Robotham et al 2011]), as these were considered to be the best available data on daily probabilities of death. However, as the mortality rate in ICUs may be higher than that in our high risk specialties, we also conducted a sensitivity analysis under much more conservative assumptions, where the daily probability of death in high risk specialities was equivalent to daily probability of death in low risk specialties.

Adjustment to the daily death probabilities according to infection status was carried out according to expert opinion (as in the MECAMIP report, where full details are provided [Robotham et al 2011]), where infected patients were considered on average 22% more likely to die on each day, than susceptible or colonised patients.

Table 4. Specialty dependent daily probabilities of death by infection status.

	Low risk specialty		High risk specialty	
	Susceptible/colonised	Infected	Susceptible/colonised	Infected
0	0.007	0.0085	0.026097	0.031246
1	0.007	0.0085	0.040642	0.05041
2	0.007	0.0085	0.027136	0.033747
3	0.007	0.0085	0.025827	0.032116
4	0.007	0.0085	0.024913	0.030864
5	0.007	0.0085	0.026675	0.032687
6	0.007	0.0085	0.017655	0.021574
7	0.007	0.0085	0.018846	0.023045
8	0.007	0.0085	0.018935	0.023017
9	0.007	0.0085	0.026315	0.031869
10	0.007	0.0085	0.013669	0.016561
11	0.007	0.0085	0.01637	0.019935
12	0.007	0.0085	0.023344	0.028224
13	0.007	0.0085	0.021429	0.025856
14	0.007	0.0085	0.022289	0.027009
15	0.007	0.0085	0.015979	0.019273
16	0.007	0.0085	0.01847	0.02239
17	0.007	0.0085	0.014736	0.017875
18	0.007	0.0085	0.012888	0.015681
19	0.007	0.0085	0.013874	0.01684
20	0.007	0.0085	0.027337	0.032801
21+	0.007	0.0085	0.012042	0.014536

<u>Readmission probabilities</u>

We assumed no difference in probability of readmission according to whether the patient was discharged from a high risk or low risk specialty. This assumption can be justified using the more detailed data collected from Sentinel Trusts, where the mean probability of readmission (to any specialty within 30 days of discharge) was 0.29 (CI: 0.19,0.68) for high risk specialty patients and 0.16 (CI: 0.07, 0.31) for low risk (from sample sizes of 468 and 2474 readmitted patients respectively).

Therefore overall readmission probabilities for the whole patient population were calculated from individual-level data from the Royal Free Hospital Trust (as described

above). Estimates of the probability of readmission, refined by number of previous hospital visits, are given in Table 5.

Table 5. Readmission probabilities given number of admissions within the past 365 days.

Probability of readmission within 365 days, after the:	
1st admission	0.21
2nd admission	0.6
3rd admission	0.5

The data were also interrogated to determine length of stay between discharge and subsequent admission, as shown in Figure 2.

Figure 2. The length of stay between discharge and subsequent admission

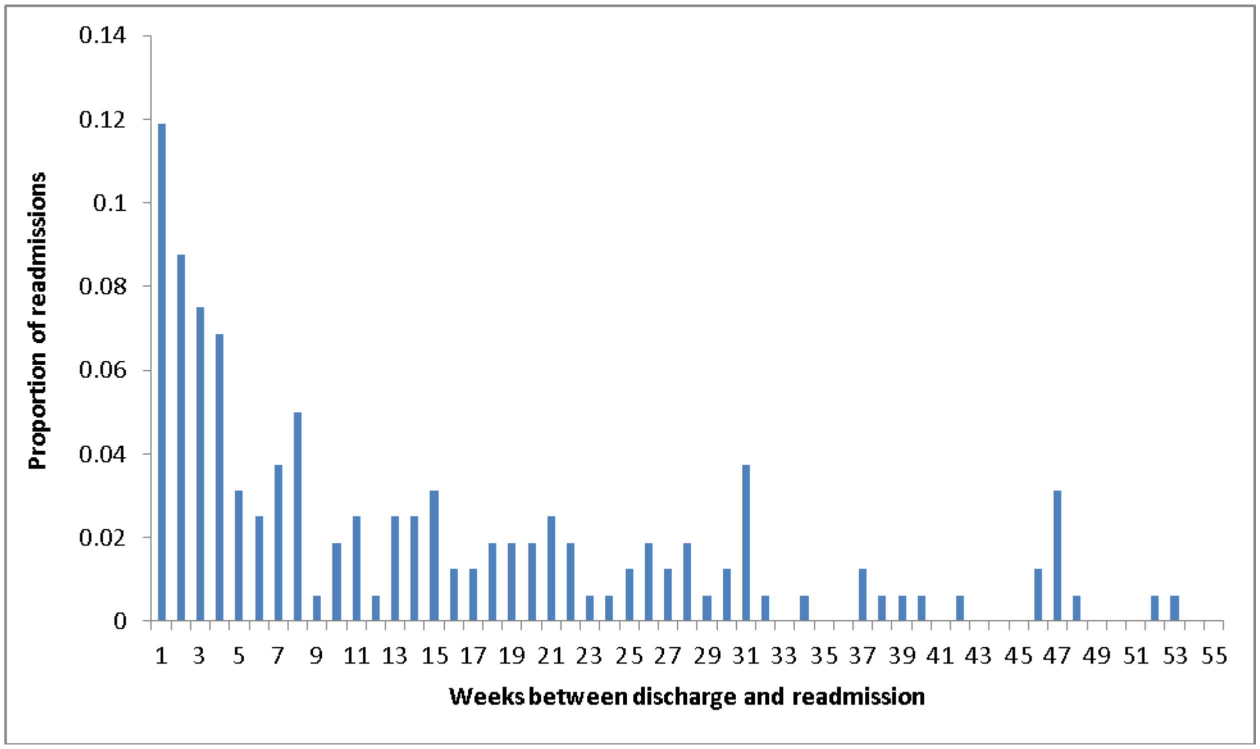

3. Transmission parameters

Transmission parameters (table 6) were taken from the MECAMIP report (Robotham et al 2011), as these were estimated using multi-state modelling techniques which analysed detailed individual-level patient data (for intensive care and general medical settings). These

estimates therefore represented the best available evidence for transmission in different settings, and provided sufficient level of detail needed for our individual-based models. We assumed transmission in high risk specialties to be equivalent to that in ICUs, and low risk specialties equivalent to that of hospital general wards. To take into account the possibility that transmission in high risk specialties may not be as high as that seen in intensive care, we conduct a sensitivity analysis where transmission in high risk specialties is assumed to be midway between the level of transmission seen in ICUs and that seen in general hospital wards.

Under the assumption that the risk of a susceptible patient becoming colonised increased linearly with the ward-level MRSA prevalence, and homogenous mixing, MECAMIP (Robotham et al 2011) transmission parameters were adjusted according to Trust dependent specialty sizes. For example, a doubling in the number of beds (i.e. patients) would reduce the probability of any given susceptible patient becoming colonized/infected by half (provided the number of infectious patients remained the same).

Table 6. Transmission parameters by trust and specialty type.

Trust type	Specialty type	Transmission Parameter (Values are means unless states otherwise. Values in square brackets represent those used in sensitivity analyses).			
		Daily probability of colonisation per source	Daily probability of infection (through transmission)per source	Daily probability of progression (from colonisation to infection)	Infectiousness
Acute	High risk	0.0004 [0.00025]	0.00007 [0.000035]	0.047 [0.0315]	1 [1]
	Low risk	0.0001	0	0.016	1
Teaching	High risk	0.00014	0.00002	0.047	1
	Low risk	0.00005	0	0.016	1
Specialist	High risk	0.00055	0.0001	0.047	1
	Low risk	0.0005	0	0.016	1

4. Intervention parameters

Table 7. Intervention parameters

Intervention	Parameter	Value	Source
Screening	Sensitivity	82.55	MECAMIP report [Robotham et al 2011]
	Specificity	83.05	
	Turn-around time	3 days	
	Delay between infection and clinical specimen being taken	1 day	
	Number of negative screens before considered negative	3	
Primary (isolation and decolonisation)	Side room capacity	Acute: 114<br>Teaching: 243<br>Specialist: 50	Audit data, using median number of wards for Trust type and assuming 5 isolation beds per ward (from Robotham et al 2011).
	Effectiveness in terms of reduction in transmissibility (mean (SD))	0.64 (0.14)	Worby et al (in submission).
Secondary (cohorting , contact precautions and decolonisation)	Effectiveness in terms of reduction in transmissibility (mean (SD))	0.24 (0.12)	MECAMIP report [Robotham et al 2011]

5. Health economic parameters

Costs used for the MECAMIP report (Robotham et al 2011) were updated in accordance with Pathfinder cost estimates [Stewart et al 2011].

Table 8. Health economic parameters, values and sources.

Parameter	Value	Source
Cost of bed day	For ICU: £1,353<br><br>For general medicine: £306<br><br>Average bed: £333	National Health Service (NHS) reference costs (median of ICU1 bed day cost distribution) [DH 2008]<br><br>Inpatient General Medicine services provided by NHS Trusts and PCTs the average cost per bed day was £306 in 2009-10. This includes Excess bed days and costs. UZ01Z (errors) and WD HRGs (mental health) are excluded.<br><br>From analysis of individual level hospital data, out of all patient days 2.6% are spent in ICU. Therefore average cost of a bed day = 298+35=£333
Swabbing costs	£3.20	Pathfinder [Health Protection Scotland 2011b] (for a large general hospital)
Screening costs (chromogenic agar – laboratory costs):<br>Positive result<br>Negative result	£7.24<br>£4.24	Pathfinder [Health Protection Scotland 2011b]
Incremental daily cost of isolating patient in a separate room	£88.43	Pathfinder [Health Protection Scotland 2011b] (estimate for large general hospital)
Contact precaution costs	£19.53	Pathfinder [Health Protection Scotland 2011b] (estimate for large general hospital)
Decolonisation costs	£54.01	Pathfinder [Health Protection Scotland 2011b]
Treatment costs	£530	MECAMIP [Robotham et al 2011]
Quality adjusted life expectancy	10 years<br><br>7 years for 2.4% of those discharged with an infection	MECAMIP [Robotham et al 2011] (general medical ward estimate)<br><br>2.4% of those patients with infections have long-term health loss. Estimate for health loss is taken from Scottish Pathfinder study at 30% reduction in QALE [Health Protection Scotland 2011b].

Appendix 4a. Parameters derived from the NOW audit.

Acute trusts

Table.1. Parameters derived from NOW audit by admission method and specialty: High Risk [HR]/Low Risk [LR] (Acute trusts)

		A	B	C	D	E	F	G	H	I	J
Elect	HR	4501.4	29.5	4471.9	12.8	16.7	4.4	8.4	16.7	1891.6	2580.3
	LR	26758.6	175.5	26583.1	76.2	99.3	27.9	48.3	99.3	11643.4	14939.7
Emerg	HR	2561.9	58.4	2503.5	26.6	31.9	19.8	6.9	31.9	1554.7	948.8
	LR	33521.1	764.6	32756.5	347.5	417.1	238.4	109.2	417.1	18245.4	14511.9

A= Total number of MRSA screens

B= Total no of Screens MRSA+ve

C= Total no of Screens MRSA-ve

D= Total of MRSA +ves newly positive

E= Total of MRSA+ves previously positive

F= Total of new MRSA+ves checklist positive

G= Total of new MRSA +ves checklist negative

H= Total of previous MRSA+ves checklist positive
I= Total of MRSA -ves checklist positive
J= Total of MRSA -ves checklist negative

Table 2. Proportions of patients checklist positive to checklist activated screening by admission type and specialty. High Risk [HR]/Low Risk [LR] (Acute trusts)

		1	2	3	4	5	6	7	8	9	10
Elect	HR	1912.7	2588.7	21.1	0.0110	0.4249	1.10	8.4	0.00324	0.32	3.40
	LR	11770.6	11671.3	147.6	0.0125	0.4399	1.25	27.9	0.00239	0.24	5.25
	All	13683.3	14260	168.7	0.0123	0.4377	1.23	36.3	0.00255	0.25	13683.3
Emerg	HR	1606.4	955.7	51.7	0.0322	0.6270	3.22	6.9	0.00722	0.72	4.46
	LR	18900.9	14621.1	655.5	0.0347	0.5639	3.47	109.2	0.00747	0.75	4.64
	ALL	18900.9	14621.1	655.5	0.0347	0.5639	3.47	109.2	0.00747	0.75	4.64

1=Total no checklist positive
2=Total checklist negative
3= Total MRSA+ves checklist positive
4=Proportion of checklist positives that are MRSA+ve
5=Proportion of admissions checklist positive
6=% of checklist +ves that are MRSA+ve
7=Total MRSA+ves that are checklist +ve
8=Proportion checklist –ves that are MRSA+ve
9=% checklist –ves that are MRSA+ve
10= How much more likely are checklist +ve patients to be MRSA+ve

Table 3. Proportions of screens MRSA+ve by admission type and admission specialty High Risk [HR]/Low Risk [LR] (Acute trusts)

		Total screens	Proportion screens (elective/emergency)	Proportion (all screens)	No screens +ve	Proportion screens +ve
Elect	HR	4501.4	0.144		29.5	0.007
	LR	26758.6	0.856		175.5	0.007
	ALL	31260	1.000	0.464	205	0.007
Emerg	HR	2561.9	0.071		58.4	0.023
	LR	33521.1	0.929		764.6	0.023
	ALL	36083	1.000	0.536	823	0.023

Table 4. Proportions of MRSA+ves previously known to be MRSA+ve by admission type and admission specialty High Risk [HR]/Low Risk [LR] (Acute Trusts)

		Total MRSA+ve	Number previously known MRSA+ve	Proportion previously known MRSA+ve
Elect	HR	29.5	16.700	**0.566101695**
	LR	29.5	16.700	**0.566101695**
	ALL	205	116.000	0.566
Emerg	HR	58.4	31.900	0.546232877
	LR	764.6	417.100	0.545513994
	ALL	823	449.000	0.546

Teaching trusts

Table5. Parameters derived from NOW audit by admission method and specialty High Risk [HR]/Low Risk [LR] (teaching trusts)

		A	B	C	D	E	F	G	H	I	J
Elect	HR	3739.3	34.1	3705.3	17.8	16.3	6.1	11.7	16.3	1567.3	3739.3
	LR	11399.7	103.9	11295.8	54.2	49.7	19.8	34.4	49.7	4947.6	11399.7
Emerg	HR	1428.5	23.9	1404.6	12.4	11.5	9.3	3.2	11.5	872.3	1428.5
	LR	12307.5	206.1	12101.4	106.6	99.5	73.1	33.5	99.5	6740.5	12307.5

A= Total number of MRSA screens

B= Total no of Screens MRSA+ve

C= Total no of Screens MRSA-ve

D= Total of MRSA +ves newly positive

E= Total of MRSA+ves previously positive

F= Total of new MRSA+ves checklist positive

G= Total of new MRSA +ves checklist negative

H= Total of previous MRSA+ves checklist positive
I= Total of MRSA -ves checklist positive
J= Total of MRSA -ves checklist negative

Table 6. Proportions of patients checklist positive to checklist activated screening by admission type and specialty High Risk [HR]/Low Risk [LR] (teaching trusts)

		1	2	3	4	5	6	7	8	9	10
Elect	HR	1589.7	2149.7	22.4	0.0141	0.4251	1.41	11.7	0.00544	0.54	2.59
	LR	5017.1	6382.6	69.5	0.0139	0.4401	1.39	34.4	0.00539	0.54	2.57
	All	6606.8	8532.3	91.9	0.0139	0.4364	1.39	46.1	0.00540	0.54	2.57
Emerg	HR	893.1	526.5	20.8	0.0233	0.6252	2.33	3.2	0.00608	0.61	3.83
	LR	6913.1	5394.4	172.6	0.0250	0.5617	2.50	33.5	0.00621	0.62	4.02
	ALL	7806.2	5920.9	193.4	0.0248	0.5683	2.48	36.7	0.00620	0.62	4.00

1=Total no checklist positive
2=Total checklist negative
3= Total MRSA+ves checklist positive
4=Proportion of checklist positives that are MRSA+ve
5=Proportion of admissions checklist positive
6=% of checklist +ves that are MRSA+ve
7=Total MRSA+ves that are checklist +ve
8=Proportion checklist –ves that are MRSA+ve
9=% checklist –ves that are MRSA+ve
10= How much more likely are checklist +ve patients to be MRSA+ve

Table 7. Proportions of screens MRSA+ve by admission type and admission specialty High Risk [HR]/Low Risk [LR] (teaching trusts)

		Total screens	Proportion screens (elective/emergency)	Proportion (all screens)	No screens +ve	Proportion screens +ve
Elect	HR	3739.3	0.247		34.1	0.009
	LR	11399.7	0.753		103.9	0.009
	ALL	15139	1.000	0.524	138	0.009
Emerg	HR	1428.5	0.104		23.9	0.017
	LR	12307.5	0.896		206.1	0.017
	ALL	13736	1.000	0.476	230	0.017

Table 8. Proportions of MRSA+ves previously known to be MRSA+ve by admission type and admission specialty High Risk [HR]/Low Risk [LR] (teaching Trusts)

		Total MRSA+ve	Number previously known MRSA+ve	Proportion previously known MRSA+ve
Elect	HR	34.1	16.3	**0.478**
	LR	103.9	49.7	**0.478**
	ALL	138	66	0.478
Emerg	HR	23.9	11.5	0.481
	LR	206.1	99.5	0.483
	ALL	230	111	0.483

Specialist trusts

Table9. Parameters derived from NOW audit by admission method and specialty High Risk [HR]/Low Risk [LR] (Specialist trusts)

		A	B	C	D	E	F	G	H	I	J
Elect	HR	682.8	7.5	675.3	3	4.5	1	2	4.5	285.7	682.8
	LR	2325.2	25.5	2299.7	10.1	15.5	3.7	6.4	15.5	1007.3	2325.2
Emerg	HR	215.2	1.7	213.5	1.3	0.3	1	0.3	0.3	132.6	215.2
	LR	436.8	3.4	433.5	2.7	0.7	1.9	0.9	0.7	241.6	436.8

A= Total number of MRSA screens

B= Total no of Screens MRSA+ve

C= Total no of Screens MRSA-ve

D= Total of MRSA +ves newly positive

E= Total of MRSA+ves previously positive

F= Total of new MRSA+ves checklist positive

G= Total of new MRSA +ves checklist negative

H= Total of previous MRSA+ves checklist positive
I= Total of MRSA -ves checklist positive
J= Total of MRSA -ves checklist negative

Table 10. Proportions of patients checklist positive to checklist activated screening by admission type and specialty. High Risk [HR]/Low Risk [LR] (specialist trusts)

		1	2	3	4	5	6	7	8	9	10
Elect	HR	291.2	391.7	5.5	0.0189	0.4265	1.89	2	0.00511	0.51	3.70
	LR	1026.5	1298.8	19.2	0.0187	0.4415	1.87	6.4	0.00493	0.49	3.80
	All	1317.7	1690.5	24.7	0.0187	0.4381	1.87	8.4	0.00497	0.50	3.77
Emerg	HR	133.9	81.2	1.3	0.0097	0.6222	0.97	0.3	0.00369	0.37	2.63
	LR	244.2	192.9	2.6	0.0106	0.5591	1.06	0.9	0.00467	0.47	2.28
	ALL	378.1	274.1	3.9	0.0103	0.5799	1.03	1.2	0.00438	0.44	2.36

1=Total no checklist positive
2=Total checklist negative
3= Total MRSA+ves checklist positive
4=Proportion of checklist positives that are MRSA+ve
5=Proportion of admissions checklist positive
6=% of checklist +ves that are MRSA+ve
7=Total MRSA+ves that are checklist +ve
8=Proportion checklist –ves that are MRSA+ve
9=% checklist –ves that are MRSA+ve
10= How much more likely are checklist +ve patients to be MRSA+ve

Table 11. Proportions of screens MRSA+ve by admission type and admission specialty High Risk [HR]/Low Risk [LR] (specialist trusts)

		Total screens	Proportion screens (elective/emergency)	Proportion (all screens)	No screens +ve	Proportion screens +ve
Elect	HR	682.8	0.227		7.5	0.011
	LR	2325.2	0.773		25.5	0.011
	ALL	3008	1.000	0.822	33	0.011
Emerg	HR	215.2	0.330		1.7	0.008
	LR	436.8	0.670		3.4	0.008
	ALL	652	1.000	0.178	5.1	0.008

Table 12. Proportions of MRSA+ves previously known to be MRSA+ve by admission type and admission specialty High Risk [HR]/Low Risk [LR] (specialist Trusts)

		Total MRSA+ve	Number previously known MRSA+ve	Proportion previously known MRSA+ve
Elect	HR	7.5	4.500	0.6
	LR	25.5	15.500	0.607843137
	ALL	33	20.000	0.606
Emerg	HR	1.7	0.300	0.176470588
	LR	3.4	0.700	0.205882353
	ALL	5.1	1.000	0.196

Appendix 5a. Sentinel Questionnaire

Section A. Patient readmissions - elective and emergency.

All patients (For completion by informatics)

Please record the number of all patients discharged between 1st April 2010 and 31st March 2011 and readmitted to the same or another consultant specialty within 30 days.

Total number of discharges from "high risk"* specialties.	
Number of those discharges readmitted to ***any*** specialty within 30 days.	
Number of those discharges readmitted to the ***same*** specialty within 30 days.	

* = Haematology/oncology, neurosurgery, nephrology, trauma/orthopaedics, cardiothoracic surgery, vascular surgery.

Total number of discharges from "low-risk"** specialties.	
Number of those discharges readmitted to ***any*** specialty within 30 days.	
Number of those discharges readmitted to the ***same*** specialty within 30 days.	

**= All other specialties not mentioned above

Instructions:

- Include emergency and elective admissions/discharges
- Include only those discharge episodes that occur between 1st April 2010 and 31st March 2011.
- Readmission episodes between the 1st and 30th of April that are **also** connected to a discharge occurring in March 2011 should be included in the dataset.

Section A contd. Patient readmissions - elective and emergency (contd).

II. MRSA positive patients (For completion by informatics and infection control team)

Please record the number of all MRSA +ve patients discharged between 1st April 2010 and 31st March 2011 and readmitted to the same or another consultant specialty within 30 days.

Total number of discharges from "high risk"* specialties.	
Number of those discharges readmitted to ***any*** specialty within 30 days.	
Number of those discharges readmitted to the ***same*** specialty within 30 days.	

* = Haematology/oncology, neurosurgery, nephrology, trauma/orthopaedics, cardiothoracic surgery, vascular surgery.

Total number of discharges from "low-risk"** specialties.	
Number of those discharges readmitted to ***any*** specialty within 30 days.	
Number of those discharges readmitted to the ***same*** specialty within 30 days.	

**= All other specialties not mentioned above

Instructions:

- The infection control team will need to identify all patients flagged as MRSA +ve previous to 1st April 2010. Include those patients for whom 3 negative screens had not been obtained. This list should then be supplied to the informatics team to run the readmission query.
- Include emergency and elective admissions/discharges
- Include only those discharge episodes that occur between 1st April 2010 and 31st March 2011**.**
- Readmission episodes between the 1st and 30th of April that are **also** connected to a discharge occurring in March 2011 should be included in the dataset.

Section B. Number of ward stays per patient admission - elective and emergency (For completion by informatics with information provided by Infection prevention and control teams)

For patients discharged between 1st April 2010 and 31st March 2011, please record the number of wards on which patients were nursed during each admission episode.

Specialty on discharge	Mean LOS	No. of patients with **one** ward stay per admission	No. of patients with **two** ward stays per admission	no of patients with **three** ward stays per admission	no of patients with **four** ward stays per admission	no of patients with **five or more** ward stays per admission
"High risk"*						
"Low Risk"**						

* = Haematology/oncology, neurosurgery, nephrology, trauma/orthopaedics, cardiothoracic surgery, vascular surgery.
**= All other specialties

Include A&E wards, observation wards, admission wards etc.

Section C. ITU Discharge destinations (For completion by informatics dept)

Please record the destination of ITU patients discharged from an ITU in your trust between 1st April 2010 and 31st March 2011.

Total Number discharged.	
Number died.	
Number transferred to a "high risk"* specialty.	
Number transferred to a "low risk" **specialty.	
Number transferred to another trust.	
Number transferred to any other destination	

*= Haematology/oncology, neurosurgery, nephrology, trauma/orthopaedics, cardiothoracic surgery, vascular surgery.
**= All other specialties

Section D. Numbers and types of Intensive Care Units (ICU). (For completion by infection control team)

Please record the number of ICU/HDU wards/beds in your trust (complete a separate column for each ICU/HDU)

	**ICU 1**	**ICU 2**	**ICU n**
Type [1]			
No of Beds			
No of single bedded rooms			

[1]Specify type ie general medical/surgical, cardiothoracic, liver, neurological, paediatric medical/surgical, neonatal, spinal injury neurological etc.

Instructions

Include: Adult ICUs, Paediatric ICUs, Neonatal Intensive Care units, High dependency units, coronary care units.

Section E Using PCR for MRSA admission screening (For completion by microbiology lab manager)

1. Could you introduce MRSA admission screening using PCR as your **main** laboratory method with your existing staff?

Yes ☐ go to q3 No ☐ go to q2

2. If no, how many additional staff would you need and at what grade?

Agenda for change grade	1	2	3	4	5	6	7	8	9	
Number required										

3. Could you introduce MRSA admission screening using PCR as your **main** laboratory method with your existing capital?

Yes ☐ No ☐ go to q4

4. If no, how much further capital investment would you need?

£..............................

Section F. Antibiotic Costs (For completion by pharmacy manager)

Please record the total quantity and total cost for the following antibiotics used in the trust between 1st April 2010 and 31st March 2011.

Grade	Vanc IV	Doxy IV	Doxy po	Daptomycin IV	Synercid IV	Linezolid IV	Linezolid po	Fusidic acid iv	Fusidic acid po
Total amount (kilos)									
Total cost (Pounds)									

Appendix 5b. Sentinel data. Methods and results

SECTION A

Proportions of readmissions were higher for patients originally admitted to HR specialties and for MRSA-ve patients. The lowest proportion of readmissions was for MRSA-ve patients admitted to low risk specialties (21%). The median proportion of readmissions per trust was highest for MRSA-ve patients admitted to HR specialties (49%: IQR 10-74%). This may be explained by the fact that one of the trusts was a tertiary referral centre and had a large number of patients with multiple admissions for renal dialysis.

Table 1. Readmissions within 30 days.

		**% readmitted**	**%readmitted to same specialty**	**Median (IQR) by trust**
MRSA+ve	HR (5 trusts)	468/1187 (39.4%)	387/468 (83%)	30% (11-46%)
	LR (6 trusts)	2474/9503 (26%)	1115/2474 (45%)	18% (14-25%)
MRSA-ve	HR (7 trusts)	108705/161601 (67%)	23561/108705 (22%)	49% (10%-74%)
	LR (7 trusts)	114964/572539 (21%)	48590/114964 (42%)	25% (9%-27%)

SECTION B

Data on the number of ward stays per admission were received from 7 trusts. This represents 73 028 admissions (162 302 to High risk specialties and 567 966 to Low risk specialties).

There was relatively little movement between wards of patients admitted to high-risk specialties. 87% remained on one ward during the whole admission compared to 74% of low-risk patients.

Table 2. Percentage of patients with 1-5+ ward stays in a single admission

	No of ward stays per admission				
	1	2	3	4	5+
All patients	77%	16%%	5%	2%	1%
HR specialties	87%	9%	3%	1%	1%
LR specialties	74 %	18%	5%	2%	1%

SECTION C

Data were received from 7 trusts regarding the destinations of ITU patients discharged/transferred in the financial year 2010-2011. Data were available for 7890 patients of whom the majority (53%) were transferred to a low-risk specialty within the trust

Table 3. ITU Discharge destination

Number discharged	Died	Transferred to HR specialty	Transferred to LR specialty	Other
7890	1039(13%)	1119 (14%)	4168(53%)	1564 (20%)

SECTION D

Eight Trusts reported a total of 34 ITU wards (258 beds).

The median number of ITU/HDU wards per trust was4.5 (IQR 3-6) and the median number of beds per ward was 8 (IQR 6-12).

The total proportion of side-rooms was 34/258 (16%) with a median proportion per ward of 29% (IQR 19-34%)

SECTION E

In response to the question about how many extra staff would be needed if PCR were used as the as main laboratory method for admission screening, 6/7 respondents answered that they would require a median of 2.5 extra staff ranging from 1.5 to 3.5.

In terms of capital investment required to introduce routine PCR screening , 6 trusts returned estimates ranging from £30 000 to £290 000 (median £80 000)

SECTION E.

Data regarding annual spend on a range of antibiotics was returned by 7 trusts. This represented a total spend of £804 512 with a median overall spend per trust of £117 371 (IQR £36 452- £144 519).

IV Vancomycin represented the largest proportion of this spend (36.7% of the total) closely followed by oral Linezolid.

Table 4 . Annual spend by 7 trusts on individual antibiotics and the relative proportion of the total spend.

Vanc IV	Doxy IV	Doxy po	Daptomycin IV	Synercid IV	Linezolid IV	Linezolid po	Fusidic acid IV	Fusidic acid po
£295904 (36.7%)	£0 (0%)	£16117.74 (2%)	£29493.46 (3.7%)	£26109.05 (3.3%)	£142449.60 (17.7%)	£238979.68 (29.7%)	£20351.23 (2.5%)	£35107.63 (4.3%)

Appendix 6. Expected number of screens per week

Table 1: Total numbers of *high-risk* specialty patients by screening category and mean numbers per trust per week (All responding trusts). Using five-item checklist

	Elective patients (total number + (mean per trust) n=127 trusts	Emergency patients total number + (mean per trust) n=127 trusts
A. Total number of MRSA screens	7315.4 (57.6)	3703.9 (29.16)
B. Total no of screens MRSA +ve	55.6 (0.44)	77.5 (0.61)
C. Total no of screens MRSA negative	7259.8 (57.16)	3626.4 (28.55)
D. Total of MRSA +ves newly positive	29.8 (0.23)	36.4 (0.29)
E. Total of MRSA +ves previously positive	25.8 (0.20)	41.8 (0.32)
F. Total no of new MRSA +ves, checklist positive	10.2 (0.08)	27.2 (0.21)
G. Total no of new MRSA +ves checklist negative	19.6 (0.15)	9.2 (0.07)
H. Total of previous MRSA +ves checklist positive	25.8 (0.20)	41.8 (0.32)
I. Total no of MRSA negatives checklist positive	3070.9 (24.18)	2252 (18.16)
J. Total no of MRSA negatives checklist negative	4188.9 (32.98)	1374.4 (10.82)

Table 2: Total numbers of *low-risk* specialty patients by screening category an mean numbers per trust per week (All responding trusts) using five-item checklist

	Elective patients total number + (mean per trust) n=127 trusts	Emergency patients total number + (mean per trust) n=127 trusts
A. Total number of MRSA screens	42280 (332.92)	47035 (370.35)
B. Total no of screens MRSA +ve	321.4 (2.53)	984.5 (7.75)
C. Total no of screens MRSA negative	41959 (330.39)	46050. (362.6)
D. Total of MRSA +ves newly positive	172.2 (1.36)	461.7 (3.64)
E. Total of MRSA +ves previously positive	149.2 (1.17)	522.8 (4.12)
F. Total no of new MRSA +ves, checklist positive	63.0 (0.50)	316.7 (2.50)
G. Total no of new MRSA +ves checklist negative	109.2 (0.86)	145 (1.14)
H. Total of previous MRSA +ves checklist positive	149.2 (1.17)	522.8 (4.12)
I. Total no of MRSA negatives checklist positive	18378 (144.71)	25650 (207.97)
J. Total no of MRSA negatives checklist negative	23581 (185.68)	20400 (160.63)

Table 3: Total numbers of *high-risk* specialty patients by screening category and mean numbers per trust per week (All trusts) using Pathfinder 3-item checklist

	**Elective patients (total number + (mean per trust) n=127 trusts**	**Emergency patients total number + (mean per trust) n=127 trusts**
A. Total number of MRSA screens	7315.3 (57.6)	3703.9 (29.16)
B. Total no of screens MRSA +ve	55.6 (0.44)	77.5 (0.61)
C. Total no of screens MRSA negative	7259.8 (57.16)	3626.4 (28.55)
D. Total of MRSA +ves newly positive	29.8 (0.23)	36.4 (0.29)
E. Total of MRSA +ves previously positive	25.8 (0.20)	41.2 (0.32)
F. Total no of new MRSA +ves, checklist positive	6.7 (0.05)	20.1 (0.16)
G. Total no of new MRSA +ves checklist negative	23.1 (0.18)	16.3 (0.13)
H. Total of previous MRSA +ves checklist positive	25.8 (0.20)	41.8 (0.32)
I. Total no of MRSA negatives checklist positive	776.8 (6.12)	1207.6 (9.51)
J. Total no of MRSA negatives checklist negative	6483.0 (51.05)	2415.2 (19.01)

Table 4: Total numbers of *low-risk* specialty patients by screening category and mean numbers per trust per week (All trusts) using Pathfinder 3-item checklist

	**Elective patients total number + (mean per trust) n=127 trusts**	**Emergency patients total number + (mean per trust) n=127 trusts**
A. Total number of MRSA screens	42279.7 (332.92)	47035 (370.35)
B. Total no of screens MRSA +ve	321.4 (2.53)	984.5 (7.75)
C. Total no of screens MRSA negative	41958.3 (330.39)	46050.6 (362.6)
D. Total of MRSA +ves newly positive	172.2 (1.36)	461.7 (3.64)
E. Total of MRSA +ves previously positive	149.2 (1.17)	522.8 (4.12)
F. Total no of new MRSA +ves, checklist positive	23.1 (0.18)	191.6 (1.51)
G. Total no of new MRSA +ves checklist negative	149.1(1.17)	270.1 (2.13)
H. Total of previous MRSA +ves checklist positive	149.2 (1.17)	522.8 (4.12)
I. Total no of MRSA negatives checklist positive	4237.9 (33.37)	10453.4 (82.31)
J. Total no of MRSA negatives checklist negative	37721.1 (297.02)	35596.6 (280.29)

Appendix 7: Results of cost effectiveness evaluation

This section presents results of the cost-effectiveness analysis of each of the alternative screening and control strategies.

The screening strategies evaluated using each model were:

1. no screening,
2. routine screening all Elective and Emergency admissions,
3. screening only admissions to “high-risk” specialties,
4. patient level risk-based screening of all admissions (checklist activated screening,
5. screening admissions to “high-risk” specialties plus checklist activated screening of admissions to “low-risk” specialties,
6. screening of all admissions with pre-emptive isolation of those known to be previously MRSA positive.

Results are split by Trust type. For each Trust type we first present results in terms of the *effectiveness* of each strategy, followed by the *costs* of each strategy (split into cost components), then we combine both costs and effects by presenting results cost-effectiveness planes and as mean incremental cost-effectiveness ratios (ICERs). In order to include the impact of uncertainty (namely the effectiveness of interventions, which has previously been shown to be the dominating parameter [Robotham et al 2011] we present the results of probabilistic sensitivity analyses. Here we plot results of multiple model runs under multiple parameter values and show the full extent of uncertainty in the model outputs (due to uncertainty in model inputs). This uncertainty is considered in the comparison of policies in the cost-effectiveness acceptability curves (CEACs). CEACs show the *proportion of simulations* in which each strategy is cost-effective. While CEACs are valuable for representing the degree of uncertainty in the optimal strategy, and thus showing us how likely each strategy is to be suboptimal for each willingness to pay, they do not in themselves contain sufficient information to enable us to rationally choose between the strategies. This is because they take no account of the absolute magnitude of the net monetary benefit (NMB) associated with each strategy at each

set of parameter values, but consider only the relative magnitude (which strategy has the highest NMB). A more rational approach would be to choose the strategy with the highest *expected* NMB over the full range of parameter uncertainty. This is shown on the cost-effectiveness acceptability frontiers (CEAFs) which show only the probability that the strategy with the highest NMB is cost-effective for a given willingness to pay.

We also present results of numerous scenario analyses:

- For each Trust type we compare strategies under baseline prevalence, as well as a prevalence of twice and half this value.
- For Acute Trusts only (and under baseline prevalence values) we evaluate strategies where:
    - Transmission parameters in high risk settings are reduced to the midpoint between ICU and general medical ward estimates.
    - Daily death probabilities in high risk specialties are reduced to be equivalent to those in low risk specialties.

Lastly, and again for Acute Trusts only under baseline prevalence, we present an evaluation of strategies where the definition of ‘checklist positive’, determining who is screened under a checklist activated screening strategy, conforms to the Scottish pathfinder definition of ‘checklist positive’ [Stewart et al 2011].

8 A Acute Trusts

1. Baseline analyses

Effectiveness

Figure A1 shows the degree to which each of the strategies applied the primary intervention (i.e. use of side rooms accompanied by patients decolonisation) appropriately - where MRSA positive patients were isolated, inappropriately - where MRSA negative patients were isolated, as well as the number of unisolated days – MRSA positive days spent out of side room isolation.

As may be expected, strategies 2 and 6 (screening all admissions, and screening all admissions with pre-emptive isolation of those known to be previously MRSA positive) do particularly well in terms of appropriate isolation use, but also give most inappropriate isolation days (simply due to a greater number of patients being isolated). Strategy 6, which includes pre-emptive isolation of patients known to be previously MRSA positive, reduces unisolated MRSA positive bed days only slightly compared to simply screening all patients. This is because those who are infected typically have long stays, therefore catching a small proportion of the MRSA positive population 'early' through pre-emptive isolation makes little overall difference to unisolated days. This finding would be missed if infection status dependent lengths of stay were not included within the model.

Strategy 3 (screening admissions to high risk specialties) appropriately (and inappropriately) isolates very few patients, with the majority remaining unisolated. This is due to the relatively small proportion of admissions going to high risk specialties (16% of beds in Acute Trusts belong to high risk specialties. Strategies 4 and 5 (checklist activated screening (CAS), and screening all high risk specialty patients plus 'CAS of patients in low risk specialties, respectively) perform between the two extremes described above. However, it is worth noting that strategy 4, screening of only 'checklist positive' patients (and isolating those identified as positive), while reducing the amount of appropriate isolation (compared to

screening all patients) by ~30%, reduces inappropriate isolation usage by over 50%. This is because the prevalence in the checklist positive group is approximately 2.6% compared to 1.4% of the overall Acute admission population. Therefore, if isolation capacity is a limiting factor, checklist activated screening may be an option to ‘free up’ 50% of the isolation capacity.

Figure A1. Primary isolation usage under each screening strategy, showing appropriate isolation (isolation of patients who are MRSA positive), inappropriate isolation (isolation of MRSA negative patients) and unisolated bed days of MRSA positive patients.

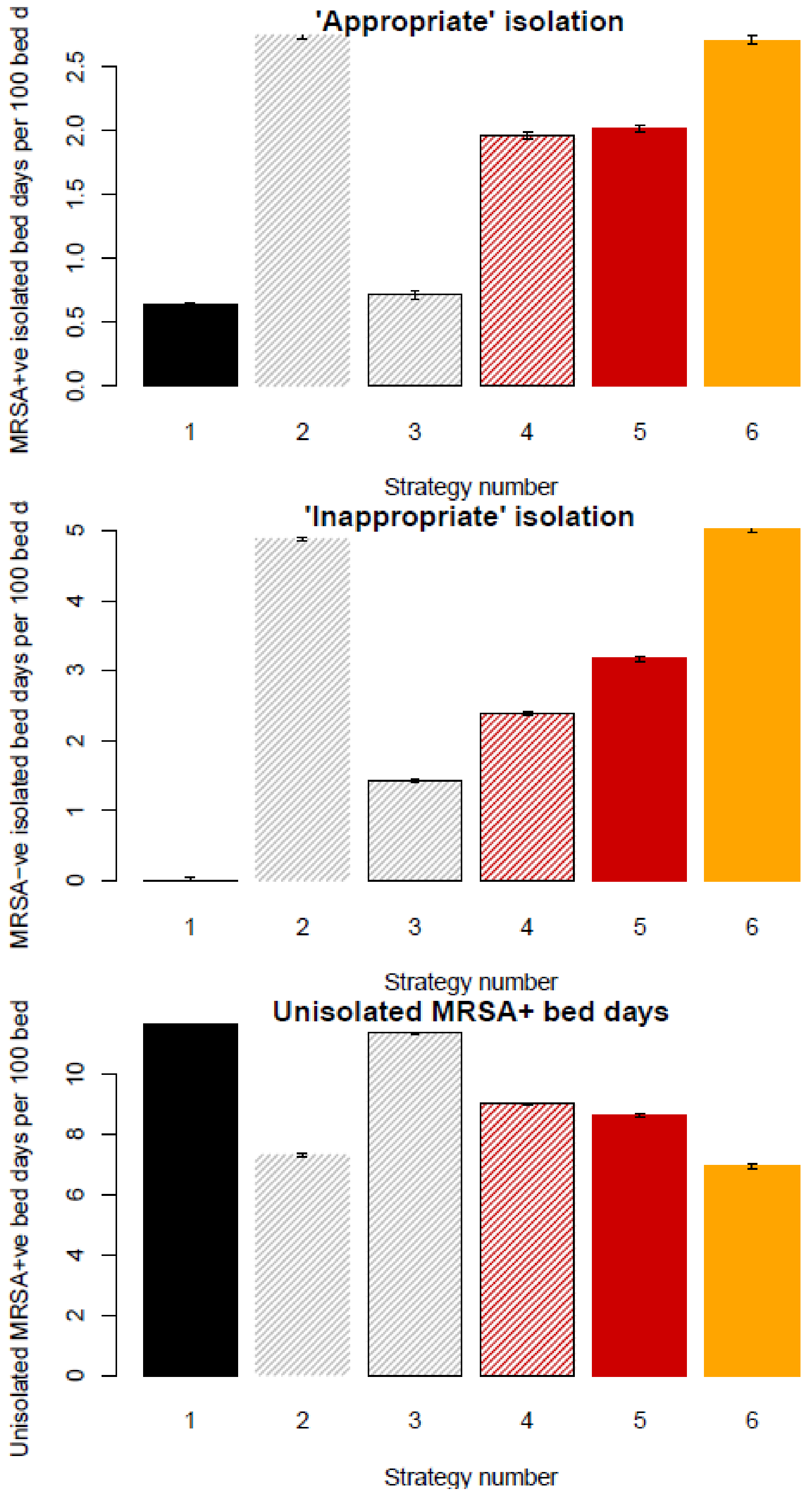

Figure A2 shows the extent to which the ability of each strategy to identify patients for control translates into reduction in transmission, infections and deaths. As may be expected, those strategies which appropriately isolate the greatest number of patients reduce transmission to the greatest extent, with strategies 2 and 6 (both involving screening of all patients) performing the best. However, overall reduction in transmission does not translate directly into reduction in MRSA infections; this is because some strategies concentrate control to high risk specialty patients. In the model the probability of acquiring infection is greater in high risk specialties compared to low risk specialties. This means that only small reductions in transmission in high risk settings can more greatly impact the subsequent number of infections and, as the probability of death is directly related to infection status, the number of deaths. Therefore strategies which have a greater impact on high risk specialties will have a greater impact on the number of infections and deaths. For example, while strategy 3 (screening high risk specialty patients only) only marginally reduces transmission, because this reduction in transmission occurs in high risk specialties it has a greater impact on reducing infection and deaths than other strategies which may reduce overall hospital transmission to a greater extent (e.g. strategy 4). The strategy reducing infections and deaths to the greatest degree is screening all patients plus pre-emptive isolation of those previously identified as MRSA positive (strategy 6).

Costs

Figure A3 shows total costs of each of the strategies, as well as costs broken down into their component parts. Aside from strategy 1, no screening (clinical cultures of infected patients only) strategy 3 is the least costly, due to the fact it screens and therefore isolates the fewest number of patients. The total costs per admission for each strategy can be seen to be dominated by bed day costs.

Figure A2. Patient outcomes under each screening strategy, showing new acquisitions of MRSA by hospital patients, total MRSA infections in the hospital, and total deaths (all per 100 admissions).

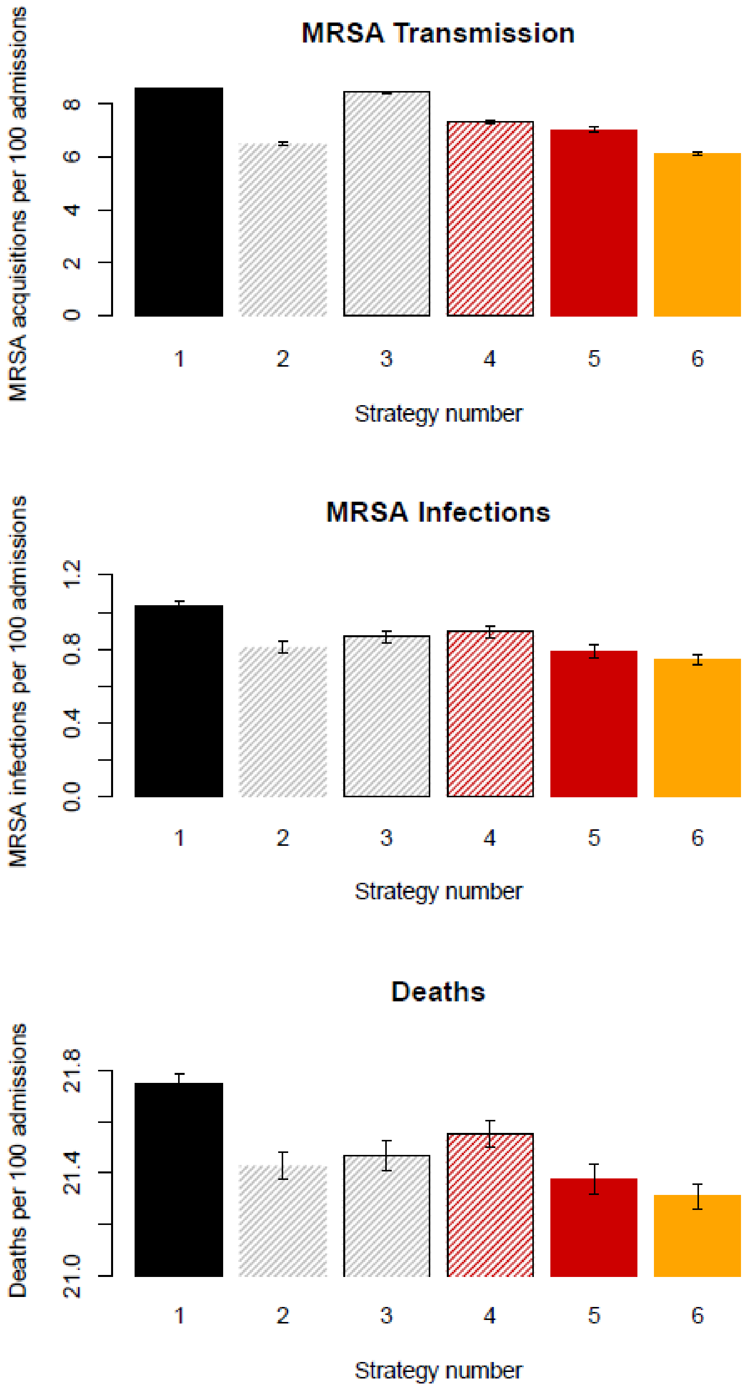

Figure A3. Cost per admission for each strategy, presented as total costs and broken down into component parts (note different scales of sub graphs).

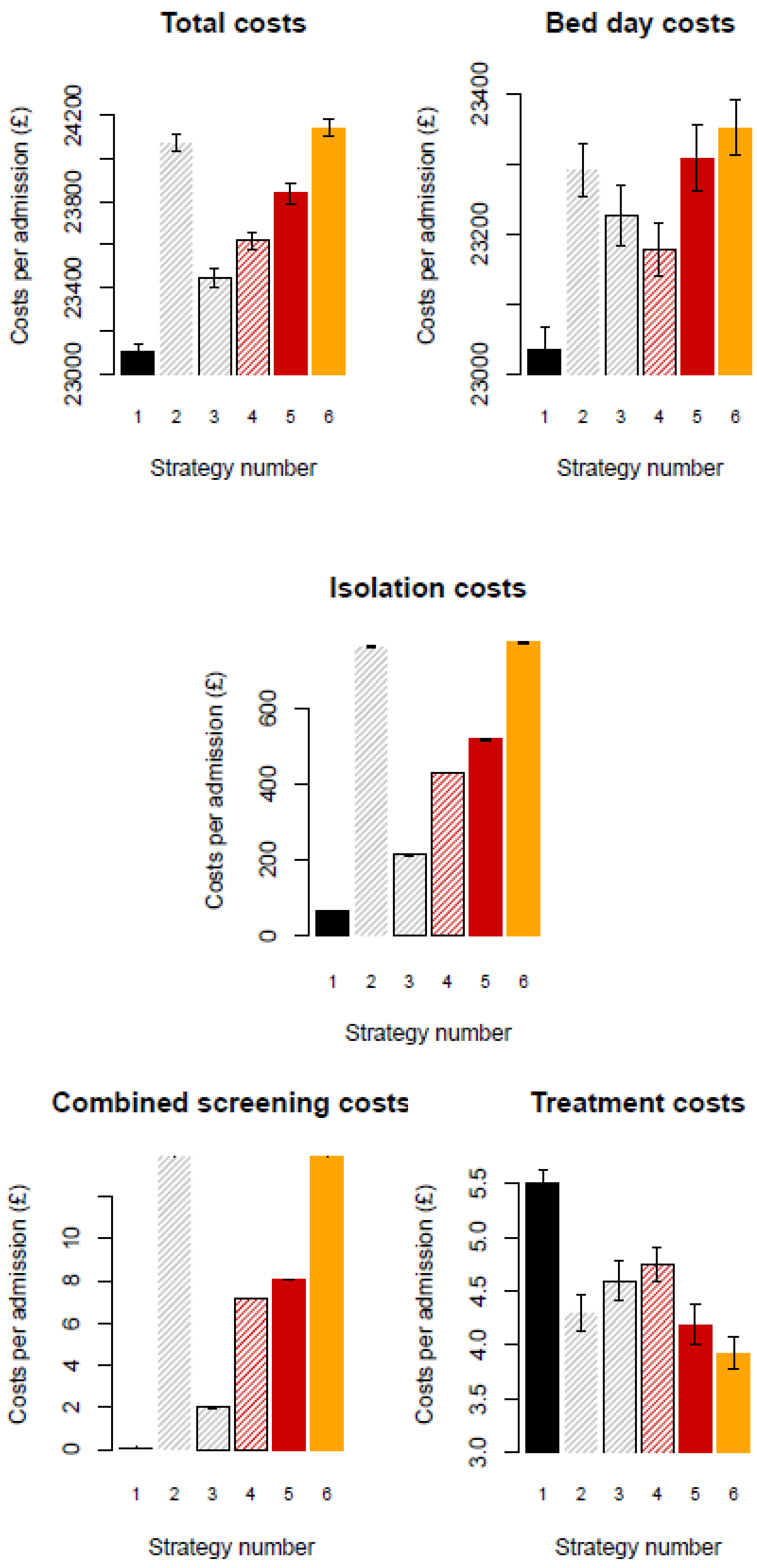

Cost-effectiveness

To combine these costs and effects in strategy assessment, each strategy can be evaluated on a cost-effectiveness plane. Here, effectiveness of each strategy is measured in terms of health-benefits (measured in QALYs) per admission. A reduction in the number of infections has two effects: 1) it decreases length of stay which decreases cost per admission and 2) it reduces the number of deaths. Both effects will lead to improvements in cost per QALYs gained.

Model results (Figure A4) confirmed that any investment in screening, compared to no screening, was likely to lead to increases in health benefits. While strategy 6 (screening of all patients plus pre-emptive isolation of those known to be previously MRSA positive) gave the highest health benefits, it was also associated with the greatest costs.

Figure A4. Incremental cost-effectiveness plot comparing each of the screening strategies. Numbers indicate strategy numbers as outlined above. Error bars represent random error brought about by stochasticity in the model and parameter uncertainty, and correspond to plus or minus one standard error.

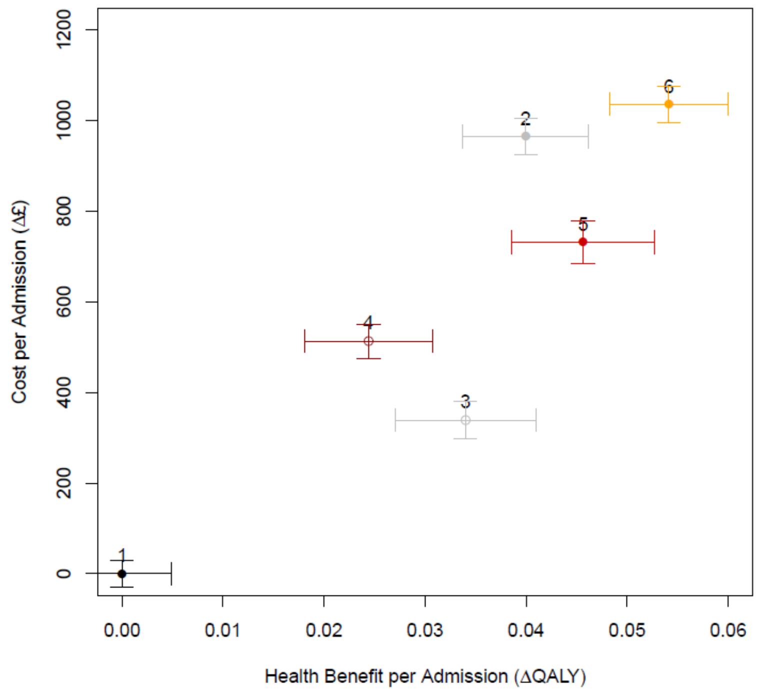

Table A1 describes all strategies in terms of mean change in costs and mean change in health benefits compared to the baseline (strategy 1), and then combines these in terms of a mean cost per QALY gained by changing strategy from the baseline ‘do nothing’ approach. Each strategy is also evaluated using the techniques of dominance and extended dominance, allowing some strategies to be eliminated from further evaluation. Dominated strategies are those that are both more costly and provide less benefit than at least one other strategy. An extendedly dominated strategy is one that is more costly and provides less benefit than a combination of another two strategies. Since it would never be cost-effective to pay more for less benefit, these strategies can be excluded from any further evaluation.

Table A1. Cost-effectiveness evaluation of screening strategies for an Acute Trust.

Screening strategy (ranked by cost)	Cost per admission	QALY per admission	Change in costs, ΔC (compared to baseline)	Change in effects, ΔE (compared to baseline)	Difference in Costs / Difference in Effects	Option evaluation
1	£23,106	8.05123	-	-	-	Remains
3	£23,445	8.08524	£338.86	0.03401	£9,964	Remains
4	£23,619	8.07565	£513.30	0.02441	£21,025	Dominated
5	£23,838	8.09687	£731.94	0.04564	£16,038	Remains
2	£24,071	8.09119	£965.06	0.03996	£24,149	Dominated
6	£24,142	8.10537	£1,036.14	0.05413	£19,140	Remains

Incremental cost-effectiveness evaluations are then applied to the remaining strategies, which form the ‘cost-effectiveness frontier’. We now evaluate each of the options along the cost-effectiveness frontier. We start at our existing strategy, initially our baseline strategy 1, and then ask whether it is cost-effective (in terms of some threshold of willingness to pay for health benefits) to move from the currently selected strategy to the next most costly strategy on the frontier. This process is iterated, calculating the change in costs and health benefits in moving to the next strategy on the frontier. We stop when no move from the current strategy to a new strategy would be cost-effective according to our threshold. This provides mean incremental cost-effectiveness ratios (ICERs).

Whether a move from one strategy to another is considered cost-effective is dependent on the decision-maker's maximum willingness to pay for a unit of health outcome, such as a QALY. NHS decision makers tend to use values between £20,000 and £30,000 per QALY for a willingness to pay threshold [Rawlins et al 2004.]. Here we use the upper threshold of £30,000 per QALY.

The evaluation of each move along the cost-effectiveness frontier is described in Table A2. The mean ICERs for strategies 1 and 3 fall beneath the cost-effectiveness threshold. The mean incremental cost per QALY for strategy 3 (screening all admissions to high risk specialties), at £9,964/QALY, was the final strategy to be considered cost-effective and would therefore be the optimal approach (under the specific model parameters and assumptions used). Strategy 5 was only marginally more costly than the £30,000 willingness to pay threshold at £33,806/QALY, and therefore whether to consider this option would be dependent on the decision-makers willingness to pay for health benefits.

Table A2 Evaluation of cost-effectiveness frontier of strategies for an Acute Trust.

Move between policies	Cost per admission	QALY per admission	Change in costs, ΔC (compared to previous policy)	Change in effects, ΔE (compared to previous policy)	Difference in Costs / Difference in Effects (ICER, cost/QALY)	Option evaluation
Stay at policy 1	£23,106	8.051232	-	-	-	-
Or move:						
to 3	£23,445	8.085241	£338.86	0.03401	£9,964	Cost effective
to 5	£23,838	8.096869	£393.07	0.011627	£33,806	Not cost effective
to 6	£24,142	8.105367	£304.20	0.008498	£35,796	Not cost effective

Consideration of uncertainty

When sources of parameter uncertainty (namely the effectiveness of the intervention) are accounted for, choosing between competing strategies becomes more complicated. On the cost-effectiveness plane (Figure A5) we can see that the costs and health benefits for each strategy are highly variable and show considerable overlap. This suggests that no single strategy is likely to be preferred over the entire range of plausible parameter values.

Figure A5. Simulation results comparing each strategy on a cost-effectiveness plane for an Acute Trust setting. Each dot represents the mean of 1000 simulation runs for each parameter set. The results of 50 parameter sets for each strategy are plotted, where each parameter set is obtained by taking the mean value for all parameters apart from the effectiveness of the intervention, which is sampled from its probability distribution (see appendix 4 table 7).

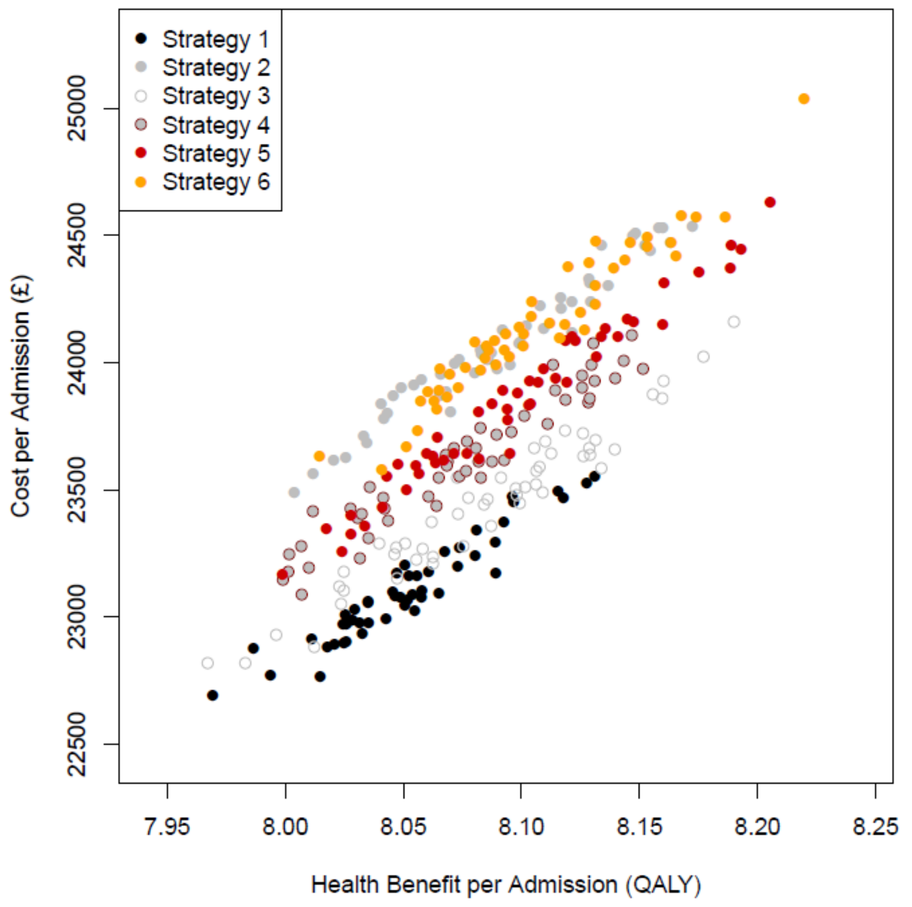

The importance of this uncertainty becomes clear when we consider the cost-effectiveness acceptability curves (CEACs), which show the proportion of simulations in which each strategy is cost-effective (Figure A6). CEACs therefore show the probability of each intervention being cost-effective *accounting for the variation in cost/QALY outcome for each model run* at each value of willingness to pay.

Figure A6 shows substantial uncertainty over which is the most cost-effective strategy as a result of the parameter uncertainty. Figure A6 shows that for willingness to pay values between £20,000 to £30,000 (the usual NHS threshold range) the probability that any one strategy is the most cost-effective option does not exceed 30%.

Figure A6. Cost-effectiveness acceptability curves. Each line represents the proportion of simulations, for a particular strategy, that are cost-effective, as a function of willingness to pay for health benefits.

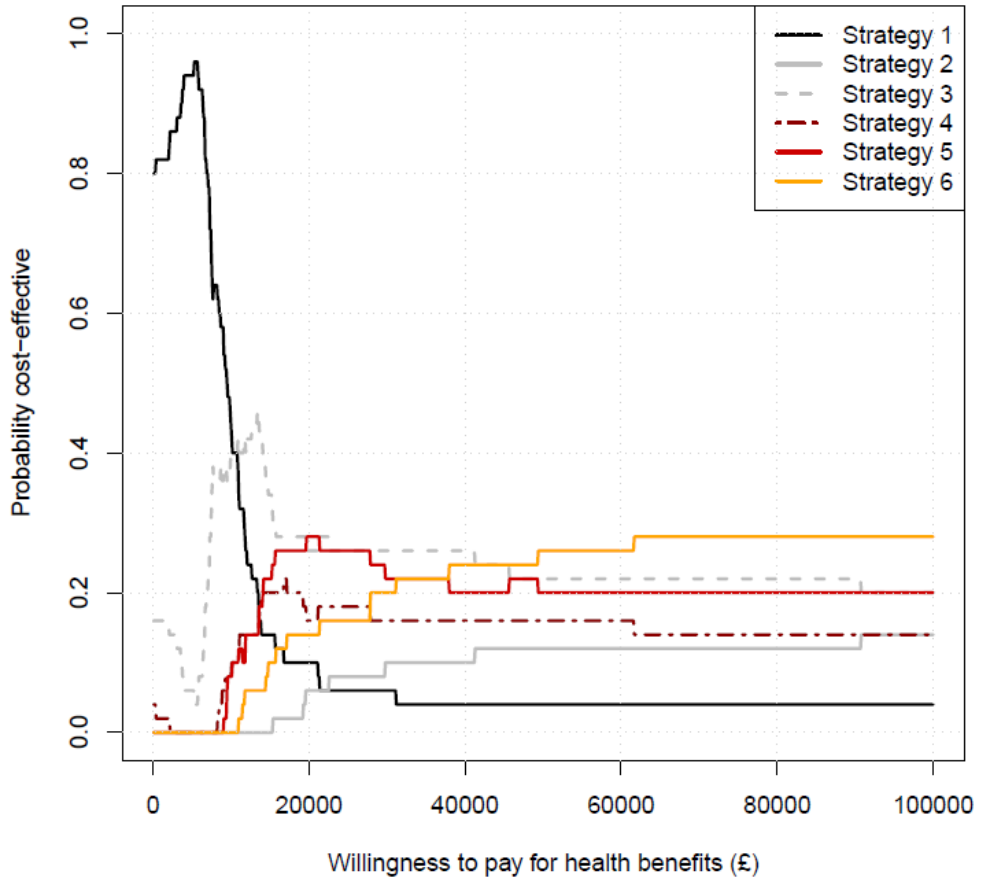

The decision is made slightly clearer by the CEAF (Figure A7), which shows only the strategy with the highest *expected* NMB over the full range of parameter uncertainty, and describes the probability that this strategy is cost-effective for a given willingness to pay. The CEAF therefore shows which strategy the model suggests we should choose for each willingness to pay. It can be seen that Figure A7 is split into 4 sections, meaning that the optimal strategy changes according to the willingness to pay for health benefits. At a willingness to pay of less than approximately £10,000/QALY, strategy 1 (the 'no screening' approach) is optimal. However above this, and up to the NHS willingness to pay threshold of £30,000/QALY, strategy 3 (screening of only high risk specialty patients) is the optimal option.

Figure A7. Cost-effectiveness acceptability frontier. Lines depict the strategies with the highest expected net monetary benefit, dependent on the willingness to pay for health benefits, while dotted vertical lines the willingness to pay values at which the decision changes.

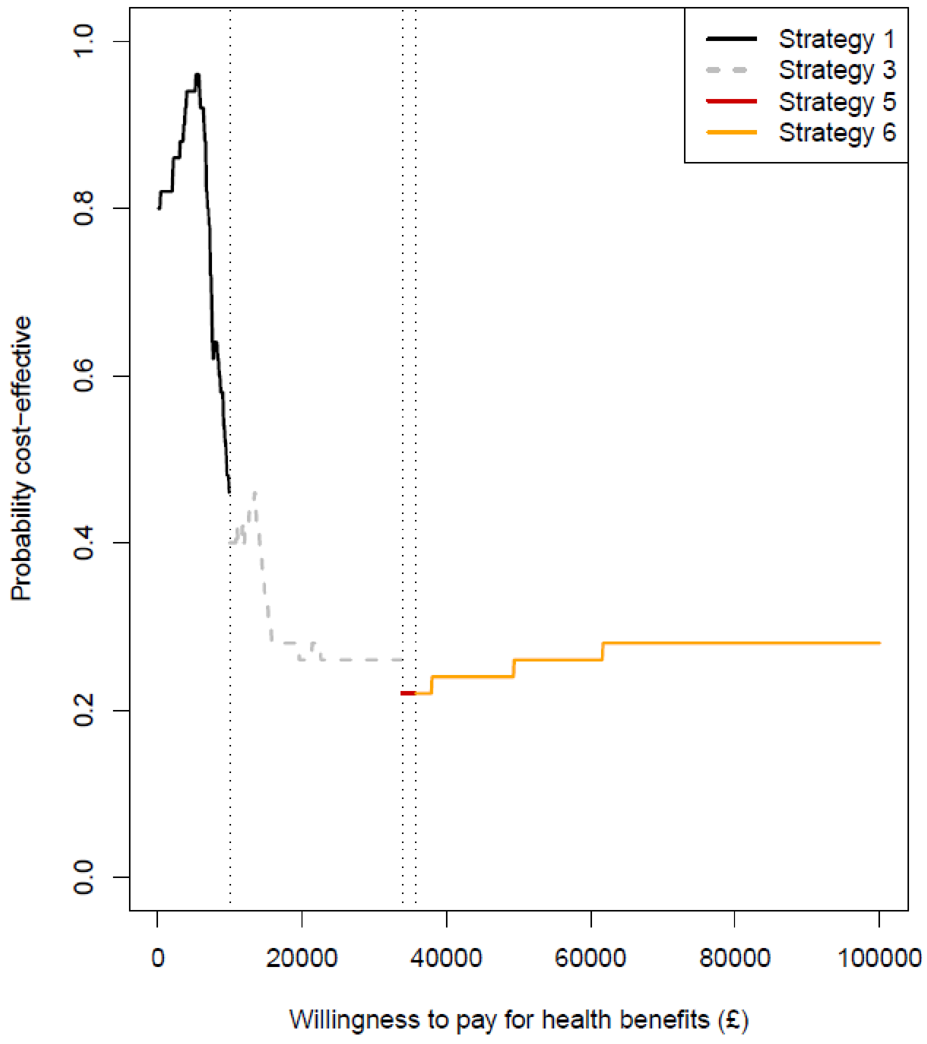

2. Scenario analyses

The following results explore each of the strategies in an Acute Trust setting in a series of scenario analyses, namely: i) a high prevalence setting (where prevalence is twice that found in the audit for Acute Trusts: 2.8% compared to 1.4%); ii) a low prevalence setting (where the prevalence is half that found from the audit for Acute Trusts: 0.7% compared to 1.4%); iii) a setting where transmission in high risk specialties is reduced to be between ICU and general medical ward transmission probabilities (see appendix 4 table 6); and iv) a setting where the probability of death is homogenous across the hospital(see appendix 4 Table 4) .

High prevalence setting

Figure A8 assesses the screening strategies ability to impose control measures on MRSA positive patients. The pattern seen is the same as that in the baseline prevalence setting (Figure A1). However, overall the number of MRSA positive isolated bed days per 100 bed days is greater, as there are more MRSA positive patients to isolate, and more unisolated bed days, as more MRSA positive patients means a greater number will be missed. Interestingly, in the higher prevalence setting there is around the same level of inappropriate isolation, this may be explained through the positive predictive value of the test – even though overall more patients will be isolated (and therefore more also inappropriately isolated) this will be offset as the chance of a false positive will be reduced.

The greater prevalence on admission leads to a slightly greater level of transmission (due to more infectious imports to the hospital) and, in turn, slight increases in the absolute numbers of infections and deaths (Figure A9).

Differences in costs between the higher prevalence setting and baseline prevalence setting are minimal (Figure A10), and when costs and effects are combined on the cost-effectiveness plane it can be seen that overall health benefits per cost accrued are similar to those at a baseline prevalence setting (Figure A11 compared to Figure A4). Overall, strategies provide slightly greater health benefits, as they are able to prevent more transmission events, leading to fewer infections and therefore deaths. While strategy 6 (screening all patients plus pre-emptive isolation of those known to have previously been

MRSA positive) appears to do less well than strategy 2 (screening all patients) in this setting, it can be seen from the error bars that there is considerable uncertainty and overlap between the two policies. This uncertainty is dealt with in the CEAC and CEAF plots.

Figure A8. Primary isolation usage under each screening strategy, showing appropriate isolation (isolation of patients who are MRSA positive), inappropriate isolation (isolation of MRSA negative patients) and unisolated bed days of MRSA positive patients.

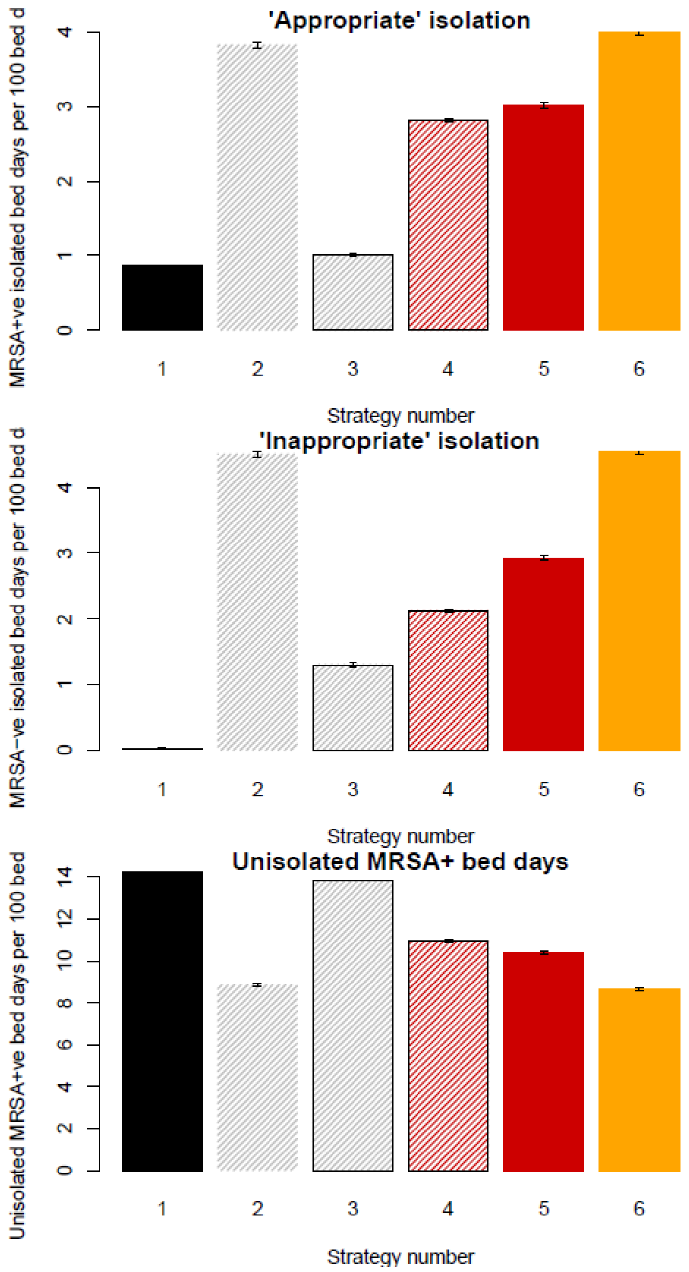

Figure A9. Patient outcomes under each screening strategy, showing new acquisitions of MRSA by hospital patients, total MRSA infections in the hospital, and total deaths (all per 100 admissions).

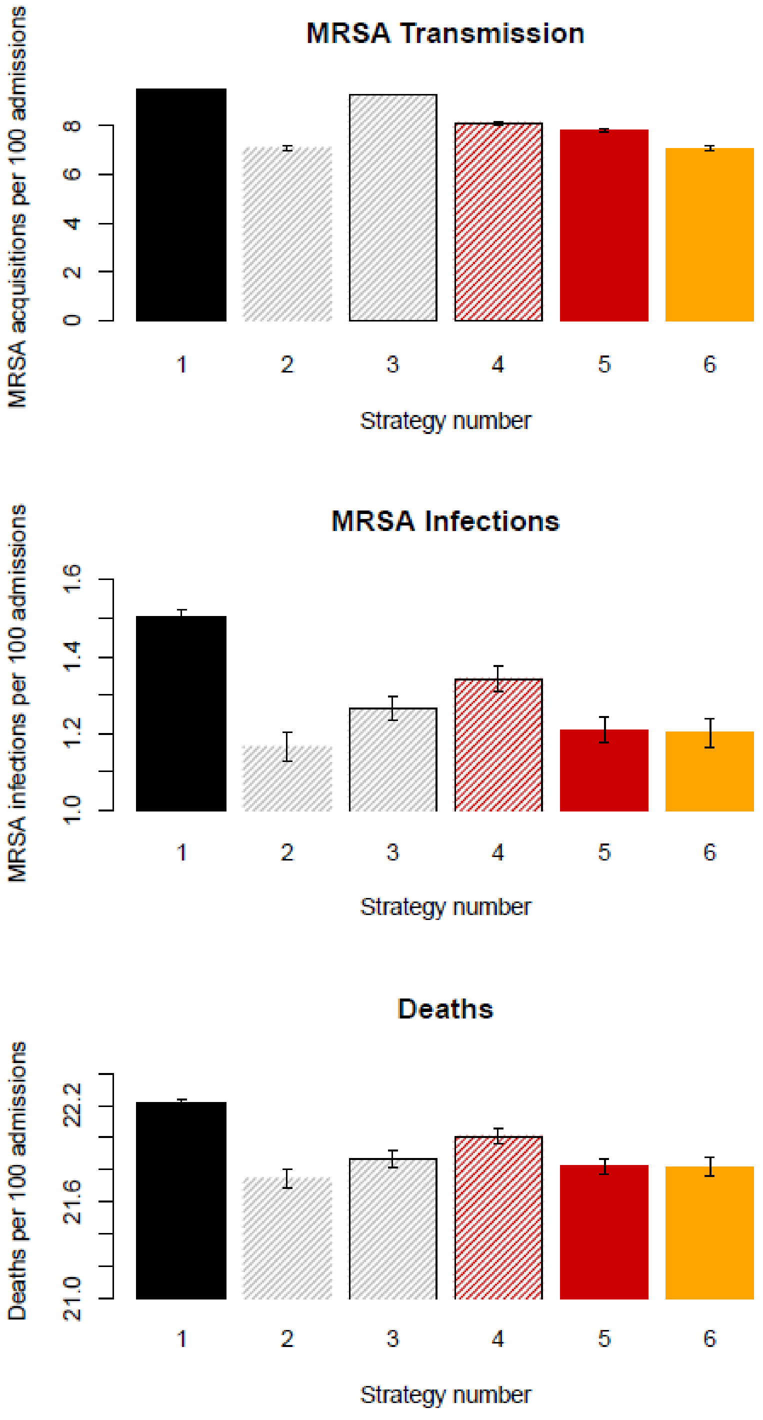

Figure A10. Cost per admission for each strategy, presented as total costs and broken down into component parts (note different scales of sub graphs).

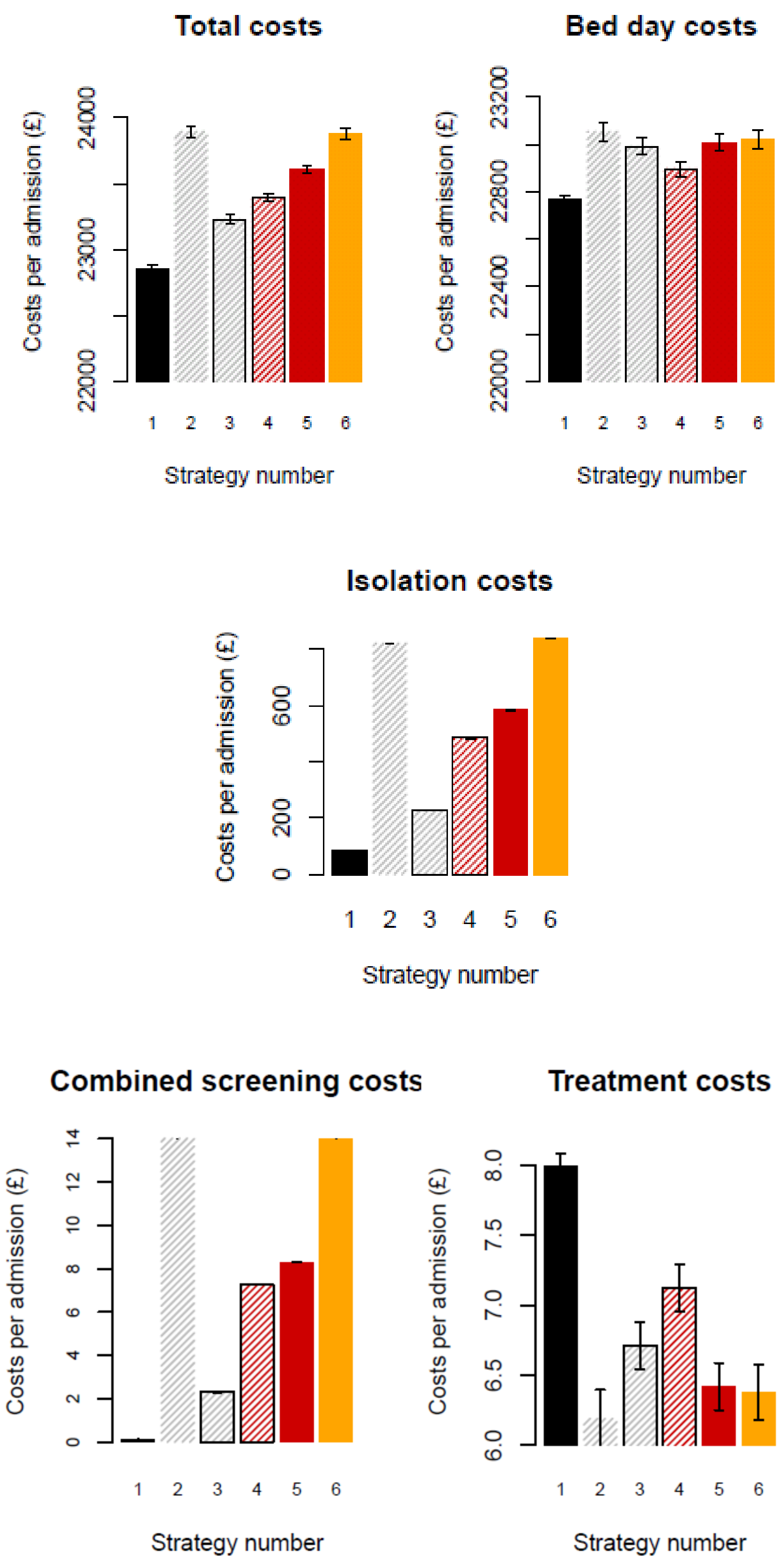

Figure A11. Incremental cost-effectiveness plot comparing each of the screening strategies. Numbers indicate strategy numbers as outlined above. Error bars represent random error brought about by stochasticity in the model and parameter uncertainty, and correspond to plus or minus one standard error.

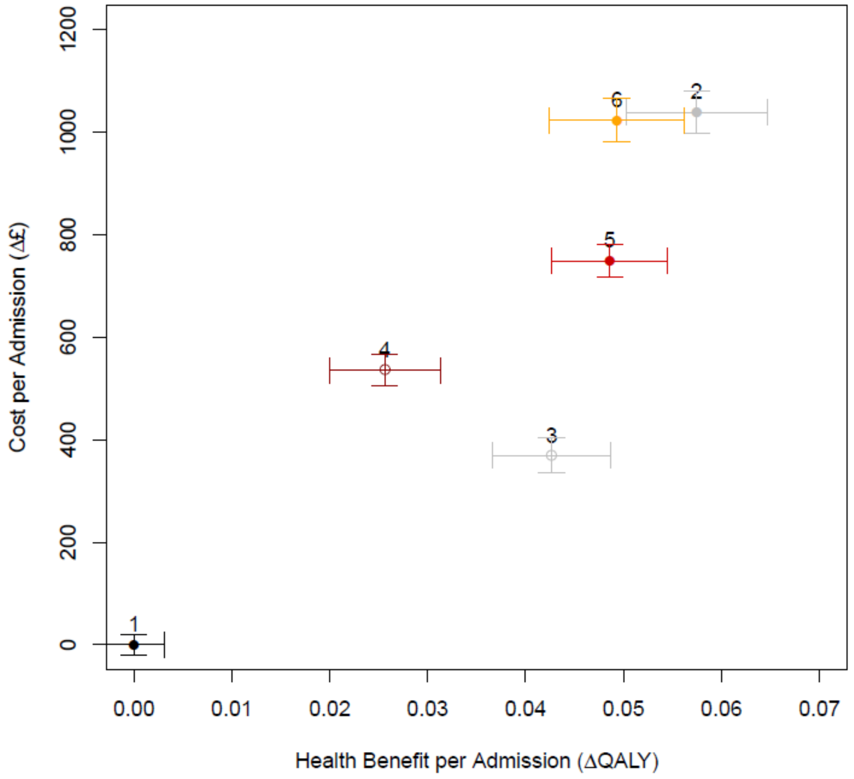

Evaluation of the cost-effectiveness frontier shows that despite the ICERs shifting slightly, the decision remains the same in the higher prevalence setting. With strategy 3 (screening of patients admitted to high risk specialties) the only cost-effective option (at a willingness to pay threshold of £30,000/QALY gained) (Table A3).

Table A3. Evaluation of cost-effectiveness frontier of strategies for an Acute Trust in a high prevalence setting, using a willingness to pay threshold of £30,000/QALY gained.

Move between policies	Cost per admission	QALY per admission	Change in costs, ΔC (compared to previous policy)	Change in effects, ΔE (compared to previous policy)	Difference in Costs / Difference in Effects (ICER, cost/QALY)	Option evaluation
Stay at policy 1	£22,858	7.990682	-	-	-	-
Or move:						
to 3	£23,227	8.033346	£369.03	0.042664	£8,650	Cost effective
to 5	£23,897	8.048153	£670.14	0.014807	£45,257	Not cost effective

Very similar levels of uncertianty in the decision between strategies can be seen in a higher prevalence setting (Figure A12), compared to the baseline prevalence setting (Figure A6). However at higher willingness to pay values (greater than approximately £30,000/QALY), strategy 2, screening all patients, has a greater probability of being cost-effective compared to the competing options.

Figure A12. Cost-effectiveness acceptability curves. Each line represents the proportion of simulations, for a particular strategy, that are cost-effective, as a function of willingness to pay for health benefits.

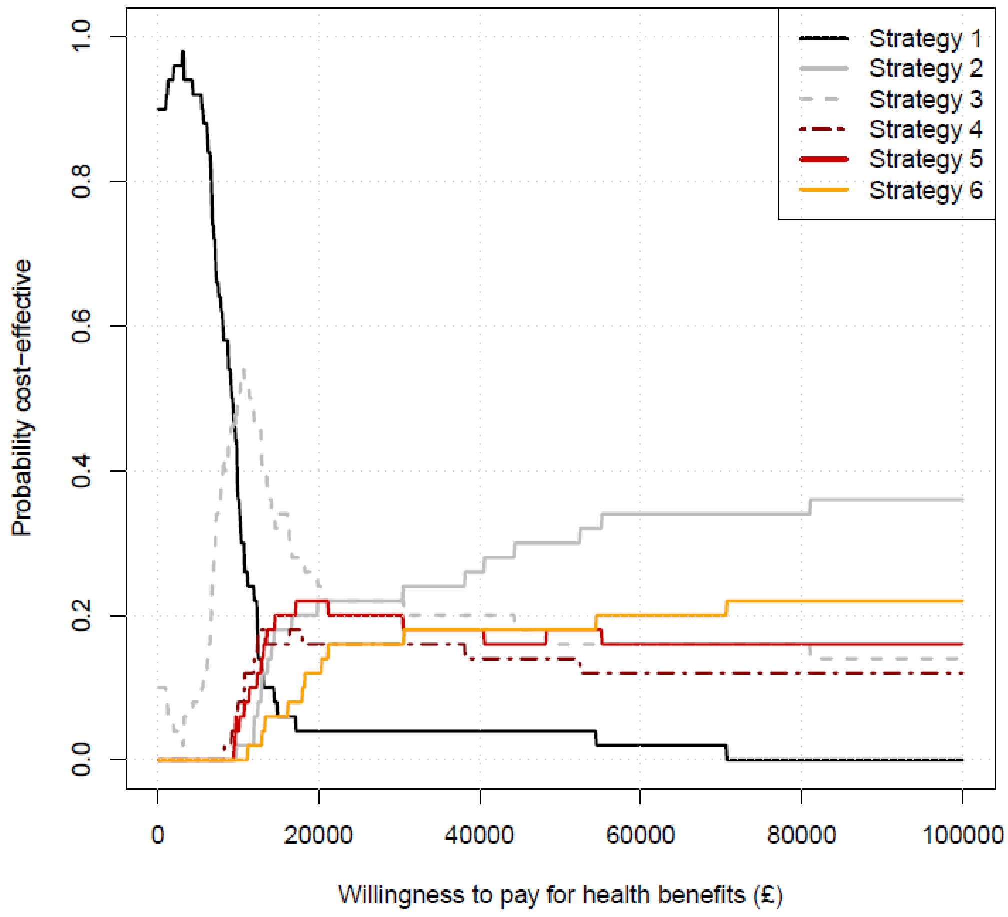

The CEAF (Figure A13) demonstrates that, as in the baseline prevalence setting, strategy 3 (screening all patients admitted to high risk specialties) is optimal within the usual NHS willingness to pay range of £20,000-£30,000/QALY. As the CEAC indicated (Figure A12), only at higher willingness to pay values does this decision change: above approximately £45,000/QALY screening all patients on admission becomes optimal.

Figure A13. Cost-effectiveness acceptability frontier. Lines depict the strategies with the highest expected net monetary benefit, dependent on the willingness to pay for health benefits, while dotted vertical lines the willingness to pay values at which the decision changes.

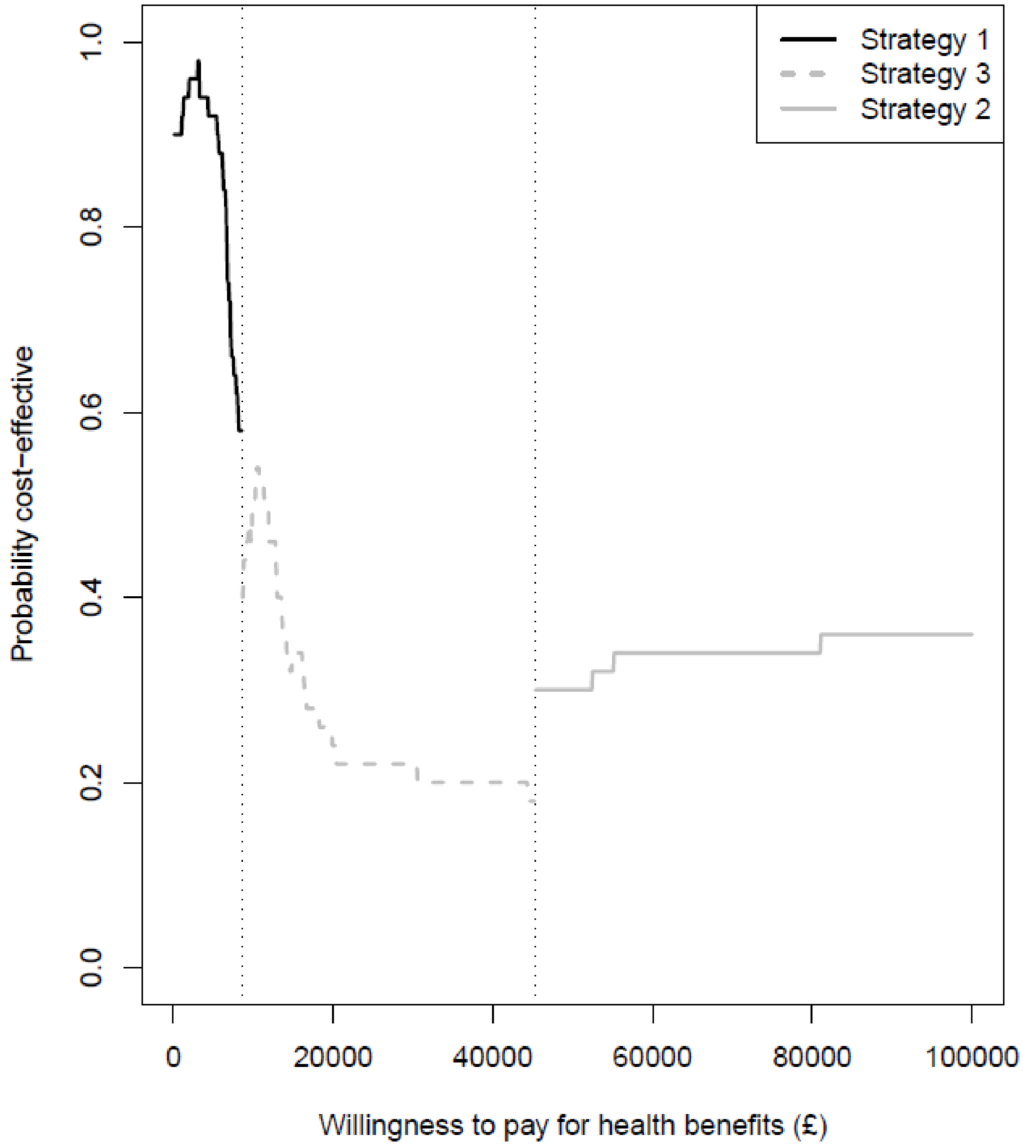

Low prevalence setting

Figure A14 shows that the overall pattern of the ability of strategies to identify MRSA positive patients and impose control remains the same in a low prevalence setting, compared to the baseline prevalence setting (Figure A1). As might be expected, as there are fewer MRSA positive patients admitted to the hospital, overall there is less isolation and fewer unisolated patient days. However, the degree of inappropriate isolation increases slightly with a lower admission prevalence due to the lower positive predictive value of the test in this setting.

Again, while comparison between policies remains the same, in a lower prevalence setting there is less within hospital transmission, leading to fewer infections and deaths, due to a lessened infectious assault on the hospital (Figure A15).

Interestingly, costs associated with each of the screening strategies are very slightly higher in a low prevalence setting (Figure A16), compared to the baseline prevalence setting (Figure A3), by approximately £200 per admission). This is due to the fact that in a lower prevalence setting for the same level of screening effort the yield will be less, meaning the screening costs will not be offset to the same extent by cost savings brought about though reduction in transmission (and therefore infections).

Figure A14. Primary isolation usage under each screening strategy, showing appropriate isolation (isolation of patients who are MRSA positive), inappropriate isolation (isolation of MRSA negative patients) and unisolated bed days of MRSA positive patients.

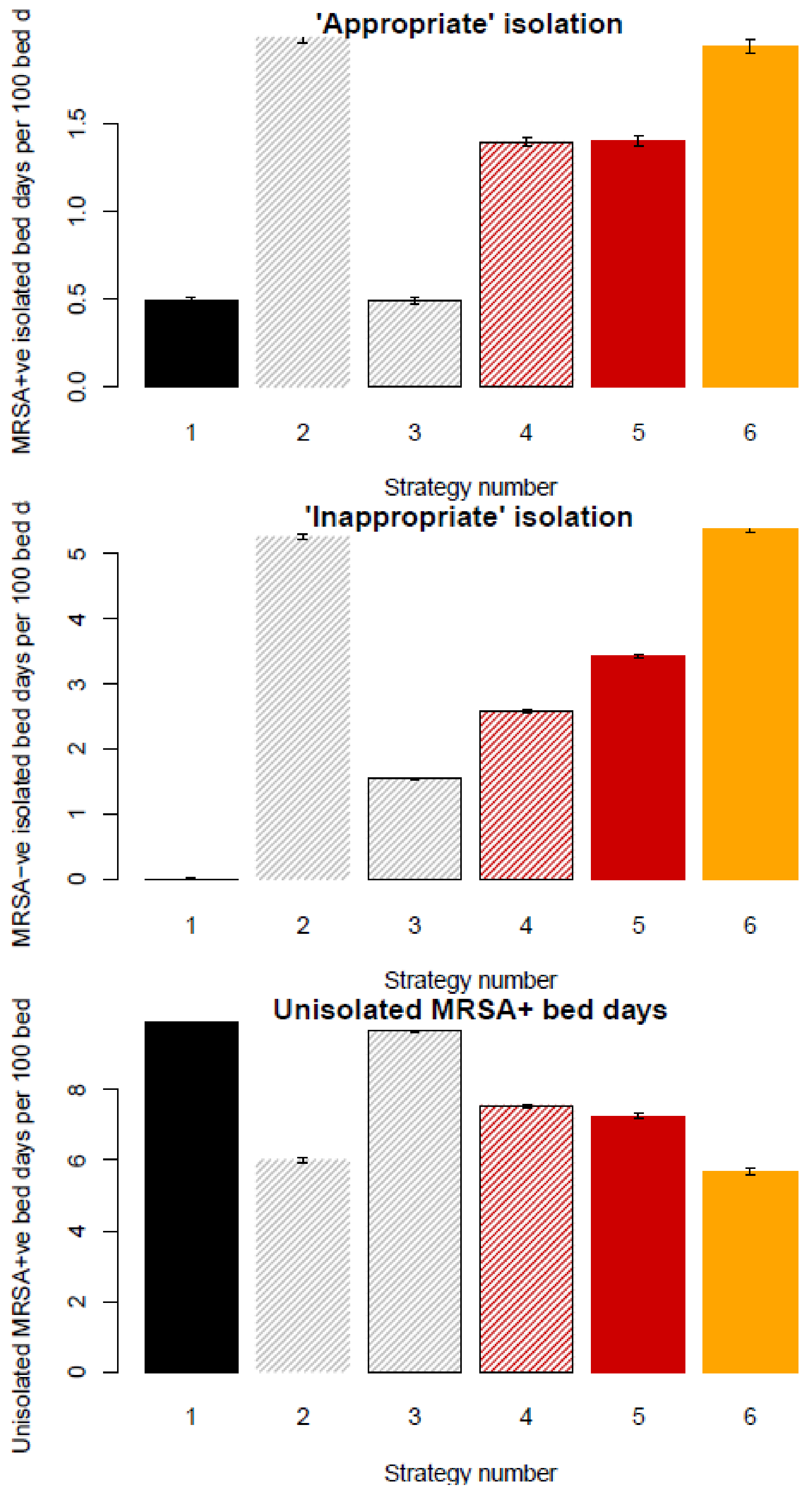

Figure A15. Patient outcomes under each screening strategy, showing new acquisitions of MRSA by hospital patients, total MRSA infections in the hospital, and total deaths (all per 100 admissions).

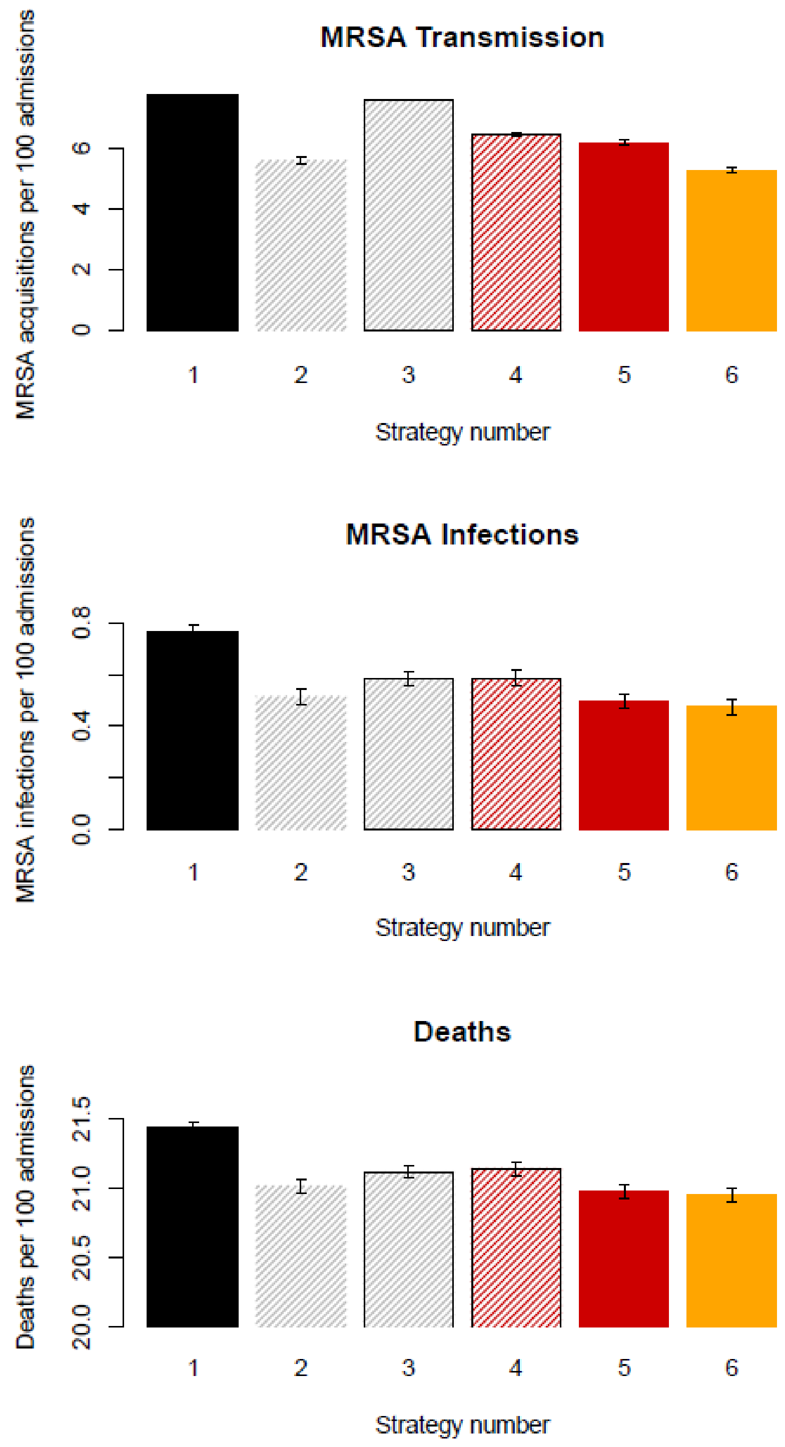

Figure A16. Cost per admission for each strategy, presented as total costs and broken down into component parts (note different scales of sub graphs).

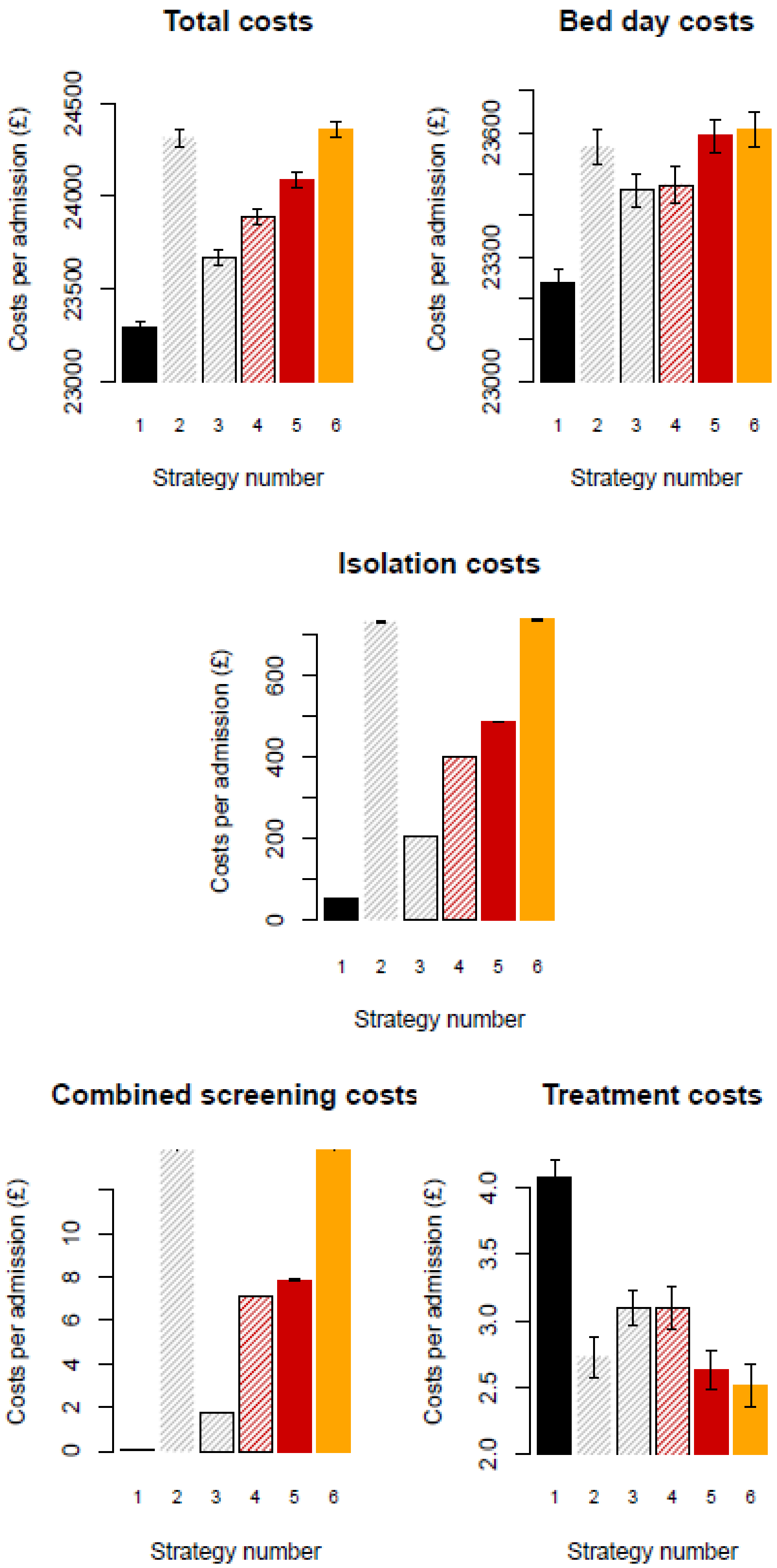

On the cost-effectiveness plane (Figure A17) the ordering of strategies remains the same under a lower prevalence. ICERs, as described in the evaluation of the cost-effectiveness frontier (Table A4) change only slightly with strategy 3 (screening of patients admitted to high risk specialties) remaining cost-effective. However, in a low prevalence setting, checklist activated screening in addition to screening all admissions to high risk specialties (strategy 5) becomes a cost-effective option.

Figure A17. Incremental cost-effectiveness plot comparing each of the screening strategies. Numbers indicate strategy numbers. Error bars represent random error brought about by stochasticity in the model and parameter uncertainty, and correspond to plus or minus one standard error.

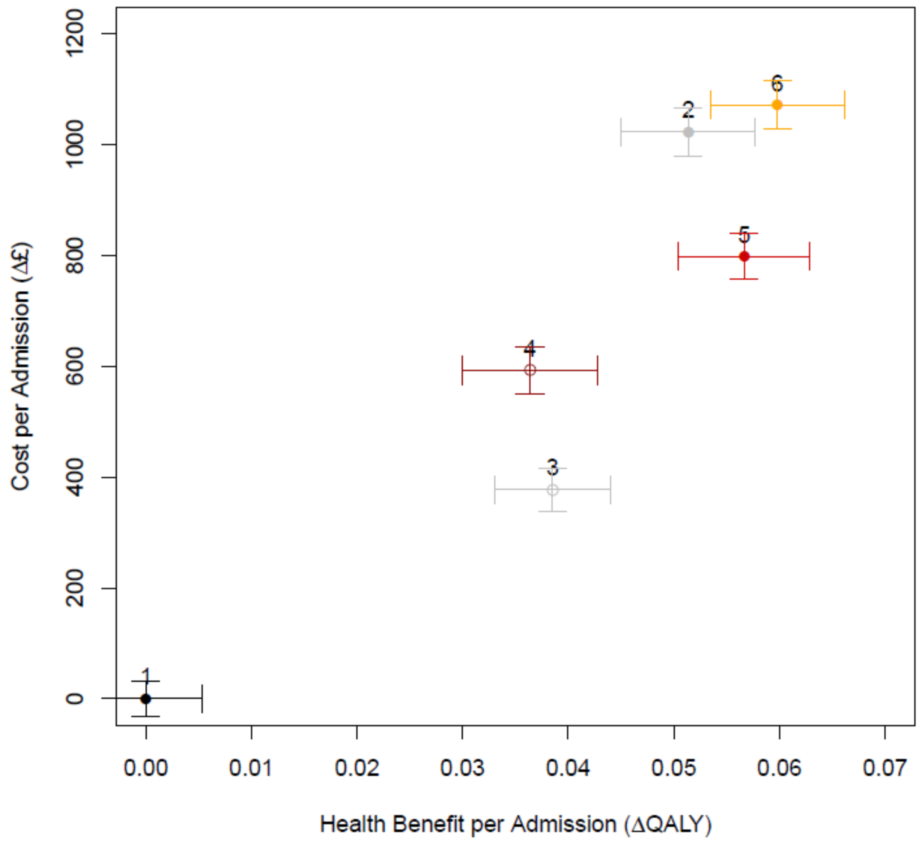

Table A4. Evaluation of cost-effectiveness frontier of strategies for an Acute Trust in a high prevalence setting, using a willingness to pay threshold of £30,000/QALY gained.

Move between policies	Cost per admission	QALY per admission	Change in costs, ΔC (compared to previous policy)	Change in effects, ΔE (compared to previous policy)	Difference in Costs / Difference in Effects (ICER, cost/QALY)	Option evaluation
Stay at policy 1	£23,292	8.090747	-	-	-	-
Or move:						
to 3	£23,670	8.12928	£377.47	0.038533	£9,796	Cost effective
to 5	£24,090	8.147422	£420.83	0.018142	£23,196	Cost effective
to 6	£24,364	8.150547	£273.41	0.003124	£87,517	Not cost effective

However, again, as demonstrated in the CEAC (Figure A18) there is considerable uncertainty in the decision, where within the NHS willingness to pay range no strategy has more than a 30% chance of being cost-effective. From the CEAF (Figure A19) it can be seen that the decision varies within the NHS range: screening patients admitted to high risk specialties (strategy 3) being optimal at the lower end of the willingness to pay range, and adding checklist activated screening of patients in low risk settings to this policy (strategy 5) at the higher end of the range.

Figure A18. Cost-effectiveness acceptability curves. Each line represents the proportion of simulations, for a particular strategy, that are cost-effective, as a function of willingness to pay for health

benefits.

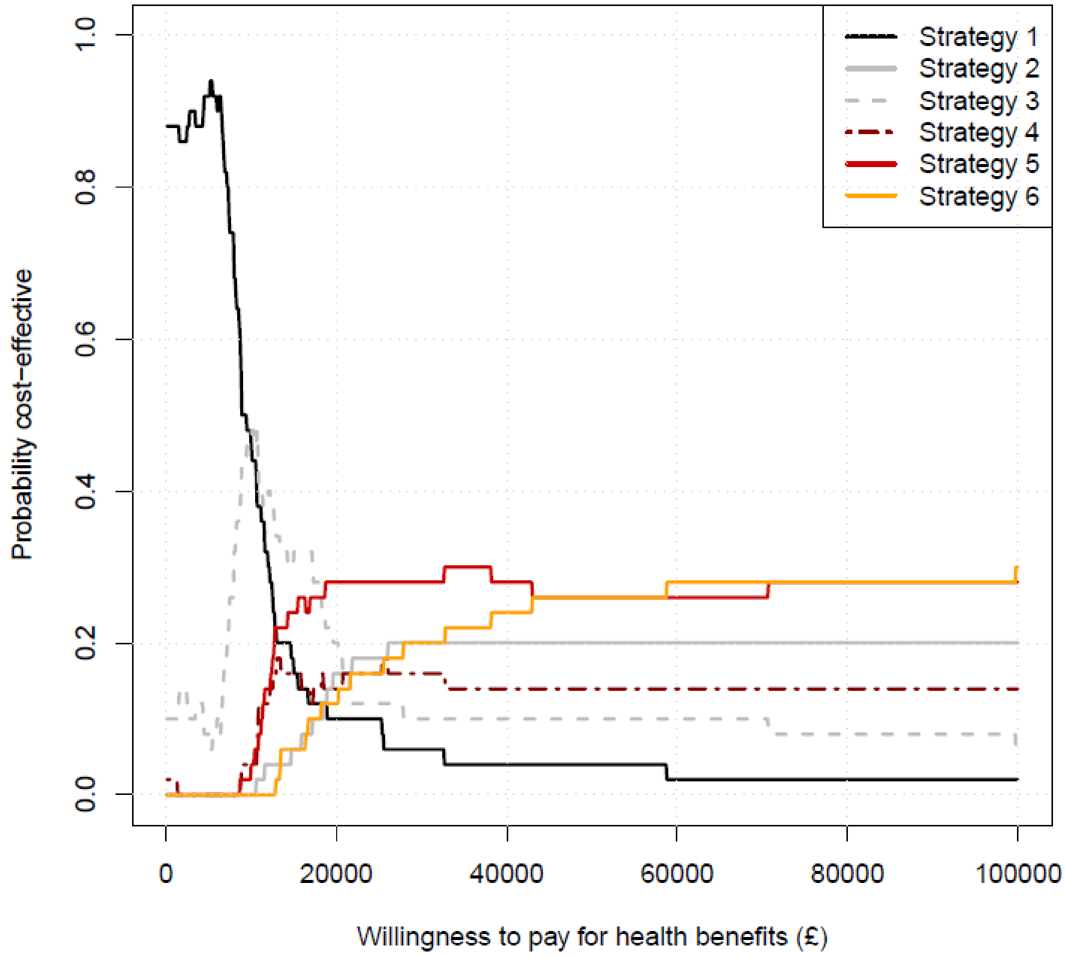

Figure A19. Cost-effectiveness acceptability frontier. Lines depict the strategies with the highest expected net monetary benefit, dependent on the willingness to pay for health benefits, while dotted vertical lines the willingness to pay values at which the decision changes.

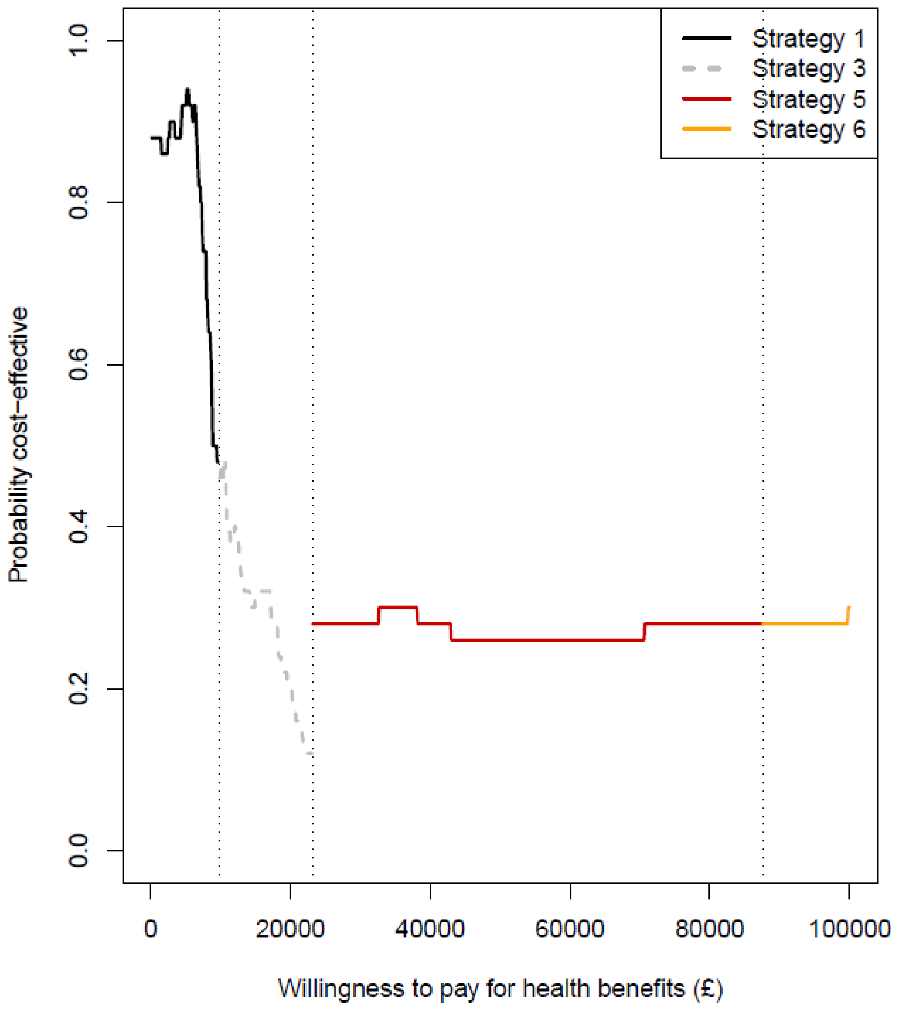

Low transmission in high risk specialties

A change in the degree of onward transmission (and probability of infection) within the hospital itself has no effect on the ability of each screening strategy to effectively isolate colonised patients, as screening and identification of colonisation occurs on admission to hospital (Figure A20). The slightly greater numbers of appropriate isolation events seen in this setting of lower transmission/infection compared to that at baseline transmission (Figure A1) is due to fewer numbers of infections and therefore fewer infected patients identified (and subsequently isolated) via clinical samples (as opposed to screens).

It can be seen from Figure A21 (compared to Figure A2 for the baseline transmission setting) that the reduction in transmission parameters in high risk settings has the most marked difference on the overall number of infections (which are approximately halved) and, in turn, deaths.

Total costs for the strategies (Figure A22) are greater than under baseline transmission parameters (Figure A3) as costs are not offset to the same degree by savings through reductions in infections.

From the cost-effectiveness plane (Figure A23)it can be seen that compared to the baseline strategy of no screening , none of the screening strategies provide the same gains in health benefits as are achieved in a setting with increased transmission in high risk specialties (Figure A4). However, despite a higher ICER, strategy 3 (screening all patients admitted to high risk specialties) is still cost-effective at £12,382/QALY (Table A5).

Figure A20. Primary isolation usage under each screening strategy, showing appropriate isolation (isolation of patients who are MRSA positive), inappropriate isolation (isolation of MRSA negative patients) and unisolated bed days of MRSA positive patients.

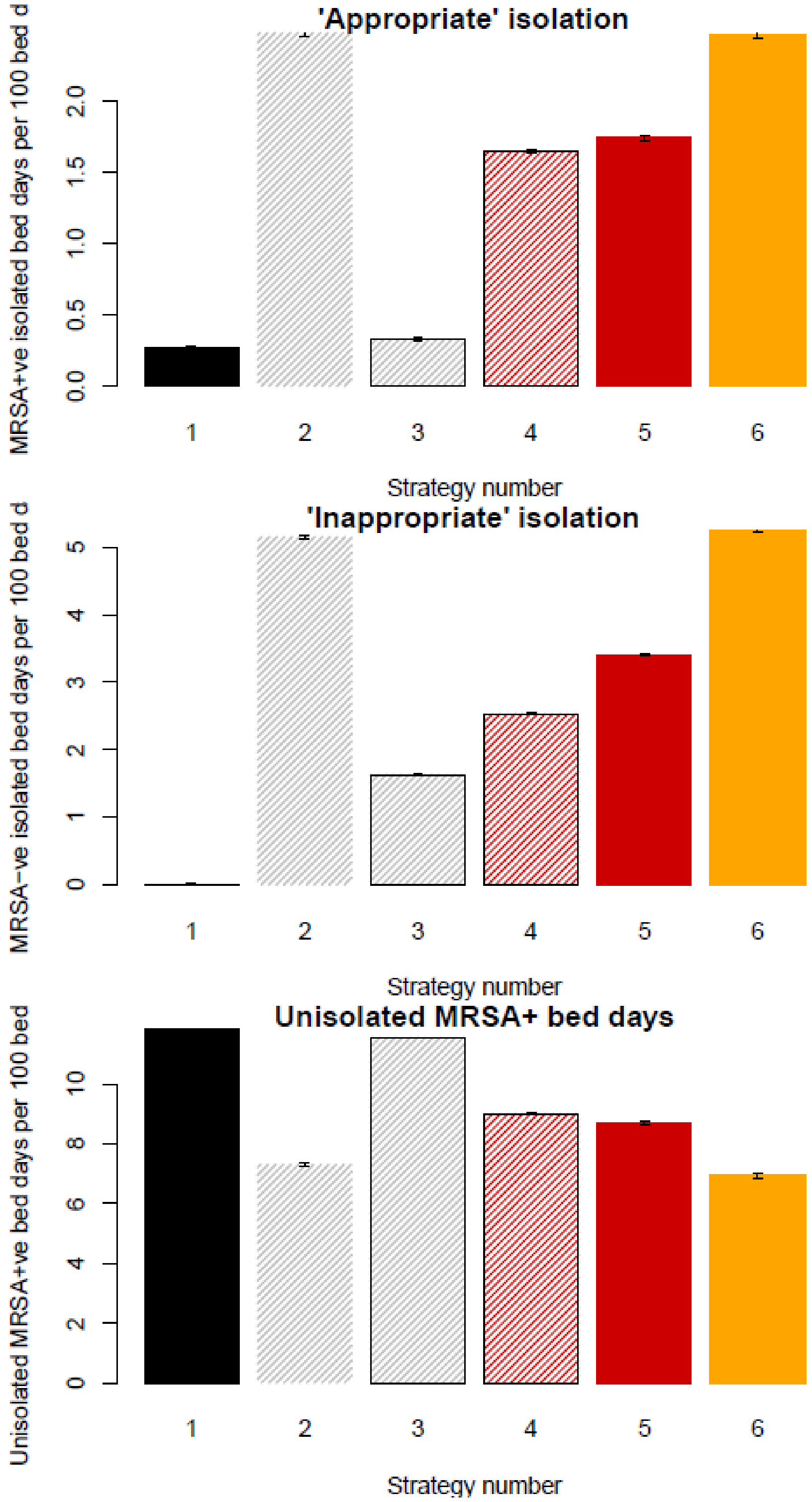

Figure A21. Patient outcomes under each screening strategy, showing new acquisitions of MRSA by hospital patients, total MRSA infections in the hospital, and total deaths (all per 100 admissions).

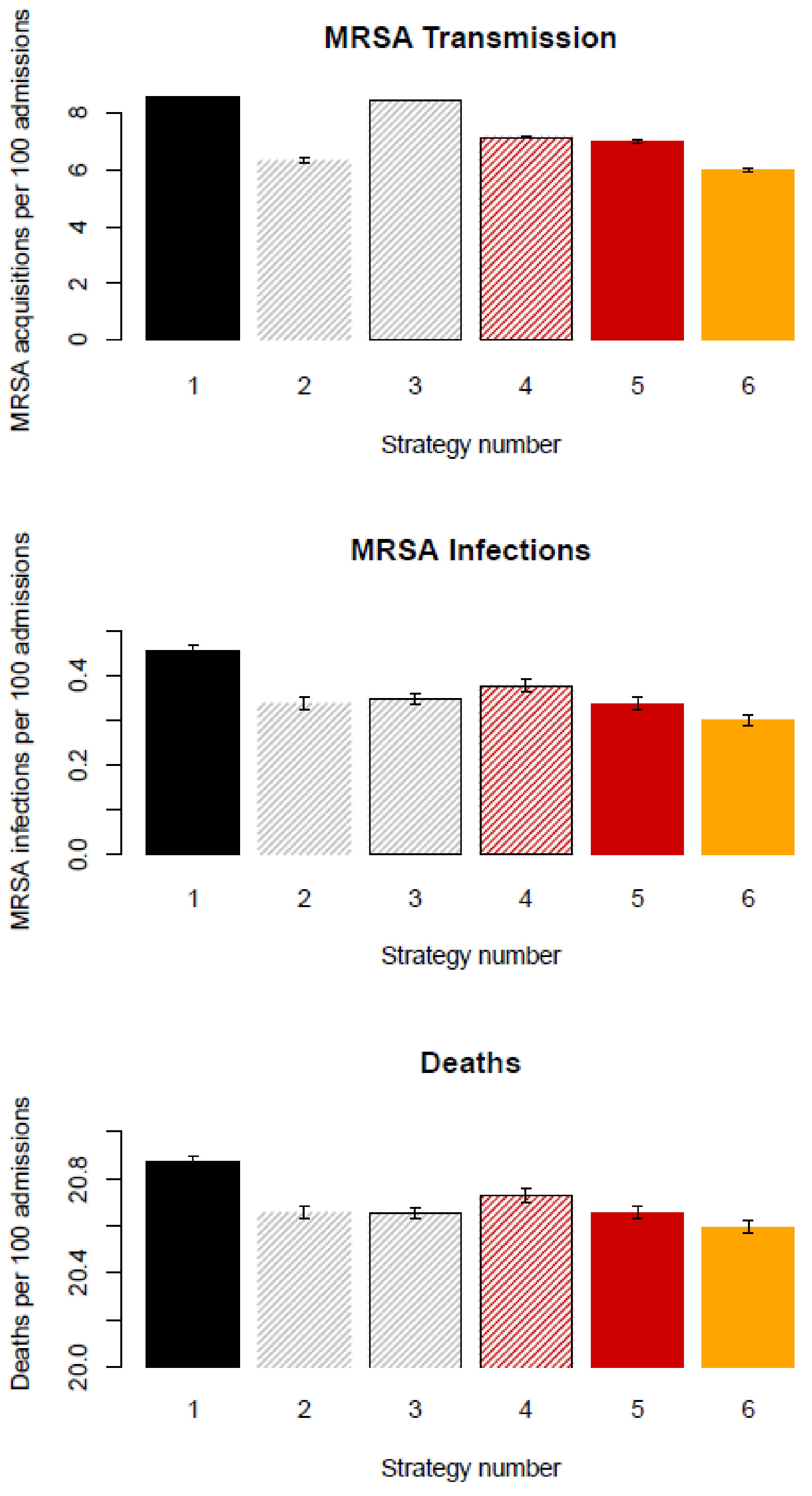

Figure A22. Cost per admission for each strategy, presented as total costs and broken down into component parts (note different scales of sub graphs).

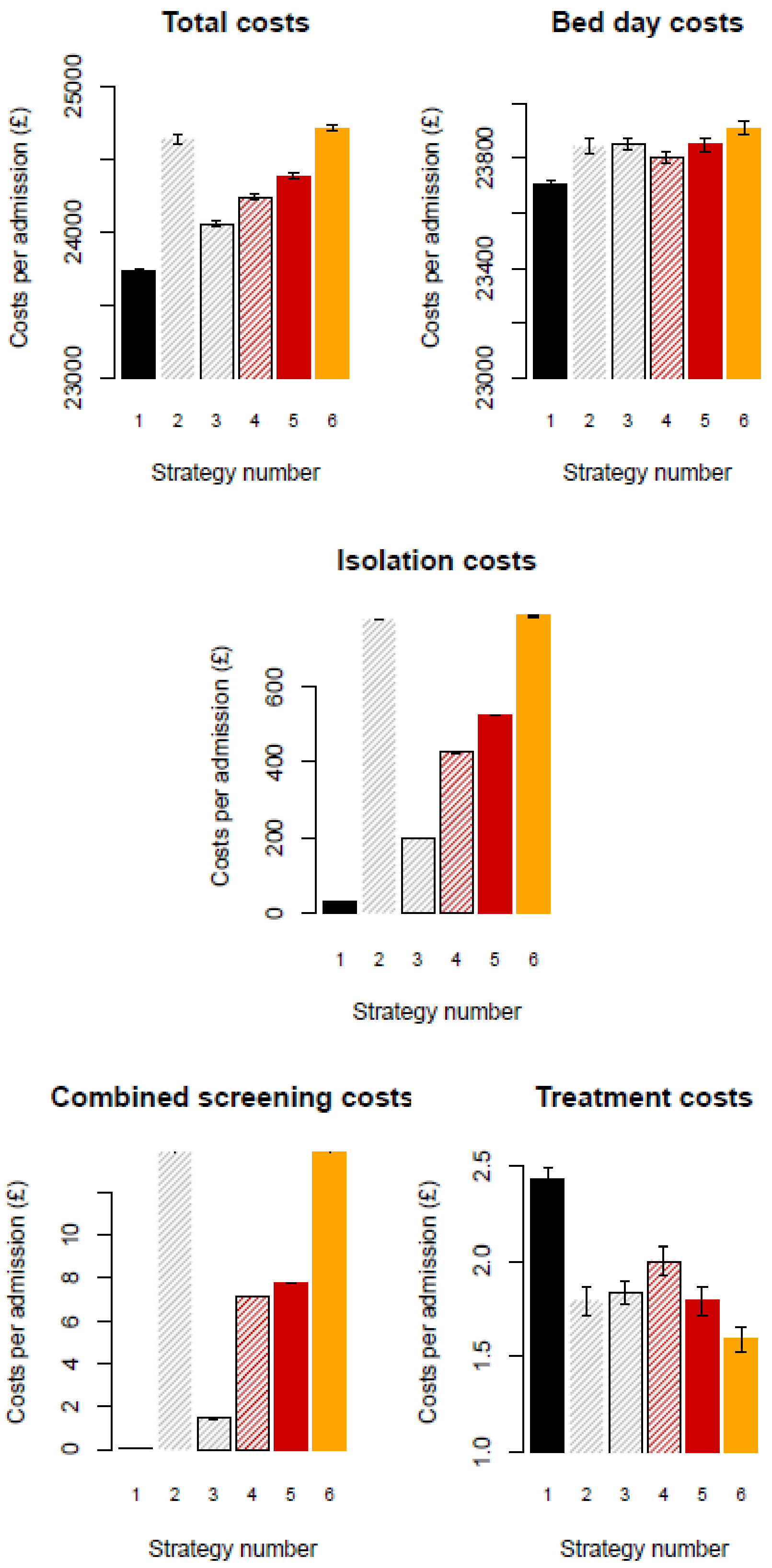

Figure A23. Incremental cost-effectiveness plot comparing each of the screening strategies. Numbers indicate strategy numbers as outlined above. Error bars represent random error brought about by stochasticity in the model and parameter uncertainty, and correspond to plus or minus one standard error.

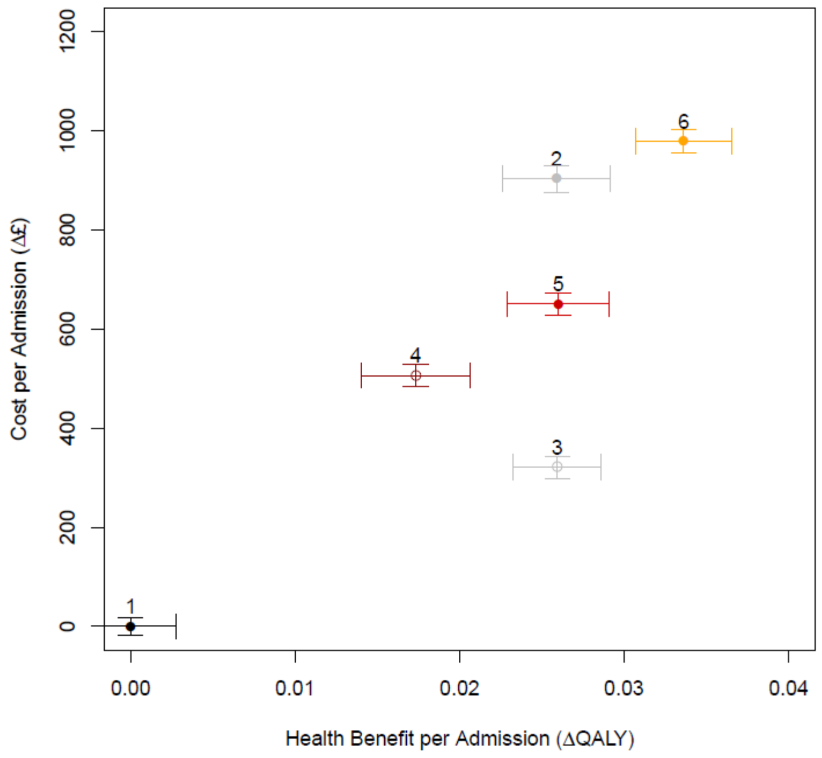

Table A5. Evaluation of cost-effectiveness frontier of strategies for an Acute Trust in a high prevalence setting, using a willingness to pay threshold of £30,000/QALY gained.

Move between policies	Cost per admission	QALY per admission	Change in costs, ΔC (compared to previous policy)	Change in effects, ΔE (compared to previous policy)	Difference in Costs / Difference in Effects (ICER, cost/QALY)	Option evaluation
Stay at policy 1	£23,733	8.159388	-	-	-	-
Or move:						
to 3	£24,054	8.185329	£321.22	0.025942	£12,382	Cost effective
to 6	£24,712	8.193005	£657.88	0.007675	£85,713	Not cost effective

The CEAC (Figure A24) shows strategy 3 (screening admissions to high risk specialties) to have the greatest probability of being cost-effective within the NHS willingness to pay range, compared to the competing strategies – with an almost 60% of being cost-effective. This is reflected in the CEAF (Figure A25) where strategy 3 is the optimal option from willingness to pay values of just over £10,000/QALY to nearly £90,000/QALY.

Figure A24. Cost-effectiveness acceptability curves. Each line represents the proportion of simulations, for a particular strategy, that are cost-effective, as a function of willingness to pay for health benefits.

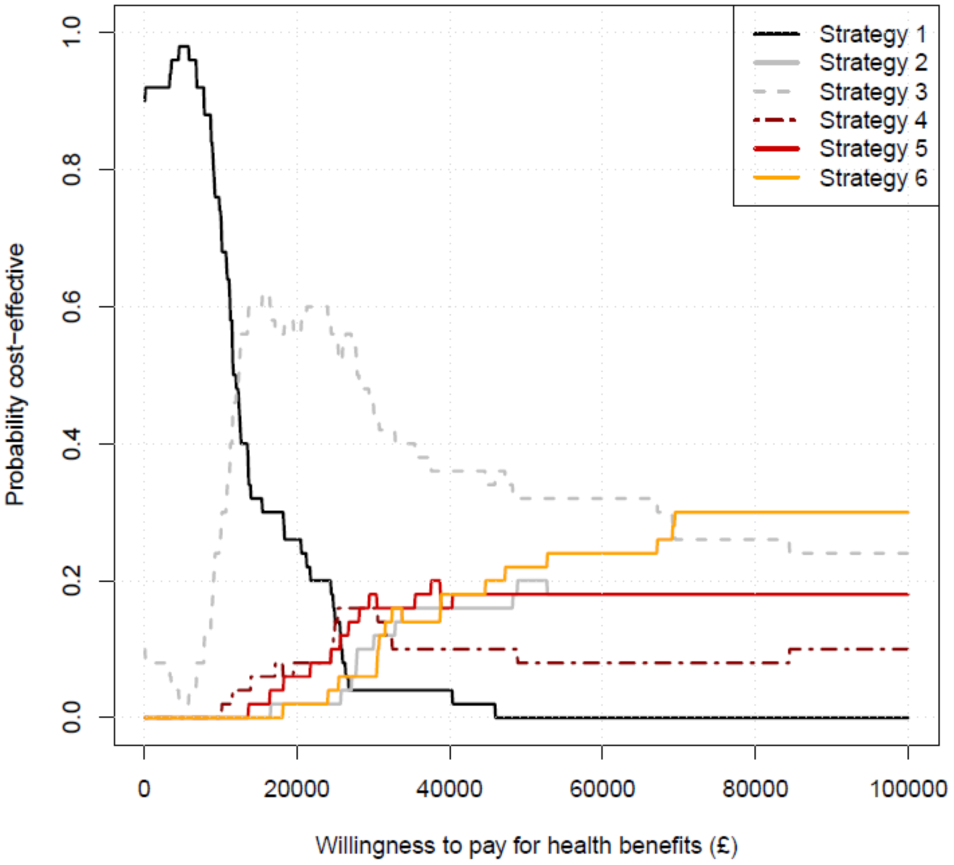

Figure A25. Cost-effectiveness acceptability frontier. Lines depict the strategies with the highest expected net monetary benefit, dependent on the willingness to pay for health benefits, while dotted vertical lines the willingness to pay values at which the decision changes.

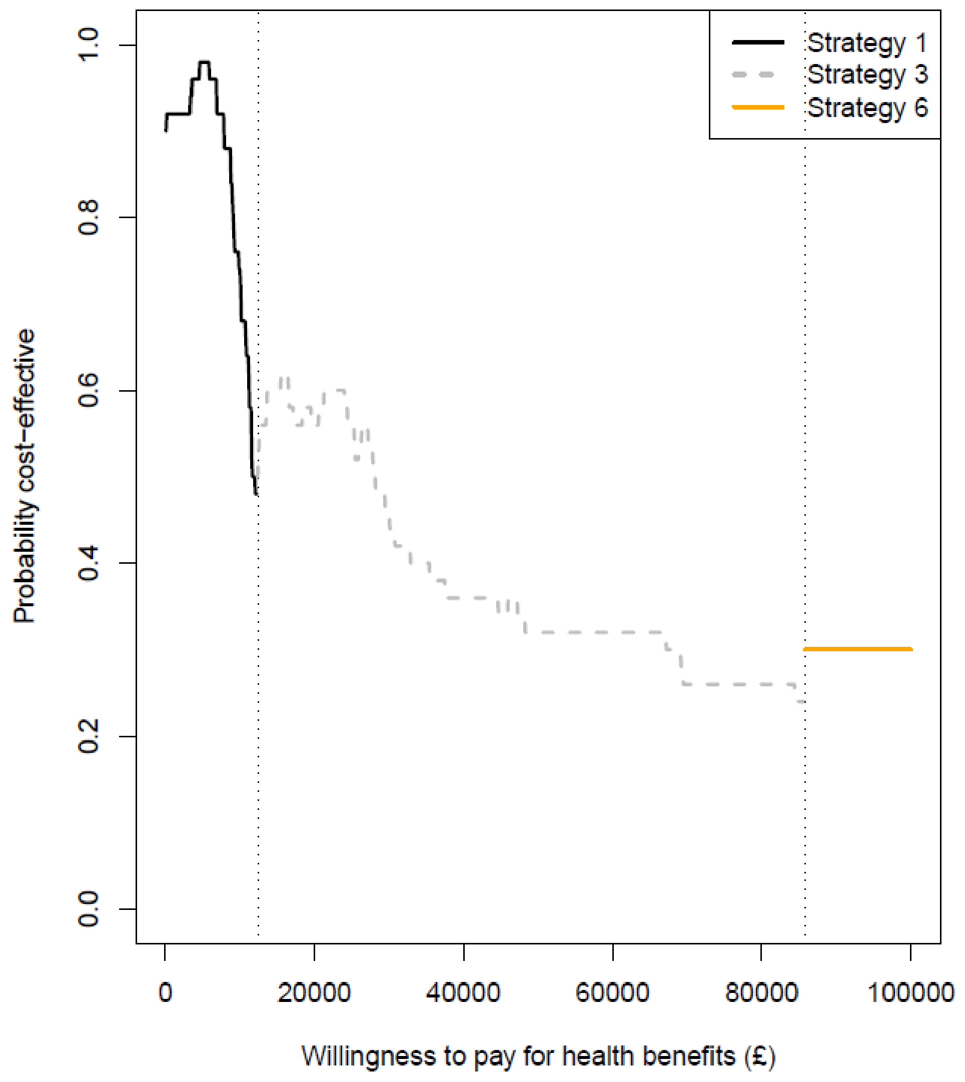

Homogenous probability of death across all specialties

In a setting where the daily probability of death in high risk specialties is reduced to be equivalent to that in low risk specialties, there will be little, or no, difference to the effectiveness of interventions in terms of reduction in transmission or infection events. The differences arise due to differences in long term QALY accrual, as death events prevent patients being discharged successfully and going on to accrue QALYs post discharge. We therefore present results, and compare policies, in terms of costs per QALY gained only.

As, under the revised death parameters, prevention of infections has less impact on deaths in high risk specialties, each of the screening strategies has a reduced ability to prevent death events and therefore reduced ability to gain health benefits (QALYs). As can be seen from the incremental cost-effectiveness plot (Figure A26) the health benefits gained under each of the strategies is reduced by approximately 10-fold (compared to baseline death parameters, Figure A4). This, in turn, is translated to the ICER values, where cost/QALY values are much greater (Table A6 compared to Table A2).

However, even with these conservative assumptions around mortality in high risk specialties, strategy 3 remains cost-effective at £26,551/QALY. Indeed, the CEAC (Figure A27) depicts strategy 3 to have an approximately 60% probability of being cost-effective within the NHS willingness to pay threshold. The CEAF (Figure A28), which accounts for the magnitude of the potential benefit as well as the probability of cost-effectiveness, shows the decision changes dependent on willingness to pay. At the lower end of the NHS willingness to pay range the baseline ‘no screening’ approach is optimal, up to approximately £25,000/QALY, while strategy 3 becomes optimal above this value.

Figure A26. Incremental cost-effectiveness plot comparing each of the screening strategies. Numbers indicate strategy numbers as outlined above. Error bars represent random error brought about by stochasticity in the model and parameter uncertainty, and correspond to plus or minus one standard error.

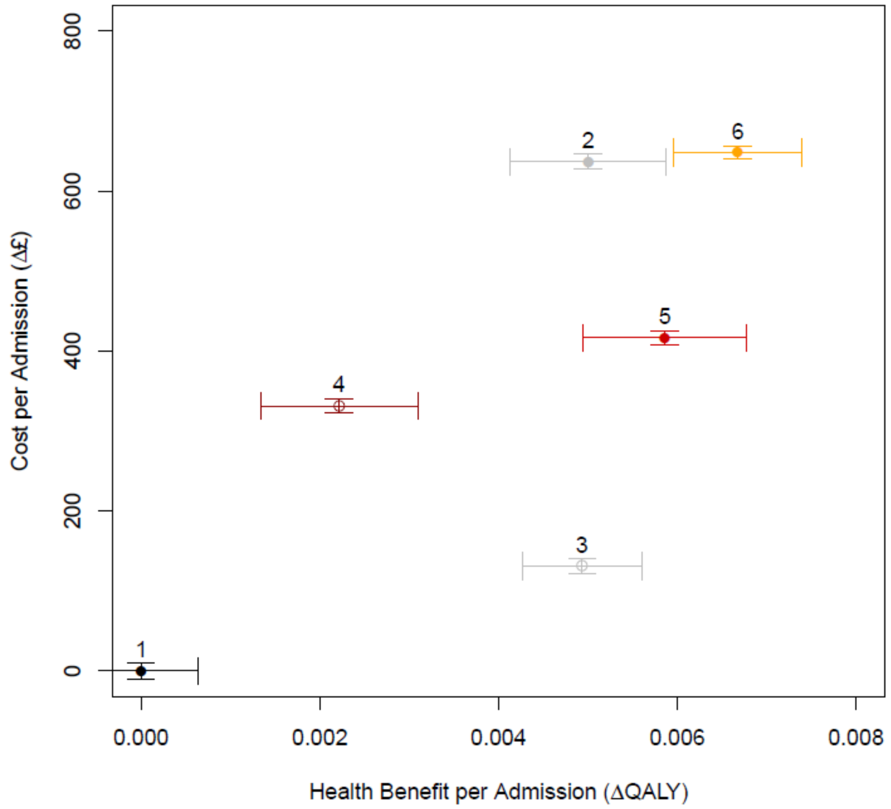

Table A6. Evaluation of cost-effectiveness frontier of strategies.

Move between policies	Cost per admission	QALY per admission	Change in costs, ΔC (compared to previous policy)	Change in effects, ΔE (compared to previous policy)	Difference in Costs / Difference in Effects (ICER, cost/QALY)	Option evaluation
Stay at policy 1	£22,321	8.245049	-	-	-	-
Or move:						
to 3	£22,452	8.249984	£131.03	0.004935	£26,551	Cost effective
to 6	£22,969	8.251726	£517.07	0.001742	£296,859	Not cost effective

Figure A27. Cost-effectiveness acceptability curves. Each line represents the proportion of simulations, for a particular strategy, that are cost-effective, as a function of willingness to pay for health benefits.

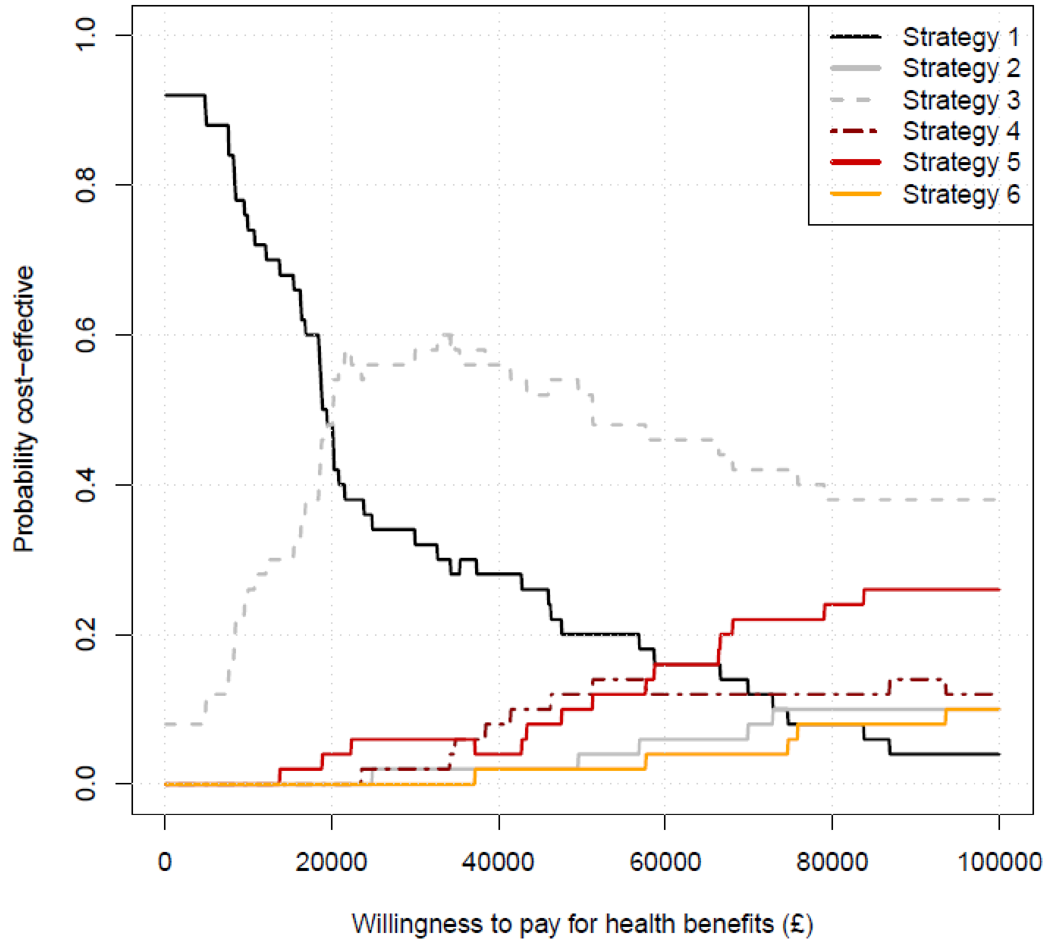

Figure A28. Cost-effectiveness acceptability frontier. Lines depict the strategies with the highest expected net monetary benefit, dependent on the willingness to pay for health benefits, while dotted vertical lines the willingness to pay values at which the decision changes.

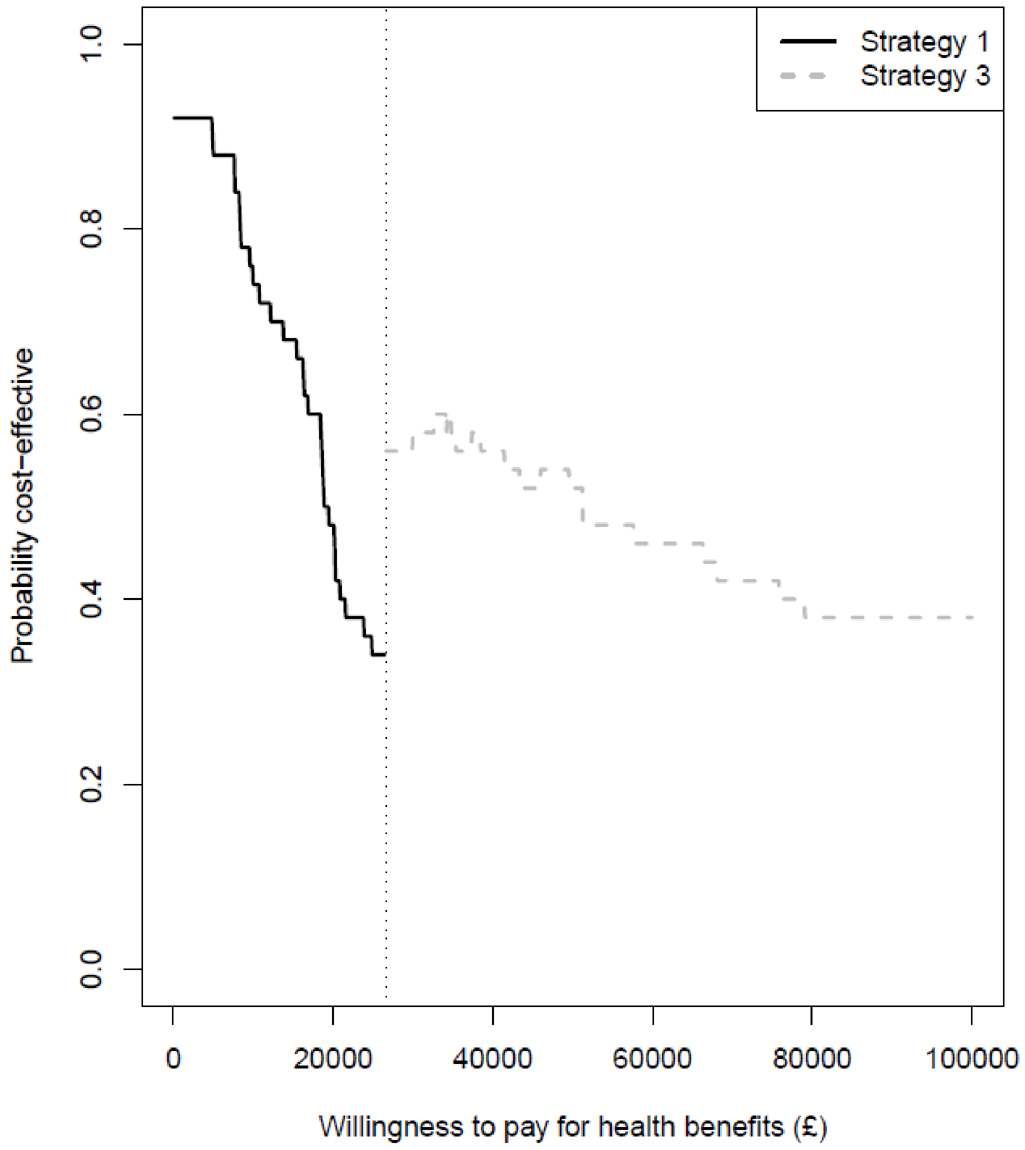

3. Comparison with Scottish Checklist

Simulation results for strategies 4 (checklist activated screening patients) and 5 (screening admissions to high risk specialities plus checklist activated screening of patients in low risk specialties), are dependent on parameters concerning proportions of checklist positive patients in various patient groups. For all simulation results in this report the definition of checklist positive s is as defined specifically for this audit, as described in appendix 12b. The model parameters affected by this definition of checklist positivity are: the proportion colonised on admission in the checklist negative patient population and in the checklist positive population; and the proportion of elective admissions classified as checklist positive as well as the proportion of emergency admissions that are classified as checklist positive.

In these scenario analyses we amend these parameters to be defined by the definition of checklist positivity as used in the Scottish Pathfinder report (Stewart 2011), rather than the audit definition of checklist positivity (see appendix 12a tables 3 & 4 and appendix 12b tables E32 & E33). This has the effect of increasing the prevalence in the checklist positive group to 5.6% (compared to 2.6% using the NOW audit definition), and decreasing the percentage of admissions classified as checklist positive (from 44% to 10.5% of elective admissions, and from 57% to 25% of emergency admissions).

Through an incremental cost-effectiveness plot (Figure A29) the differences in both costs and effects for each of the strategies can be compared in a rational way, through incremental comparison with the baseline ‘no screening’ strategy (strategy 1). As would be expected, the incremental costs (as compared to the baseline) for both strategies 4 and 5 are markedly reduced. This effect is likely to be largely due to the smaller number of patients being classified as checklist positive, and thus a reduction in numbers screened. This reduction in incremental costs is less pronounced for strategy 5 as in this strategy all high risk specialty admissions are still screened, irrespective of the definition of checklist positivity.

Figure A29. Incremental cost-effectiveness plot comparing each of the screening strategies. Numbers indicate strategy numbers as outlined above. Error bars represent random error brought about by stochasticity in the model and parameter uncertainty, and correspond to plus or minus one standard error.

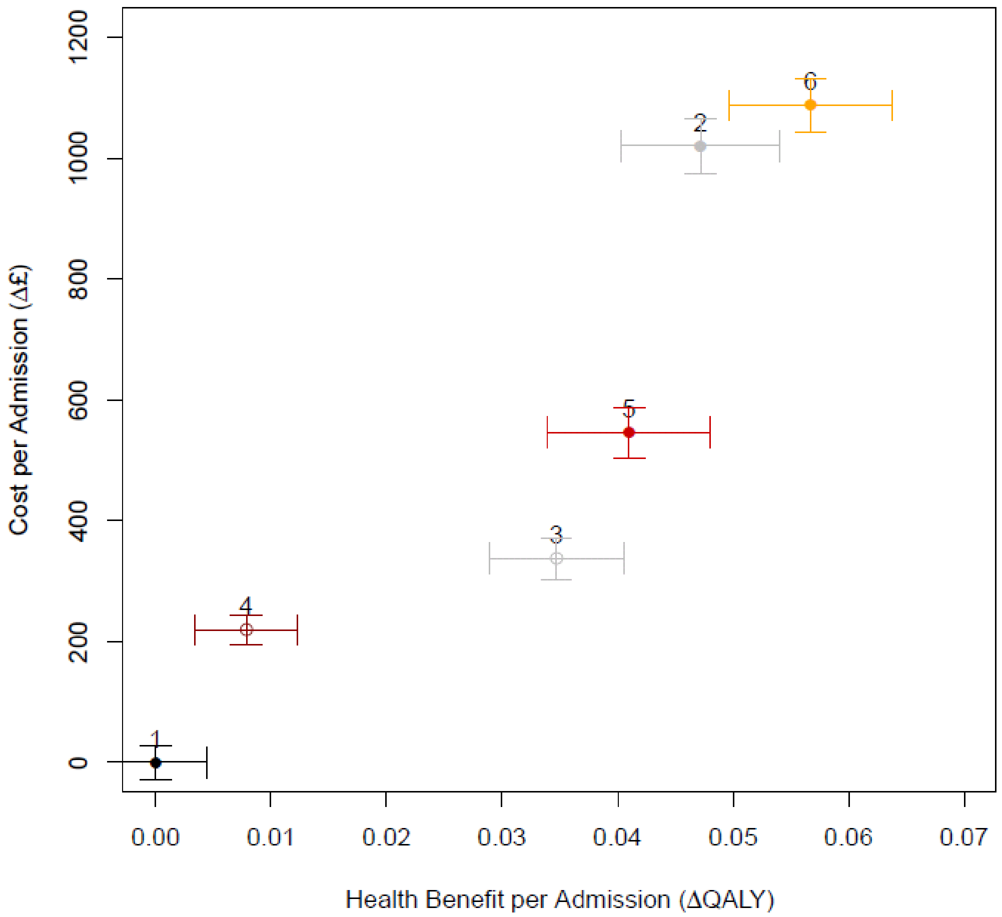

However, as well as the reduction in costs for these two strategies, gains in health benefits are also reduced in comparison to when the NOW audit definition of checklist positivity was used (Figure A4).

Despite the prevalence in the checklist positive population being greater when using the Pathfinder definition of checklist positivity (and thus the number of 'positives per screen' being greater), this is counteracted by the vast reduction (approximately 2/3) in the number classified as checklist positive under this definition (and therefore reduction in numbers of admissions screened). Taking these two effects into

account, the strategies involving checklist-activated screening will 'miss' more positive patients under the Pathfinder definition.

Putting these changes to incremental costs and incremental effects (or health benefits) together, as both costs and effects for checklist-activated screening strategies (strategies 4 and 5), the overall incremental cost-effectiveness evaluations of the cost-effectiveness frontier (Table A7) are very similar to those found using the audit definition of checklist positivity. Strategy 3, screening admissions to high risk specialties, remains the only strategy to be cost-effective at £9,731 /QALY gained.

Table A7. Evaluation of cost-effectiveness frontier of strategies.

Move between policies	Cost per admission	QALY per admission	Change in costs, ΔC (compared to previous policy)	Change in effects, ΔE (compared to previous policy)	Difference in Costs / Difference in Effects (ICER, cost/QALY)	Option evaluation
Stay at policy 1	£22,859	7.99248	-	-	-	-
Or move:						
to 3	£23,197	8.02719	£337.77	0.034711	£9,731	Cost effective
to 5	£23,405	8.033475	£208.70	0.006285	£33,206	Not cost effective
to 6	£23,946	8.049186	£541.02	0.015711	£34,437	Not cost effective

The CEAC demonstrates similar levels of uncertainty in the decision between all strategies whether the NOW audit or Pathfinder definition of checklist positivity is used (Figure A30 compared to Figure A6). The CEAF (Figure A31) shows strategy 3, screening of admissions to high risk specialties, remains the optimal option for all values of willingness to pay in the £20,000-£30,000/QALY range, albeit with a level of certainty in this decision of less than 30% (due to uncertainty in the effectiveness of the intervention).

Figure A30. Cost-effectiveness acceptability curves. Each line represents the proportion of simulations, for a particular strategy, that are cost-effective, as a function of willingness to pay for health benefits.

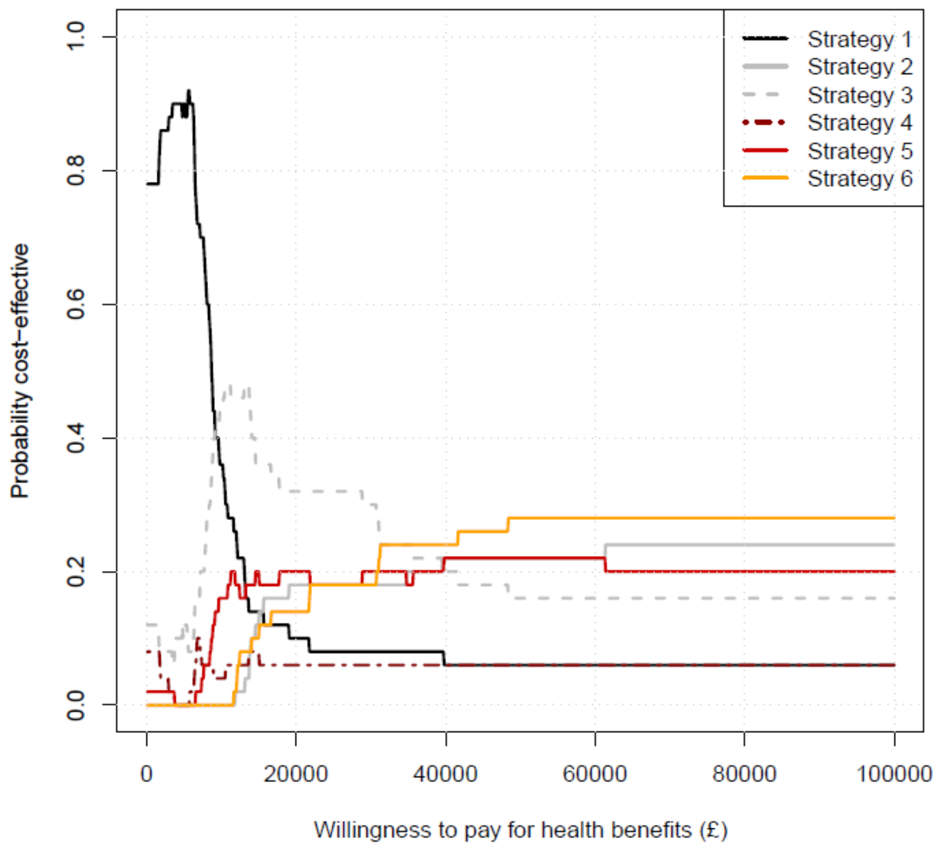

Figure A31. Cost-effectiveness acceptability frontier. Lines depict the strategies with the highest expected net monetary benefit, dependent on the willingness to pay for health benefits, while dotted vertical lines the willingness to pay values at which the decision changes.

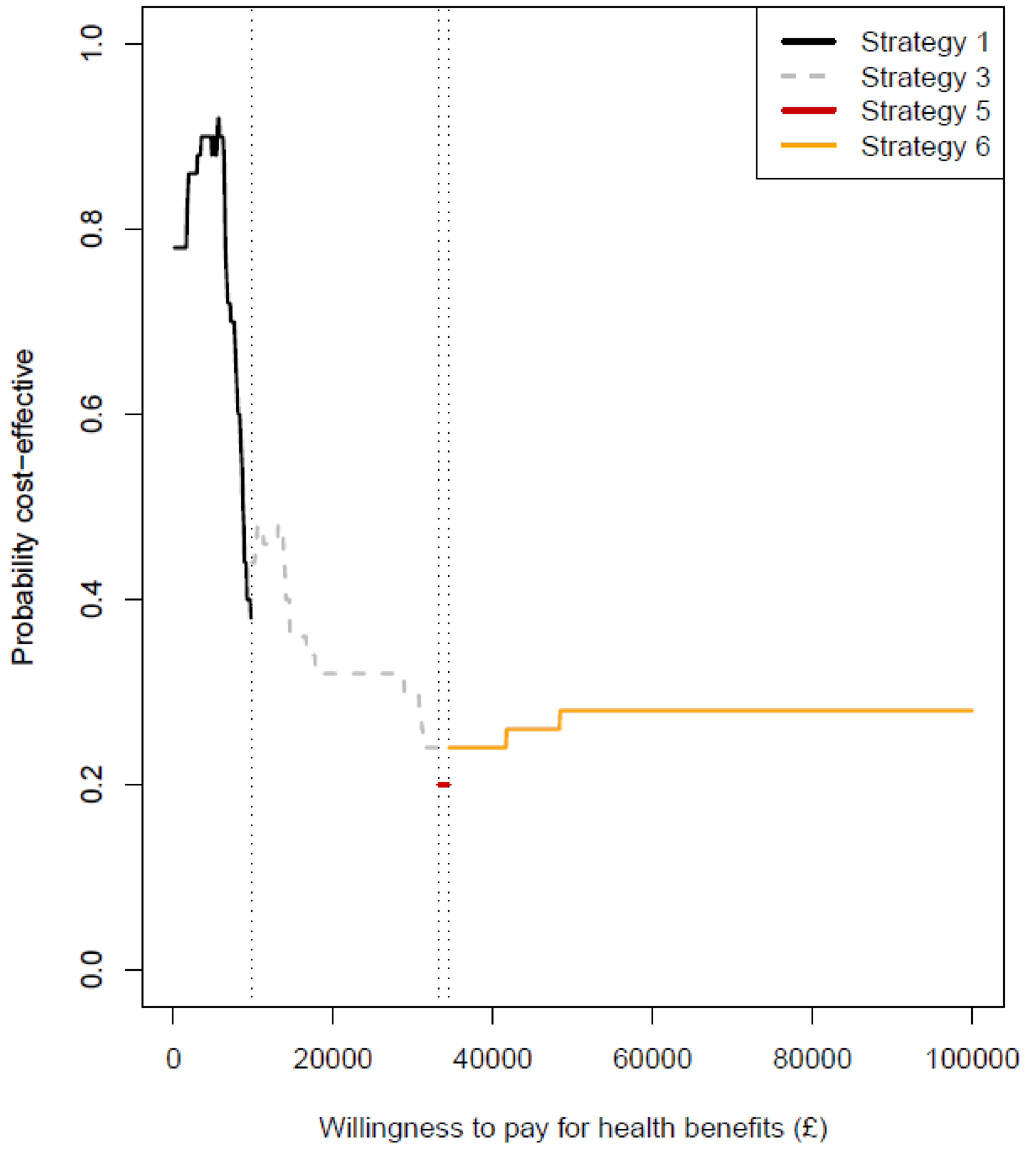

8 b. Teaching trusts

1. Baseline analyses

Effectiveness

In terms of the ability of the screening strategies to identify MRSA positive patients, the relative differences between strategies are approximately the same for Teaching Trusts (Figure B1), as were seen in Acute Trusts (Figure A1), with strategies 2 and 6 having the highest levels of isolation (both appropriate and inappropriate usage) and fewest unisolated MRSA positive bed days. However, due to a combination of a slightly reduced overall prevalence on admission in Teaching Trusts and lower transmission parameters leading to fewer clinical cases, absolute numbers of isolated bed days (per 100 bed days) are slightly lower in Teaching Trusts.

Due to the method by which transmission parameters have been estimated for each Trust type (see Model Parameters section), there is less onward transmission in this setting, as shown in Figure B2, meaning that there are fewer unisolated MRSA positive bed days in Teaching Trusts compared to Acute Trusts (Figure B1 compared to Figure A1). This is because each of the screening strategies is implemented on the admission population only, and therefore unable to influence the degree of onward transmission in the hospital caused by MRSA positive patients who are missed by screening. If these 'missed patients' go on to cause less onward transmission, as is the case here, it follows that there will be fewer unisolated MRSA positive days.

The reduced levels of onward transmission, infections events, and thus death events, are demonstrated in Figure B2 (as compared to A2). However, again, while absolute numbers may vary between Trust types, the ordering of the strategies remains the same.

Figure B1. Primary isolation usage under each screening strategy, showing appropriate isolation (isolation of patients who are MRSA positive), inappropriate isolation (isolation of MRSA negative patients) and unisolated bed days of MRSA positive patients.

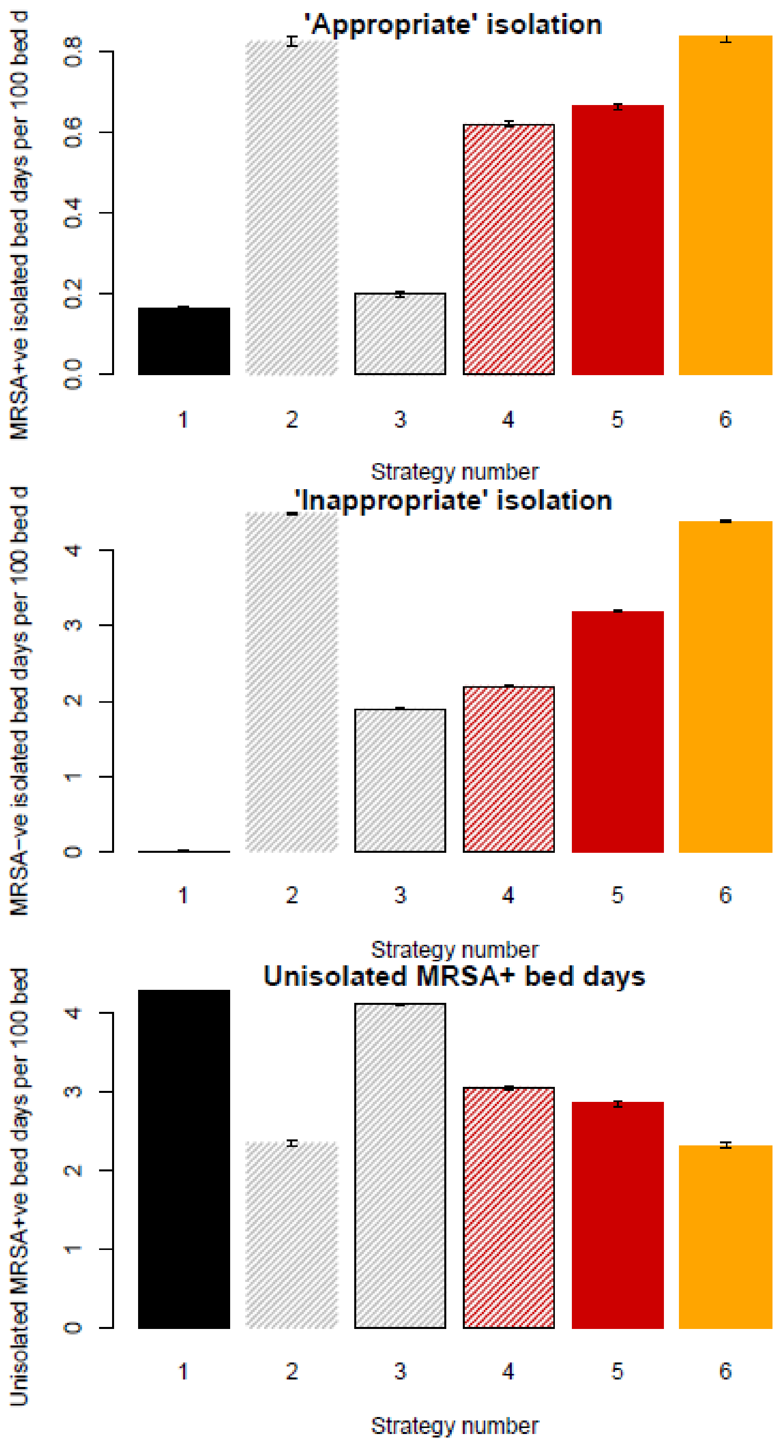

Figure B2. Patient outcomes under each screening strategy, showing new acquisitions of MRSA by hospital patients, total MRSA infections in the hospital, and total deaths (all per 100 admissions).

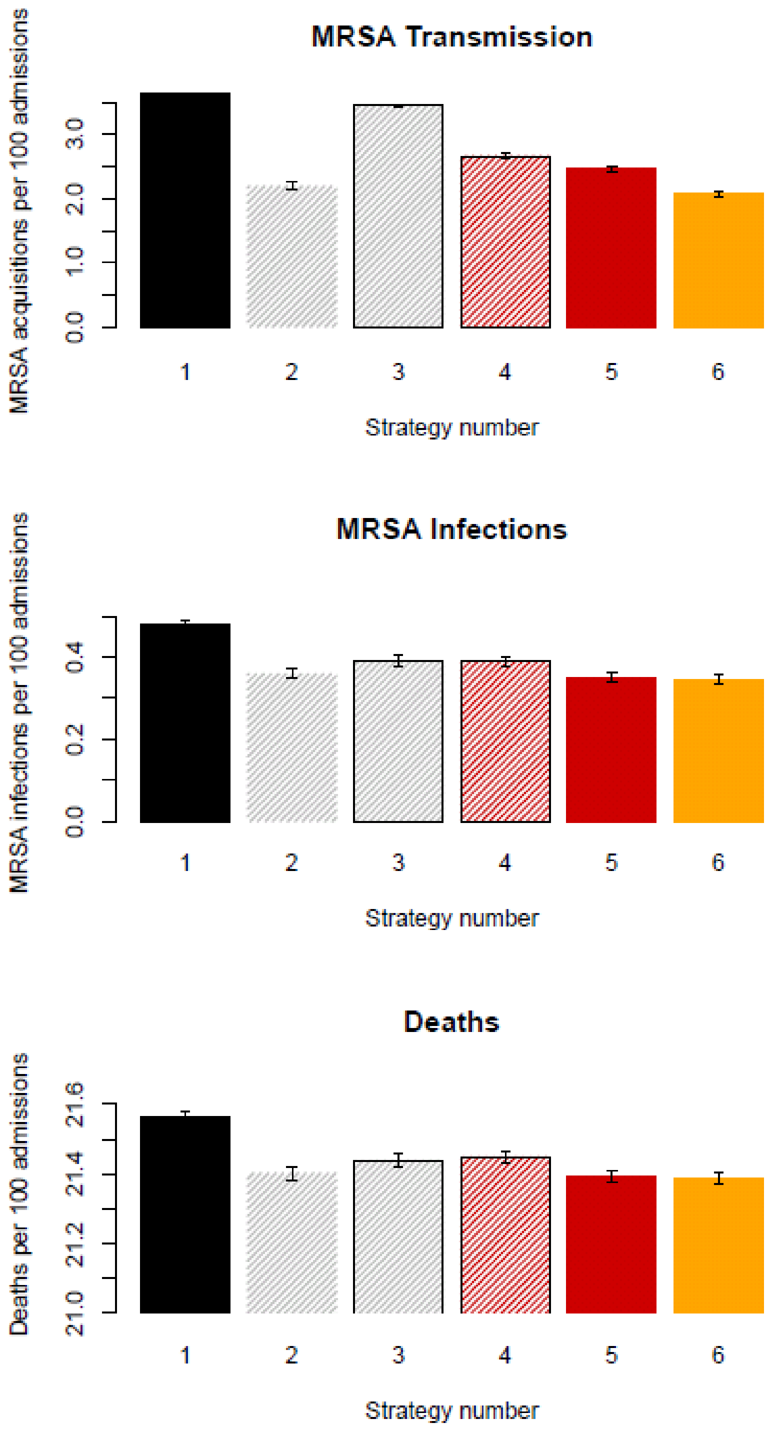

Costs

Compared to an Acute setting, in a Teaching Trust setting each of the strategies are associated with greater costs per admission (Figure B3 for Teaching, compared to Figure A3 for Acute). This is due to the larger size of the Teaching Trusts (a median of 1113 beds, compared to 553 for Acute) leading to greater numbers of bed days over the simulation period. Overall bed day costs for the 5 year simulation period in Acute Trusts are 50% less than those for Teaching Trusts. Therefore the numerator in the cost per admission equation is halved for Acute Trusts. Whereas, the differences in the number of admissions over the simulation period (the denominator) are not so pronounced, with Acute Trusts having only 22% fewer admissions compared to Teaching Trusts). Together, these two effects lead to Teaching Trusts having greater costs per admission.

The lower numbers of admissions to Teaching Trusts than might be expected given their size, are due to the distribution of high risk and low risk specialty beds – Teaching Trusts having approximately 44% more high risk specialty beds than Acute Trusts. Having more high risk specialty patients will result in altered movement parameters within the hospital and between the hospital and community (for movement parameters see Appendix 4).

It is worth noting that while the absolute costs of the screening itself (as a component of the total costs) will be greater in Teaching Trusts due to larger number of patients needing to be screened, the costs per admission are identical to those in Acute Trusts (Figure B3, ‘Combined screening costs’ panel; compared to Figure A3).

Figure B3. Cost per admission for each strategy, presented as total costs and broken down into component parts (note different scales of sub graphs).

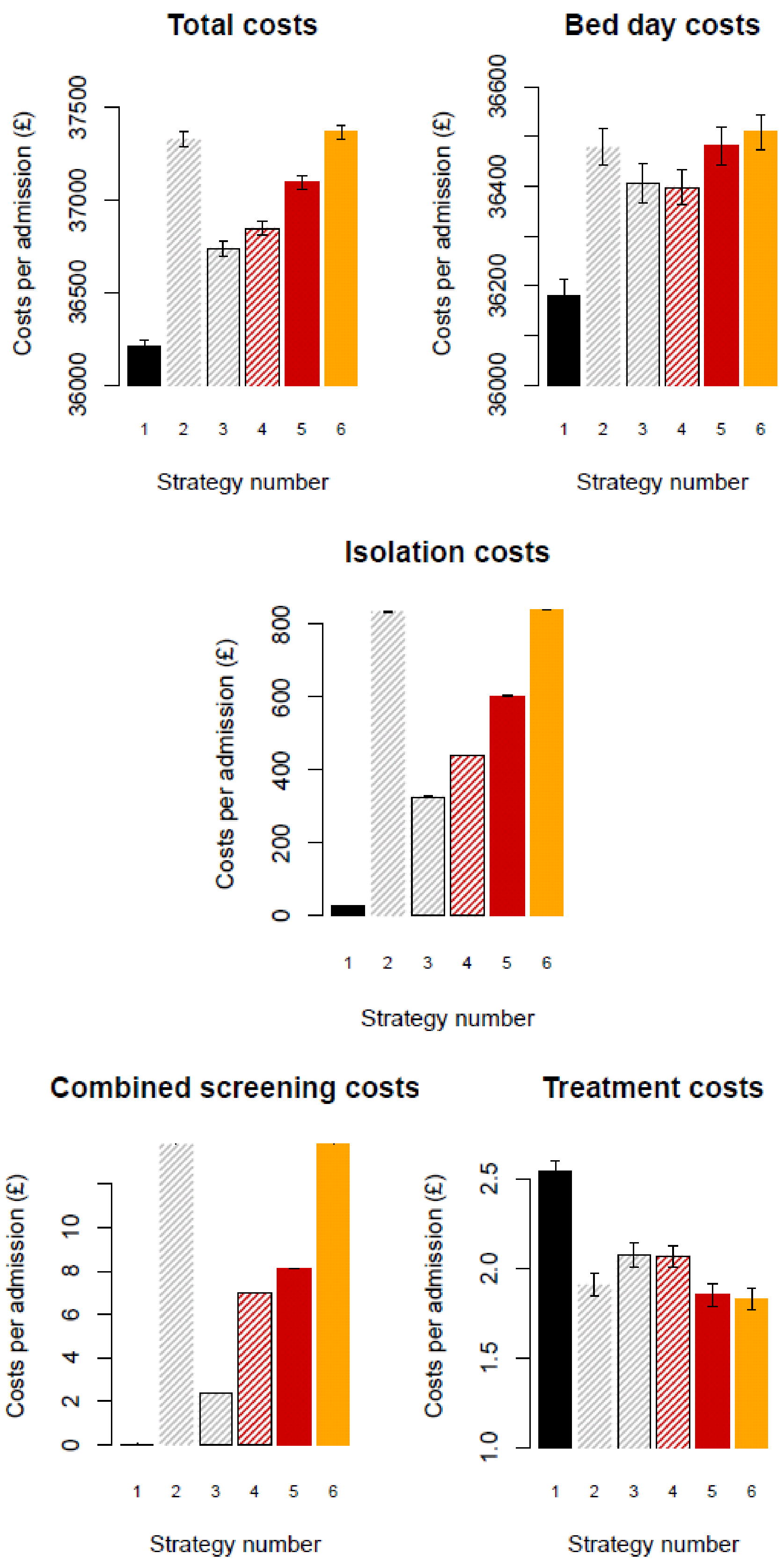

Cost-effectiveness

From the incremental cost-effectiveness plot (Figure B4), it can be seen that each of the strategies generates fewer health benefits (compared to the baseline 'no screening' strategy, strategy 1) than are achieved in Acute settings (Figure A4). This is due to the lower transmission seen in a Teaching Trust setting (Figure B2). However, despite the fact that fewer QALYs are gained, strategy 3 (screening patients admitted to high risk specialties) - the most cost-effective option on the cost-effectiveness frontier in Acute Trusts (Table A2) – is only approximately £1,000 more costly than the NHS willingness to pay threshold of £30,000/QALY in this setting, at £31,077/QALY (Table B1).

Figure B4. Incremental cost-effectiveness plot comparing each of the screening strategies. Numbers indicate strategy numbers as outlined above. Error bars represent random error brought about by stochasticity in the model and parameter uncertainty, and correspond to plus or minus one standard error.

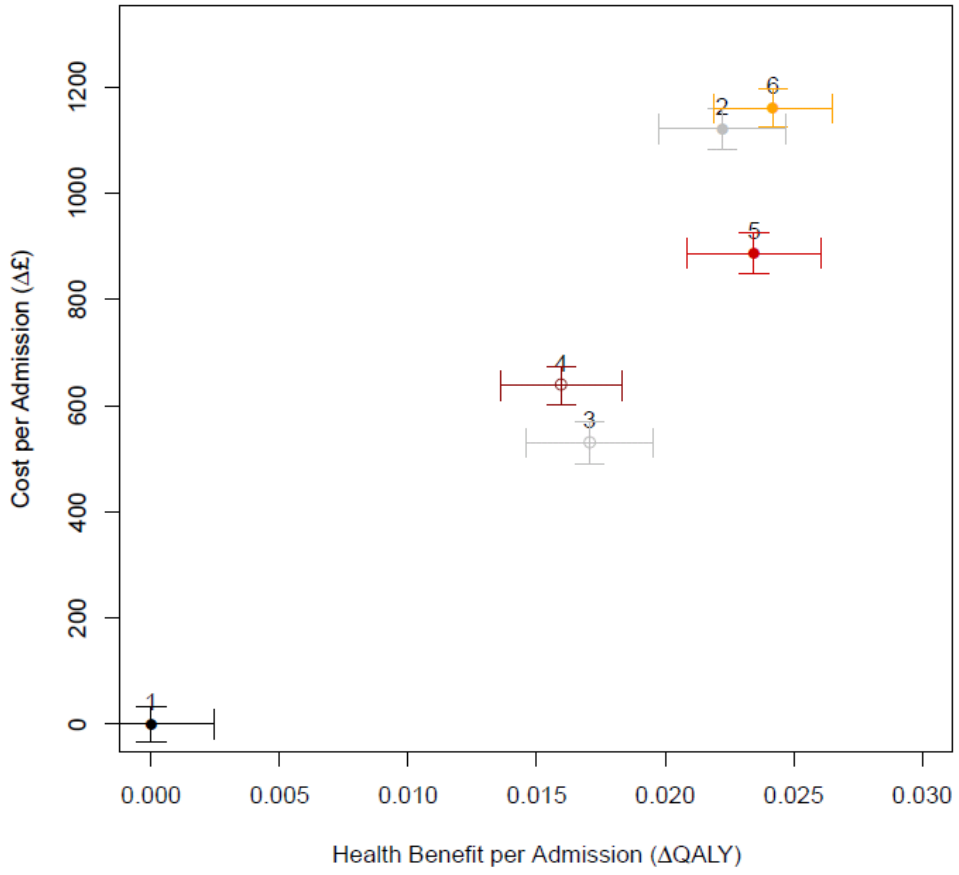

Table B1. Evaluation of cost-effectiveness frontier of strategies.

Move between policies	Cost per admission	QALY per admission	Change in costs, ΔC (compared to previous policy)	Change in effects, ΔE (compared to previous policy)	Difference in Costs / Difference in Effects (ICER, cost/QALY)	Option evaluation
Stay at policy 1	£36,207	8.225403	-	-	-	-
Or move:						
to 3	£36,737	8.242465	£530.26	0.017063	£31,077	Not cost effective
to 5	£37,094	8.248859	£356.39	0.006394	£55,742	Not cost effective
to 6	£37,366	8.249589	£273.18	0.0073	£374,070	Not cost effective

Consideration of uncertainty

It must be noted that the degree of uncertainty in this decision is large (depicted graphically in Figure B5). Considering this uncertainty the CEAC (Figure B6) demonstrates that within the NHS willingness to pay range of £20,000 - £30,000/QALY, no one screening strategy has a greater than 30% chance of cost-effectiveness. Indeed, the CEAF (Figure B7) shows that considering all uncertainty, the optimal option is the isolation and decolonisation of clinical cases only. The decision only changing at willingness to pay values slightly over £30,000/QALY. However, under only slightly different assumptions for transmission parameters it is likely that the cost-effectiveness of strategies would shift, which (given the proximity of the decision to the threshold), would be likely to change the decision.

Figure B5. Simulation results comparing each strategy on a cost-effectiveness plane for a Teaching Trust setting. Each dot represents the mean of 1000 simulation runs for each parameter set. The results of 50 parameter sets for each strategy are plotted, where each parameter set is obtained by taking the mean value for all parameters apart from the effectiveness of the intervention, which is sampled from its probability distribution.

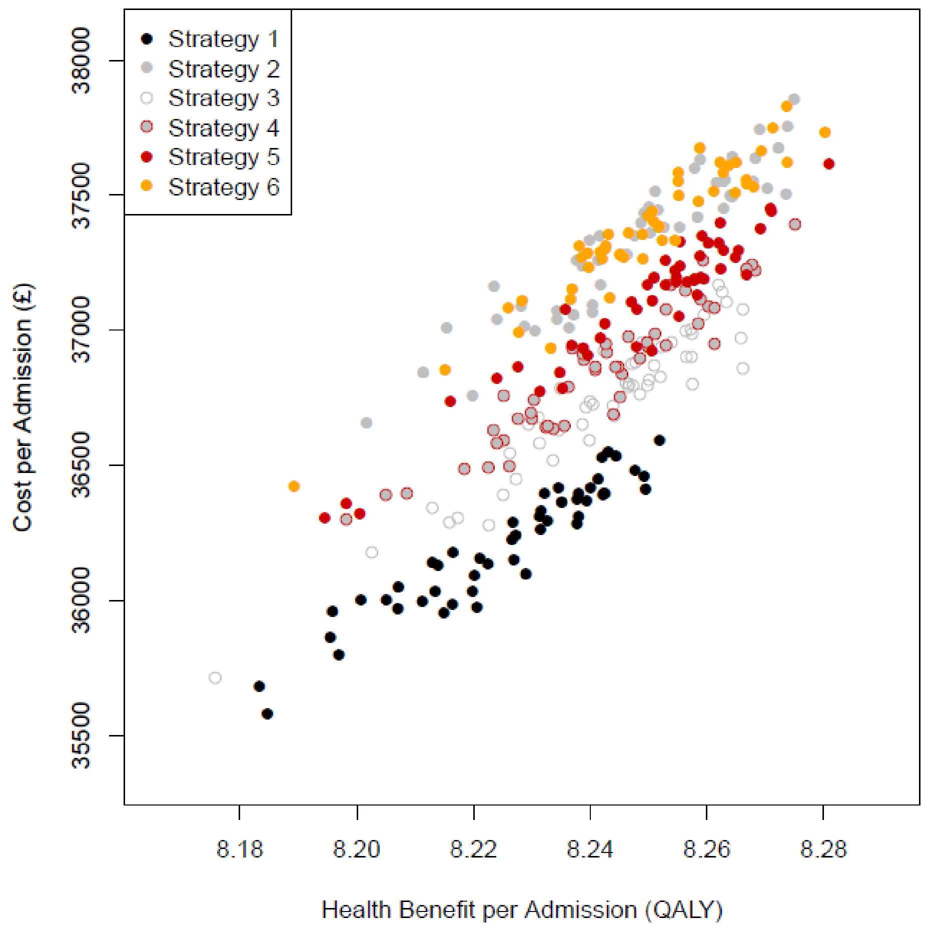

Figure B6. Cost-effectiveness acceptability curves. Each line represents the proportion of simulations, for a particular strategy, that are cost-effective, as a function of willingness to pay for health benefits.

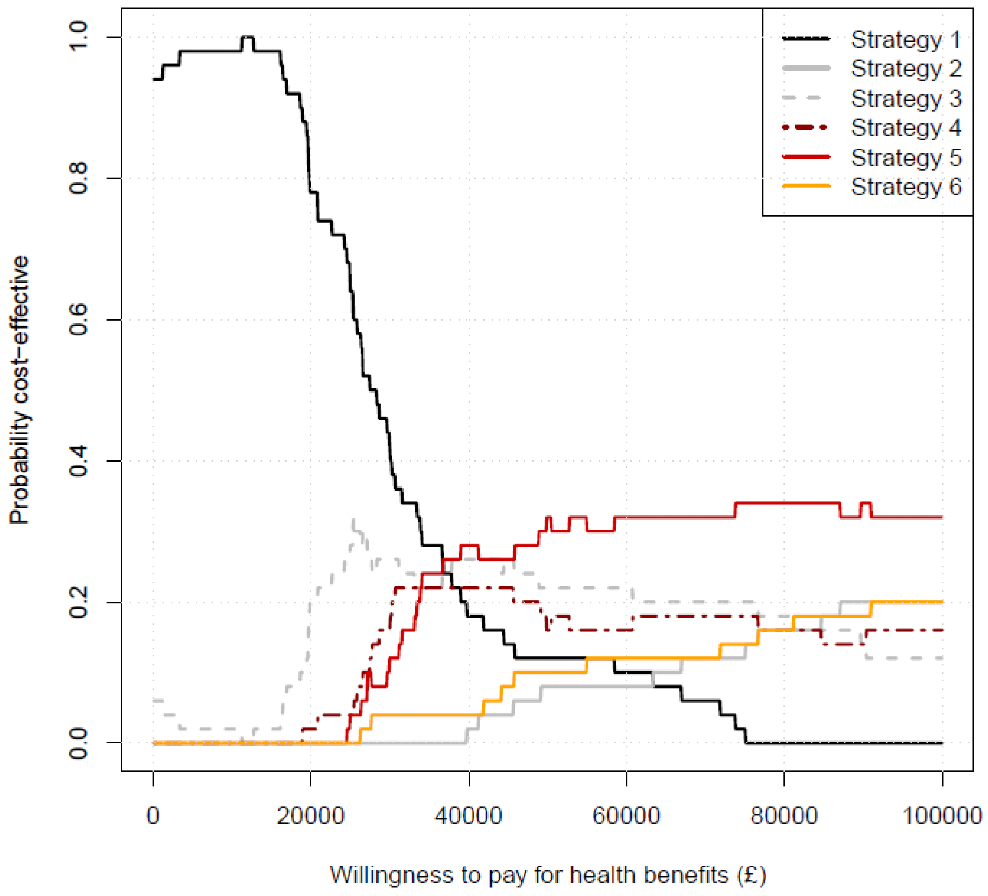

Figure B7. Cost-effectiveness acceptability frontier. Lines depict the strategies with the highest expected net monetary benefit, dependent on the willingness to pay for health benefits, while dotted vertical lines the willingness to pay values at which the decision changes.

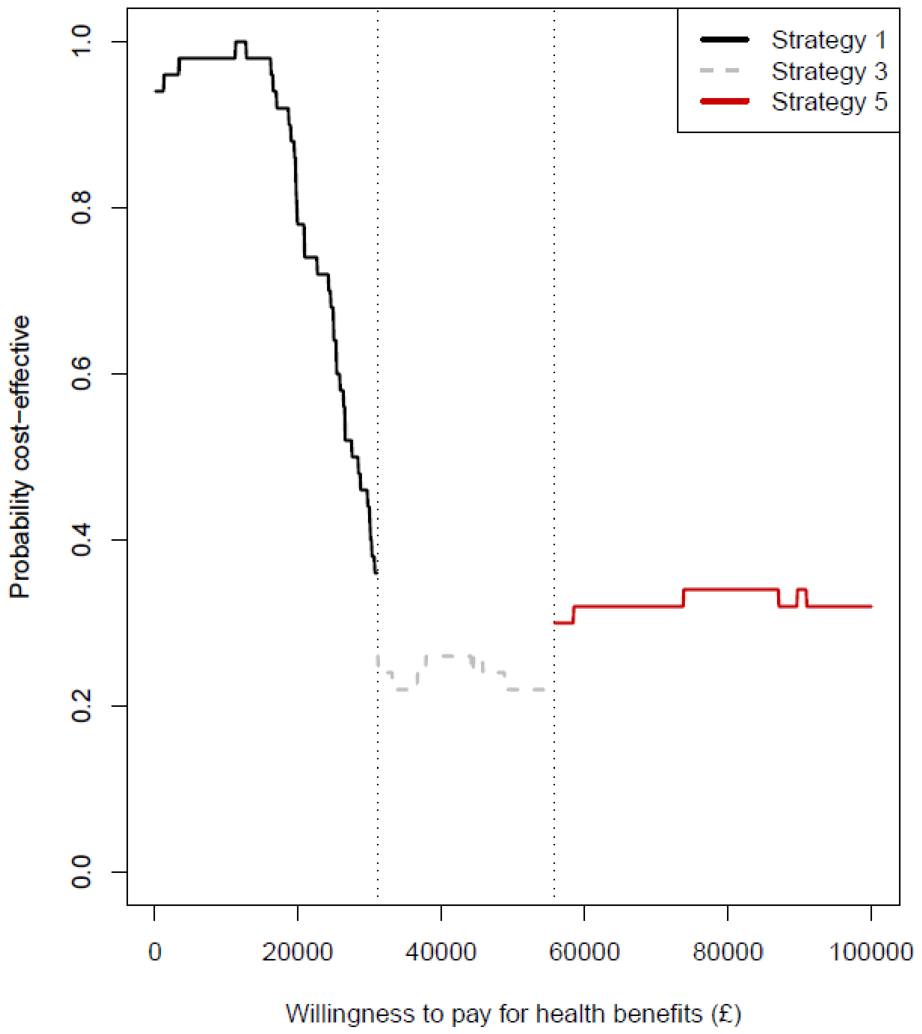

2. Scenario analyses

The following results explore each of the strategies in a Teaching Trust setting in a series of scenario analyses, namely: i) a high prevalence setting (where prevalence is twice that found in the audit for Teaching Trusts: 2.6% compared to 1.3%); and ii) a low prevalence setting (where the prevalence is half that found from the audit for Teaching Trusts 0.65% compared to 1.3%).

High prevalence setting

As would be expected in a high prevalence setting, both appropriate and inappropriate isolation increase, compared to in a baseline prevalence (Figure B8 compared to Figure B1), due to a greater absolute burden of colonisation, meaning more MRSA positive patients will be both 'picked up' and missed through any screening approach. As in an Acute setting, despite more patients being screened and isolated the degree of inappropriate isolation remains the same as in a baseline prevalence setting, potentially due to the greater positive predictive value of the screening test in high prevalence settings.

The higher prevalence leads to greater numbers of transmission events, MRSA infections and therefore death events per 100 admissions (Figure B9) compared to the baseline prevalence setting (Figure B2). Total costs are reduced slightly (Figure B10 compared to Figure B3), due to screening strategies being more able to impact transmission and therefore reduce infections leading to cost savings. Indeed, each of the screening strategies (compared to the baseline strategy) are able to provide greater gains in health benefits in a high prevalence setting (Figure B11), compared to gains seen in the baseline prevalence setting (Figure B4). These greater gains in health benefits have the consequence of making the ICER for the optimal strategy, screening patients admitted to high risk specialties (strategy 3), cost-effective at £27,715/QALY.

While the CEAC (Figure B12) still exhibits considerable uncertainty, the CEAF (Figure B13) shows strategy 3 to be optimal throughout the NHS willingness to pay range, albeit at a probability of cost –effectiveness of around only 40%.

Figure B8. Primary isolation usage under each screening strategy, showing appropriate isolation (isolation of patients who are MRSA positive), inappropriate isolation (isolation of MRSA negative patients) and unisolated bed days of MRSA positive patients.

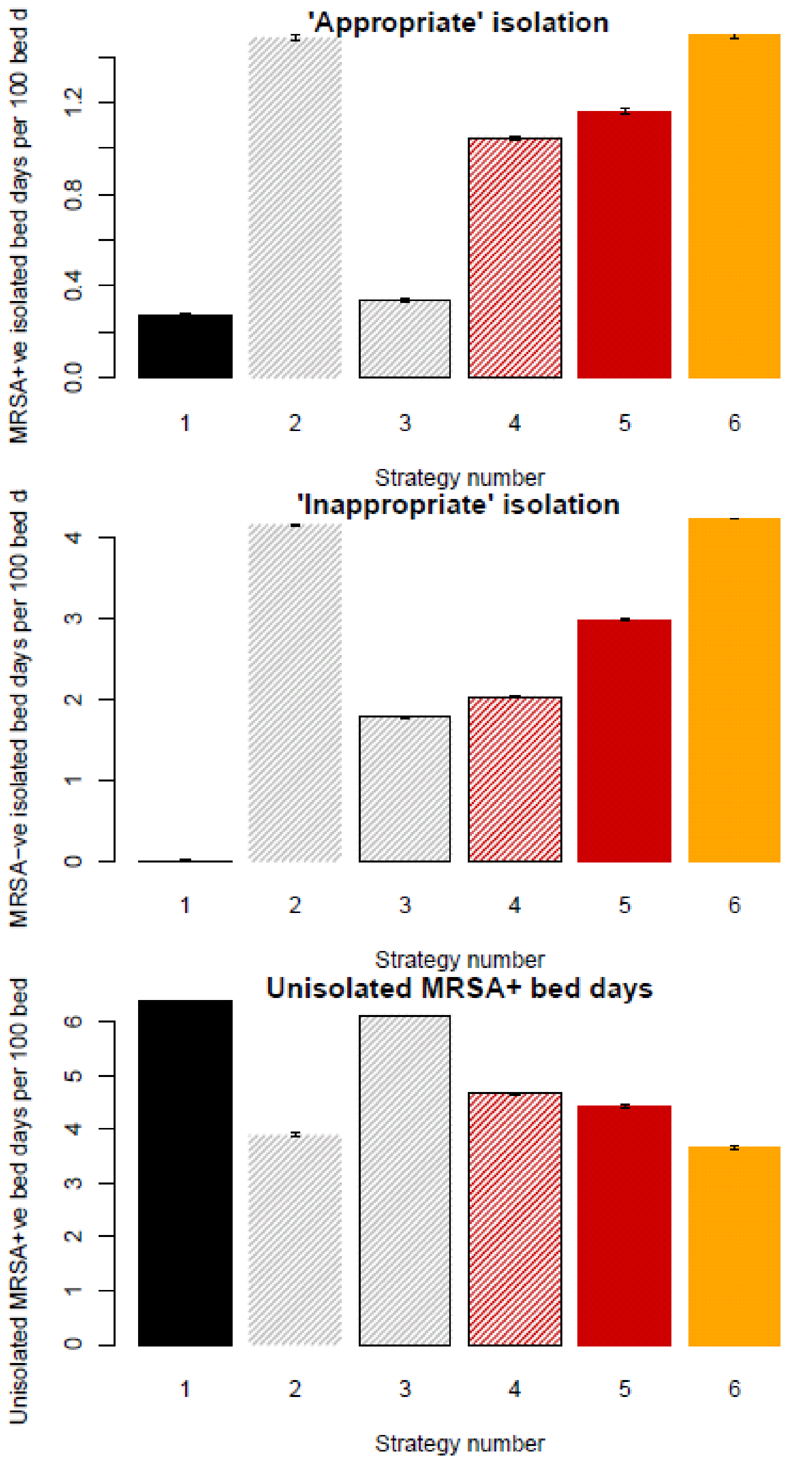

Figure B9. Patient outcomes under each screening strategy, showing new acquisitions of MRSA by hospital patients, total MRSA infections in the hospital, and total deaths (all per 100 admissions).

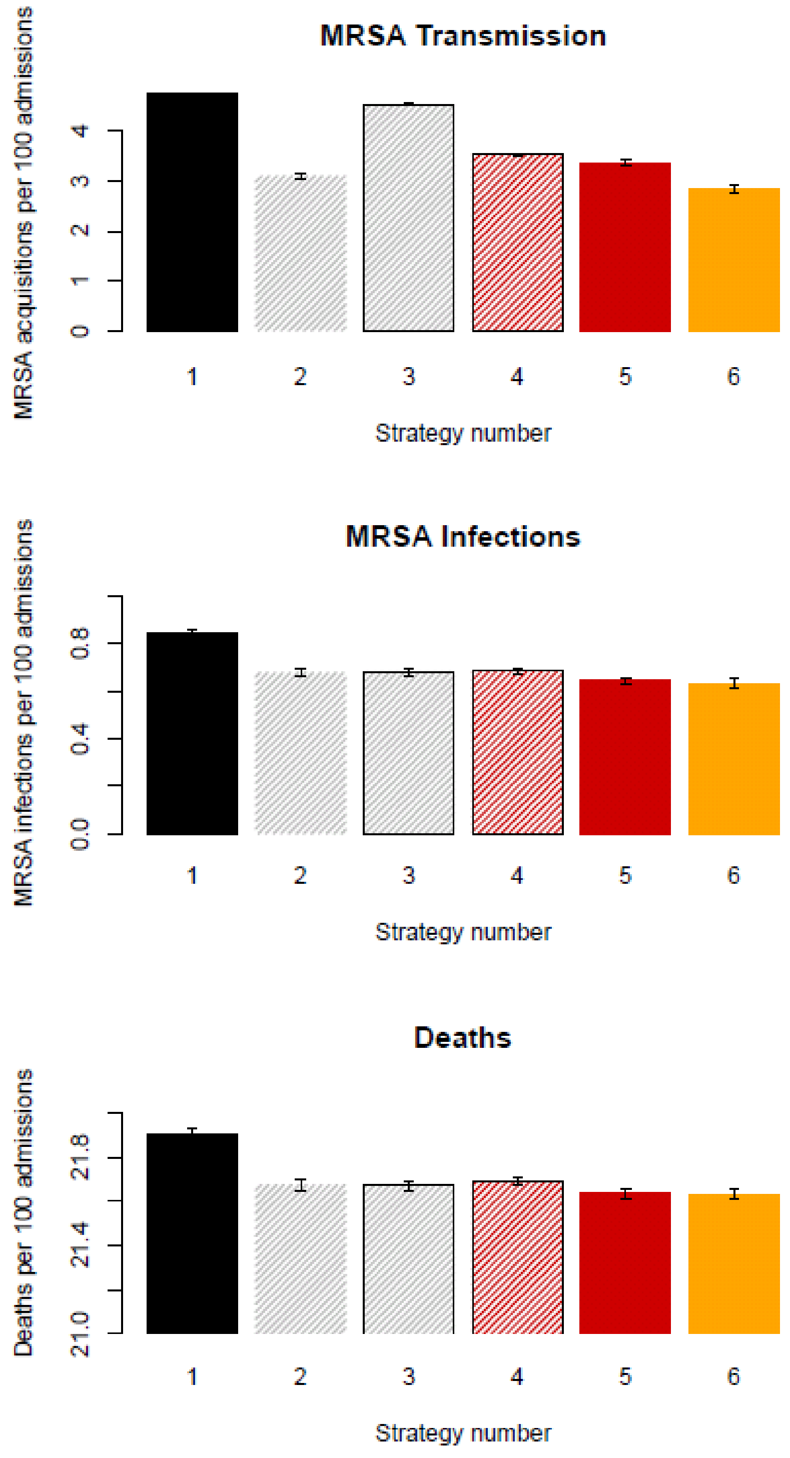

Figure B10. Cost per admission for each strategy, presented as total costs and broken down into component parts (note different scales of sub graphs).

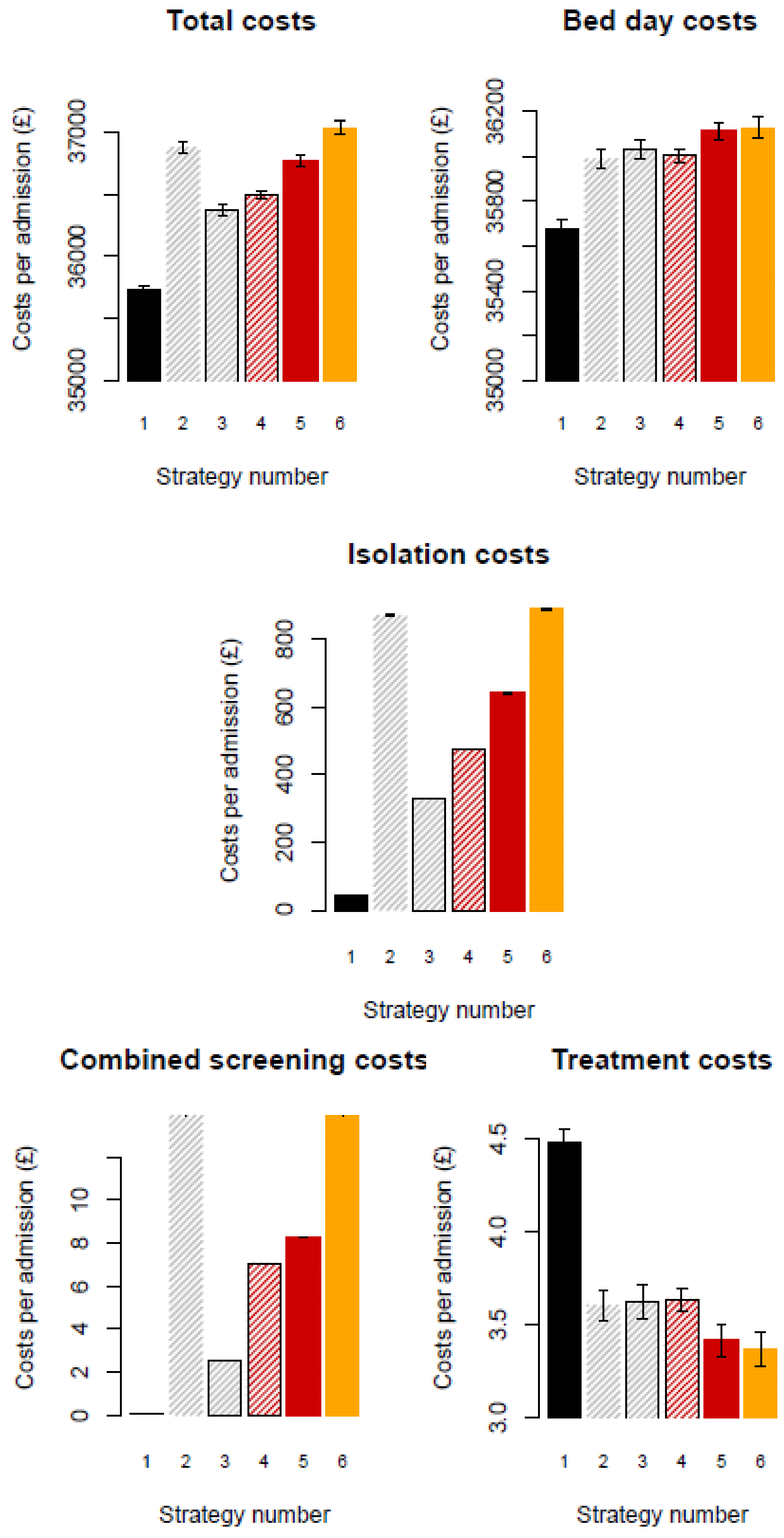

Figure B11. Incremental cost-effectiveness plot comparing each of the screening strategies. Numbers indicate strategy numbers as outlined above. Error bars represent random error brought about by stochasticity in the model and parameter uncertainty, and correspond to plus or minus one standard error.

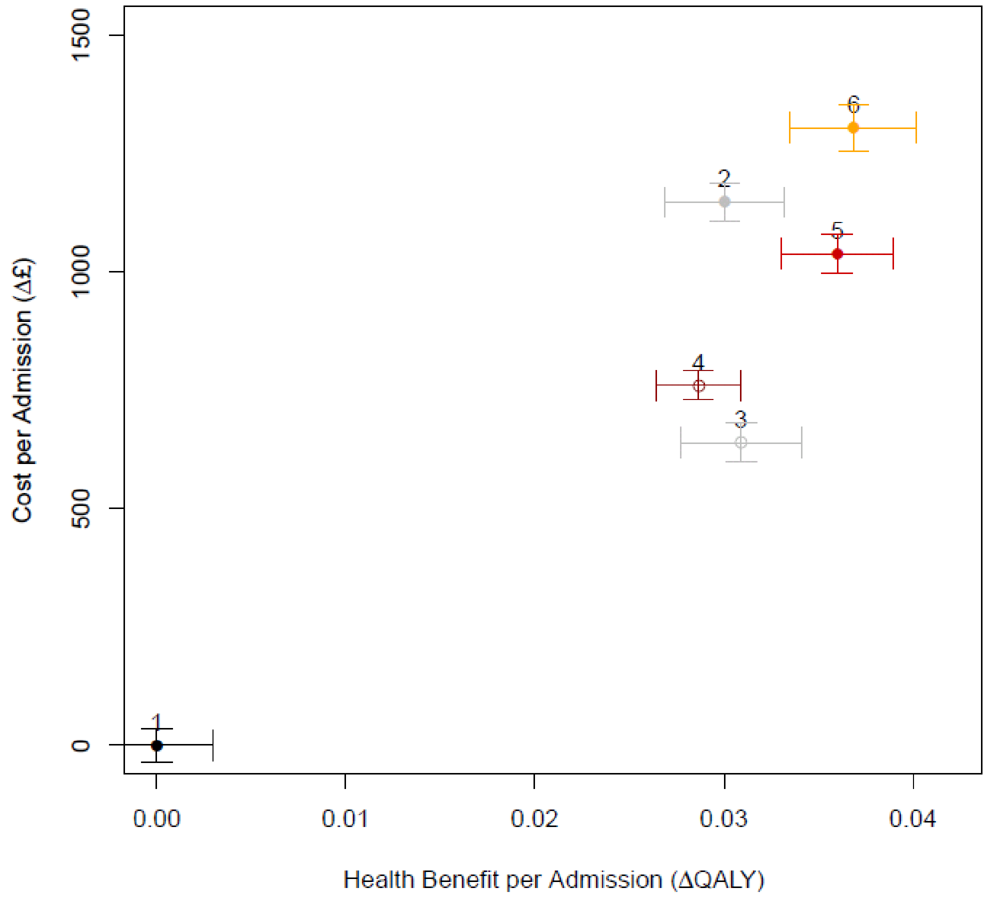

Table B2. Evaluation of cost-effectiveness frontier of strategies.

Move between policies	Cost per admission	QALY per admission	Change in costs, ΔC (compared to previous policy)	Change in effects, ΔE (compared to previous policy)	Difference in Costs / Difference in Effects (ICER, cost/QALY)	Option evaluation
Stay at policy 1	£35,728	8.177714	-	-	-	-
Or move:						
to 3	£36,368	8.208617	£640.15	0.030903	£20,715	Cost effective
to 5	£36,766	8.213712	£398.69	0.005095	£78,258	Not cost effective
to 6	£37,032	8.213712	£266.04	0.000861	£308,839	Not cost effective

Figure B12. Cost-effectiveness acceptability curves. Each line represents the proportion of simulations, for a particular strategy, that are cost-effective, as a function of willingness to pay for health benefits.

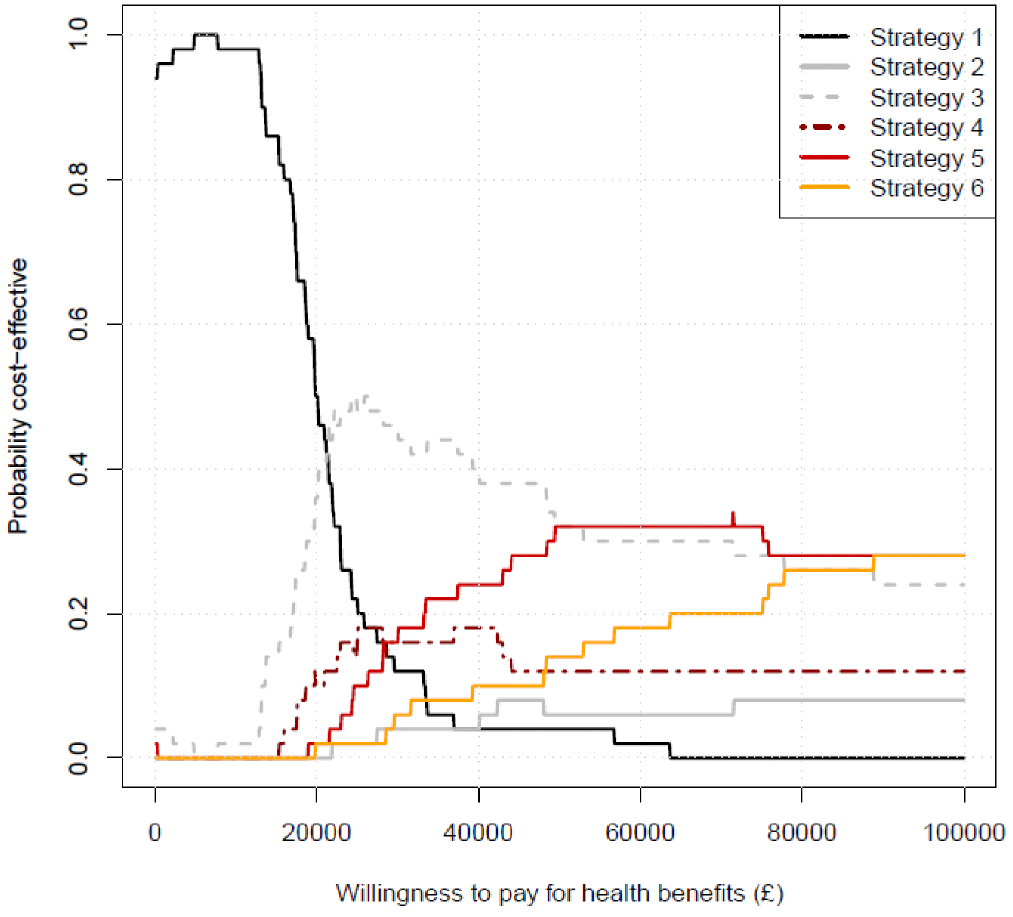

Figure B13. Cost-effectiveness acceptability frontier. Lines depict the strategies with the highest expected net monetary benefit, dependent on the willingness to pay for health benefits, while dotted vertical lines the willingness to pay values at which the decision changes.

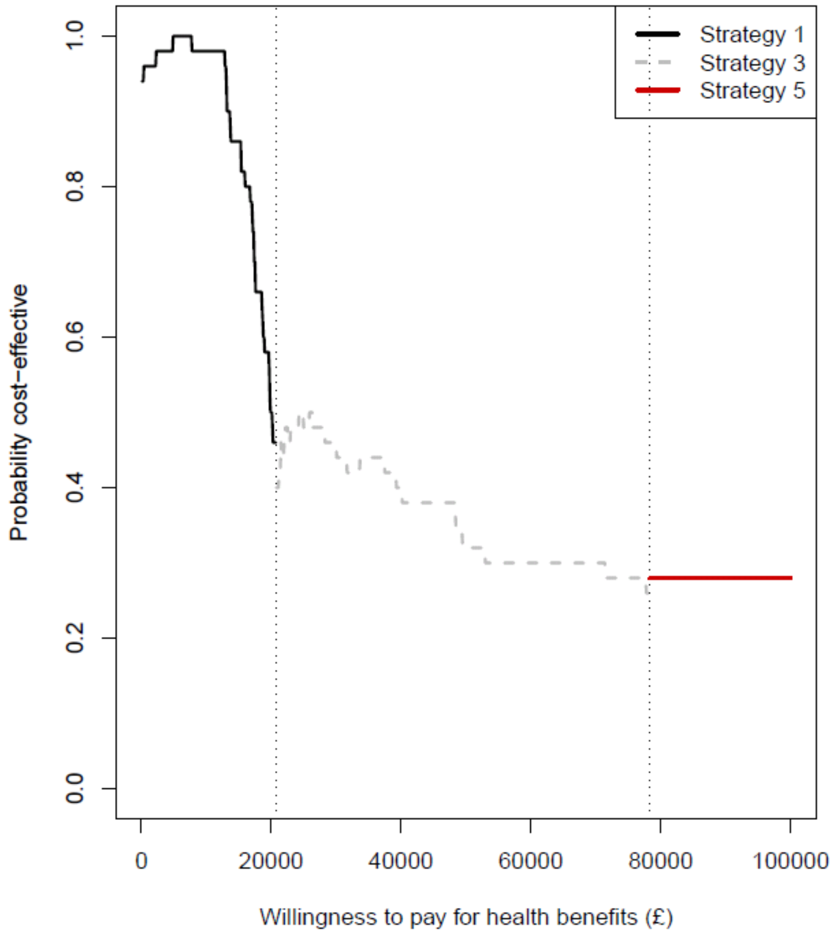

Low prevalence setting

In a low prevalence of setting the amount of intervention usage is reduced (Figure B14), as are transmission, infection and death events (Figure B15), due to the lower infectious assault on the hospital. This translates to very slightly higher total costs as, in a setting of reduced infections, the same level of effort will yield less of an impact (Figure B16). From the incremental cost-effectiveness plot (Figure B17), it can be seen that incremental gains in health benefits are lower (compared to the baseline strategy) in a lower prevalence setting, compared to an average prevalence setting (Figure B4). The incremental effectiveness, in terms of QALY gain, of strategy 2 in particular, screening all patients on admission, is reduced in a low prevalence setting.

Overall, with a lower prevalence on admission none of the strategies are cost-effective. It is much harder for the screening strategies to be cost-effective, with ICERs (Table B3) for all strategies on the cost-effectiveness frontier falling above the cost-effectiveness threshold of £30,000/QALY.

In this case, the CEAC (Figure B18) shows that up to a willingness to pay of approximately £40,000/QALY none of the screening strategies are cost-effective, with strategy 1, isolating only clinical cases, being optimal (as seen from the CEAF, Figure B19).

Figure B14. Primary isolation usage under each screening strategy, showing appropriate isolation (isolation of patients who are MRSA positive), inappropriate isolation (isolation of MRSA negative patients) and unisolated bed days of MRSA positive patients.

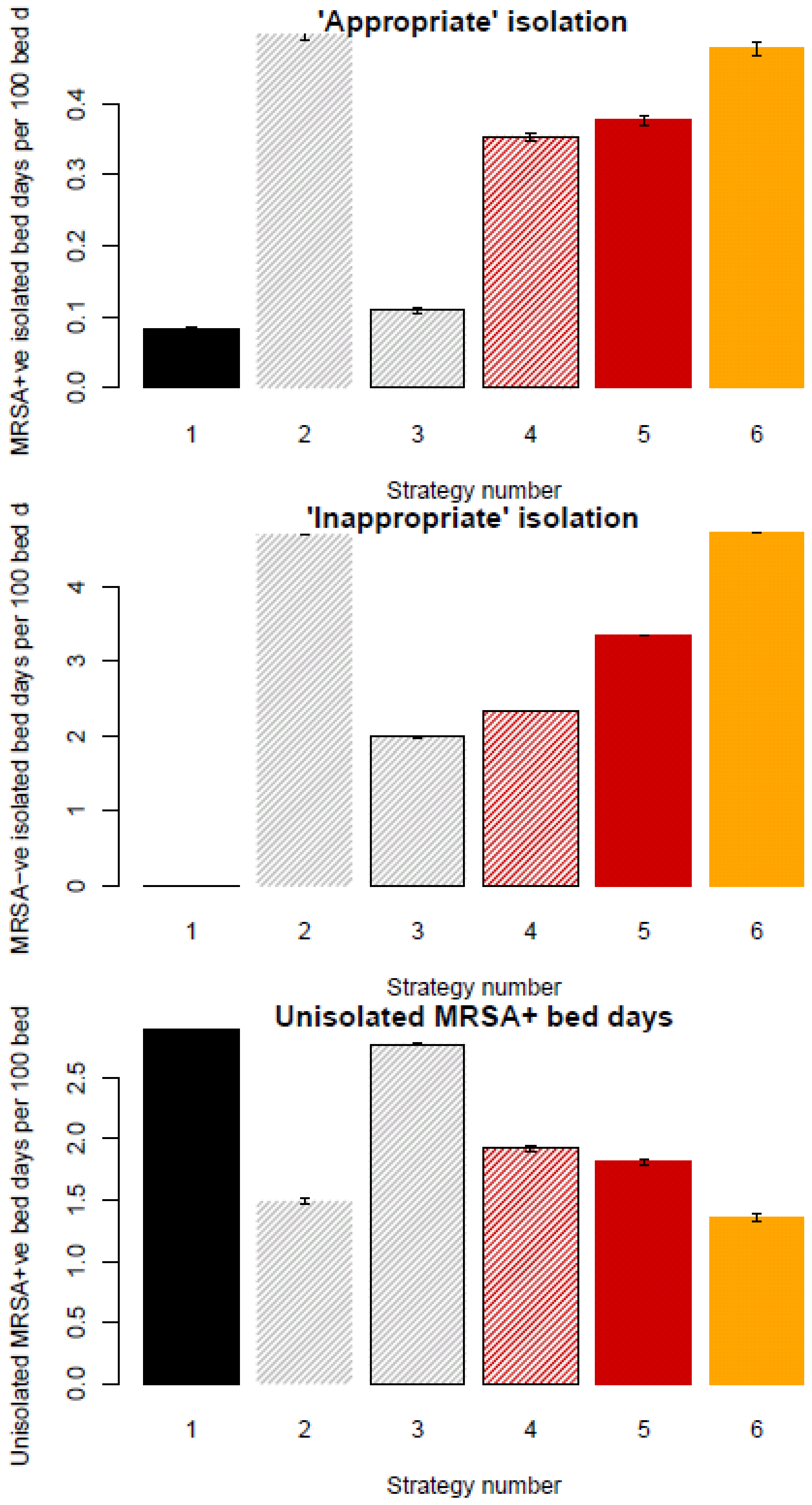

Figure B15. Patient outcomes under each screening strategy, showing new acquisitions of MRSA by hospital patients, total MRSA infections in the hospital, and total deaths (all per 100 admissions).

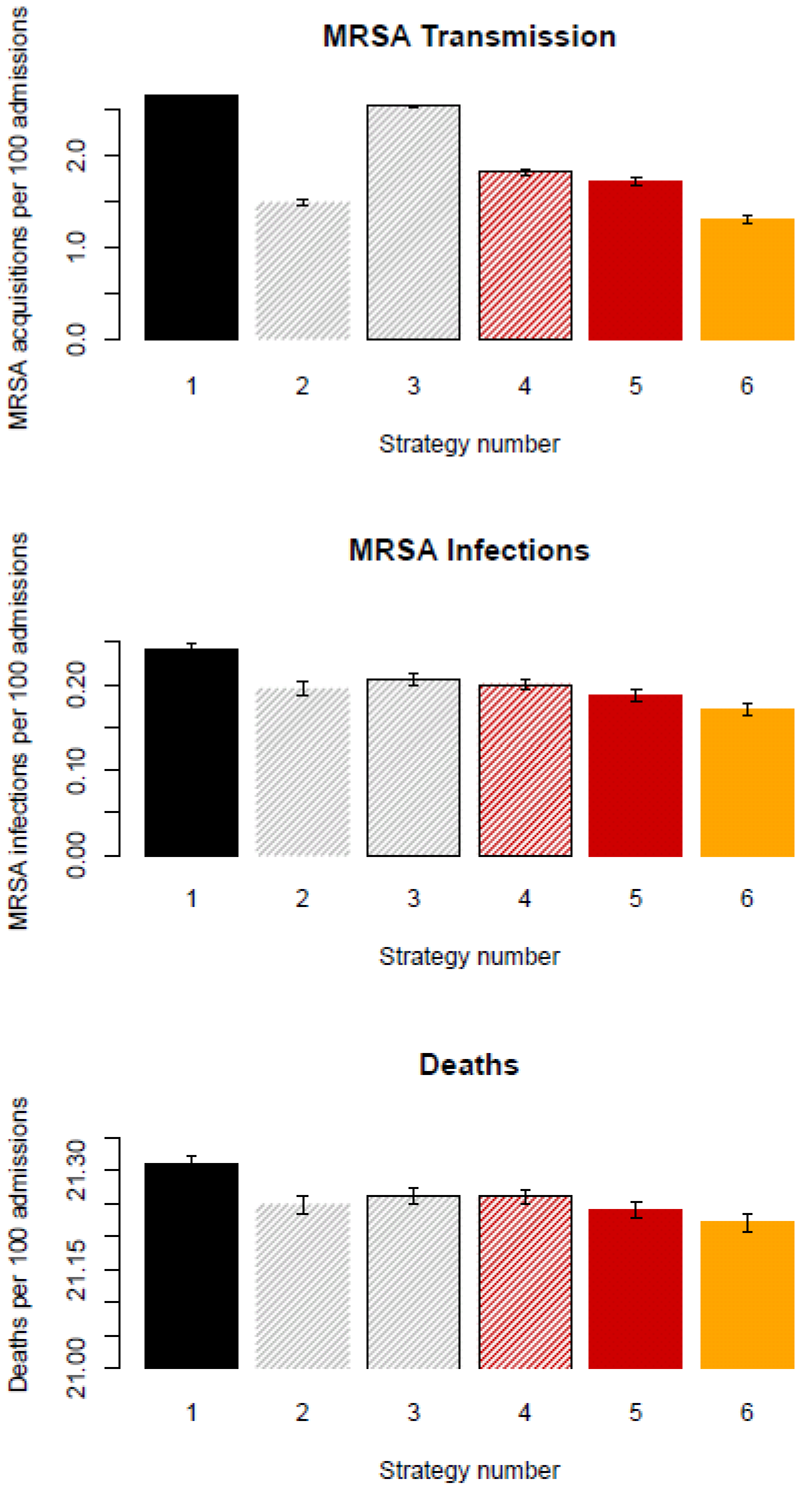

Figure B16. Cost per admission for each strategy, presented as total costs and broken down into component parts (note different scales of sub graphs).

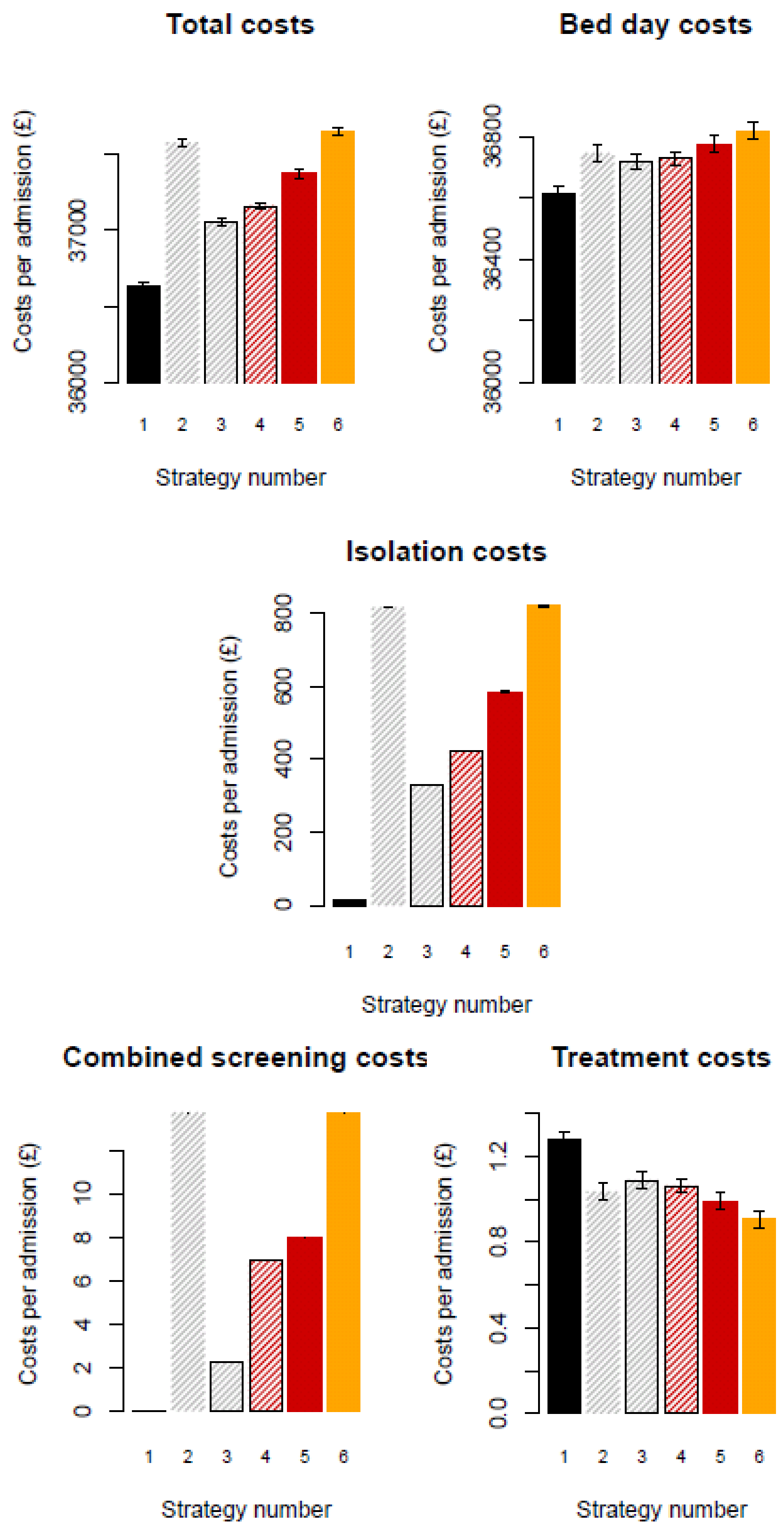

Figure B17. Incremental cost-effectiveness plot comparing each of the screening strategies. Numbers indicate strategy numbers as outlined above. Error bars represent random error brought about by stochasticity in the model and parameter uncertainty, and correspond to plus or minus one standard error.

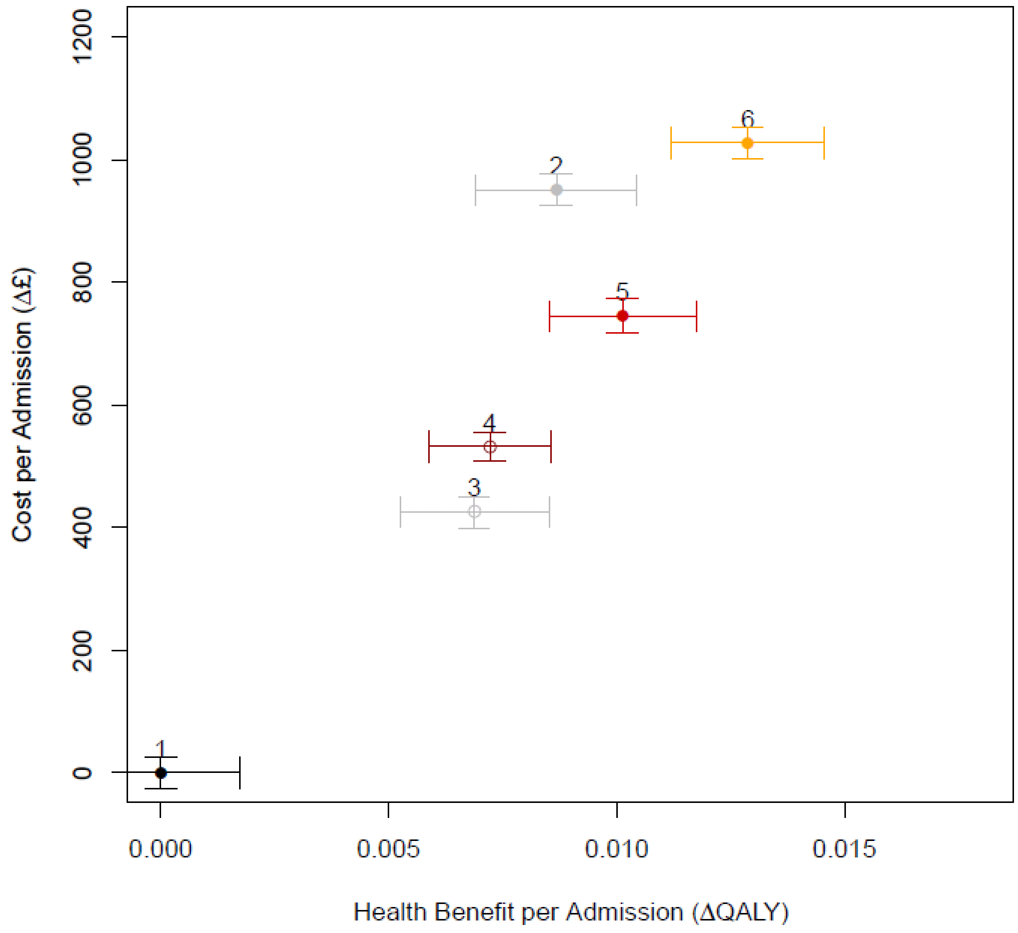

Table B3. Evaluation of cost-effectiveness frontier of strategies.

Move between policies	Cost per admission	QALY per admission	Change in costs, ΔC (compared to previous policy)	Change in effects, ΔE (compared to previous policy)	Difference in Costs / Difference in Effects (ICER, cost/QALY)	Option evaluation
Stay at policy 1	£36,627	8.261082	-	-	-	-
Or move:						
to 3	£37,052	8.267965	£424.79	0.006884	£61,710	Not cost effective
to 5	£37,371	8.271209	£319.92	0.003243	£98,634	Not cost effective
to 6	£37,655	8.273961	£282.89	0.002752	£102,797	Not cost effective

Figure B18. Cost-effectiveness acceptability curves. Each line represents the proportion of simulations, for a particular strategy, that are cost-effective, as a function of willingness to pay for health benefits.

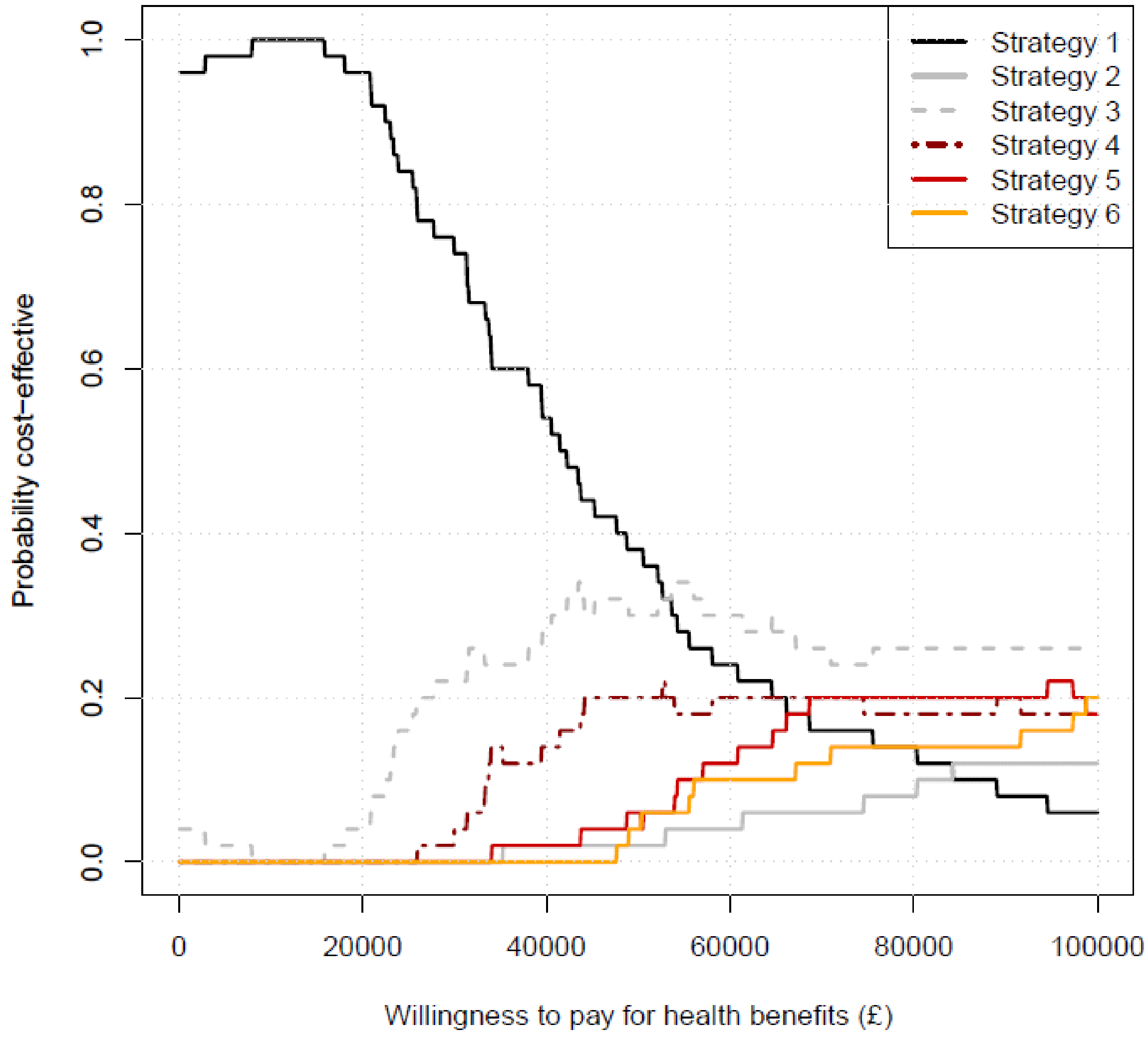

Figure B19. Cost-effectiveness acceptability frontier. Lines depict the strategies with the highest expected net monetary benefit, dependent on the willingness to pay for health benefits, while dotted vertical lines the willingness to pay values at which the decision changes.

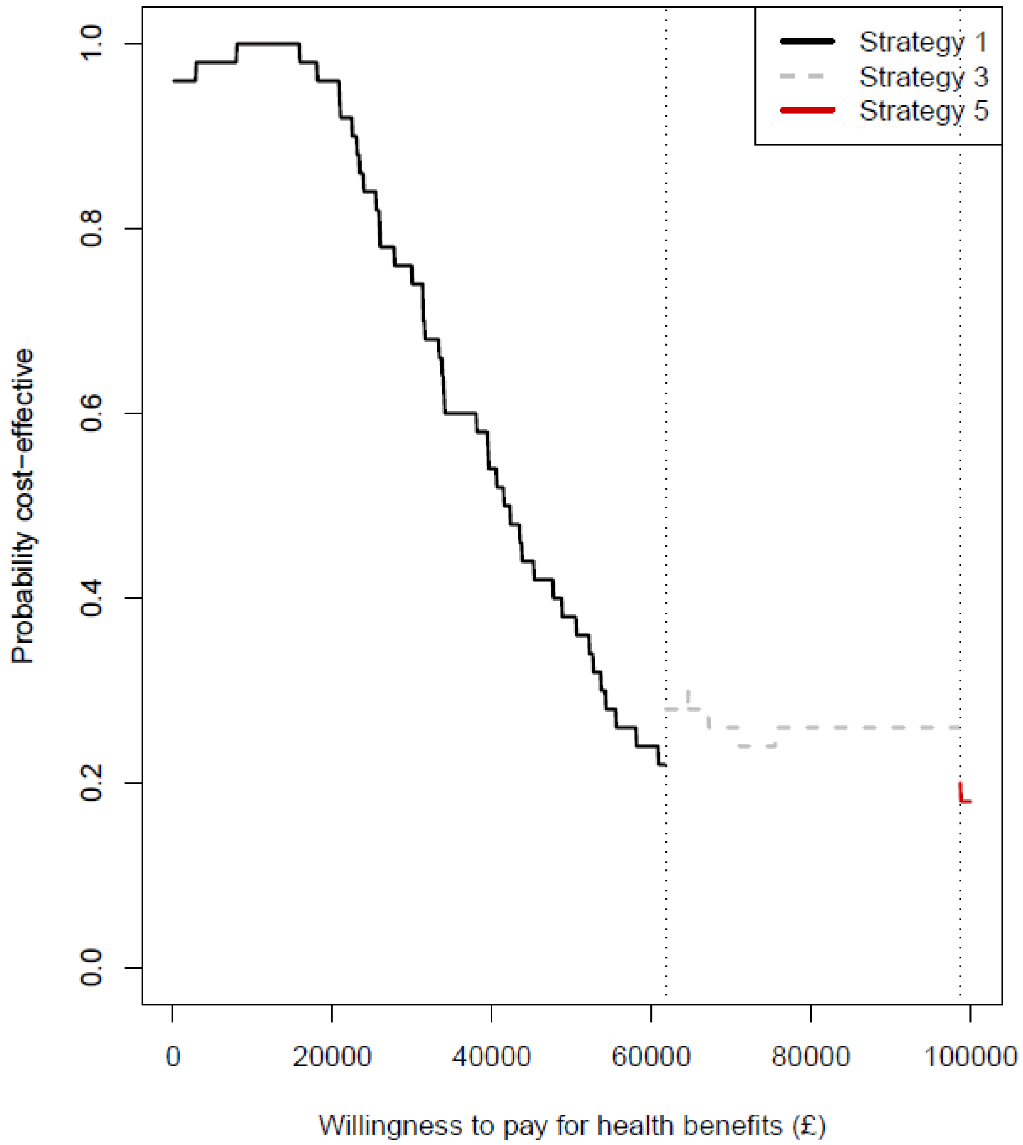

8c Specialist Trusts

1. Baseline analyses

Effectiveness

All strategies isolate greater numbers of patients per 100 bed days in a Specialist setting, compared to in an Acute Trust setting (Figure C1, compared to Figure A1). Very similar numbers of patients are isolated in both Trust types, however the denominator of total patient days is much less over the simulation period in Specialist Trusts due to their smaller size.

The greater number of infections in this setting (Figure C2) compared to an Acute setting (brought about due to the method of transmission parameter estimation – (see appendix 4), translates to increased isolation of clinical cases, leading to greater appropriate and reduced inappropriate isolation usage (Figure C1).

Interestingly, compared to Acute Trusts, the relative difference between strategies 3 and 4 is altered in terms of their ability to correctly isolate patients. These differences are due to the difference in distribution of high risk specialty vs. low risk specialty beds: 44% of beds are in high risk specialties in Specialist Trusts, compared to 16% in Acute Trusts. Therefore in Specialist Trust settings, strategy 3, screening admissions to high risk specialties, will screen a greater proportion of the admission population – leading to greater appropriate and inappropriate isolation. In fact, this different distribution between specialties, coupled with the slightly lower prevalence on admission to Specialist Trusts, strategy 3 actually leads to more inappropriate isolation compared to strategy 4 in this setting (Figure C1), whereas the opposite is seen in Acute Trusts (Figure A1).

There are greater numbers of unisolated MRSA positive bed days per 100 bed days in Specialist Trusts compared to Acute Trusts, due to the greater levels of onward transmission in the Specialist setting (Figure C2 compared to Figure A2), meaning

that any cases missed by the admission screening strategies will go on to generate more cases within the hospital.

While the ordering of strategies remains the same as in Acute Trusts in terms of their effectiveness at reducing transmission, there is overall a greater level of transmission in a Specialist setting (Figure C2). This is due to both the way in which transmission parameters were adjusted by Trust type (see appendix 4), and the greater number of high risk specialty beds in Specialist Trusts (as patients in high risk specialties have higher probabilities of transmission, infection and progression from colonisation to infection than those in low risk specialties, (see appendix 4). This, in turn, leads to more infection events (approximately three times the number in Acute Trusts), translating to an approximate 20% increase in death events per 100 admissions.

Figure C1. Primary isolation usage under each screening strategy, showing appropriate isolation (isolation of patients who are MRSA positive), inappropriate isolation (isolation of MRSA negative patients) and unisolated bed days of MRSA positive patients.

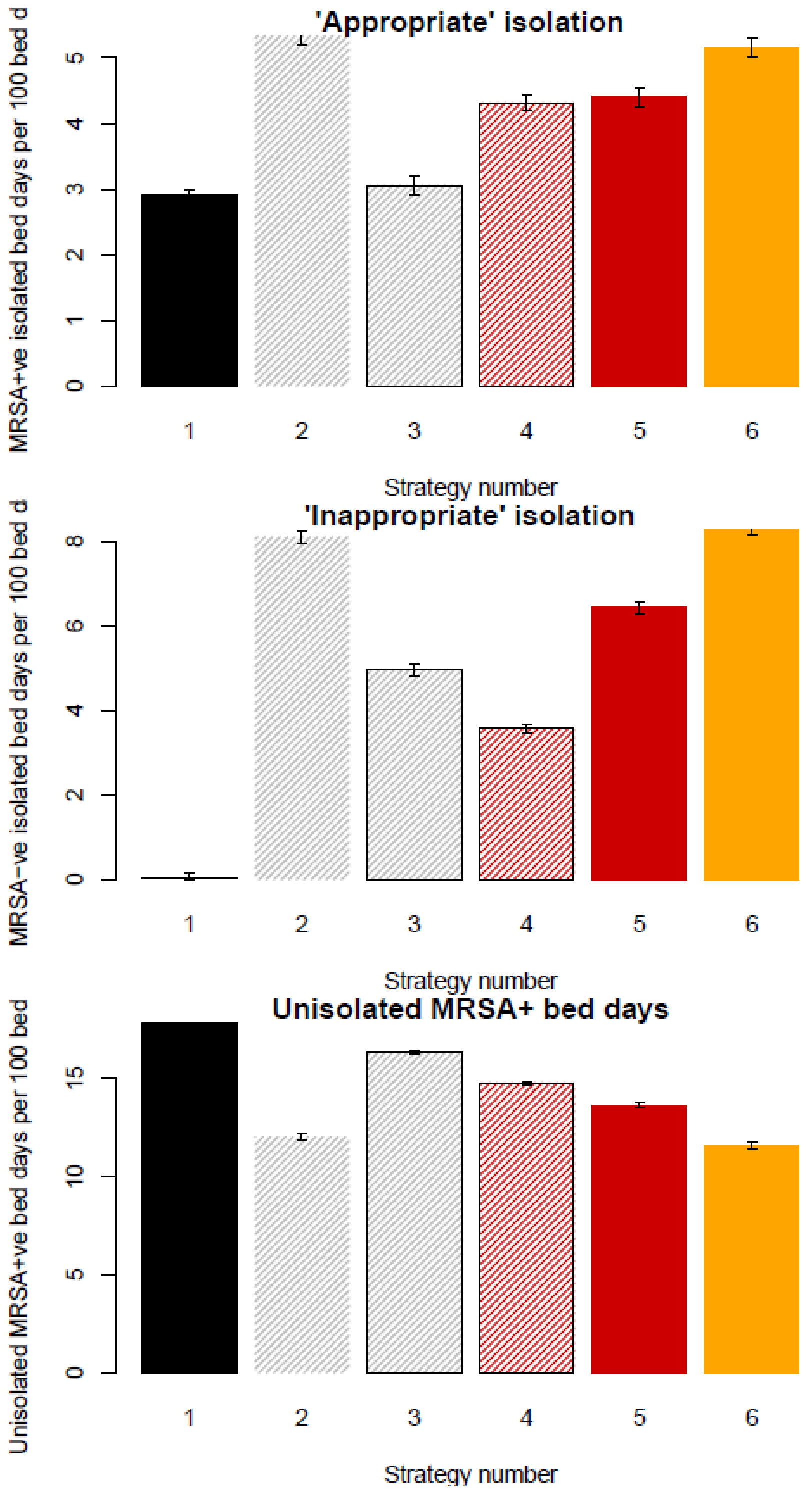

Figure C2. Patient outcomes under each screening strategy, showing new acquisitions of MRSA by hospital patients, total MRSA infections in the hospital, and total deaths (all per 100 admissions).

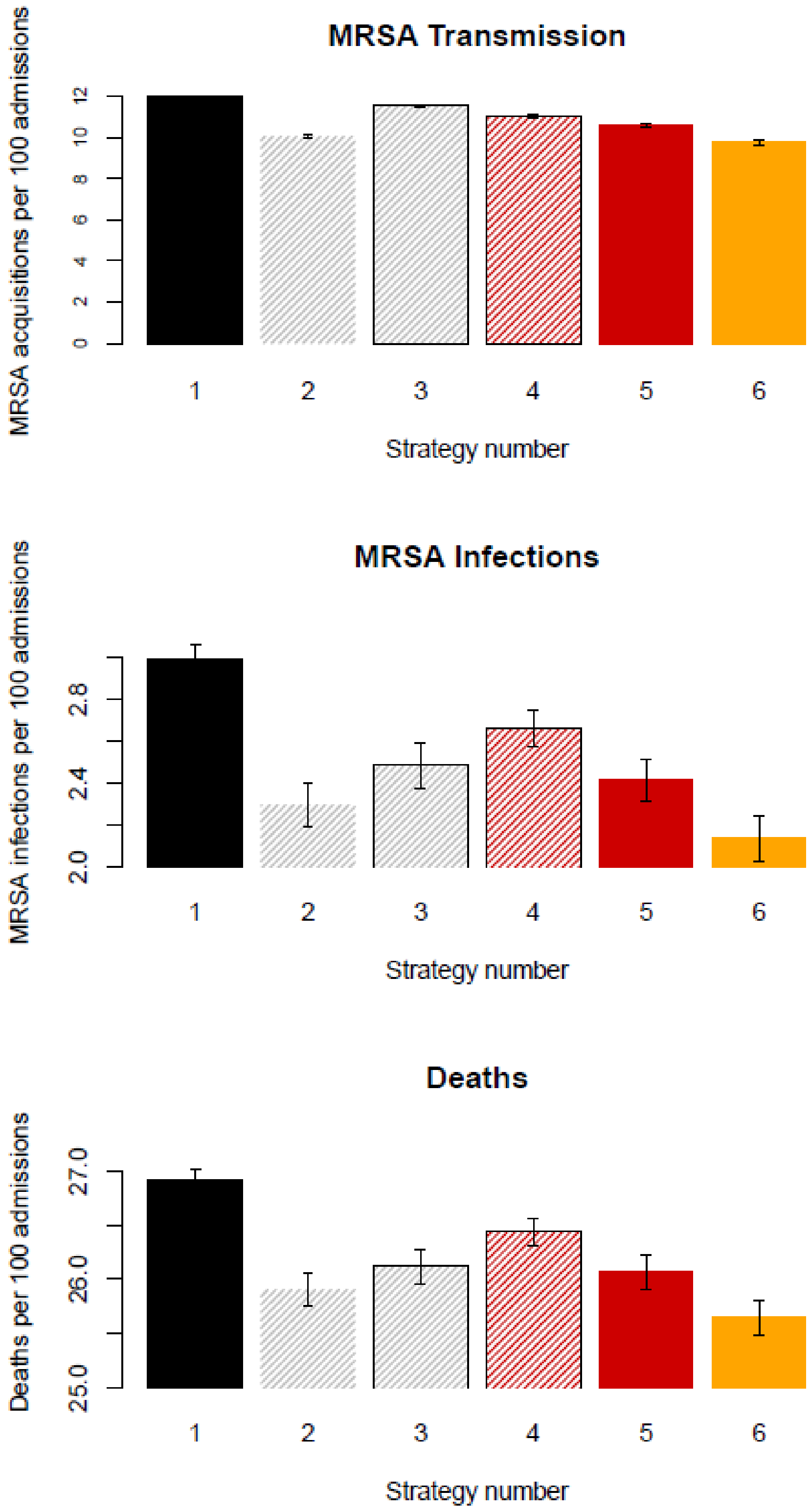

Costs

Overall costs per admission in Specialist Trusts (Figure C30 are less than in acute Trusts (Figure A3). As was the case for Teaching Trusts compared to Acute Trusts, the reduction in bed day costs (the dominant cost) are proportional to the difference in Trust size i.e. bed day costs in Specialist settings are approximately 72% less than in an Acute setting, due to a 72% smaller Trust size. However, the difference in the number of admissions over the simulation period (the denominator in costs/admission) is not proportional to the difference in Trust size. Instead, due to more high risk specialty patients in Specialist Trusts, and therefore altered movement parameters, Specialist Trusts have only 59% fewer admissions. This discrepancy in the degree of change in the numerator vs. the denominator in the cost/admission calculation leads to overall costs/admission being less in Acute Trusts.

Figure C3. Cost per admission for each strategy, presented as total costs and broken down into component parts (note different scales of sub graphs).

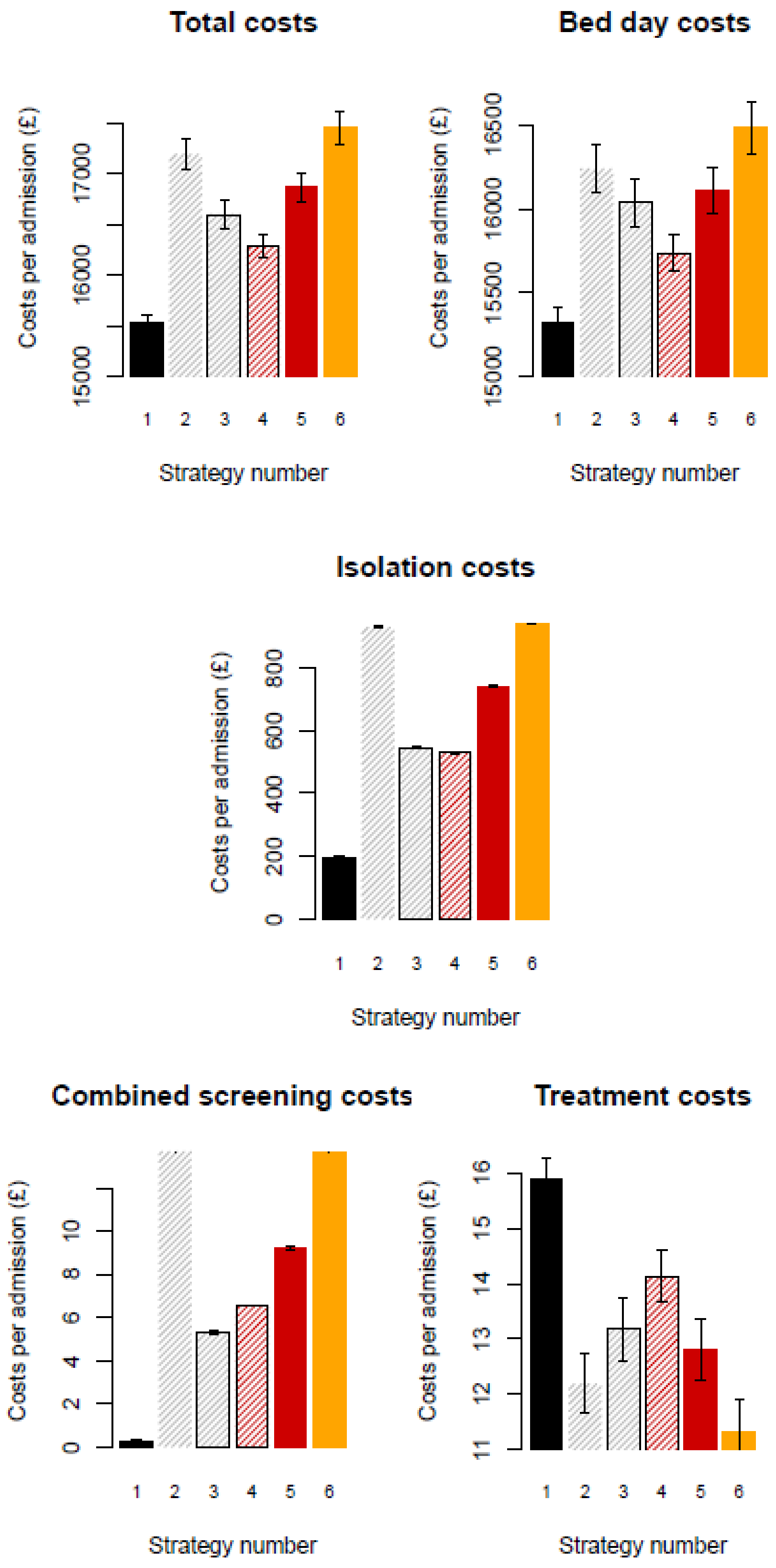

Cost-effectiveness

Comparing incremental costs (Figure C4), shows that while costs remain broadly similar (compared to an Acute setting (Figure A4)), the ordering of strategies shifts slightly. In this setting strategy 3, screening admissions to high risk specialties, becomes a more costly option (compared to the baseline) due to the greater proportion of patients admitted to high risk specialties in this setting.

Incremental health benefits gained for all screening strategies (compared to the baseline 'no screening' strategy, strategy 1) are at least two times greater in a Specialist setting (Figure C4). These QALY gains lead to ICERs below the cost-effectiveness threshold. In this setting strategy 6, screening all patients on admission plus pre-emptive isolation of those patients known to have been previously MRSA positive, leads to the greatest gains in health benefits but is the most costly. It is still, however, cost-effective at £14,324/QALY.

Figure C4. Incremental cost-effectiveness plot comparing each of the screening strategies. Numbers indicate strategy numbers as outlined above. Error bars represent random error brought about by stochasticity in the model and parameter uncertainty, and correspond to plus or minus one standard error.

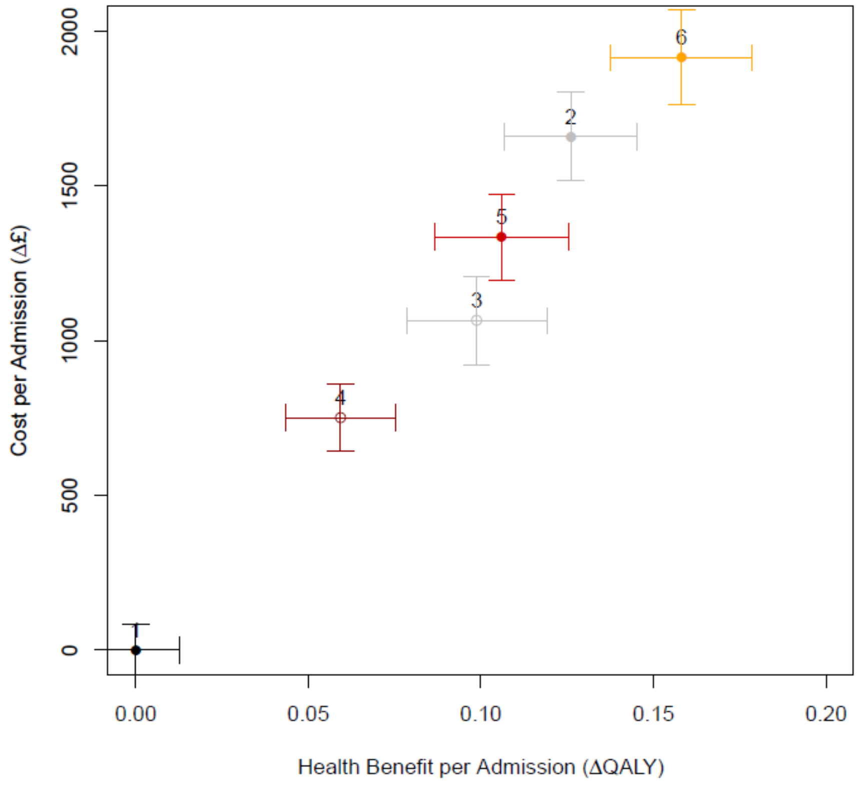

Table C1. Evaluation of cost-effectiveness frontier of strategies.

Move between policies	Cost per admission	QALY per admission	Change in costs, ΔC (compared to previous policy)	Change in effects, ΔE (compared to previous policy)	Difference in Costs / Difference in Effects (ICER, cost/QALY)	Option evaluation
Stay at policy 1	£15,536	7.408665	-	-	-	-
Or move:						
to 3	£16,601	7.507464	£1,064.77	0.098798	£10,777	Cost effective
to 6	£17,451	7.566808	£850.08	0.059344	£14,324	Cost effective

Consideration of uncertainty

The uncertainty in costs and QALYs are represented in Figure C5, and consideration of this uncertainty in strategy comparison is shown in the CEAC (Figure C6). The CEAC demonstrates that the policies are very tightly clustered in this setting, with the probability of any one strategy being the most cost-effective being around only 20% (at the willingness to pay values of between £20,000 and £30,000/QALY). This is reflected in the CEAF (Figure C7) where despite strategy 6, screening all patients on admission plus pre-emptive isolation of those patients known to have been previously MRSA positive, being shown as optimal for all willingness to pay values over approximately £15,000/QALY, the certainty in this decision does not exceed 30% for any willingness to pay.

Figure C5. Simulation results comparing each strategy on a cost-effectiveness plane for a Specialist Trust setting. Each dot represents the mean of 1000 simulation runs for each parameter set. The results of 50 parameter sets for each strategy are plotted, where each parameter set is obtained by taking the mean value for all parameters apart from the effectiveness of the intervention, which is sampled from its probability distribution.

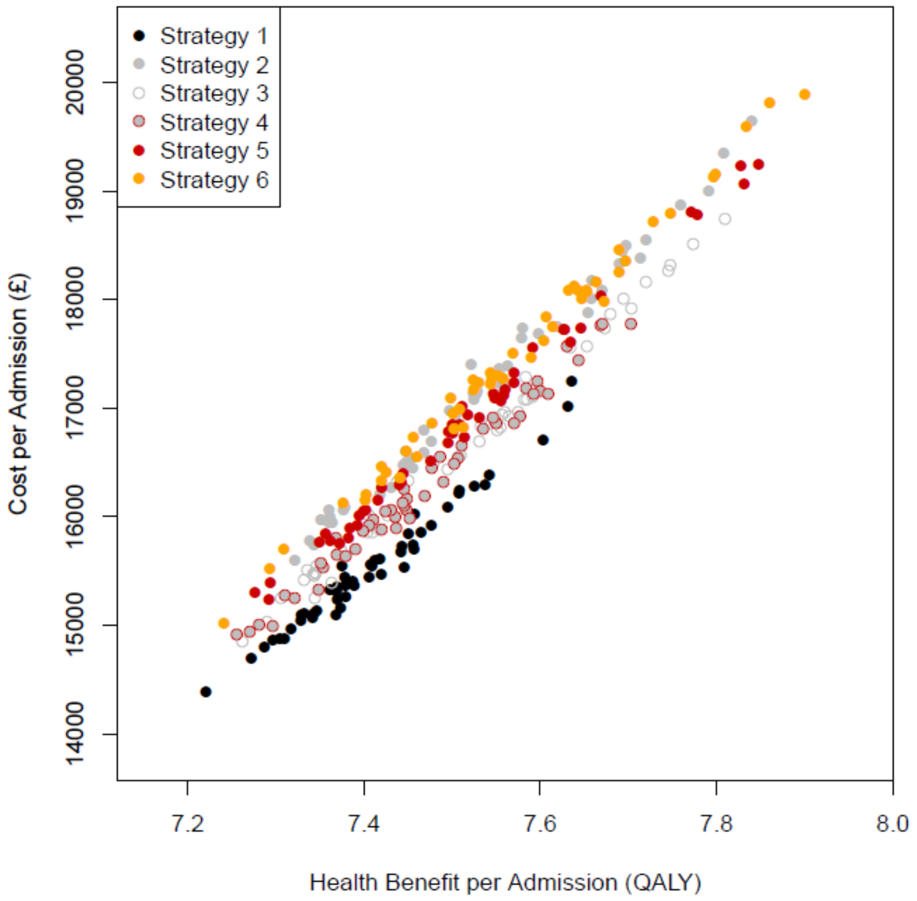

Figure C6. Cost-effectiveness acceptability curves. Each line represents the proportion of simulations, for a particular strategy, that are cost-effective, as a function of willingness to pay for health benefits.

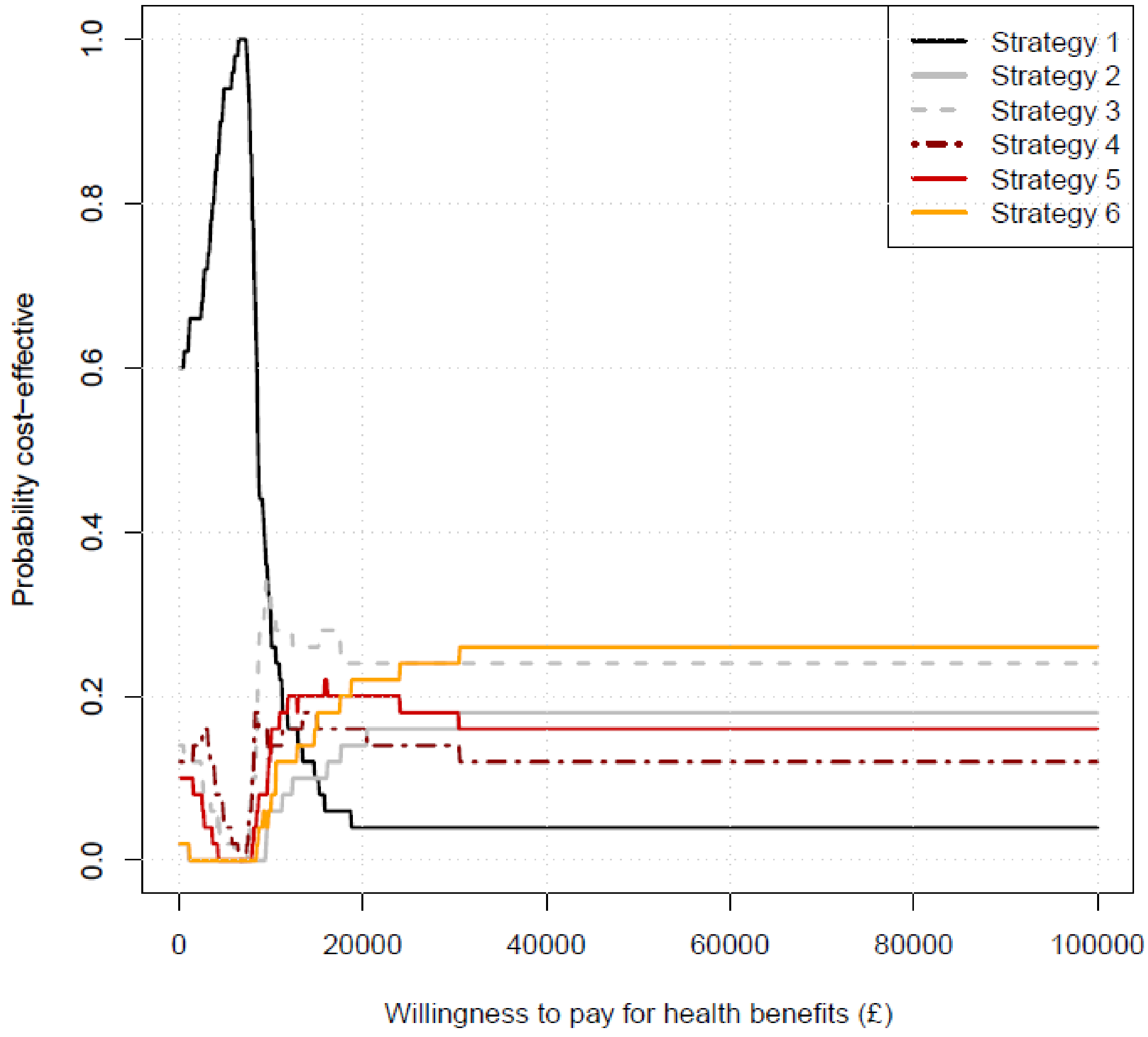

Figure C7. Cost-effectiveness acceptability frontier. Lines depict the strategies with the highest expected net monetary benefit, dependent on the willingness to pay for health benefits, while dotted vertical lines the willingness to pay values at which the decision changes.

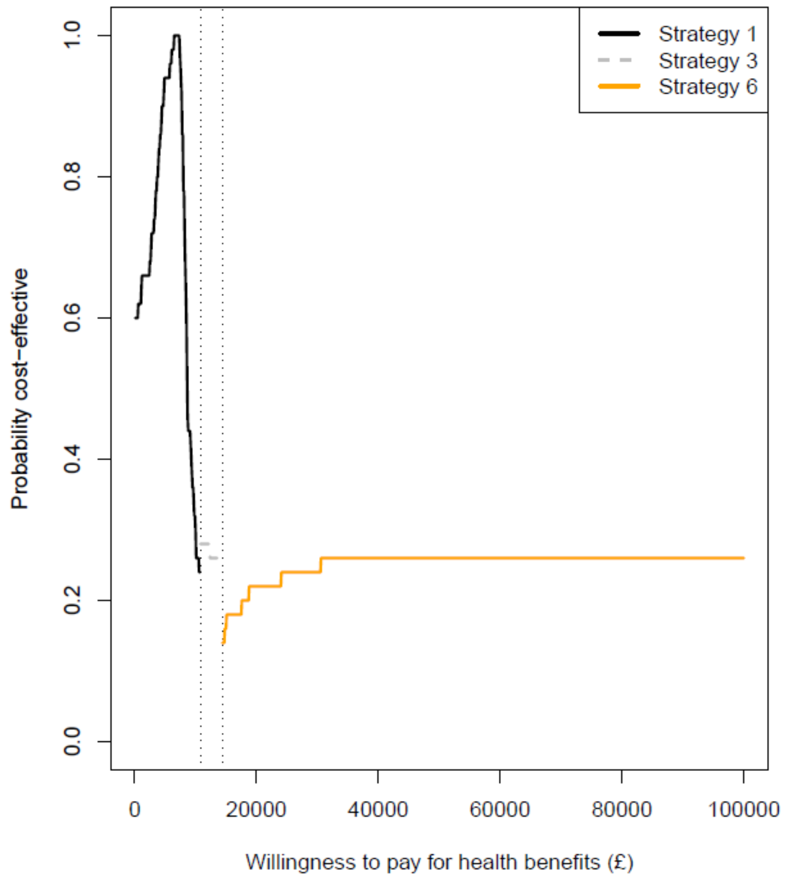

2. Scenario analyses

The following results explore each of the strategies in a Specialist Trust setting in a series of scenario analyses, namely: i) a high prevalence setting (where prevalence is twice that found in the audit for Specialist Trusts: 2.1% compared to 1.04%); and ii) a low prevalence setting (where the prevalence is half that found from the audit for Specialist Trusts: 0.52% compared to 1.04%).

High prevalence setting

In high prevalence setting Specialist Trusts, the relative ability of each of the strategies to effectively identify and therefore isolate (and decolonise) MRSA positive patients (Figure C8) remains the same: the ordering of strategies can be seen to be the same as in an average prevalence setting (Figure C1). However, the overall degree of appropriate isolation increases slightly, due to the higher infectious assault. Conversely, inappropriate isolation decreases slightly, due to the improved positive predictive value of the test under higher prevalence conditions (and due to the isolation of more clinical cases). Unisolated MRSA positive bed days per 100 bed days increase marginally due to more missed cases, and more onwards transmission and infections (see Figure C9 for transmission levels).

Only very slight increases ion transmission are seen in response to the increase in colonised admissions to the hospital, compared to baseline prevalence conditions (Figure C9, compared to Figure C2). Translating also to slight increases in the number of infection and death events per 100 bed days. It is worth noting that, compared to the other strategies, strategy 2, screening all patients on admission, appears to fare slightly better in a higher prevalence setting in terms of reduction in infection events.

As might be expected, total costs per admission are slightly reduced in a higher prevalence setting (Figure C10, compared to Figure C3) due to all strategies having the ability to prevent more infection events and thus produce cost-savings.

Figure C8. Primary isolation usage under each screening strategy, showing appropriate isolation (isolation of patients who are MRSA positive), inappropriate isolation (isolation of MRSA negative patients) and unisolated bed days of MRSA positive patients.

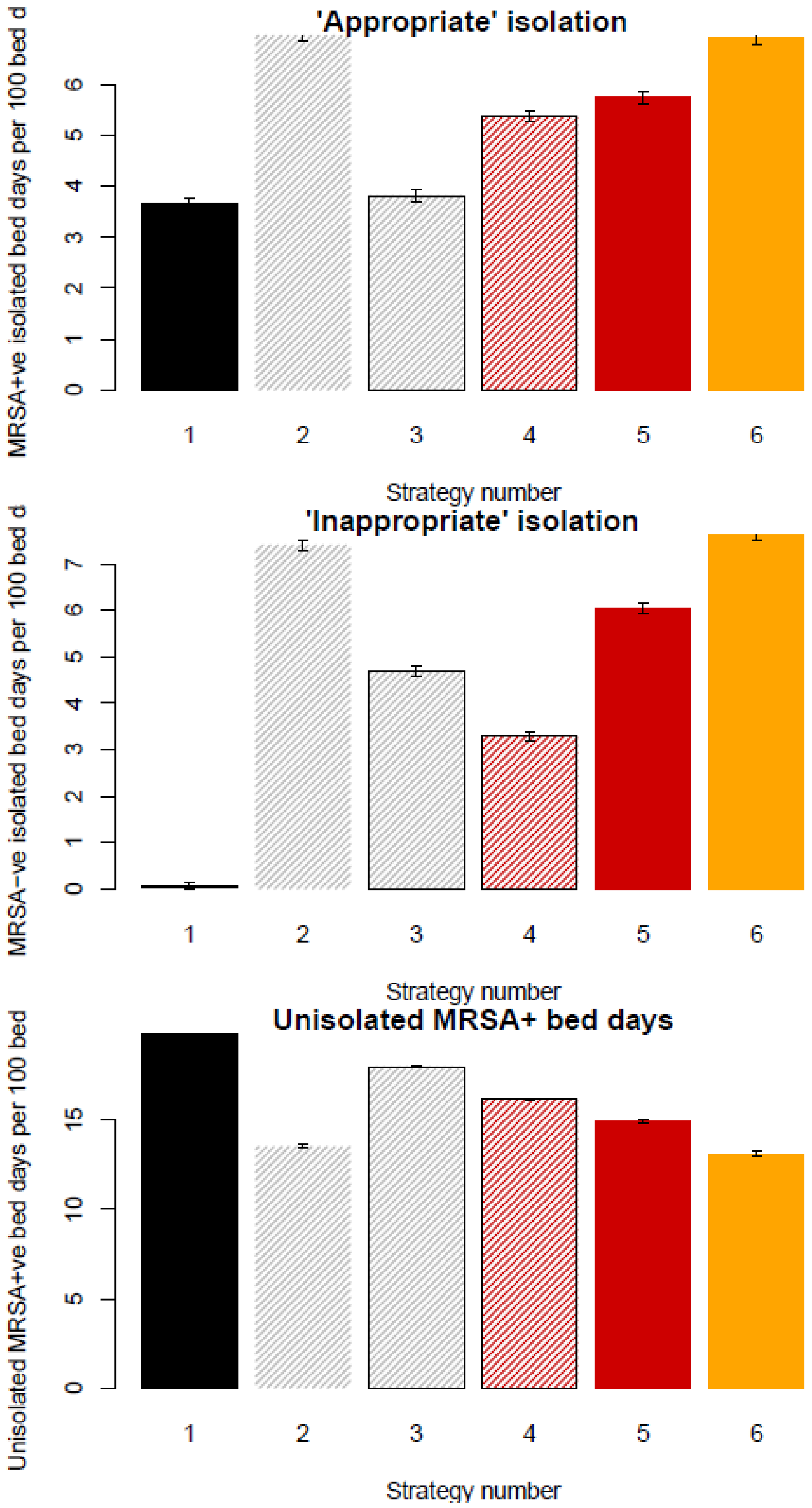

Figure C9. Patient outcomes under each screening strategy, showing new acquisitions of MRSA by hospital patients, total MRSA infections in the hospital, and total deaths (all per 100 admissions).

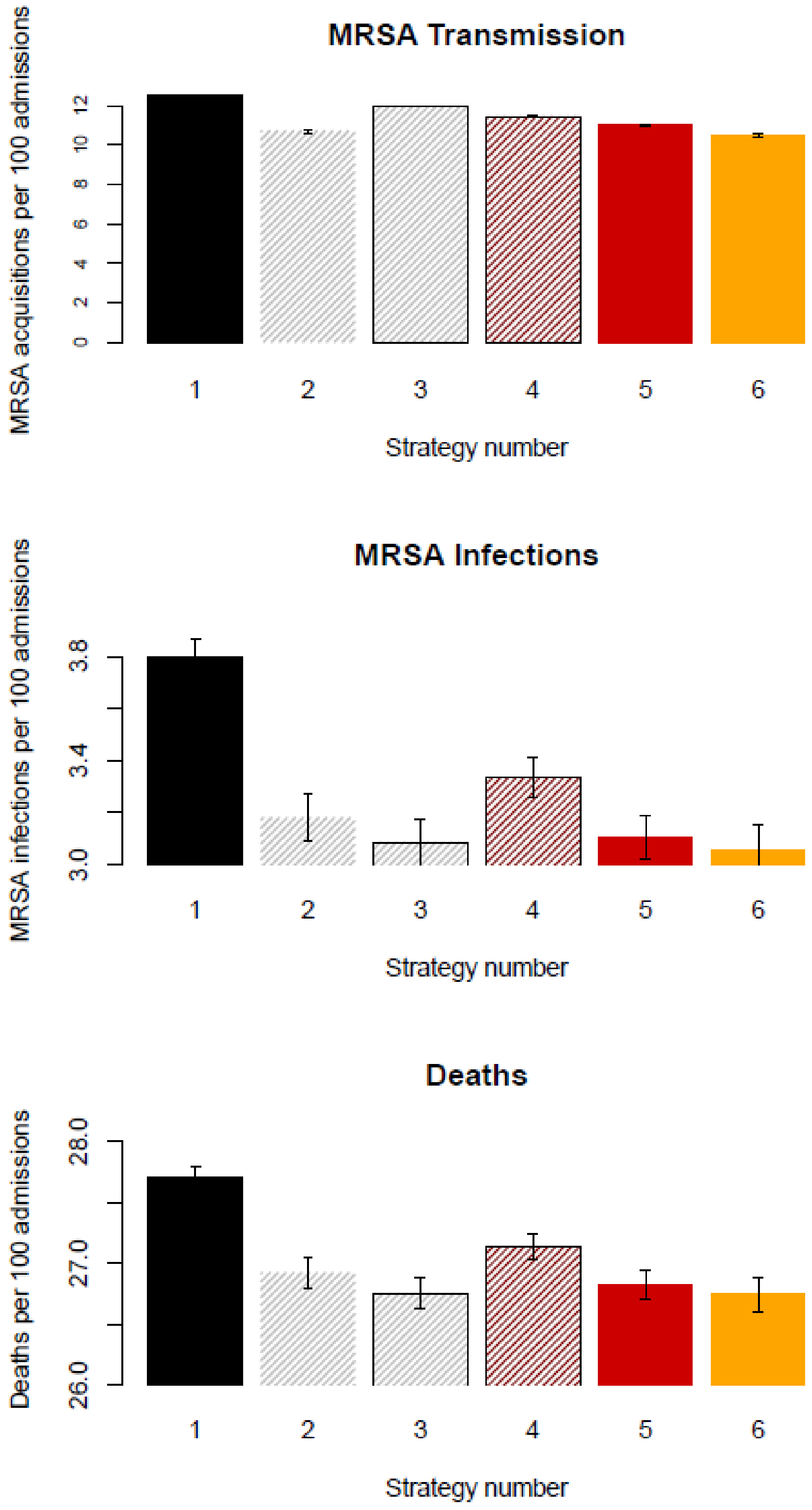

Figure C10. Cost per admission for each strategy, presented as total costs and broken down into component parts (note different scales of sub graphs).

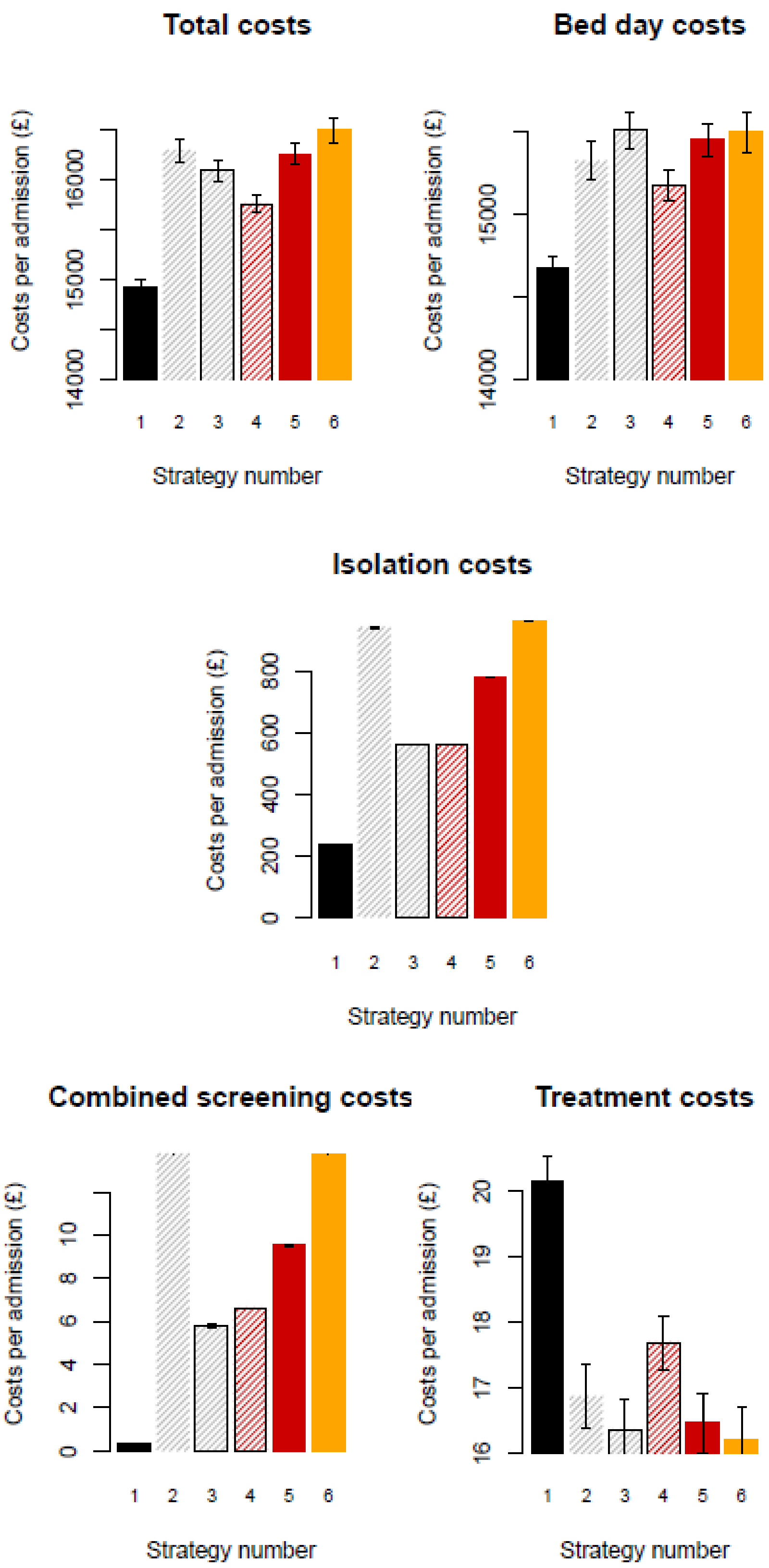

Putting these costs and effects together for a high prevalence setting, and comparing strategies to the baseline ‘no screening; approach (strategy 1), the incremental cost-effectiveness plot (Figure C11) demonstrates that strategies are much more tightly clustered in terms of both incremental costs and incremental health benefits than in an average prevalence setting. Due to this tight clustering, the choice between strategies becomes more difficult. While evaluation of the cost-effectiveness frontier (Table C2) shows strategy 3, screening patients admitted to high risk specialties, is cost-effective at £9,745/QLAY (slightly better value than in a baseline prevalence setting) the CEAC (Figure C12) demonstrates the uncertainty in this decision. Taking this uncertainty into account, the optimal policy, i.e. the policy that yields the greatest monetary net benefits, is strategy 3 for all values of willingness to pay above approximately £10,000/QALY.

Figure C11. Incremental cost-effectiveness plot comparing each of the screening strategies. Numbers indicate strategy numbers as outlined above. Error bars represent random error brought about by stochasticity in the model and parameter uncertainty, and correspond to plus or minus one standard error.

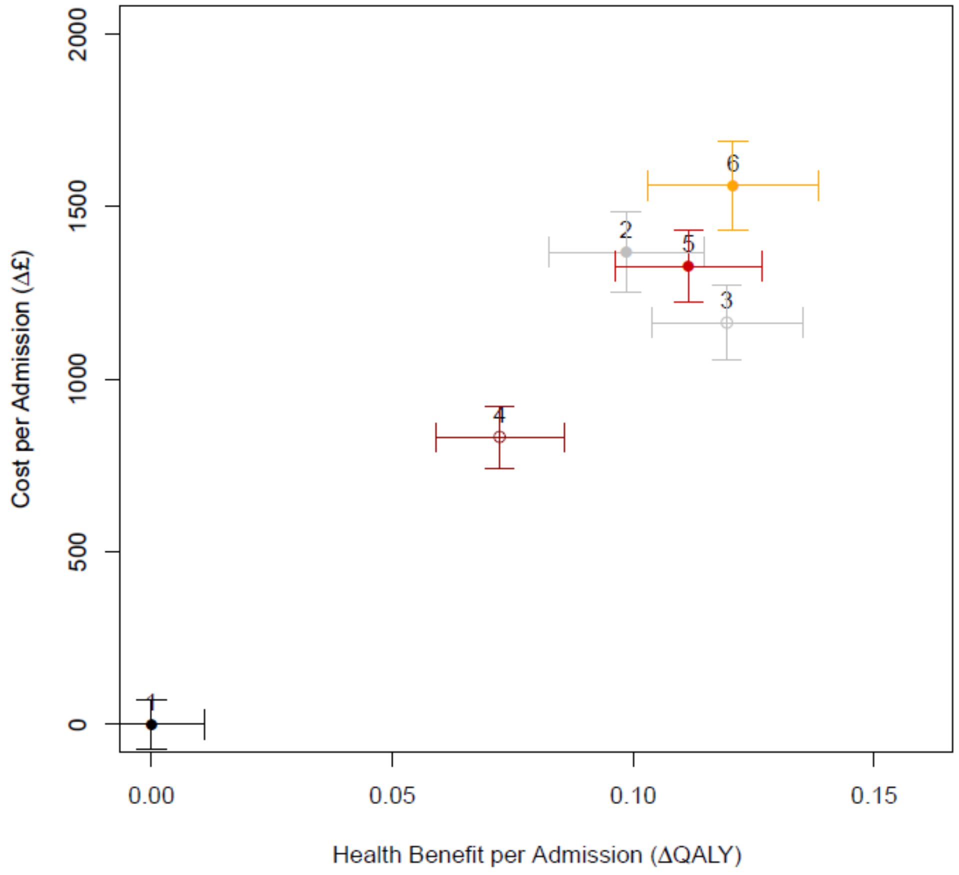

Table C2. Evaluation of cost-effectiveness frontier of strategies.

Move between policies	Cost per admission	QALY per admission	Change in costs, ΔC (compared to previous policy)	Change in effects, ΔE (compared to previous policy)	Difference in Costs / Difference in Effects (ICER, cost/QALY)	Option evaluation
Stay at policy 1	£14,930	7.306292	-	-	-	-
Or move:						
to 3	£16,094	7.425706	£1,163.70	0.119414	£9,745	Cost effective
to 6	£16,492	7.426992	£398.00	0.001285	£309,624	Not cost effective

Figure C12. Cost-effectiveness acceptability curves. Each line represents the proportion of simulations, for a particular strategy, that are cost-effective, as a function of willingness to pay for health benefits.

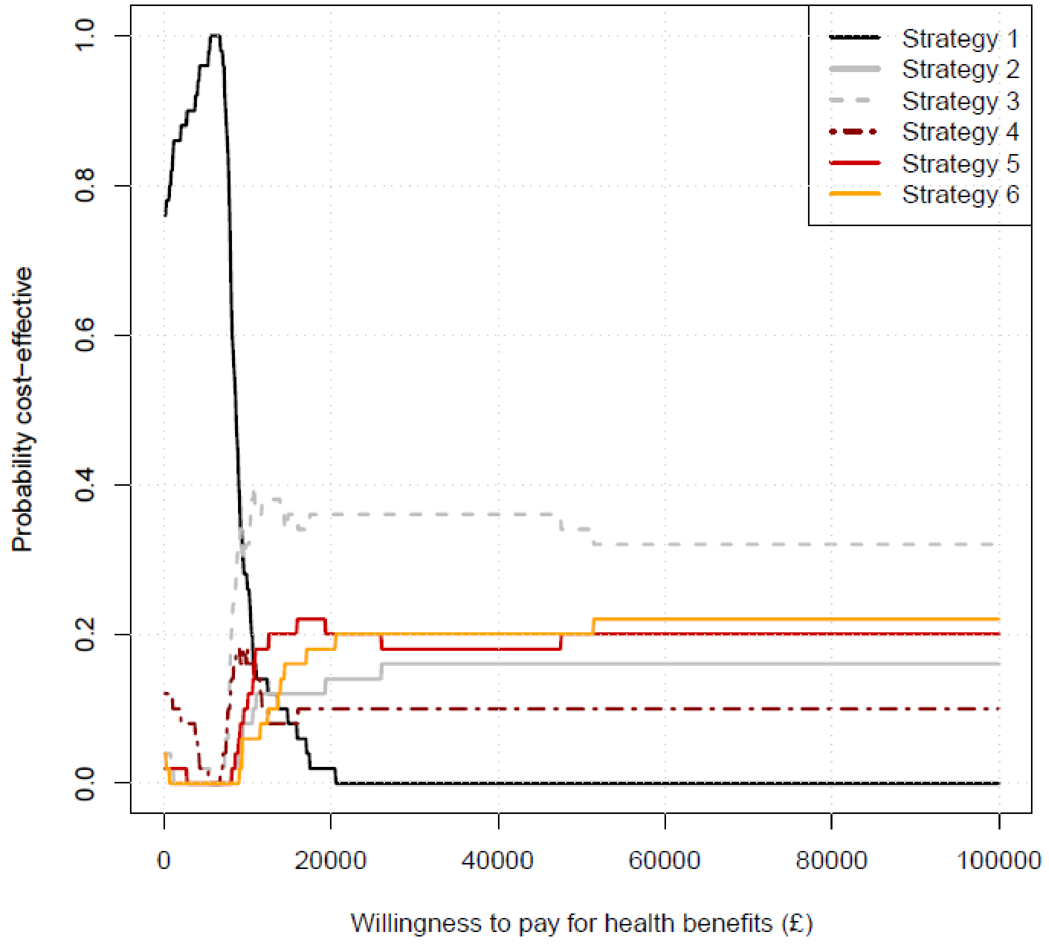

Figure C13. Cost-effectiveness acceptability frontier. Lines depict the strategies with the highest expected net monetary benefit, dependent on the willingness to pay for health benefits, while dotted vertical lines the willingness to pay values at which the decision changes.

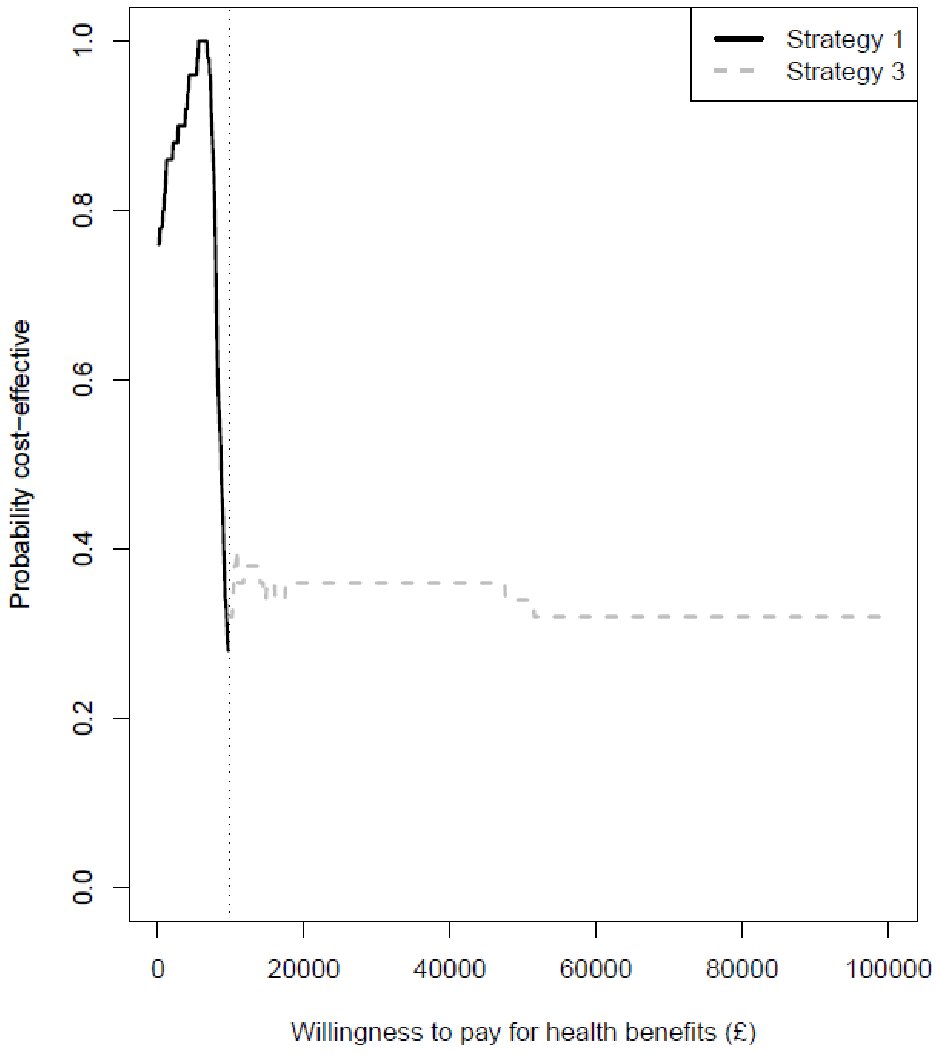

Low prevalence setting

As for the other Trust types, in a low prevalence Specialist Trust, the level of appropriate isolation and MRSA positive bed days decreases compared to an average prevalence Specialist Trust, while inappropriate isolation increases marginally (Figure C14).

While the ordering of strategies, in terms of the level of transmission, remains the same as in the average prevalence setting, under low prevalence conditions absolute numbers of transmission events per 100 bed days are reduced, leading in tern to fewer infection and death events (Figure C15).

Again, as seen for different prevalence scenario analyses for the other Trust types, a lower prevalence leads to higher costs due to the same level of intervention effort leading to the identification of fewer MRSA positive patients and therefore reduced ability to prevent infections and provide cost-savings (Figure C16).
Overall, these small differences in effects and costs leads to similar incremental cost –effectiveness values in a low prevalence Specialist Trusts (Figure C17) as seen in an average prevalence Specialist Trust (Figure C4). However, a slight shift in the ordering of policies means that while strategy 3, screening of high risk specialty admissions, remains cost-effective at £10,566/QALY (albeit at a slightly higher cost/QALY than in an average prevalence setting), strategy 2, screening of all patients, becomes part of the cost-effectiveness frontier and cost-effective (Table C3).

Indeed, strategy 2 is shown to be the optimal options for all willingness to pay values above approximately £15,000/QALY (figure C19), but the uncertainty in this decision is high (Figure C18) with the probability of any of strategies 2,3,5 or 6 having an approximately 25% chance of being the most cost-effective within the usual NHS willingness to pay range (£20,000-£30,000/QALY).

Figure C14. Primary isolation usage under each screening strategy, showing appropriate isolation (isolation of patients who are MRSA positive), inappropriate isolation (isolation of MRSA negative patients) and unisolated bed days of MRSA positive patients.

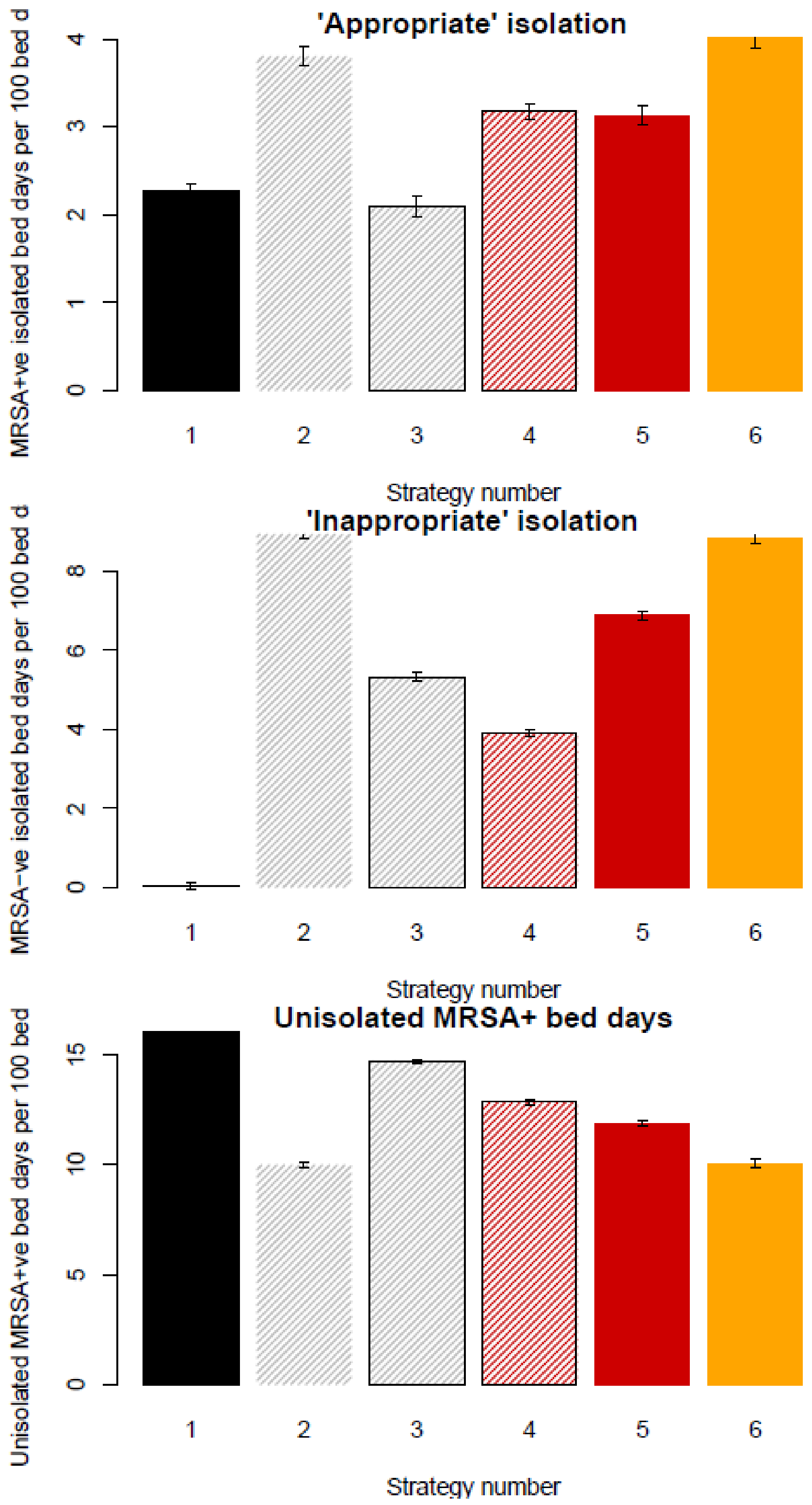

Figure C15. Patient outcomes under each screening strategy, showing new acquisitions of MRSA by hospital patients, total MRSA infections in the hospital, and total deaths (all per 100 admissions).

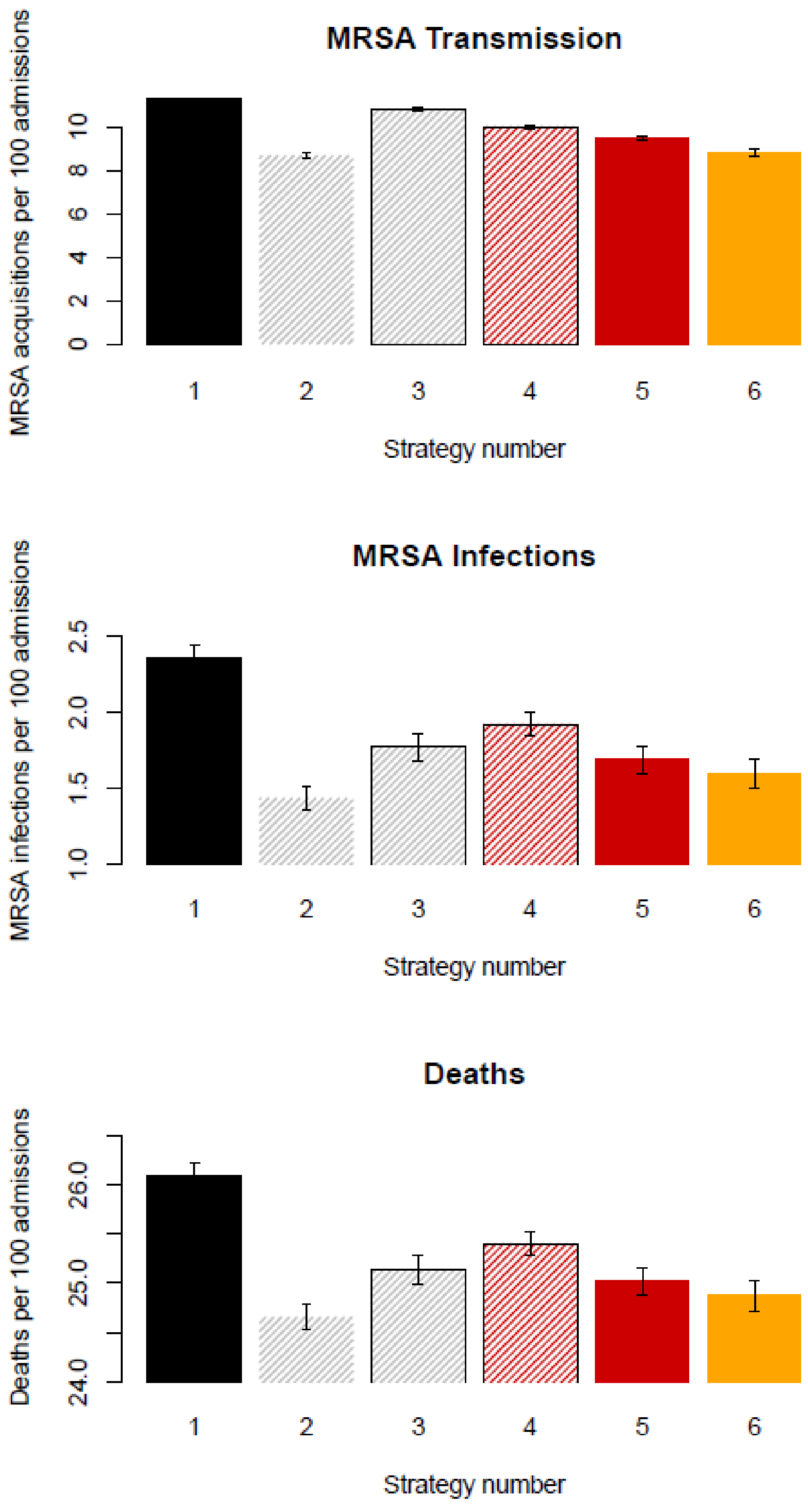

Figure C16. Cost per admission for each strategy, presented as total costs and broken down into component parts (note different scales of sub graphs).

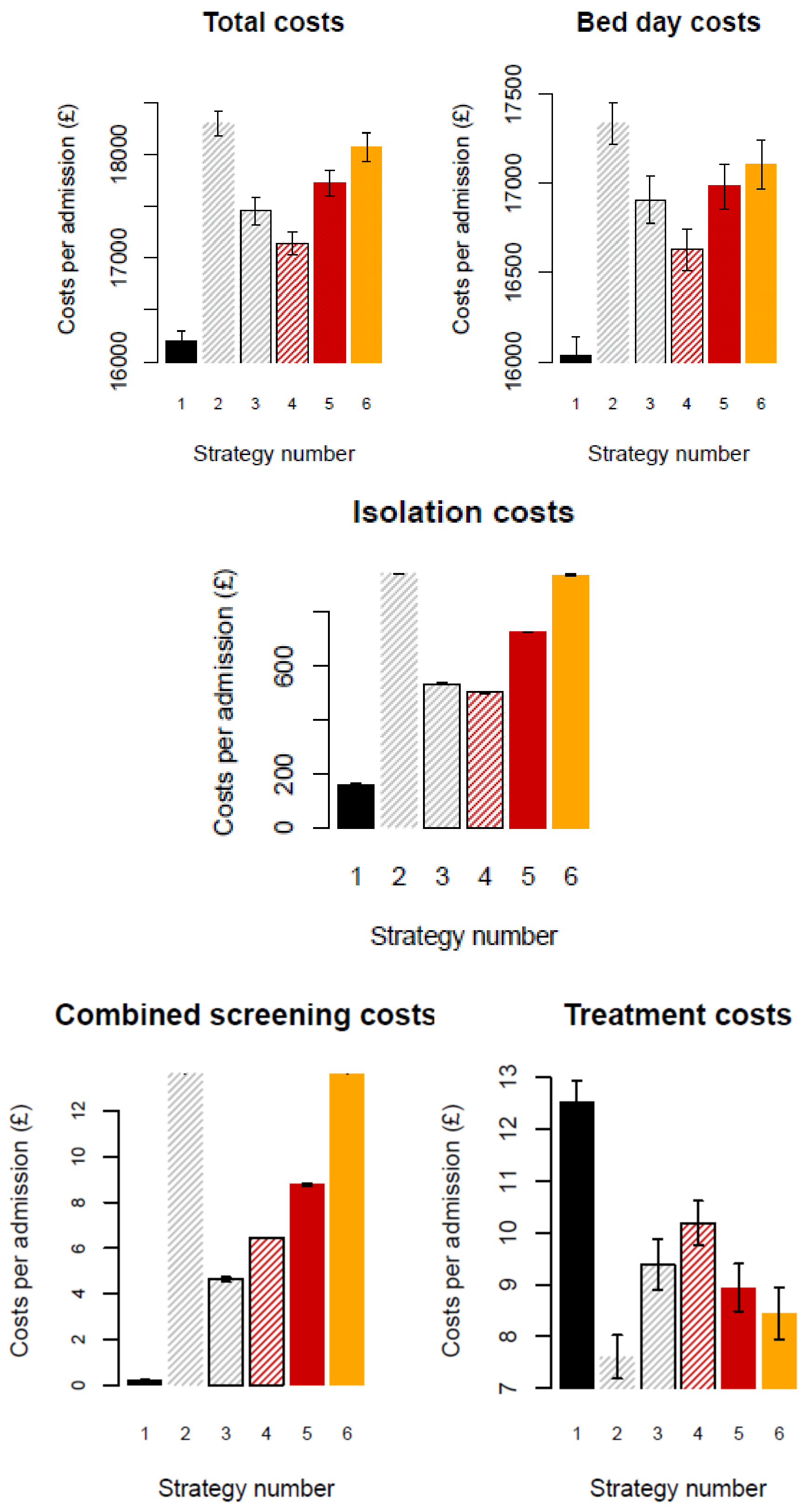

Figure C17. Incremental cost-effectiveness plot comparing each of the screening strategies. Numbers indicate strategy numbers as outlined above. Error bars represent random error brought about by stochasticity in the model and parameter uncertainty, and correspond to plus or minus one standard error.

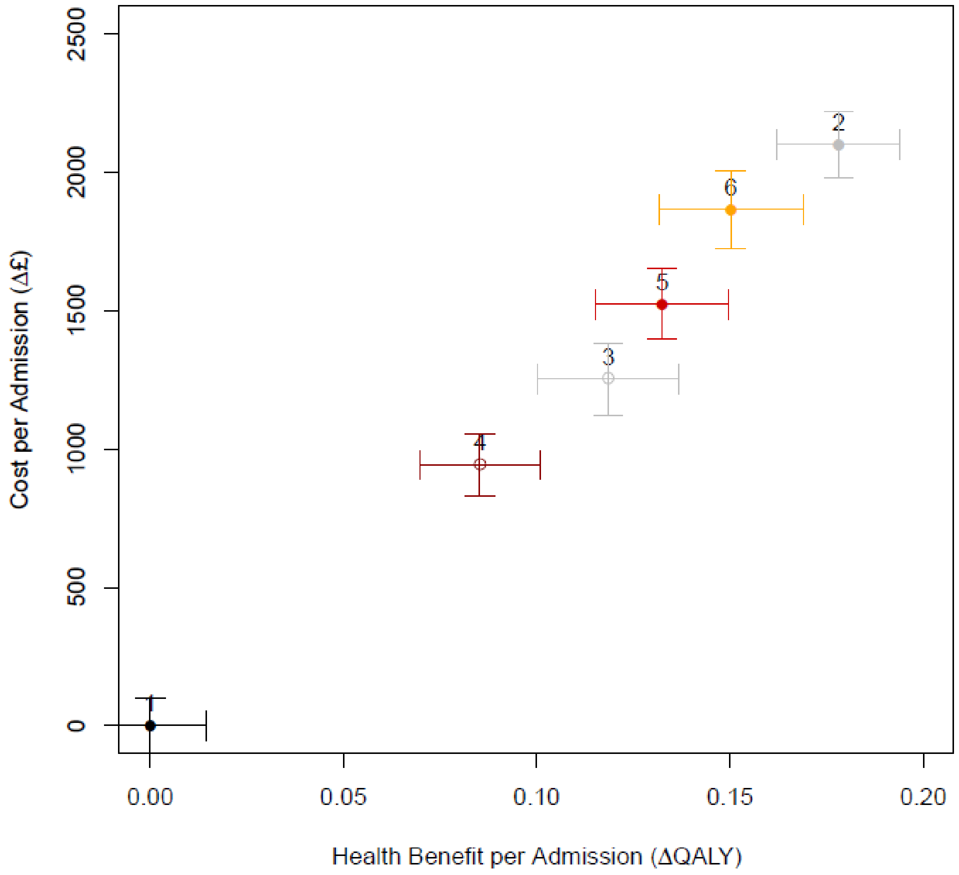

Table C3. Evaluation of cost-effectiveness frontier of strategies.

Move between policies	Cost per admission	QALY per admission	Change in costs, ΔC (compared to previous policy)	Change in effects, ΔE (compared to previous policy)	Difference in Costs / Difference in Effects (ICER, cost/QALY)	Option evaluation
Stay at policy 1	£16,205	7.51243	-	-	-	-
Or move:						
to 3	£17,457	7.630957	£1,252.36	0.118527	£10,566	Cost effective
to 2	£18,304	7.690535	£847.43	0.059578	£14,224	Cost effective

Figure C18. Cost-effectiveness acceptability curves. Each line represents the proportion of simulations, for a particular strategy, that are cost-effective, as a function of willingness to pay for health benefits.

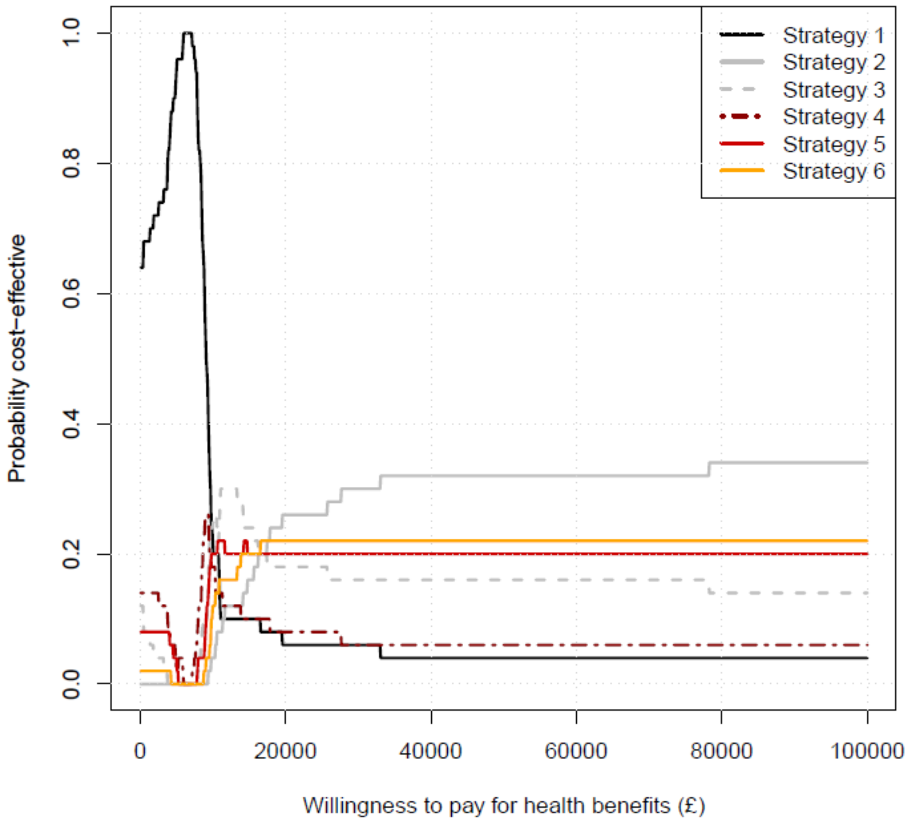

Figure C19. Cost-effectiveness acceptability frontier. Lines depict the strategies with the highest expected net monetary benefit, dependent on the willingness to pay for health benefits, while dotted vertical lines the willingness to pay values at which the decision changes.

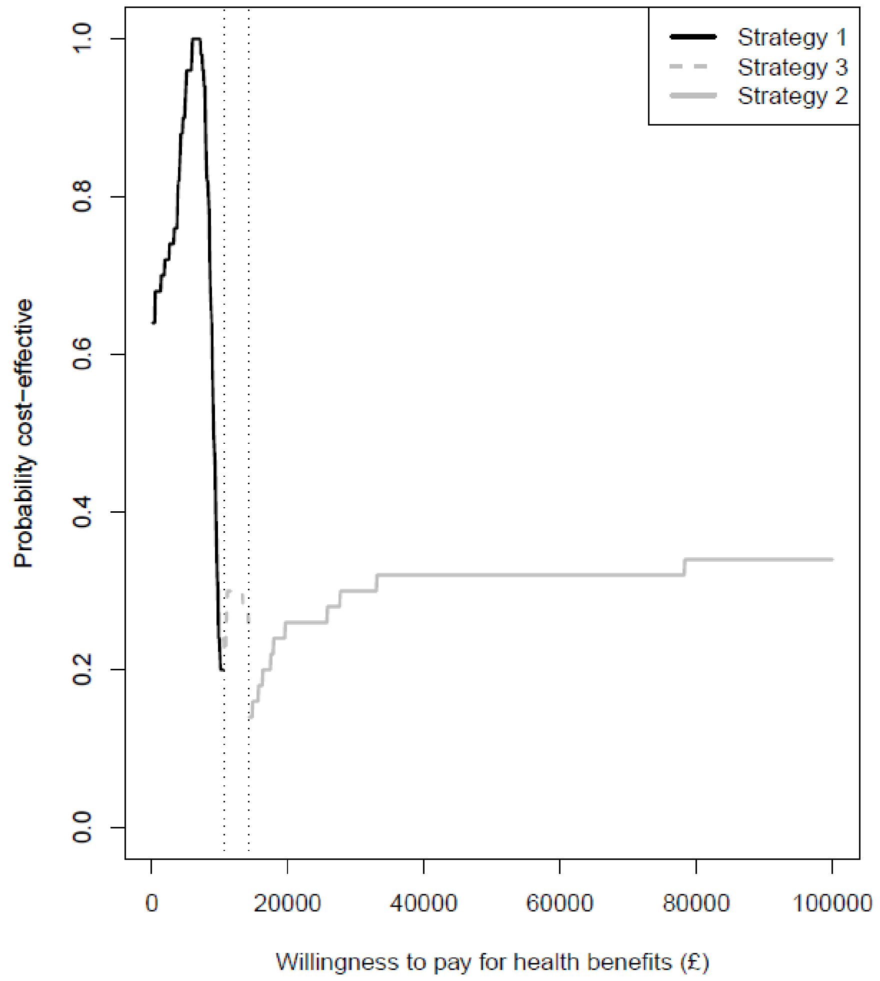

Supplement: File S1 — The NOW questionnaire. (PDF) [file pone.0074219.s001.pdf]
