# Supplementary material for: The National One Week Prevalence Audit of Universal Meticillin-Resistant Staphylococcus aureus (MRSA) Admission Screening 2012
Source: PLoS One. 2013 Sep 12;8(9):e74219. doi: 10.1371/journal.pone.0074219 (PMC3772122; doi:10.1371/journal.pone.0074219)
Supplement: File S2 — Screening, isolation & decolonisation policies/practices, and laboratory methods. (DOC) [file pone.0074219.s002.doc]

**File S2.*Screening, isolation & decolonisation policies/practices, and laboratory methods.***

*Screening policies:* Following Department of Health advice, 71% of trusts (102/143) exempted dermatology day cases, and 73% (104/143), 78% (112/143) and 76% (107/143) exempted ophthalmic and dental day-cases, low-risk paediatrics or endoscopy cases respectively.

*Sites swabbed:* All 143 trusts performed nasal swabbing and 132/143 (92%) also swabbed wounds/devices plus or minus groin or perineum.

*Isolation and decolonisation policies (Table 4):* All patients were pre-emptively isolated in 1.4% of trusts (2/143) and in 7% of trusts (10/143) there was no pre-emptive isolation. Pre-emptive decolonisation was reported by 10% (14/143) for all patients and was not used at all in 34% (49/143) of trusts.

*First-line decolonisation regimens* consisted of nasal mupirocin in 132/143 (92%) trusts, combined with chlorhexidine washes in 72/143 (50%) trusts, with octenidine washes in 51/143 (36%) trusts or with triclosan in 17/143 (12%) of trusts.

*Laboratory methods:* Broth enrichment was the routine technique in 15/142: 11%, responding trusts (emergency screens) and in 14/ 141:10% of trusts (elective screens). Samples were processed within the trust in 122/143 (85%) of cases, with 18 trusts (13%) sending samples to another trust for processing.

In total, 30 trusts used PCR for emergency and/or elective screening of selected patient groups, 25/30 (83%) processing samples in a central laboratory, two of which used point of care testing and 1 of which used a hot-laboratory. PCR tests were run daily in 8 out of 20 responding trusts (40%), twice daily in 8 (40%) and more than twice daily in the remaining four (25%) trusts. Admission swabs were tested separately in 59% of trusts (85/143) rather than pooled.
